# Manipulating Terminal Iron-Hydroxide Nucleophilicity through Redox

**DOI:** 10.1021/jacs.5c20166

**Published:** 2026-01-16

**Authors:** Jeewhan Oh, Kurtis M. Carsch, Shao-Liang Zheng, Theodore A. Betley

**Affiliations:** Department of Chemistry and Chemical Biology, 1812 Harvard University, 12 Oxford Street, Cambridge, Massachusetts 02138, United States

## Abstract

We report changes in the reactivity profile of a high-spin, terminal ferrous hydroxo complex (^Em^L)­Fe­(OH) as a function of oxidation states (i.e., Fe^II^/Fe^III^). The terminal, high-spin Fe–OH adducts were isolated within a sterically hindered dipyrrin ligand scaffold. In the ferrous state, (^Em^L)­Fe­(OH) exhibits nucleophilic reactivity toward carbon-based electrophiles (e.g., CS_2_, CO_2_, nitrile, isocyanate), highlighted by the reversible capture of CO_2_ to generate (^Em^L)­Fe­(κ^2^-O,O-HCO_3_) (Δ*G*° = −2.0 kcal/mol) both in solution and solid state as characterized by single-crystal X-ray crystallography, ^57^Fe Mössbauer spectroscopy, and IR spectroscopy. We probed the nucleophilic character of ferrous analogues with different terminal ligand motifs (X: −CH_3_, −NH_2_, −F, −SH, −H) through a comparison of their reactivity with CO_2_. In contrast to the nucleophilic character exhibited by (^Em^L)­Fe^II^(OH), its high-spin ferric analogue (^Em^L)­Fe^III^I­(OH) exhibited electrophilic reactivity at the hydroxo ligand, undergoing radical recombination with carboradicals, akin to the radical recombination reactivity observed in hydroxylation from high-valent iron oxenoids. These results highlight the effect of the oxidation level, ligand electronegativity, and basicity on the resulting nucleophilic/electrophilic character of the terminal Fe–X pair.

## Graphical abstract

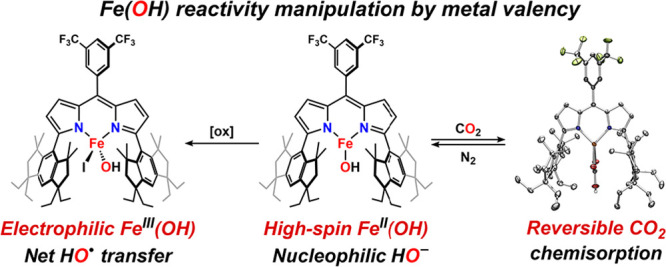

## 1 Introduction

Metal–oxygen bonds are important reactive moieties invoked in both enzymatic and synthetic transformations, including C–H hydroxylation^1,2^ and water oxidation.^3,4^ In particular, iron hydroxo species (Fe–OH) have been extensively investigated in their high-valent states as intermediates and/or products during oxidative C–H activation by iron oxo (Fe=O) complexes.^5–7^ However, due to their intrinsically high reactivity and tendency to undergo dimerization outside the confines of proteins, synthetic terminal iron–hydroxo species have not been extensively studied. Terminal iron–hydroxo complexes can be isolated with the aid of sterically encumbered ligands (Figure 1a)^8–10^ or stabilization through secondary coordination sphere hydrogen-bonding interactions (Figure 1b).^11–18^ Importantly, synthetic high-valent terminal Fe^III/IV^(OH) exhibits electrophilic radical reactivity (Figure 1c) (i.e., radical rebound with carboradicals,^19–28^ as well as H-atom abstraction reactivity^26,28,29^ ), demonstrating their intermediacy during C–H functionalization (Figure 1d,e).^1,5,25,30^

**Figure 1:**
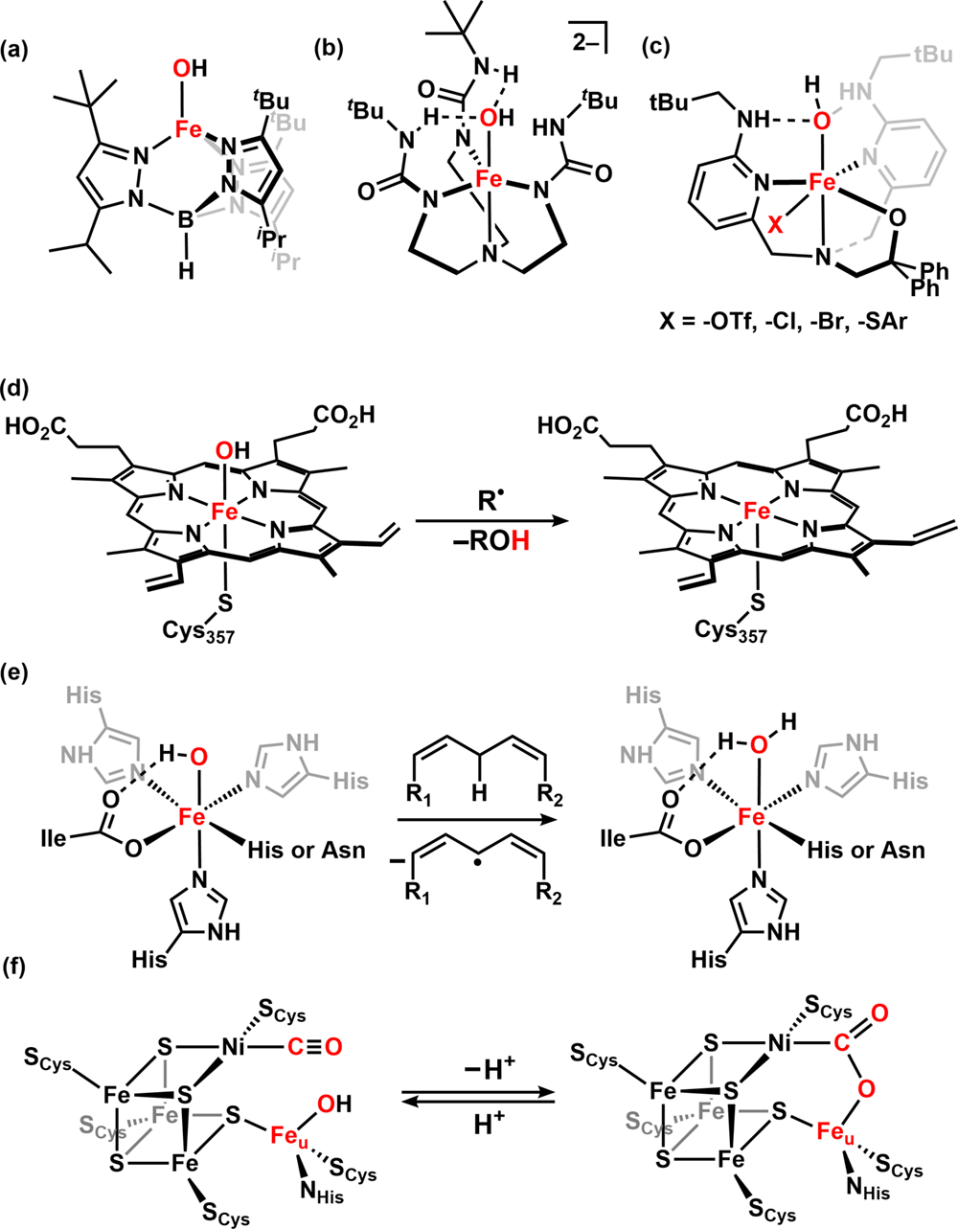
Examples of terminal Fe­(OH) moieties: (a) Fe^II^(OH) in sterically encumbered ligand environment; (b) Fe^II^(OH) stabilized by hydrogen bonding; (c) Fe^III^(OH) showing hydroxyl radical rebound reactivity; (d) enzymatic radical rebound step of C–H hydroxylation by the high-valent Fe­(OH) motif; (e) H-atom abstraction by Fe^III^(OH) of lipoxygenases; and (f) the reversible nucleophilic attack of high-spin Fe_u_^II^(OH) of NiFe-CODHs.

The group transfer reactivity from metal–ligand bonds is directly influenced by the electronic structure of the metal–ligand pair. Previous studies have indicated that the electrophilic group transfer reactivity can be attenuated, or even reversed, to become nucleophilic upon metal oxidation state reduction.^3,31–35^ Indeed, this reactivity reversal is exemplified in distinct enzymatic processes involving Fe–OH moieties: a high-spin ferrous hydroxo (Fe_u_) (Figure 1f) in nickel-containing carbon monoxide dehydrogenases (NiFe-CODHs) exhibits nucleophilic reactivity, mediating reversible CO to CO_2_ conversion,^36,37^ markedly contrasting the electrophilic nature of higher-valent Fe–OH units (Figure 1d,e). Although synthetic advancements have expanded our understanding of the high-valent Fe–OH reactivity in the enzymatic processes, investigations of the Fe^II^(OH) unit have been restricted to exploring the basicity of the hydroxide ligand owing to the propensity for terminal hydroxo ligands to dimerize, often requiring extensive secondary bonding interactions (e.g., hydrogen bonding) to prevent dimerization. Given the participation of the Fe^II^(OH) moiety in enzymatic CO_2_ transformations and its fundamental implications for metal valency effects on group transfer reactivity, exploration of the reactivity profile of a terminal Fe^II^–OH and comparison with higher-valent Fe–OH within a well-characterized synthetic system is desirable.

Herein, we report the characterization of a high-spin terminal ferrous hydroxo complex (^Em^L)­Fe­(OH)^38^ employing a sterically encumbered dipyrrin scaffold that is devoid of secondary-coordination sphere hydrogen-bonding interactions (Scheme 1). Notably, the sterically encumbered environment preserves an unusual three-coordinate ferrous ion. Leveraging this structurally distinct Fe^II^–OH species, we sought to investigate the following: (i) can the terminal Fe^II^(OH) exhibit nucleophilic reactivity toward carbon-based electrophiles, contrasting the electrophilic nature of the Fe^III^(OH) analogues? (ii) How does varying metal valency and ligand electronegativity alter the nucleophilicity of Fe^II^–X species?

**Scheme 1:**
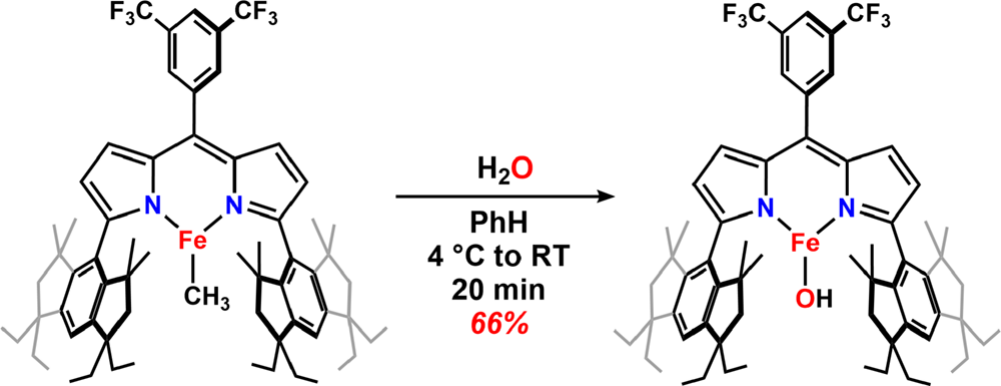
Synthesis of (^Em^L)­Fe­(OH) (**1**)

Our studies reveal the nucleophilic reactivity of this high-spin terminal Fe^II^–OH toward carbon electrophiles. To the best of our knowledge, this study demonstrates the first nucleophilic reactivity of a terminal Fe^II^(OH) motif, contrasting the reactivity profiles of higher-valent Fe^III/IV^(OH) species, which exhibit electrophilic character. The high-spin terminal Fe^II^(OH) further exhibits rapid, reversible CO_2_ capture both in solution and in the solid state. In contrast, one-electron oxidation to the corresponding Fe^III^(OH) species enables hydroxylation of a carboradical akin to other ferric hydroxo species, signifying a reactivity reversal from nucleophilic to electrophilic. Mechanistic analyses obtained from DFT highlight the role of a three-coordinate, electrophilic ferrous center during a nucleophilic hydroxo attack. The detailed investigation of metal valency and ligand electronegativity on the ensuing reactivity will be discussed from the perspective of the manipulation of the group transfer reaction by altering their relative electronegativity between the metal–ligand pair.

## 2 Results

### 2.1 Nucleophilic Reactivity of (^Em^L)­Fe­(OH)

Previously, we reported the synthesis of (^Em^L)­Fe^II^(OH) by the controlled hydrolysis of (^Em^L)­Fe­(CH_3_) (**1**) in benzene solution (Scheme 1).^38^ The hydroxide species (^Em^L)­Fe­(OH) features a three-coordinate Fe^II^ and a terminal hydroxide ligand without hydrogen-bonding interactions, exhibiting thermal stability up to 100 °C in anhydrous benzene solution monitored in a sealed J. Young NMR tube. The sterically encumbered ligand environment is critical to facilitate the isolation of **1**, which features both an electrophilic low-coordinate metal center and a nucleophilic hydroxide ligand. In contrast, using a dipyrrin flanked with quadraphenyl (2,4,6-Ph_3_C_6_H_2_) substituents failed to prevent dimerization, leading to the formation of a Fe_2_(μ-OH)_2_ core.^39^ Transmetalation attempts of (^Em^L)­FeCl with KOH led to the formation of bridging hydroxide species [(^Em^L)­Fe­(OH)]_2_(μ-OH)K (Figure S103), despite using the sterically demanding hydrindacene-substituted ligand. The electrophilic ferrous center is susceptible to ligation by coordinating solvents, as exemplified by the zero-field ^57^Fe Mössbauer spectroscopy (MB) parameters of **1** collected in frozen THF solution (δ, |*ΔE_Q_* | (^mm^/_s_) = 0.97, *1.72*) diverging significantly from the MB parameters of **1** collected in frozen benzene solution (δ, |*Δ* *E*_*Q*_| (^mm^/_s_) = 0.73, *0.78*) (Figure S59). Notably, the MB parameters of **1** in frozen THF are comparable to those of four-coordinate iron dipyrrinato species (vide infra), indicating the ligation of THF.

To probe the nucleophilic character of the terminal Fe^II^(OH), we canvassed the reactivity of (^Em^L)­Fe­(OH) with a variety of carbon electrophiles. We first examined the reactivity of **1** with carbon monoxide, a weak electrophile.^40,41^ Interestingly, **1** is stable under CO (1 atm) in C_6_D_6_ up to 100 °C. Carbon monoxide has been reported to react with low-spin ferrous-parent amido species and iron-alkyl species in both low- and high-spin states through migratory insertion following iron carbonylation to afford iron–acyl complexes.^9,42–46^ The lack of reactivity **1** exhibits toward CO could be attributed to (i) the weak nucleophilicity of the hydroxo in **1** to attack CO directly or (ii) the diminished metal-to-ligand π back-donation capability due to the high-spin electronic configuration,^47^ preventing iron carbonylation.

Given the lack of reactivity of **1** toward CO, we sought to establish the reactivity of **1** toward more electrophilic carbon-based substrates (e.g., nitriles, isocyanates). We assessed the reactivity of **1** with nitriles, typically inert owing to the strong C≡N triple bond but comparably more electrophilic than carbon monoxide (Scheme 2). Heating a benzene solution of **1** and PhCN at 80 °C affords a benzamido complex (^Em^L)­Fe­(κ^2^-N,O-NHC­(O)­Ph) (**2**), as determined by NMR, IR, and MB spectroscopies (δ, |*ΔE*_*Q*_| (^mm^/_s_) = 0.86, *2.05*) (Figure S5), and in the solid state using single-crystal X-ray diffraction (SCXRD) (Figure 2a). Similarly, the addition of 1-adamantyl isocyanate (AdNCO) to **1** in a benzene solution at room temperature (Scheme 2) yielded the carbamato complex (^Em^L)­Fe­(κ^2^-O,O-O_2_CNHAd) (**3**) characterized by NMR, IR (ν­(NH) = 3442 cm^–1^), and MB (δ, |*Δ* *E*_*Q*_| (^mm^/_s_) = 0.94, *1.41*) spectroscopies (Figure S10) and SCXRD (Figure 2b). The successful observation of the addition of hydroxo **1** to nitriles and isocyanates demonstrated the nucleophilicity of the hydroxo ligand in **1**.^48–53^

**Scheme 2:**
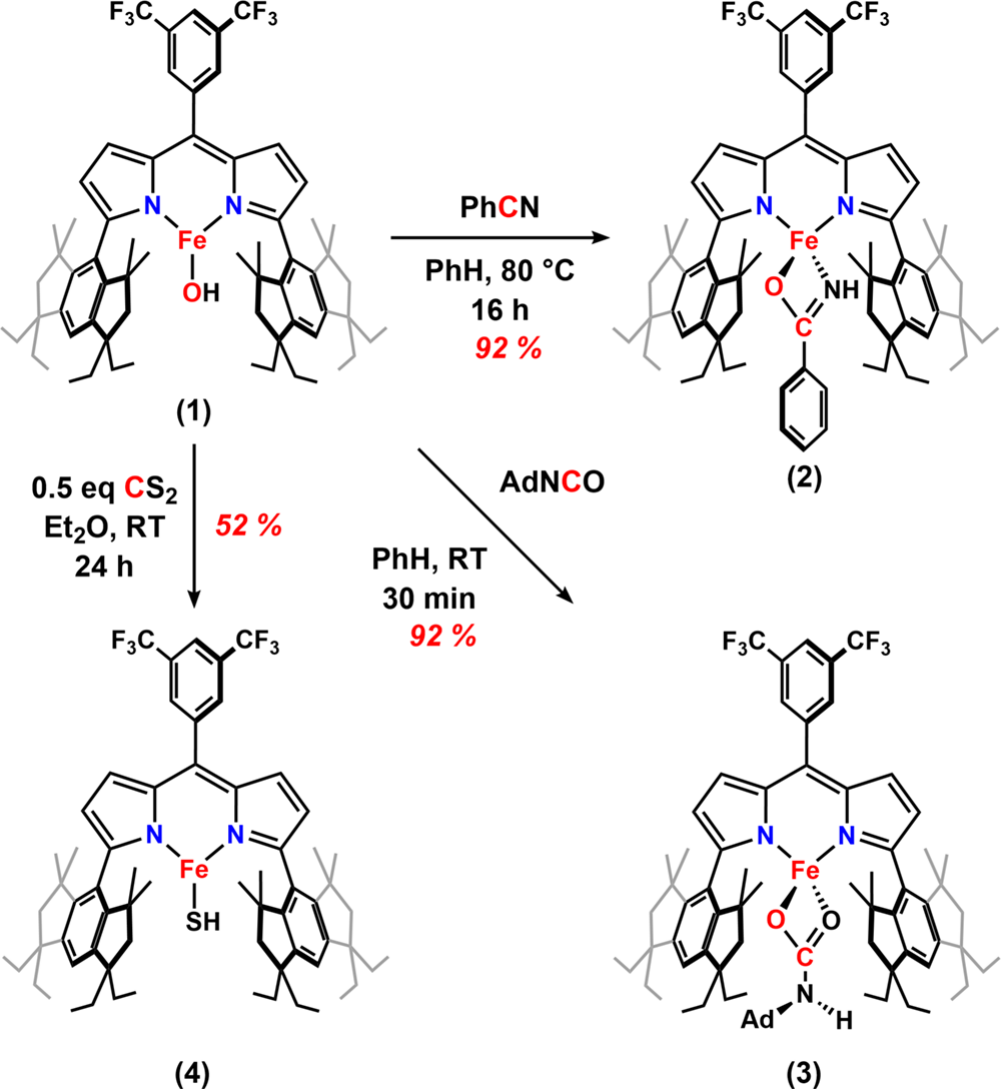
Reaction of **1** with Carbon Electrophiles (PhCN, AdNCO, CS_2_)

**Figure 2:**
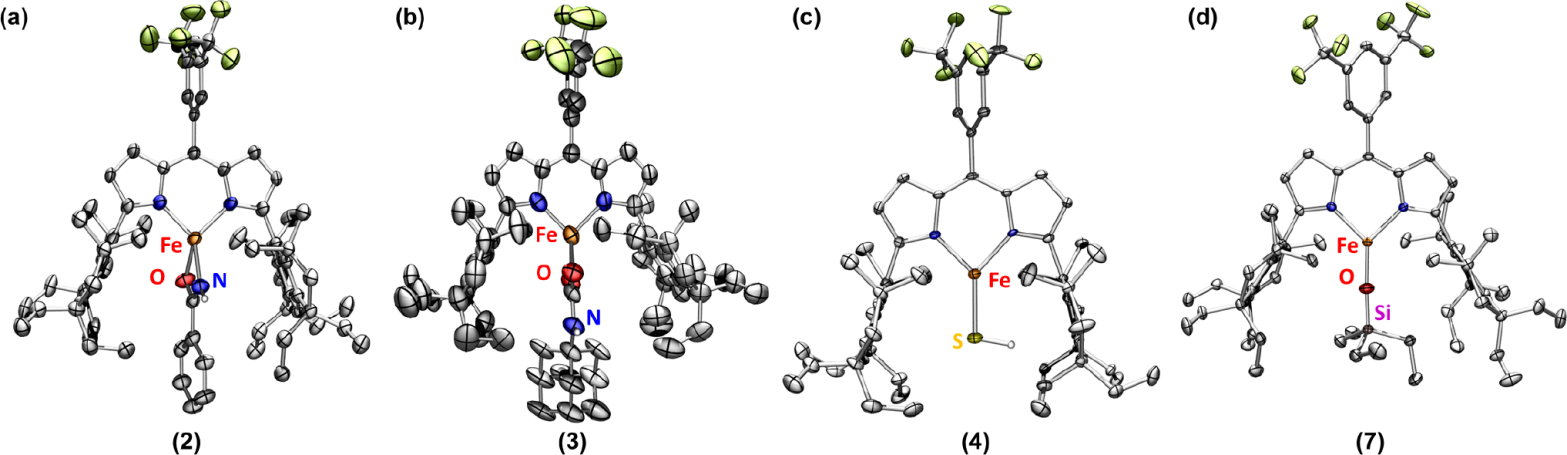
Solid-state structure of (a) **2**, (b) **3**, (c) **4**, and (d) **7** at 100 K with thermal ellipsoids at the 50% probability level (hydrogen atoms except for those located on N and S atoms and solvents are omitted for clarity; Fe, orange; C, gray; N, blue; O, red; F, yellow-green; Si, pink; and S, yellow).

In an attempt to extend the reaction chemistry of hydroxide **1** with additional heterocumulenes, we assessed the reactivity of **1** with CS_2_. The addition of CS_2_ (0.6–1 equiv) to **1** in diethyl ether solution for 24 h at room temperature afforded a new ferrous species, characterized by ^1^H, ^19^F NMR, and MB spectroscopies (δ, |*ΔE*_*Q*_| (^mm^/_s_) = 0.63, *0.33*) (Figure S14). The considerably smaller quadrupole splitting in the MB spectrum of the reaction product suggested a three-coordinate species as opposed to the anticipated four-coordinate [Fe­(κ^2^-CS_2_OH)] bicarbonato analogue. To our surprise, the product was identified as the terminal hydrosulfido complex (^Em^L)­Fe­(SH) (**4**) via SCXRD (Figure 2c), featuring an Fe–S bond distance of 2.2396(11) Å. Potential intermediates en route to the formation of **4** were not observed by NMR spectroscopy. We presume the generation of a bicarbonato analogue as an intermediate akin to that proposed in the analogous imido-sulfido exchange reaction^54^ and the release of COS or CO_2_ gas owing to the entropic driving force to release gas as well as provide thermodynamic stability to the Fe–S bonding (vide infra).^55^

### 2.2 Reversible Reactivity of (^Em^L)­Fe­(OH) with CO_2_

Following the reactivity studies that establish the nucleophilic character of **1** above, we sought to investigate the reactivity of **1** toward CO_2_ (Scheme 3). The introduction of 1 atm of CO_2_ gas into the headspace of a C_6_D_6_ solution of **1** in a sealed NMR tube rapidly generates a new paramagnetic species, as determined by ^1^H and ^19^F NMR spectroscopies. Interestingly, the starting hydroxo **1** was still observed and could be quantitatively regenerated upon application of a vacuum. The partial consumption and regeneration of **1** under varying CO_2_ pressures implies the equilibrium of **1** under a CO_2_ atmosphere. An equilibrium constant for this process (*K*_eq_ = [product] [**1**]^−1^ *p*_CO2_^–1^) was determined to be 27 atm^–1^ in C_6_D_6_ at room temperature using ^19^F NMR spectroscopy (Figures 3a and S62). The free energy change during the equilibrium of **1** with CO_2_ was calculated as Δ*G*° = −2.0 kcal/mol from *K*_eq_.

**Figure 3:**
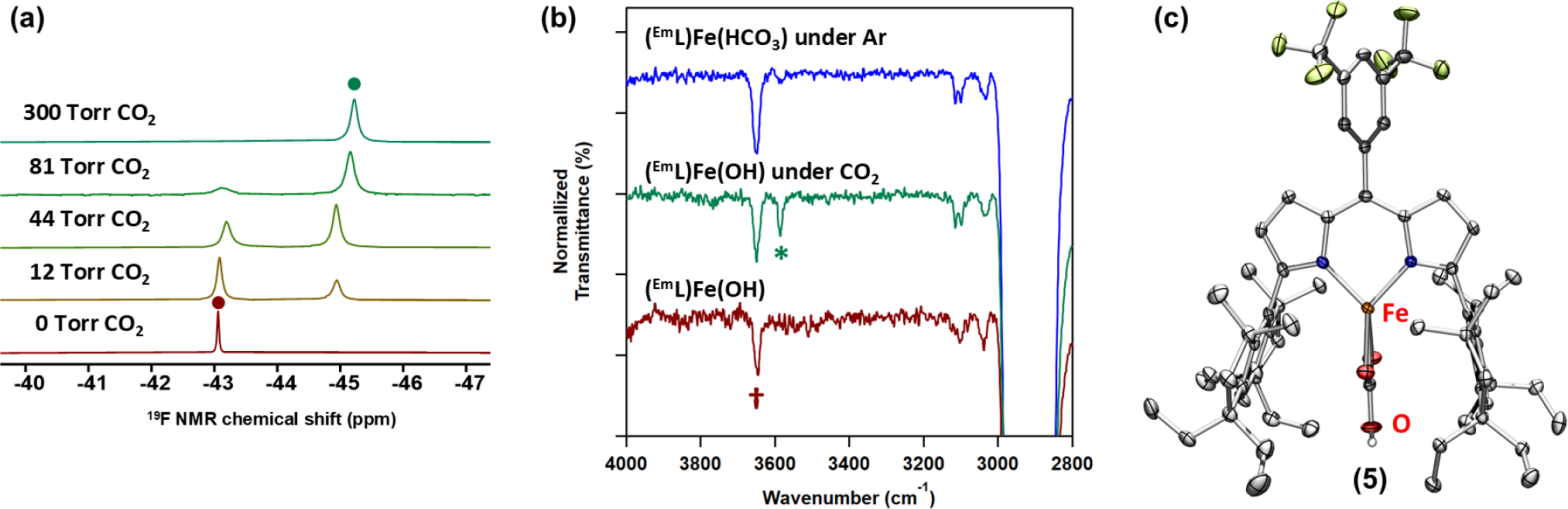
^19^F NMR spectra of (^Em^L)­Fe­(OH) (**1**) under varying pressures of CO_2_ gas from 0 Torr (*red*, bottom) to 300 Torr (*green*, top) in C_6_D_6_ solution; red and green dots represent **1** and **5**, respectively (a). Stacked IR spectra of (^Em^L)­Fe­(OH) (*red*, bottom), stored under a CO_2_ atmosphere (1.05 atm) for 24 h at room temperature (*green*), and followed by incubation under an argon atmosphere in 20 min at room temperature (*blue*, top) (b). ν­(OH) values of **1** and **5** are denoted with † and *, respectively. Solid-state structure of **5** obtained by the single-crystal conversion of **1** under a CO_2_ atmosphere at room temperature (c).

**Scheme 3:**
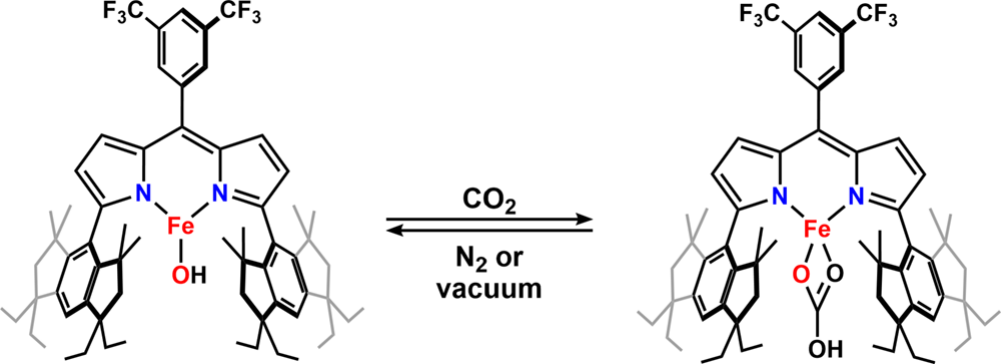
Reaction of (^Em^L)­Fe­(OH) (**1**) with CO_2_ to Reversibly Generate (^Em^L)­Fe­(κ^2^-O,O-HCO_3_) (**5**) in either Solution or Solid State

To identify the equilibrium product, a benzene solution of **1** under 1.5 atm of CO_2_ gas at room temperature was trapped by freeze-quenching the solution in liquid nitrogen and analyzed by MB (δ, |*Δ* *E*_*Q*_| (^mm^/_s_) = 0.96, *1.29*) (Figure S63). The comparable MB parameters of (^Em^L)­Fe­(κ^2^-O,O-OAc) (δ, |*Δ* *E*_*Q*_| (^mm^/_s_) = 0.93, *1.07*) and (^Em^L)­Fe­(κ^2^-O,O-O_2_CH) (δ, |*Δ* *E*_*Q*_| (^mm^/_s_) = 0.95, *1.38*) suggest the generation of a bidentate bicarbonato complex from **1** under a CO_2_ atmosphere (vide infra). Due to the fast equilibrium of the reaction between **1** and CO_2_ in the solution state, further spectroscopic analysis of the reaction of **1** with CO_2_ was challenging. Instead, examining the reactivity with more robust CO_2_ analogues (e.g., AdNCO) enabled us to investigate the equilibrium of **1** under CO_2_, by generating stable products, as well as explore the nucleophilic reactivity of **1**.

We proposed that the addition of CO_2_ to **1** in solution generates a ferrous bicarbonato adduct based on NMR and MB spectroscopies. However, detailed characterization of the presumed bicarbonato adduct (^Em^L)­Fe­(κ^2^-O,O-HCO_3_) was impeded by the rapid equilibrium of **1** under varying CO_2_ pressures. To unambiguously characterize the reaction product between **1** and CO_2_, we turned to solid-state conversion, anticipating slower kinetics due to gas diffusion through a solid crystal matrix. We monitored the solid-state transformation of **1** under CO_2_ by using IR spectroscopy. The IR spectrum was collected using an ATR-IR spectrometer under an argon atmosphere upon the exposure of crystals of **1** to 1.05 atm of CO_2_ gas for 24 h at room temperature in a Schlenk flask. The IR spectrum revealed a new red-shifted O–H stretching (ν­(OH) = 3586 cm^–1^), distinct from the ν­(OH) of **1** at 3650 cm^–1^ (Figure 3b). Notably, the new O–H vibration disappeared upon the continuous IR spectrum measurement of the crystal under an argon atmosphere at room temperatures within 20 min (Figure 3b), which is like the fast equilibrium observed in solution. Therefore, we attribute the new vibrational mode to the O–H stretch mode of a new species formed in the equilibrium of **1** under a CO_2_ atmosphere. We were unable to assign the asymmetric C=O stretch mode in the IR spectrum as this mode is red-shifted below 1600 cm^–1^ for bidentate bicarbonate ligands (vide infra), which overlaps with a dipyrrin vibrational mode (Figure S65).^56,57^

To corroborate the solid-state IR data, we sought structural validation for the formation of the ferrous bicarbonato complex. A well-diffracting single crystal of **1** was pressurized under 1.05 atm of CO_2_ in a Schlenk flask at room temperature for 24 h, then mounted on an X-ray diffractometer set at 100 K. The solid-state structure unambiguously establishes the generation of ferrous bicarbonato (^Em^L)­Fe­(κ^2^-O,O-HCO_3_) (**5**) (Figure 3c) from the equilibrium of **1** under CO_2_. The proton location on bicarbonato was determined for the terminal O atom via electron density from the Fourier difference map (Figure S87).^58^ As observed in hydroxide complex **1**, the bicarbonate motif in **5** is also sterically protected by the ligand scaffold, and no hydrogen-bonding network is present. Remarkably, warming the crystal to 300 K under a N_2_ stream resulted in the complete liberation of CO_2_ from bicarbonato **5** and regeneration of **1**. The solid-state conversion from (^Em^L)­Fe­(κ^2^-O,O-HCO_3_) (**5**) to (^Em^L)­Fe­(OH) (**1**) was monitored by the occupancy change of two moieties in the crystal lattice under dynamic N_2_ purge, completed in 7 days (Figure S74). The CO_2_ liberated from **5** freely diffused out of the crystal lattice, and we were unable to locate the CO_2_ in the Fourier difference map.^58^ Additionally, rotation of the ethyl group from the (^Em^L) ligand substituents fill the voids generated by CO_2_ loss, akin to previous observations during the in-crystallo N_2_ liberation of (^Em^L)­Fe­(N_3_) at 100 K^38^ and O_2_ binding to (^Em^L)­Cu­(N_2_) at room temperature.^59^

To support the experimental findings, densify functional theory (DFT) calculations were performed using the B3LYP functional^60,61^ and def2-tzvp (Fe, N, O)^62^ and def2-svp (F, C, H)^63^ basis sets. Geometry optimization revealed that the terminal OH, (^Em^L)­Fe­(κ^2^-O,O-HCO_3_) (**5**), is energetically favored over coordination with bicarbonato OH (i.e., (^Em^L)­Fe­(κ^2^-O,OH-HCO_3_)) by 6.25 kcal/mol (Figure S107, Table S8). The calculated Gibbs free energy for the generation of **5** from (^Em^L)­Fe­(OH) (**1**) and CO_2_ was essentially thermoneutral (Δ*G*°_calc_ = +0.15 kcal/mol at 298.15 K), aligning closely with the experimentally determined value from NMR spectroscopy (Table S8). Moreover, frequency calculations from the optimized structure using the same level of theory predicted the O–H vibration of (^Em^L)­Fe­(κ^2^-O,O-HCO_3_) and (^Em^L)­Fe­(κ^2^-O,OH-HCO_3_) as 3578 and 3543 cm^–1^, respectively, consistent with the experimentally observed value of (^Em^L)­Fe­(κ^2^-O,O-HCO_3_) (3586 cm^–1^) in solid-state conversion. In sum, the experimental and computational results demonstrate reversible CO_2_ capture and release by utilizing the nucleophilicity of hydroxo **1** in both solid and solution states.

### 2.3 Ligand Identity on Reversible CO_2_ Binding on the (^Em^L)­Fe^II^ Scaffold

To assess whether the CO_2_ uptake by **1** is unique, we sought to alter the Fe–X ligation to probe how the electronegativity of X impacts the CO_2_ binding. We previously reported the synthesis of a series of three-coordinate ferrous dipyrrin complexes of the type (^Em^L)­Fe­(X), where X is CH_3_, NH_2_, OH, SH, and Cl. To expand the ligand series, we prepared three-coordinate iron complexes bearing a range of ligand electronegativities (e.g., X: H→F) as well as electron-rich organometallic ligands (e.g., X: CH_3_, C_2_H_5_). To generate (^Em^L)­Fe^II^(F), (^Em^L)­Fe­(I) was treated with a mixture of CsF and TlOTf in a THF solution (Scheme 4) which generated a new ferrous species, as characterized by ^1^H, ^19^F NMR, and MB spectroscopies (δ, |*Δ* *E*_*Q*_| (^mm^/_s_) = 0.82, *1.03*) (Figure S17). The product was identified as the three-coordinate fluoride (^Em^L)­Fe­(F) (**6**) as verified by SCXRD (Figure S88).

**Scheme 4:**
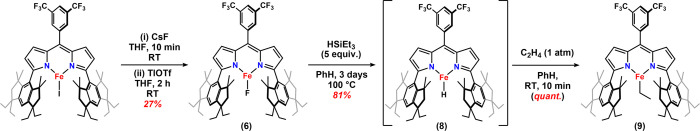
Syntheses of (^Em^L)­Fe­(F) (**6**), (^Em^L)­Fe­(H) (**8**), and (^Em^L)­Fe­(C_2_H_5_) (**9**)

In an attempt to generate a three-coordinate ferrous hydride, we utilized the nucleophilicity of **1** with the Si electrophile as observed in the generation of (^Em^L)­Fe­(N_3_) from **1** and N_3_SiMe_3_.^38^ Heating a solution of **1** with HSiEt_3_ ^64^ in benzene at 80 °C for 1 day does not generate the intended hydride but a terminal siloxide (^Em^L)­Fe­(OSiEt_3_) (**7**), as determined by NMR and MB spectroscopies (δ, |*Δ* *E*_*Q*_| (^mm^/_s_) = 0.73, *0.52*) (Figure S24) and SCXRD (Figure 2d). The narrow quadrupole splitting MB parameters are consistent with those of other three-coordinate dipyrrinato complexes generated in this study. When the reaction was conducted in a sealed J-Young NMR tube, the H_2_ generation was observed by ^1^H NMR spectroscopy (Figure S22). Although no intermediates were observed, we propose an initial formation of (^Em^L)­Fe­(H) and HOSiEt_3_, followed by the immediate deprotonation of HOSiEt_3_ by in-situ-generated (^Em^L)­Fe­(H) (**8**). To prevent the generation of the HOSiEt_3_ byproduct, the flouride adduct (^Em^L)­Fe­(F) (**6**) was heated with excess HSiEt_3_ (5 equiv) at 100 °C for 3 days (Scheme 4), which yielded a new paramagnetic species along with a quantitative amount of FSiEt_3_, as monitored by ^1^H and ^19^F NMR spectroscopies. While growing single crystals of the putative hydride **8** has remained elusive, MB analysis (δ, |*Δ* *E*_*Q*_| (^mm^/_s_) = 0.47, *1.05*; Figure S27) of the material generated in situ is consistent with a three-coordinate ferrous adduct. Furthermore, the quantitative generation of the FSiEt_3_ byproduct aligns with a previous report on the analogous synthesis of a three-coordinate hydride from a fluoride starting material.^64^ The addition of ethylene gas (1 atm)^64–67^ with in-situ-generated (^Em^L)­Fe­(H) (**8**) in benzene solution (Scheme 4) quantitatively generates the ethyl complex (^Em^L)­Fe­(C_2_H_5_) (**9**), as verified by NMR and MB spectroscopies (δ, |*Δ* *E*_*Q*_| (^mm^/_s_) = 0.43, *1.04*) (Figure S30) and in the solid state by SCXRD (Figure S91).

With the series of three-coordinate ferrous compounds in hand, we systematically explored their reactivity toward CO_2_, examining their nucleophilicity as a function of ligand electronegativity. Both (^Em^L)­Fe­(H) and (^Em^L)­Fe­(NH_2_)^38^ rapidly reacted with 1 atm of CO_2_ in C_6_D_6_ solution at room temperature to quantitatively produce new ferrous compounds as monitored by ^19^F and ^1^H NMR spectroscopies. The reaction products were identified as a formato (^Em^L)­Fe­(κ^2^-O,OiO_2_CH) (**10**) by MB (δ, |*Δ* *E*_*Q*_| (^mm^/_s_) = 0.95, *1.38*) (Figure S33) and SCXRD (Figure 4a) and a carbamato (^Em^L)­Fe­(κ^2^-O,O-O_2_CNH_2_) (**11**) (Figure 4b).^38^ In contrast, the two least basic ligands, hydrosulfide and fluoride, (^Em^L)­Fe­(SH) (**4**) and (^Em^L)­Fe­(F) (**6**), are stable under a CO_2_ atmosphere even at elevated temperatures. Notably, (^Em^L)­Fe­(CH_3_) reacts with CO_2_ only at elevated temperature, where the product was identified as a ferrous acetato (^Em^L)­Fe­(κ^2^-O,O-O_2_CCH_3_) (**12**) characterized by MB (δ, |*Δ* *E*_*Q*_| (^mm^/_s_) = 0.93, *1.07*) and SCXRD (Figure 4c). Interestingly, (^Em^L)­Fe­(C_2_H_5_) (**9**) yields two new ferrous species under a CO_2_ atmosphere in C_6_D_6_ solution at 100 °C, as monitored by ^19^F and ^1^H NMR spectroscopies. The new species are identified as a ferrous propionate (^Em^L)­Fe­(κ^2^-O,O-O_2_CCH_2_CH_3_) (**13**) (Figure 4d) and a ferrous formate (^Em^L)­Fe­(κ^2^-O,O-O_2_CH) (**10**), identified by comparison with the NMR spectra of independently synthesized complexes (Figure S70). We propose that β-hydride elimination from (^Em^L)­Fe­(C_2_H_5_) at elevated temperature generates (^Em^L)­Fe­(H) in situ, subsequently yielding the ferrous formate upon CO_2_ insertion (vide infra) (Scheme 5) (Figure S96). However, we cannot generate (^Em^L)­Fe­(H) in the absence of CO_2_. This observation parallels previously reported ferrous alkyl-olefin exchange in a three-coordinate iron β-dikeminate scaffold.^66^

**Figure 4:**
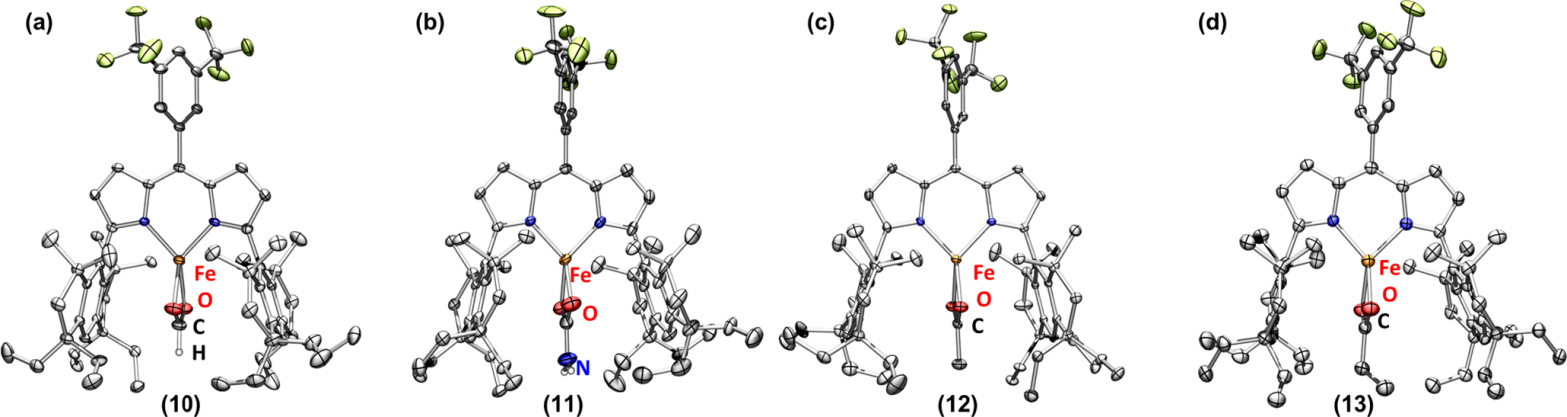
Solid-state structures of (a) **10**, (b) **11**, (c) **12**, and (d) **13** at 100 K with thermal ellipsoids at the 50% probability level (hydrogen atoms except for those located on N and formate and the solvent are omitted for clarity; Fe, orange; H, white; C, gray; N, blue; O, red; F, yellow-green).

**Scheme 5:**
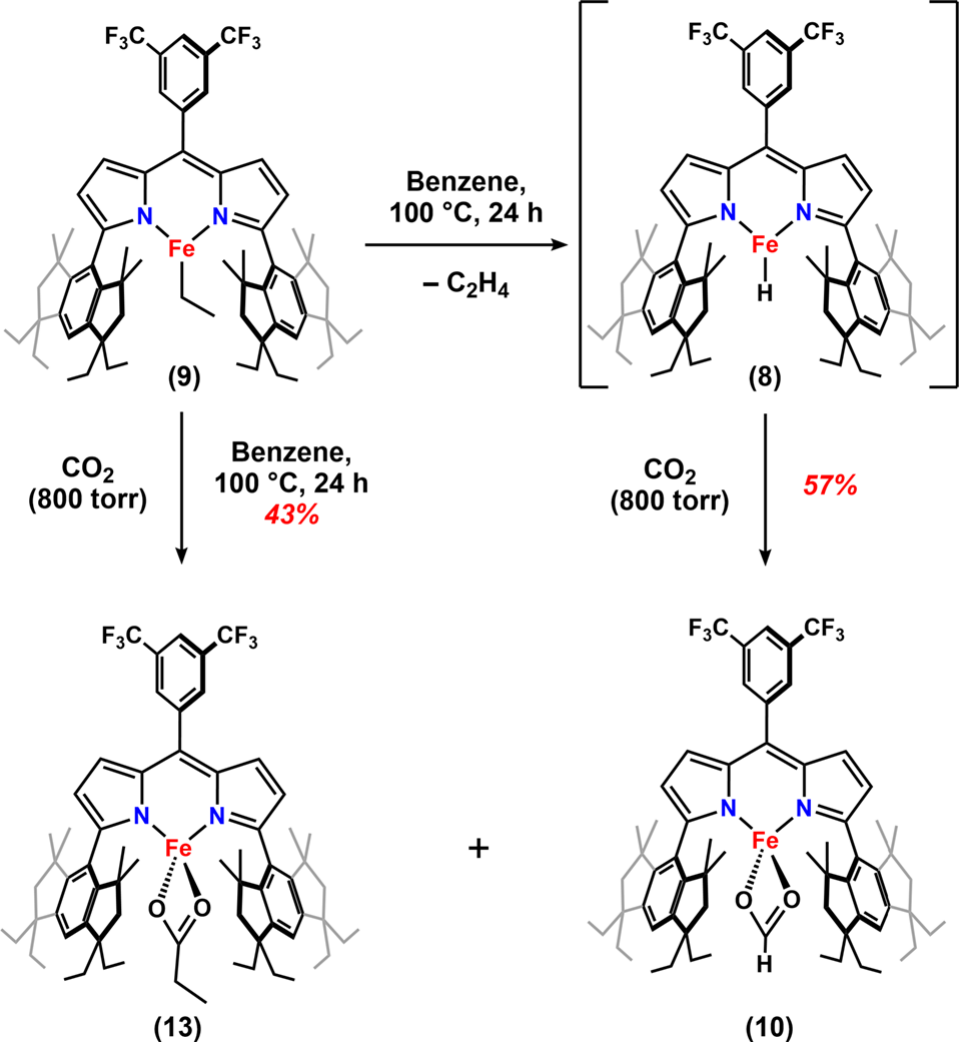
Reaction of (^Em^L)­Fe­(C_2_H_5_) (**9**) with CO_2_

To elucidate the factors underpinning the observed reversible CO_2_ binding of (^Em^L)­Fe­(OH) (**1**) to form (^Em^L)­Fe­(κ^2^-O,O-HCO_3_) (**5**), we examined whether CO_2_ could be released from ferrous formato (**10**), carbamato (**11**), or acetato (**12**) complexes. These complexes exhibited thermal stability up to 80 °C in either solid powder form under a vacuum or in solution. Given the thermoneutral equilibrium between **1** and **5**, we hypothesized significant thermodynamic changes within the series of CO_2_ capture reactions, leading to the high energy barrier for CO_2_ release from **10–12**. However, experimental determination of the thermodynamic parameters for generating **10**–**13** is challenging due to their largely exergonic nature. Thus, by systematically varying the anionic ligand identity X in (^Em^L)­FeX, we demonstrate the capacity to tune the nucleophilicity of the ferrous dipyrrin complexes toward CO_2_, highlighting the pivotal role that ligand electronegativity and basicity play in modulating iron–ligand reactivity.

### 2.4 Probing the Effect of Metal Valency on the Nucleophilic/Electrophilic Behavior of the Terminal (^Em^L)­Fe­(OH)

The relative electronegativity between the metal centers and coordinated ligands can be represented by their redox-dependent group transfer reactivity.^3,31,33–35,68^ The foregoing observations reveal the nucleophilic character of the hydroxide ligand in **1**, which can be contrasted with the electrophilic character observed for higher-valent Fe­(OH) complexes.^1,5,19–30^ To explore whether different redox states of **1** are accessible, cyclic voltammetry (CV) of **1** was conducted in the noncoordinating solvent 1,2-difluorobenzene (Figure 5a). CV reveals a reversible one-electron reduction at −1.83 V (vs [Cp_2_Fe]^+/0^) and an irreversible oxidation at +0.72 V. The chemical reduction of **1** using one equivalent of KC_8_ in thawing THF solution yielded a new paramagnetic species, as observed by ^1^H, ^19^F NMR, MB (δ, |*Δ* *E*_*Q*_| (^mm^/_s_) = 0.59, *0.68*) (Figure S45) and IR spectroscopies (ν­(OH) = 3637 cm^–1^) (Figure S43). The new paramagnetic species can be identified as [KC_222_]­[(^Em^L)­Fe­(OH)] (**14**) by SCXRD following the encapsulation of the potassium cation using [2.2.2]­cryptand (Figure 5b). IR spectroscopy showed the disappearance of characteristic dipyrrin stretching (ν­(dipyrrin, **1**) = 1538 cm^–1^) (Figure S44), suggesting a loss of electron delocalization over the dipyrrin ligand scaffold (Figure S108). Furthermore, structural evidence from the solid-state structure of **14** indicated the elongation of pyrrole–C_meso_ bonds (1.396(4), 1.402(4) Å to 1.424(10), 1.439(10) Å; Figure S101) and a very similar isomer shift for **14** (0.59 mm/s) compared to the hydroxo **1** (0.73 mm/s), supporting a dipyrrin-based, one-electron reduction to a formal (^Em^L^·^)^2–^/Fe^II^ configuration, rather than a metal-centered reduction (i.e., (^Em^L)­Fe^I^). Variable temperature susceptibility data measured via SQUID magnetometry revealed an *S* = ^3^/_2_ electronic ground state consistent with antiferromagnetic coupling between a ligand radical [(^Em^L^**•**^)^2–^, *S* = ^1^/_2_] and the high-spin Fe^II^ (*S* = 2) (Figure S47). DFT calculations further corroborate this antiferromagnetic coupling, yielding a broken symmetry solution (BS4,1) as the ground state, featuring a weak antiferromagnetic coupling (*J* = −50 cm^–1^) between the radical anion ligand and iron (Figure 5c).

**Figure 5:**
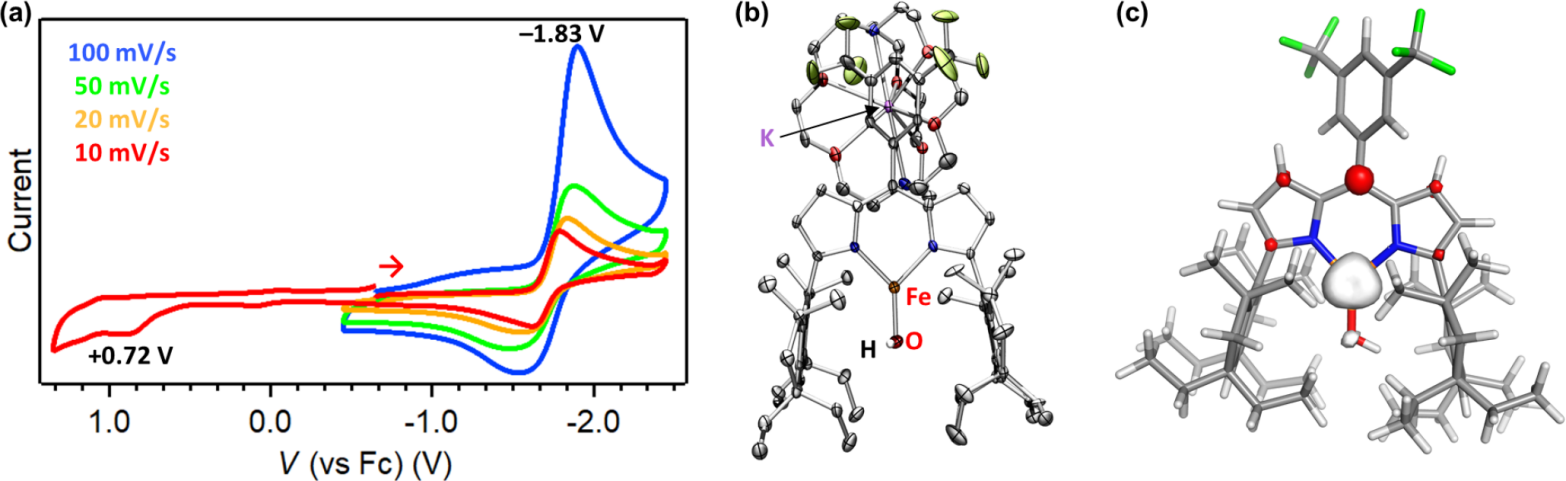
CV plot of **1** in 0.1 M [^*n*^Bu_4_N]­[PF_6_] electrolyte solution in 1,2-difluorobenzene (a); the solid-state structure of **14** at 100 K with thermal ellipsoids at the 50% probability level (hydrogen atoms except for those located on the hydroxide fragment, and solvent molecules are omitted for clarity; Fe, orange; C, gray; N, blue; O, red; F, yellow-green; K, purple) (b); and a spin-density plot (α–β) of the lowest energy electronic configuration (*S* = 3/2, BS­(4,1)) of **14** (isovalue = 0.015 *e* Å^–3^) (c).

Intriguingly, while the one-electron reduction of **1** became ligand-centered and did not affect the Fe^II^(OH), the introduction of gaseous CO_2_ into a C_6_D_6_ solution of [KC_222_]­[(^Em^L)­Fe­(OH)] (**14**) at room temperature resulted in the decomposition of **14** to multiple unidentified species, including (^Em^L)­H, consistent with demetalation. Considering the cathodic redox potential required for outer-sphere electron transfer to CO_2_ (−2.21 V vs SCE, −2.67 V vs Fc^+/0^ in DMF, and −1.90 V vs SCE in water),^69–72^ we exclude direct electron transfer to CO_2_ as a direct decomposition pathway. Instead, the existence of an acidic proton on the potential bicarbonate intermediate upon exposure to CO_2_ can be reduced to generate hydrogen akin to the competitive hydrogen evolution reaction during CO_2_ reduction in aqueous media.^72^ Thus, the ligand-centered reduction prevented further studies of the reactivity of Fe–OH.

To compare the ferrous and ferric hydroxo reactivity within the same ligand scaffold, we targeted the oxidation of (^Em^L)­Fe­(OH) (**1**). Given the highly anodic redox potential required for the first irreversible oxidation of (^Em^L)­Fe­(OH) in CV (Figure 5a), we attempted the oxidation of **1** employing inner-sphere oxidants. The addition of excess I_2_ to a solution of (^Em^L)­Fe­(OH) (**1**) generated a new paramagnetic product (Scheme 6). Subsequent MB (δ, |*Δ* *E*_*Q*_| (^mm^/_s_) = 0.36, *1.33*) and EPR analysis (Figure 6a,c) of the reaction product indicate that the product is consistent with a high-spin (*S* = ^5^/_2_) ferric species. While the solid-state structure of this species has remained elusive, ESI-MS is consistent with the new ferric species (Supporting Information) as four-coordinate ferric hydroxo (^Em^L)­FeI­(OH) (**15**). The generation of **15** was further confirmed by IR spectroscopy (Figure 6b), which showed an O–H stretching (ν­(OH)) of 3642 cm^–1^, matching with the value predicted by DFT (3631 cm^–1^). The combination of experimental and theoretical calculations confirmed the ferric state of **15**. A dimeric (hydr-)­oxo-bridged ferric complex formation cannot be ruled out from spectroscopic evidence, however, as we reported a diferric, oxo-bridged Fe_2_(μ-O)­(OH)_2_ species in a pacman-dipyrrin ligand scaffold.^73^ We unambiguously identify **15** as monomeric ferric hydroxo from the quantitative (per iron) ^•^OH transfer reactivity of **15** (vide infra) and quantitative generation of (^Em^L)­Fe­(I) following ^•^OH transfer.

**Scheme 6:**
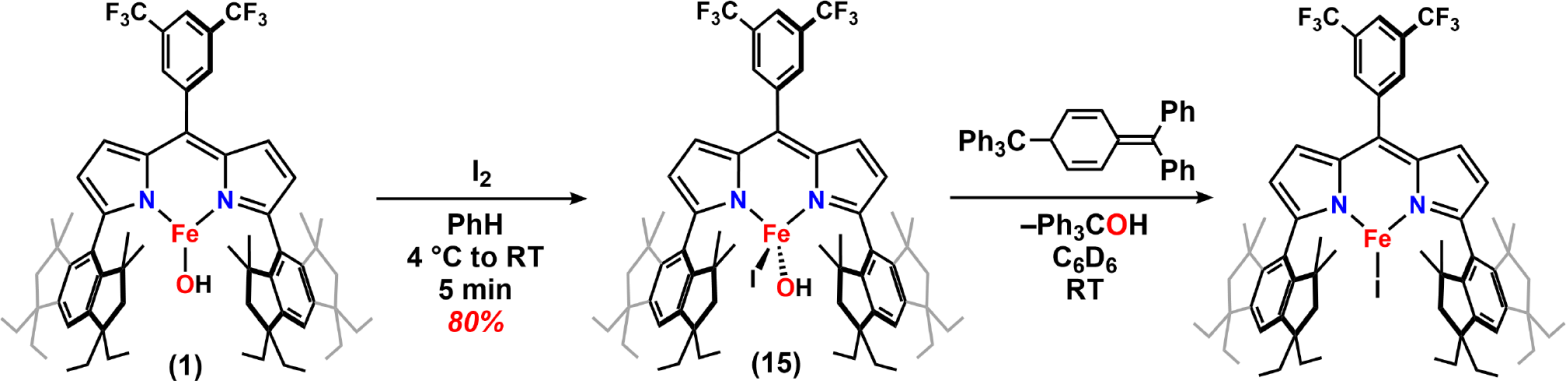
Synthesis of (^Em^L)­Fe­(OH)­(I) (**15**) and the Hydroxyl Radical Rebound Reactivity

**Figure 6:**
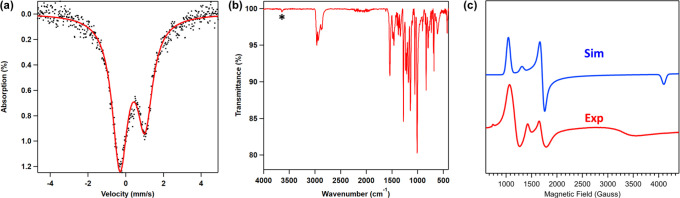
Zero-field ^57^Fe Mössbauer spectrum at 90 K of **15** (a); IR spectrum of **15** (ν­(OH) is denoted with an *) (b); EPR spectra of **15** collected in a frozen toluene matrix at 77 K (red) and simulated with *S* = 5/2 and |*E*/*D*| = 0.062 (blue) (c).

Remarkably, the ferric hydroxo (^Em^L)­FeI­(OH) (**15**) displayed no nucleophilic reactivity toward CO_2_ but did react with persistent organic radicals, consistent with the electrophilic character of **15**. Rather, the addition of Gomberg’s dimer in C_6_D_6_ solution with **15** generated (^Em^L)­Fe­(I) and Ph_3_C–OH (91 ± 1%) at room temperature (Scheme 6, Figure S66). The generation of (^Em^L)­Fe­(I) was confirmed by comparing the NMR spectra with an authentic spectrum of (^Em^L)­Fe­(I) (**16**), and the yield of Ph_3_COH was determined by ^1^H NMR spectroscopy using (Me_3_Si)_2_O as an internal standard. Given that **1** does not react with Gomberg’s dimer under similar reaction conditions, the observed carboradical rebound with the hydroxo radical of **15** underscores the less polarized and electrophilic nature of the Fe–OH moiety in the ferric state in **15** relative to its ferrous counterpart in **1**. However, the ferric hydroxo **15** was notably inert toward weak C–H sources (i.e., 1.4-cyclohexadiene), suggesting that **15** lacks sufficient oxidizing power to activate C–H bonds.^26^

Importantly, the observed reactivity of **15** aligns with previous report by other high-valent iron hydroxo moieties,^19–28^ emphasizing that the oxidation state alters the reactivity profile of iron hydroxide complexes. To probe the polarity change of Fe–O–H motifs, the basicity values of **1** and **15** were estimated by DFT calculations.^73,74^ The p*K*a values of conjugate acids, [Fe^II^(H_2_O)]^+^ (4.0) and [Fe^III^(H_2_O)­(I)]^+^ (−0.4) (Table S11), reveal that the hydroxo moiety in the ferrous state is more basic and polar than in the ferric analogue. Collectively, the differing reactivity observed between ferrous **1** and ferric **15** demonstrates that the terminal iron hydroxide moiety, previously known to be electrophilic, can engender polar nucleophilic reactivity when accessed in the understudied ferrous state.

## 3 Discussion

The three-coordinate complex (^Em^L)­Fe­(OH) (**1**) induces reactivity using both the electrophilic iron center and the nucleophilic hydroxo ligand. The low-coordinate environment is rare among reported iron hydroxo given the high basicity of the hydroxide ligand, commonly resulting in dimerization or oligomerization. Indeed, reducing the steric demands of the dipyrrin scaffold or having potassium cations in the reaction mixture (e.g., using KOH as a hydroxide source) led to rapid dimerization of the (^Em^L)­Fe­(OH) moiety, whereas coordinating solvents (e.g., THF) readily bind **1** to form four-coordinate species. Despite these constraints, we successfully synthesized **1** via the hydrolysis of (^Em^L)­Fe­(CH_3_) with a stoichiometric equivalent of H_2_O delivered in a saturated benzene solution.

Quite remarkably, the terminal hydroxo ligand can exhibit both nucleophilic and electrophilic behavior. The ferrous hydroxo adduct demonstrates rich nucleophilic reactivity, as evidenced by the addition chemistry to unsaturated substrates (e.g., nitrile, isocyanate, CO_2_), whereas the ferric hydroxo exhibits more electrophilic reactivity reminiscent of our previously reported iron nitrenoids and nitride.^3,31–35^ The observation of hydroxyl radical transfer from (^Em^L)­FeI­(OH) (**15**) to a persistent carboradical is consistent from recent reports on high-valent iron hydroxo complexes and also dipyrrin ferric *tert*-butoxide^75^ owing to the identical high-spin electronic configuration during the reaction. The capacity of **15** to furnish new C–OH bonds from radical recombination reactivity is analogous to a variety of C–H bond amination reactions.

### 3.1 Computational Analysis of the Reaction under CO_2_

Given the reversible CO_2_ binding exhibited by the hydroxo adduct **1**, we generated a family of complexes of the type (^Em^L)­FeX to understand how the nature of X could facilitate the reactivity with CO_2_. We observed that the reactions between (^Em^L)­Fe^II^(X) and CO_2_ exhibit different thermodynamics and kinetics: reversible (X = OH), irreversible (X = H, NH_2_), or irreversible but requiring elevated temperature (X = CH_3_, CH_2_CH_3_). The DFT calculations using the B3LYP functional with def2-tzvp (Fe, N, and O) and def2-svp (for other atoms) basis sets predicted well the experimental free energy changes (Δ*G*°) for the reaction between (^Em^L)­Fe­(OH) and CO_2_ (vide supra). Thus, we performed DFT calculations at the same level of theory to examine the kinetic details underlying the reversible behavior of high-spin (^Em^L)­Fe­(OH) toward CO_2_, including hypothetical lower spin states (i.e., *S* = 1 and 0). To reduce the computational complexity and cost, we truncated the (^Em^L) scaffold by replacing the three aryl groups on the dipyrrin scaffold with methyl groups (^Me^L), preserving the primary coordination sphere. Given that the electronics^76^ and sterics^77–79^ of the system can significantly affect either kinetics^77^ or thermodynamics^78,79^ of reversible CO_2_ capture processes, we examined the truncated model complex in detail before investigating the reversible CO_2_ binding reaction of interest. The preserved primary coordination structure and thermodynamics of CO_2_ capture using the truncated ligand model suggested that minimal energetic perturbations resulted from the ligand simplification and thus enabled us to examine the computational kinetic profiles. Remarkably, while the steric bulk of ^Em^L was critical to experimentally demonstrating the reactivity profile of three-coordinate ferrous hydroxo, the preserved primary coordination sphere and energetic profile of the reaction between (^Em^L)­Fe­(X) and CO_2_ examined using ligand truncation (Tables S6 and S7) reveal that the observed reactivity of three-coordinate ferrous hydroxo arises from electronic factors preserved in the simplified ligand model.

The DFT-calculated transition state for a reaction between (^Me^L)­Fe^II^(OH) and CO_2_ was obtained with only one imaginary frequency, with forward (Δ*G*_capture_^‡^) and reverse (Δ*G*_release_^‡^) energy barriers of 12.6 and 11.7 kcal/mol, respectively. The calculated low-energy barriers align with the observed fast equilibrium observed in solution under ambient conditions. The transition state exhibits partial Fe–OH cleavage (*d*_Fe–OH_ = 1.94 Å) along with partial OH–CO_2_ formation (*d*_FeO–CO2_ = 1.90 Å) (Figure 7c). Notably, the oxygen of CO_2_ associates to the iron center (*d*_Fe–O_ = 2.28 Å), which is comparable to the Fe–O distance (2.25 Å) of the DFT optimized structure of (^Me^L)­Fe­(OH)­(thf) (Table S6). The Fe–O interaction highlights the role of the electrophilic, low-coordinate iron center, complementing the nucleophilicity of the hydroxide motif. The [2 + 2]-like transition state is akin to the reactivity of previously reported late transition metal complexes with borane, silane, or ethylene.^80–84^

**Figure 7:**
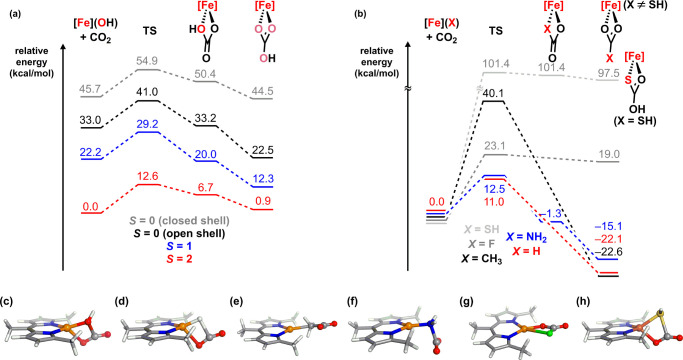
DFT-calculated reaction pathway for the reaction of ferrous hydroxo, (^Me^L)­Fe­(OH), with CO_2_ by function of spin state (a) and a transition-state structure for the quintet surface (c); DFT-calculated reaction pathway for the reaction of (^Me^L)­Fe­(X) and CO_2_ (X = H, CH_3_, NH_2_, F, SH) (b) and transition-state structures for X = H (d), CH_3_ (e), NH_2_ (f), F (g), and SH (h) (B3LYP functional, def2-tzvp (for Fe, N, and O) and def2-svp (for other atoms) basis sets using Orca5).

To understand the role of high-spin electronic configuration in the reaction between (^Em^L)­Fe^II^(OH) and CO_2_, we conducted free energy calculations on the reaction coordinate for triplet, open-shell singlet, and closed-shell singlet surfaces (Figure 7a). Given that terminal Fe^II^–OH complexes favor high-spin electronic configurations owing to the electronegative hydroxide ligand,^8,11,12,14–18^ the computational result on the hypothetical spin states cannot be compared to experimental data. The high-spin state has the highest calculated kinetic barrier (Δ*G*_capture_^‡^), but the calculated free energy changes (Δ*G* ^0^) vary significantly as a function of the spin state (*S* = 1, −9.9 kcal/mol; *S* = 0 (open-shell), −10.5 kcal/mol; *S* = 0 (closed-shell), −1.2 kcal/mol). The varying thermodynamics on the hypothetical lower spin surfaces suggest the electrophilic ferrous center also influences the Fe–O bonding energy.

As we observed that the thermodynamic and kinetic profiles for the reactivity of (^Em^L)­Fe­(X) complexes toward CO_2_ vary as a function of the identity of ligand X, we computationally explored the reaction of (^Me^L)­Fe­(X) with CO_2_ to investigate the effect of ligand identity further. The calculated free energies (Δ*G* ^0^) were endergonic (Δ*G* ^0^ > 0) for the ligands stable under CO_2_ (X: F, SH) but exergonic (Δ*G* ^0^ < 0) for ligands that showed reactivity with CO_2_ (X: H, CH_3_, and NH_2_). The thermodynamic trend is in line with the basicity of the ligand (Table S11) owing to dative metal–ligand bonding in the high-spin ferrous state. Transition-state analyses revealed comparable forward kinetic barriers (Δ*G*_capture_^‡^ = 11.0, 12.5 kcal/mol for X = H, NH_2_, respectively) to X = OH (Δ*G*_capture_^‡^ = 12.6 kcal/mol), consistent with the immediate reaction observed at room temperature. However, the kinetic barrier is significantly higher for X = CH_3_ (Δ*G*_capture_^‡^ = 40.1 kcal/mol), which parallels with the observation of proceeding only at the elevated temperature (100 °C, vide supra). Additionally, the kinetic barrier for X = C_2_H_5_ (35.1 kcal/mol) (Figure S109) is lower than that for X = CH_3_, consistent with the elongated Fe–C distance of (^Em^L)­Fe­(C_2_H_5_) (Fe–C (Å): 2.21, 2.16 Å for X = CH_3_, C_2_H_5_, respectively).

The thermodynamic stability of CO_2_ capture by Fe­(X) (X = H, NH_2_, and CH_3_) results in high free energy barriers for CO_2_ extrusion (Δ*G*_release_^‡^ = Δ*G*_capture_^‡^ + Δ*G* ^0^ = 33.1, 62.8, and 27.6 kcal/mol for X = H, CH_3_, and NH_2_, respectively), which are substantially larger than that for the reversible binding exhibited by the hydroxide (Δ*G*_release_^‡^ = 11.7 kcal/mol). Additionally, we calculate a large exergonic (Δ*G* ^0^ = – 97.5 kcal/mol) and barrier-free CO_2_ release (Δ*G*_release_^‡^ = 3.9 kcal/mol) from (^Em^L)­Fe­(S­(O)­COH) to (^Em^L)­Fe­(SH) as well as an exergonic (−15.2 kcal/mol) (^Me^L)­Fe­(SH) generation from (^Me^L)­Fe­(OH) with a half equivalent of CS_2_, explaining the large thermodynamic driving force for generating **4** from the reaction of **1** with CS_2_. The DFT-calculated thermodynamics and kinetics corroborate the experimental observations, revealing the thermodynamic manipulation of CO_2_ capture/release in three-coordinate ferrous complexes.

As the reaction of (^Em^L)­Fe­(X) with CO_2_ diverges by ligand identity on the iron center, the mode of activating the electrophilic CO_2_ substrate by iron complexes varies as a function of the ligands. Thus, we performed detailed transition-state comparisons to further elucidate ligand-dependent CO_2_ capturing pathways for this series of three-coordinate ferrous ions. All ligands except −CH_3_ afford a perpendicular Fe–X–CO_2_ transition-state structure (Figure 7d,f–h); (^Me^L)­Fe­(CH_3_) exhibited a distinctly more linear angle (*∠*(Fe–C–C) = 137°) due to the absence of π*_Fe–CH3_ electrons interacting with the electrophilic carbon center of CO_2_. Essentially, electrons in the Fe–CH_3_ bond participate in the nucleophilic CO_2_ attack in the reaction between (^Em^L)­Fe­(CH_3_) and CO_2_, leading to an Fe–C bond elongation by 0.18 Å and a higher energy barrier at the transition state. While we observed the involvement of the electrophilic iron center at the transition states calculated for X = H, OH, F, SH, we calculate exclusive nucleophilic attack of the X-ligand without Fe–CO_2_ interactions (Fe–O > 3.3 Å) to occur when X = CH_3_ and NH_2_. In sum, the transition-state analyses reveal the role of a low-coordinate metal center leading to a polarizing electrophilic carbon center during the activation of CO_2_ by **1**.

Mechanistically, the reactions of (^Em^L)­Fe­(OH) with electrophiles involve the cooperation of the electrophilic ferrous ion and nucleophilic hydroxide, as revealed by the transition-state structure of the reaction with CO_2_. Although the ferrous center participation during nucleophilic reactions has been reported in migratory insertion reactions of ferrous alkyl or parent amido complexes with carbon monoxide, these reactions proceed via 18-electron intermediates following the CO coordination.^9,42–46^ In contrast, the electrophilic nature of **1** precludes coordination with a π-acidic CO ligand, resulting in a distinct reaction pathway from more traditional 18-electron species. Indeed, coordinatively unsaturated, low-coordinate, late transition metal nitrenoids can react with electrophiles,^81^ carbodiimide, isocyanate,^32,33^ or ketones^68^ via [2 + 2]-type cycloaddition, generating metallacycle products. Thus, the formation of **5** implies a reaction mechanism similar to that of the precedent low-coordinate late transition metal nitrenoids. Additionally, the hydrolysis of nitriles by **1** to generate **2** reveals the reactive nature of **1**, which has been considered as a challenging transformation.^48–53^

### 3.2 Application toward CO_2_ Capture and Conversion

Remarkably, (^Em^L)­Fe­(OH) (**1**) exhibits reversible CO_2_ capture to form the bicarbonato complex **5** at low temperatures both in solid and solution states. While organic molecular systems (e.g., phosphine, amine, NHC, FLPs) are known for reversible CO_2_ chemisroption, their CO_2_-captured adducts require substantial thermal energy to overcome the energy barrier in releasing CO_2_ (stable in solid state^76,85^ and in solution state in ambient conditions^78,79,86^ ). Similarly, transition metal complexes (i.e., metal hydrides,^57,87,88^ metal–ligand cooperativity of the Zn^II^(MeOH) complex,^89^ and Mg^II^(amine)^90^) have demonstrated reversible CO_2_ capture; remarkably, metal hydroxo complexes^91–93^ including **1** showed reversibility or lability under CO_2_ across various metal and supporting ligand scaffolds, while anionic ligands other than hydroxide do not exhibit reversible binding.^93^ The previous report on oxygen atom scrambling between CO_2_ and H_2_O catalyzed by zinc carbonic anhydrase^94^ suggested that O-atom scrambling occurs on zinc bicarbonato by the rotation of bicarbonato or internal proton transfer between oxygen atoms. While we cannot computationally find the transition state between two binding modes of bicarbonato (e.g., Fe­(κ^2^-O,O-HCO_3_) and Fe­(κ^2^-O,OH-HCO_3_)), previously reported nickel pincer complexes facilitate the low-barrier, rapid rearrangement among bicarbonato conformers.^77,92^ Indeed, the free energy difference between two conformers of bicarbonato (6.3 kcal/mol) is half that of carbamato (Fe­(κ^2^-O,N-NHC­(O)­OH), Fe­(κ^2^-O,O-O_2_CNH_2_)) (13.8 kcal/mol). The disparities between the calculated free energy of bicarbonato and carbamato among their conformers suggested that the low reorganization energy for the former also contributes to the unique thermoneutral and low-transition barrier of metal hydroxide under CO_2_ as well as the thermodynamics and kinetics of nucleophilic CO_2_ attack.

Carbon dioxide association to the electrophilic ferrous center of **1** plays a crucial role in overcoming the high energy barrier for CO_2_ hydration. Similarly, NiFe-CODHs leverage CO_2_ association to an electrophilic ferrous center (Fe_u_) for CO_2_ reduction to CO while generating Fe_u_^II^(OH) simultaneously.^36,37^ The conversion is reversible so that the nucleophilic attack by high-spin Fe_u_^II^(OH) converts the carbonyl of Ni^II^(CO) to carboxylate and subsequently release CO_2_. As (^Em^L)­Fe^II^ scaffolds can provide electrophilic and coordinatively unsaturated metal centers as well as nucleophilic Fe^II^(OH), (^Em^L)­Fe­(OH) can represent a compelling model system to understand an interconversion between CO_2_ and CO at NiFe-CODHs.^95,96^ Thus, future work will explore the reactivity of **1** with nickel carbonyl complexes to mechanistically investigate this bioinspired CO_2_ transformation. Moreover, given the current industrial standard (temperature swing using monoethanolamine requires high energy cost to overcome the energy barrier to release CO_2_), we envisioned that the low-coordinate metal hydroxo complexes can expand the repertoire of CO_2_ capture materials^97–99^ owing to the unique reversibility at low temperatures.

## 4 Conclusions

The foregoing results describe the reactivity of the three-coordinate terminal ferrous hydroxo (^Em^L)­Fe­(OH) toward electrophiles, including the reversible binding of CO_2_. The terminal ferrous hydroxo reacts with electrophiles, including activating the benzonitrile C≡N bond and generating hydrosulfido from CS_2_. Notably, the reaction with CO_2_ to generate bicarbonate is thermoneutral, with a low kinetic barrier, enabling reversibility at room temperature. The hydroxide ligand is unique in the series of (^Em^L)­Fe^II^(X) complexes with regard to the reversibility toward CO_2_ capture. However, the higher-valent ferric hydroxo (^Em^L)­Fe­(I)­(OH) is not nucleophilic toward CO_2_ but transfers electrophilic OH^•^ to the carboradical owing to the less polarized Fe–OH bonding. This difference in reactivity by metal valency highlights the importance of metal valency in the group transfer reactivity. Remarkably, the computational study reveals the involvement of the electrophilic ferrous center of (^Em^L)­Fe­(OH) in the reaction with electrophiles.

## Supplementary Material



## Notes

Materials and methods, syntheses, ^1^H NMR, ^13^C NMR, ^19^F NMR, EPR, zero-field ^57^Fe Mössbauer, and infrared spectra, solid-state molecular structures, crystallographic details and data, and theoretical calculation details (PDF)
