# Supplementary material for: Manipulating Terminal Iron-Hydroxide Nucleophilicity through Redox
Source: J Am Chem Soc. 2026 Jan 16;148(3):3723–35. doi: 10.1021/jacs.5c20166 (PMC12856904; doi:10.1021/jacs.5c20166)
Supplement: Supplementary file 1 [file ja5c20166_si_001.pdf]

Supplementary Information for

Manipulating Terminal Iron-Hydroxide Nucleophilicity through Redox

Jeewhan Oh[†], Kurtis M. Carsch[†], Shao-Liang Zheng[†], and Theodore A. Betley[†]*

[†]Department of Chemistry & Chemical Biology, Harvard University, 12 Oxford Street, Cambridge, MA 02138, United States

*Corresponding Author: betley@chemistry.harvard.edu

Table of Contents

Materials and Methods S7
General Considerations S7
Characterization and Physical Measurements S7

Synthesis S9
(^{Em}L)Fe(κ^2-N,O-NHC(O)Ph) (2) S9
Figure S1. ^{1}H NMR spectrum of **2** S9
Figure S2. ^{19}R NMR spectrum of **2** S10
Figure S3–S4. IR spectra of **2** S11
Figure S5. Zero-field ^{57}Fe Mössbauer spectrum of **2** S12
(^{Em}L)Fe(κ^2-O,O-O$_2$CN(H)Ad) (3) S13
Figure S6. ^{1}H NMR spectrum of **3** S13
Figure S7. ^{19}F NMR spectrum of **3** S14
Figure S8–S9. IR spectra of **3** S14
Figure S10. Zero-field ^{57}Fe Mössbauer spectrum of **3** S16
(^{Em}L)Fe(SH) (4) S17
Figure S11. ^{1}H NMR spectrum of **4** S17
Figure S12. ^{19}F NMR spectrum of **4** S18
Figure S13. IR spectrum o **4** S18
Figure S14. Zero-field ^{57}Fe Mössbauer spectrum of **4** S19
(^{Em}L)Fe(F) (6) S20
Figure S15. ^{1}H NMR spectrum of **6** S21
Figure S16. ^{19}F NMR spectrum of **6** S21
Figure S17. Zero-field ^{57}Fe Mössbauer spectrum of **6** S22
Figure S18. ^{1}H NMR spectrum of (^{Em}L)Fe(F)$_2$(Tl) S23
Figure S19. ^{1}H NMR spectrum of (^{Em}L)Fe(F)$_2$(Tl) S23
Figure S20. Zero-field ^{57}Fe Mössbauer spectrum of (^{Em}L)Fe(F)$_2$(Tl) S24
(^{Em}L)Fe(OSiEt$_3$) (7) S25
Figure S21–S22. ^{1}H NMR spectra of **7** S25
Figure S23. ^{19}F NMR spectrum of **7** S26
Figure S24. Zero-field ^{57}Fe Mössbauer spectrum of **7** S27
***In-situ* generation of (^{Em}L)Fe(H) (8)** S28
Figure S25. ^{1}H NMR spectrum of **8** S28
Figure S26. ^{19}F NMR spectrum of **8** S29
Figure S27. Zero-field ^{57}Fe Mössbauer spectrum of **8** S29
(^{Em}L)Fe(C$_2$H$_5$) (9) S30
Figure S28. ^{1}H NMR spectrum of **9** S30
Figure S29. ^{19}F NMR spectrum of **9** S31
Figure S30. Zero-field ^{57}Fe Mössbauer spectrum of **9** S31
(^{Em}L)Fe(*κ*^{2}O,O-O$_2$CH) (10) S32
Figure S31. ^{1}H NMR spectrum of **10** S33

Figure S32. ^{19}F NMR spectrum of **10** **S33**
Figure S33. Zero-field ^{57}Fe Mössbauer spectrum of **10** **S34**
(^{Em}L)Fe(κ^2O,O-O_2CCH_3) (12) **S35**
Figure S34. ^{1}H NMR spectrum of **12** **S35**
Figure S35. ^{19}F NMR spectrum of **12** **S36**
Figure S36. Zero-field ^{57}Fe Mössbauer spectrum of **13** **S36**
(^{Em}L)Fe(κ^2O,O-$O_2CCH_2CH_3$) (13) **S37**
Figure S37. ^{1}H NMR spectrum of **13** **S37**
Figure S38. ^{19}F NMR spectrum of **13** **S38**
Figure S39. Zero-field ^{57}Fe Mössbauer spectrum of **13** **S38**
[KC_{222}][(^{Em}L)Fe(OH)] (14) **S39**
Figure S40. ^{1}H NMR spectrum of **14** **S40**
Figure S41. ^{19}F NMR spectrum of **14** **S40**
Figure S42-S44. IR spectra of **14** **S41**
Figure S45. Zero-field ^{57}Fe Mössbauer spectrum of **14** **S44**
Figure S46. Zero-field ^{57}Fe Mössbauer spectrum of the crude mixture of oxidation of **14** **S45**
Figure S47–S48. SQUID magnetometry data of **14** **S46**
(^{Em}L)Fe(OH)(I) (15) **S48**
Figure S49–S50. ^{1}H NMR spectra of **15** **S48**
Figure S51. ^{19}F NMR spectrum of **15** **S49**
Figure S52–S53. IR spectra of **15** **S50**
Figure S54. EPR spectrm of **15** **S52**
Figure S55. Zero-field ^{57}Fe Mössbauer spectrum of **15** **S53**
(^{Em}L)Fe(I) (16) **S54**
Figure S56. ^{1}H NMR spectrum of **16** **S55**
Figure S57. ^{19}F NMR spectrum of **16** **S55**
Figure S58. Zero-field ^{57}Fe Mössbauer spectrum of **16** **S56**
Additional Characterization of (^{Em}L)Fe(OH) (1) **S57**
Figure S59. Zero-field ^{57}Fe Mössbauer spectra of **1** in frozen benzene and THF at 90 K **S57**

Characterization of (^{Em}L)Fe(κ^2O,O-HCO_3) (5) **S58**
Determination of equilibrium constant of 1 under CO_2 by NMR spectroscopy **S58**
Table S1. Pressure of CO_2 introduced and at equilibrium **S58**
Figure S60. Plot of [**5**]/[**1**] vs p_{CO2} **S59**
Figure S61. Stacked ^{1}H NMR spectra of **1** under varying CO_2 pressure **S59**
Figure S62. Stacked ^{19}F NMR spectra of **1** under varying CO_2 pressure **S60**
Characterization of frozen benzene solution of 5 by zero-field ^{57}Fe Mössbauer spectroscopy **S61**
Figure S63. Zero-field ^{57}Fe Mössbauer spectrum of **5** **S61**
Characterization of a solid state conversion of 1 under CO_2 by IR spectroscopy **S62**
Figure S64–S65. IR spectra of **5** **S62**

Reactivity study of 15 **S64**

Figure S66–S67. Stacked ^{1}H NMR spectra of the reaction between **15** and Gomberg's dimer **S64**

Investigation of oxidation of 1 **S65**
Figure S68. EPR spectra of oxidation of **1** with I_2, and $[FeCp_2][BArF_{24}]$ **S66**
Figure S69. Stacked ^{19}F NMR spectra of the titrating the mixture of **1** oxidation with $[FeCp_2][BArF_{24}]$ **S67**
Figure S70–S71. IR spectrum of $(^{Em}L)Fe(OH)_2$ **S68**

Reaction of $(^{Em}L)Fe(C_2H_5)$ with CO_2 **S70**
Figure S72. ^{19}F NMR spectrum of the reaction between **9** with CO_2 **S70**
Figure S73. Stacked ^{1}H NMR spectra of **10**, **12**, and the reaction between **13** with CO_2 **S71**

X-ray diffraction techniques **S72**
Single crystal conversion between 1 and 5 **S74**
Table S2. Unit cell parameters of conversion of **5** to **1** *in crystallo* **S75**
Table S3. Conversion ratio of **5** to **1** **S75**
Figure S74–S75. Conversion ratio of **5** to **1** by function of irradiation time *in crystallo* **S76**
Table S4. X-ray diffraction experimental details **S77**
Figure S76–S78. Solid-state structure of **2** **S81**
Figure S79–S80. Solid-state structure of **3** **S83**
Figure S81–S82. Solid-state structure of **4** **S85**
Figure S83–S87. Solid-state structure of **5** **S87**
Figure S88. Solid-state structure of **6** **S91**
Figure S89–S90 Solid-state structure of **7** **S92**
Figure S91. Solid-state structure of **9** **S94**
Figure S92–S93. Solid-state structure of **10** **S95**
Figure S94–S95. Solid-state structure of **12** **S97**
Figure S96–S97. Solid-state structure of **13** **S99**
Figure S98–S100. Solid-state structure of **14** **S101**
Figure S101.Comparison of the primary coordination sphere geometry between **1** and **14** **S103**
Figure S102. Solid state structure of **16** **S104**
Figure S103–106. Solid state structure of **SI-1** **S105**

Computational details **S109**
Table S5. Comparison of experimental and DFT optimized structure of **1** and **5** **S109**
Table S6. Comparison of experimental and DFT optimized structure of **1** and $(^{Me}L)Fe(OH)$ **S110**
Table S7. Experimental and Calculated ^{57}Fe Mössbauer parameters of $Fe^{II}(OH)$ and $Fe^{II}(OH)(thf)$ **S110**
Table S8. Comparison of experimental and DFT optimized structure of **5** and $(^{Me}L)Fe(HCO_3)$ **S111**
Table S9. Comparison of DFT optimized structure of **1** and two conformers of **5** **S111**
Figure S107. Optimized structure of **1** and two conformers of **5** **S112**
Table S10. DFT result of **14** by function of spin-state **S112**
Figure S108. Visualization of a dipyrrin vibrational mode of **1** **S113**
Table S11. DFT calculated acidity and basicity of $(^{Me}L)Fe(OH)$ and $(^{Me}L)Fe(OH)(I)$ **S113**

Table S12. Geometry of transition state during the reaction of (^{Em}L)Fe(X) with CO_2 **S114**
Table S13. pKa of conjugate acid and ΔG^o of (^{Em}L)Fe^{II}(X) **S114**
Table S14. Geometry of transition state during the reaction of **8** with C_2H_4 to generate **9** **S115**
Figure S109. Energy diagram of two competitive reaction pathway of **9** under CO_2 **S115**
Table S15. Coordinates of the optimized molecular structure for the (^{Em}L)Fe(OH) **S116**
Table S16. Coordinates of the optimized molecular structure for the (^{Em}L)Fe(κ^2-O,O-HCO_3) **S118**
Table S17. Coordinates of the optimized molecular structure for the (^{Em}L)Fe(κ^2-O,OH-HCO_3) **S120**
Table S18. Coordinates of the optimized molecular structure for the (^{Em}L)Fe(OH)(I) **S122**
Table S19. Coordinates of the optimized molecular structure for the [(^{Em}L)Fe(OH)]$^-$
S = 5/2 **S125**
S = 3/2 **S127**
S = 1/2 **S129**
S = 3/2, BS(4,1) **S132**
Table S20. Coordinates of the optimized molecular structure for the (^{Me}L)Fe(OH)
S = 2 **S134**
S = 1 **S134**
S = 0 (open shell) **S135**
S = 0 (closed shell) **S135**
Table S21. Coordinates of the optimized molecular structure for the (^{Me}L)Fe(OH)(thf) **S136**
Table S22. Optimized molecular structure for pKa calculation
[(^{Me}L)Fe(H_2O)]$^+$ **S136**
(^{Me}L)Fe(H_2O) **S137**
[(^{Me}L)Fe(O)]$^-$ **S137**
[(^{Me}L)Fe(H_2O)(I)]$^+$ **S138**
(^{Me}L)Fe(H_2O)(I) **S138**
[(^{Me}L)Fe(O)(I)]$^-$ **S139**
Table S23. Coordinates of the transition state of the reaction between the (^{Me}L)Fe(OH) and CO_2
S = 2 **S139**
S = 1 **S140**
S = 0 (open shell) **S140**
S = 0 (closed shell) **S141**
Table S24. Coordinates of the optimized molecular structure for the (^{Me}L)Fe(κ^2-O,O-HCO_3)
S = 2 **S141**
S = 1 **S142**
S = 0 (open shell) **S142**
S = 0 (closed shell) **S143**
Table S25. Coordinates of the optimized molecular structure for the (^{Me}L)Fe(κ^2-O,OH-HCO_3)
S = 2 **S143**
S = 1 **S144**
S = 0 (open shell) **S144**
S = 0 (closed shell) **S145**
Table S26. Coordinates of the molecular structure during the reaction between (^{Me}L)Fe(H) and CO_2

(^{Me}L)Fe(H) **S145**
Transition state **S146**
(^{Me}L)Fe(O_2CH) **S146**
Table S27. Coordinates of the molecular structure during the reaction between (^{Me}L)Fe(CH_3) and CO_2
(^{Me}L)Fe(CH_3) **S147**
Transition state **S147**
(^{Me}L)Fe(O_2CCH_3) **S148**
Table S28. Coordinates of the molecular structure during the reaction between (^{Me}L)Fe(C_2H_5) and CO_2
(^{Me}L)Fe(CH_2CH_3) **S148**
Transition state **S149**
(^{Me}L)Fe($O_2CCH_2CH_3$) **S150**
Table S29. Coordinates of the molecular structure during the reaction between (^{Me}L)Fe(NH_2) and CO_2
(^{Me}L)Fe(NH_2) **S150**
Transition state **S151**
(^{Me}L)Fe(κ^2-O,N-O_2CNH_2) **S151**
(^{Me}L)Fe(κ^2-O,O-O_2CNH_2) **S152**
Table S30. Coordinates of the molecular structure during the reaction between (^{Me}L)Fe(F) and CO_2
(^{Me}L)Fe(F) **S152**
Transition state **S153**
(^{Me}L)Fe(κ^2-O,O-O_2CF) **S153**
Table S31. Coordinates of the molecular structure during the reaction between (^{Me}L)Fe(SH) and CO_2
(^{Me}L)Fe(SH) **S154**
Transition state **S154**
(^{Me}L)Fe(κ^2-O,SH-O_2CSH) **S155**
(^{Me}L)Fe(κ^2-O,S-O_2CSH) **S155**
(^{Me}L)Fe(κ^2-O,O-O_2CSH) **S156**
Table S32. Coordinates of the molecular structure for the reaction product between (^{Me}L)Fe(OH) and CS_2
(^{Me}L)Fe(κ^2-O,S-S_2COH) **S156**
(^{Me}L)Fe(κ^2-S,SH-S_2COH) **S157**
(^{Me}L)Fe(κ^2-S,S-S_2COH) **S157**
(^{Me}L)Fe(κ^2-OH,S-S_2COH) **S158**
Table S33. Coordinates of the transition state during the reaction between (^{Me}L)Fe(H) and C_2H_4 **S158**

References **S160**

Materials and Methods

General Considerations

All manipulation of metal complexes were carried out in the absence of water and dioxygen using standard Schlenk techniques, or in an MBraun drybox under an inert dinitrogen atmosphere. All glassware was oven dried overnight and cooled in an evacuated antechamber prior to use in the drybox. Where noted, reaction equipment (including flasks, vials, pipets, and J-Young NMR tubes) were silanized by allowing a 10% v/v solution of dichlorodimethylsilane in anhydrous hexanes to be in contact with the surface for 30 minutes, washing the surface with additional hexanes, quenching with absolute ethanol, and oven drying overnight prior to use. Benzene, hexanes, toluene, diethyl ether, tetrahydrofuran, acetonitrile, and dichloromethane were dried and deoxygenated on a Glass Contour System (SG Water, Nashua, NH), and stored over 4 Å molecular sieves (Strem) prior to use. Benzene-d_6 was purchased from Cambridge Isotope Labs and was degassed and stored over 4 Å molecular sieves prior to use. Solvents *n*-Pentane, 2-methyltetrahydrofuran, 1,2-dichloroethane, 1,4-dioxane, hexamethyldisiloxane, 1.2-difluorobenzene, and pyridine were purchased from Sigma-Aldrich, distilled over potassium (2-methyltetrahydrofuran and 1,4-dioxane), distilled over calcium hydride (hexamethyldisiloxane, 1,2-difluorobenzene), and stored over 4 Å molecular sieves prior to use. Silver bis(trifluoromethanesulfonyl)imide, Thallium triflate, 1-adamantylisocyanate, zinc powder, and methyl magnesium bromide were purchased from Sigma-Aldrich and used as received.

Ferrous chloride and ferrous iodide were purchased from Strem. (^{Em}L)Fe(Cl),[1] (^{Em}L)Fe(OH),[1] Gomberg's dimer,[2] ferrocenium tetrakis(3,5-bis(trifluoromethyl)phenyl)borate ([Fc][BArF$_{24}$])[3], and $FeI_2(thf)_2$[4,5] were prepared following previously reported procedures. CO_2 gas (purity > 99.999%) and ethylene gas (purity > 99.5%) were purchased from Airgas, and Sigma-Aldrich, respectively. Benzonitrile, triethylsilane, carbon disulfide, and trimethylsilyl azide were purchased from Sigma-Aldrich, degassed by three freeze-pump-thaw cycles, and stored over 4 Å molecular sieves prior to use. 1,4-Cyclohexadiene was purchased from Sigma-Aldrich, distilled over $MgSO_4$, and stored over 4 Å molecular sieves prior to use. Potassium formate and sodium propionate were purchased from Sigma-Aldrich, and dried at room temperature in a Schlenk flask overnight under dynamic vacuum. The resulting solids were brought into the drybox, suspended in dried tetrahydrofuran, and vigorously stirred at room temperature for 3 hours. The solution was decanted and repeated two times more, then the process was repeated with diethyl ether. The resulting powder was dried *in vacuo* and stored in the drybox. Cesium fluoride was purchased from Oakwood Chemical, and dried at 130 °C in a Schlenk flask overnight under dynamic vacuum. [2.2.2]Cryptand was purchased from Sigma-Aldrich, and dried by the 4 Å molecular sieves in diethyl ether solution for a 3 days followed by removal of the solvent *in vacuo*. Trityl chloride was purchased from Sigma-Aldrich, and recrystallized from boiling hexanes. Iodine was purchased from MilliporeSigma, and purified by sublimation. Tetrabutylammonium hexafluoriphosphate ([TBA][PF_6]) was purchased from Sigma-Aldrich, and purified by recrystallization from boiling ethanol under air followed by second recrystallization from boiling tetrahydrofuran under inert condition. Celite 545 (J. T. Baker) and Silica gel 32-63 μ (AIC, Framingham, MA) was dried in a Schlenk flask for 24 hours under dynamic vacuum while heating to at least 150 °C prior to use.

Characterization and Physical Measurements

^{1}H, ^{19}F, and ^{13}C NMR spectra were acquired on Varian Unity/Inova500 spectrometer. ^{1}H NMR and ^{13}C NMR chemical shifts were referenced to the chemical shift of residual solvent peaks.

^{19}F NMR chemical shifts were referenced to an external standard of boron trifluoride diethyl etherate (–153 ppm). Integration and assignment of ^{1}H NMR of paramagnetic three-coordinate (^{Em}L)Fe^{II} complexes were referred to (^{Em}L–d_{24})FeCl[1] and (β-diketiminate)Fe^{II}.[6,7,8]

Zero-field ^{57}Fe Mössbauer spectra were measured with a constant acceleration spectrometer (SEE Co, Minneapolis, MN). Samples were prepared by dissolving the analyte in benzene, benzene-d_6, or suspending the ground analyte in Paratone oil and immobilizing by rapid freezing in liquid nitrogen. Isomer shifts are reported relative to α-iron foil at room temperature. Data were analyzed and simulated using Igor Pro 6 software (WaveMetrics, Portland, OR) via Lorentzian fitting functions.

Magnetic data was collected using a Quantum Design MPMS 3 SQUID magnetometer. Measurements were obtained on powders restrained in a frozen eicosane matrix within a flame sealed high purity borosilicate NMR tube. The direct current (dc) magnetization measurements were obtained in the temperature range of 2 – 300 K under a field of 1 T. The susceptibility data was corrected for the diamagnetic contributions from the sample holder, eicosane, and the core electrons of the sample using Pascal's constants.[9]

Infrared (IR) spectra were acquired on a Varian 1000 FT-IR spectrometer using ATR mode under an Argon atmosphere. Elemental analyses (%CHN) were carried out by Midwest Microlab (Indianapolis, IN) or at Harvard University on a PerkinElmer 2400 series II CHNS/O Analyzer using bulk recrystallized compounds. In several cases, satisfactory elemental analysis were obtained by considering the presence of solvent molecules intercalated within the unit cell as ascertained by single-crystal X-ray diffraction and ^{1}H NMR spectroscopy. High-resolution mass spectrometry (HRMS) measurements were recorded using a Thermo Scientific Dionex UltiMate 3000 UHPLC coupled to a Thermo Q Exactive Plus mass spectrometer system equipped with a HESI-II electrospray ionization source at the Harvard Center for Mass Spectrometry facilities. EPR spectra were obtained on a Bruker EleXsys E-500 CW-EPR spectrometer at a microwave power of 0.6325–2 mW. Spectra were measured as frozen toluene glasses by inserting a finger liquid nitrogen dewar into the cavity. Spectral simulations incorporating spin state and rhombicity were performed using VisualRhombo.[10]

Syntheses

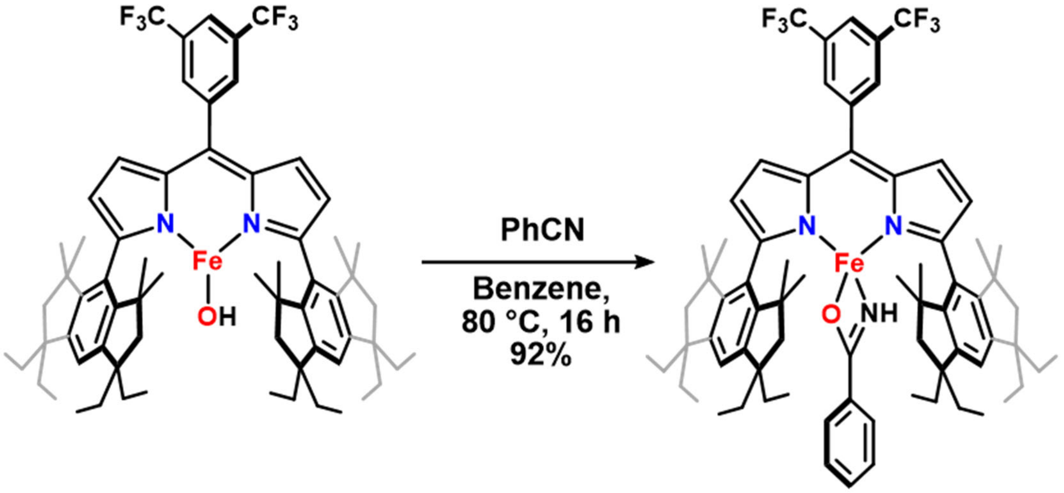

(^{Em}L)Fe(κ^2-N,O-NHC(O)Ph) (2): In the drybox, a solution of (^{Em}L)Fe(OH) (21.6 mg, 0.0201 mmol) and benzonitrile (2.24 mg, 0.0217 mmol) in 2 mL benzene was transferred to a Schlenk tube. The Schlenk tube was sealed, and stirred at 80 °C for 16 hours, and the volatiles were removed *in vacuo*. The resulting solids were dissolved in hexanes, and filtered through Celite in hexanes. The product was obtained as red powder after the volatiles were removed *in vacuo* (21.8 mg, 0.0185 mmol, 92%). ^{1}H NMR (500 MHz, 298 K, C_6D_6): δ 56.14 (2H), 51.81 (4H), 39.75 (1H), 32.40 (2H), 18.30 1H), 9.31 (1H), 8.81 (4H), 7.20 (12H), 5.58 (2H), –0.29 (4H), –0.58 (1H), –1.87 (1H), –1.94 (2H), –2.08 (12 H), –2.31 (4H), –9.75 (4H), –11.05 (12H), –13.20 (4H), –67.84 (12H) ppm. ^{19}F NMR (471 MHz, 298 K, C_6D_6): δ –53.82 ppm. FTIR (ATR): υ(N–H) = 3418 cm^{-1}. Zero-field ^{57}Fe Mossbauer (90 K) (δ, $|\Delta E_Q|$ (mm/s)): 0.86, 2.05 (γ = 0.31 mm/s). HRMS (ESI$^+$): m/z Calc. 1180.6175 $[C_{72}H_{87}F_6FeN_3O+H]^+$, Found 1180.6170 $[M+H]^+$.

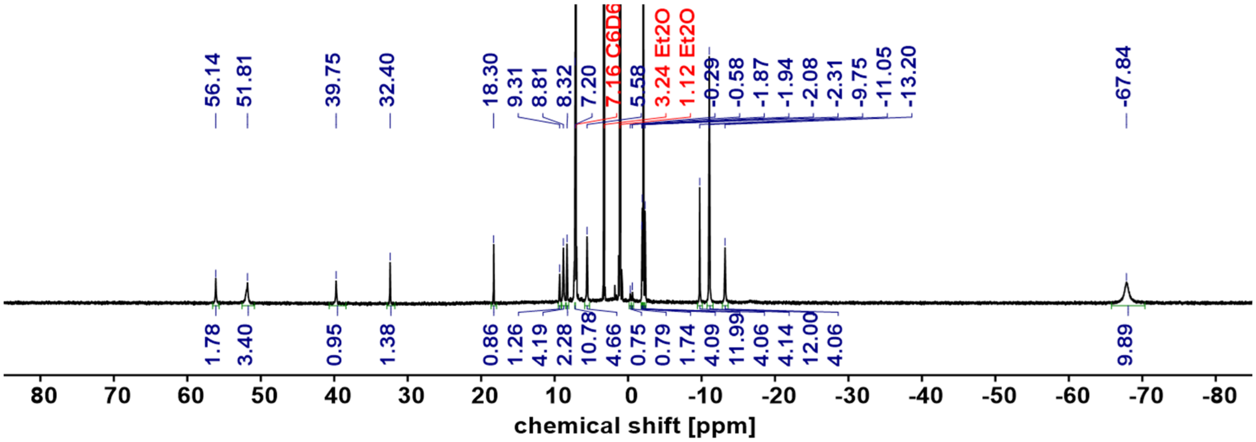

Figure S1. ^{1}H NMR spectrum of (^{Em}L)Fe(κ^2N,O-NHC(O)Ph), (500 MHz, 298 K, C_6D_6).

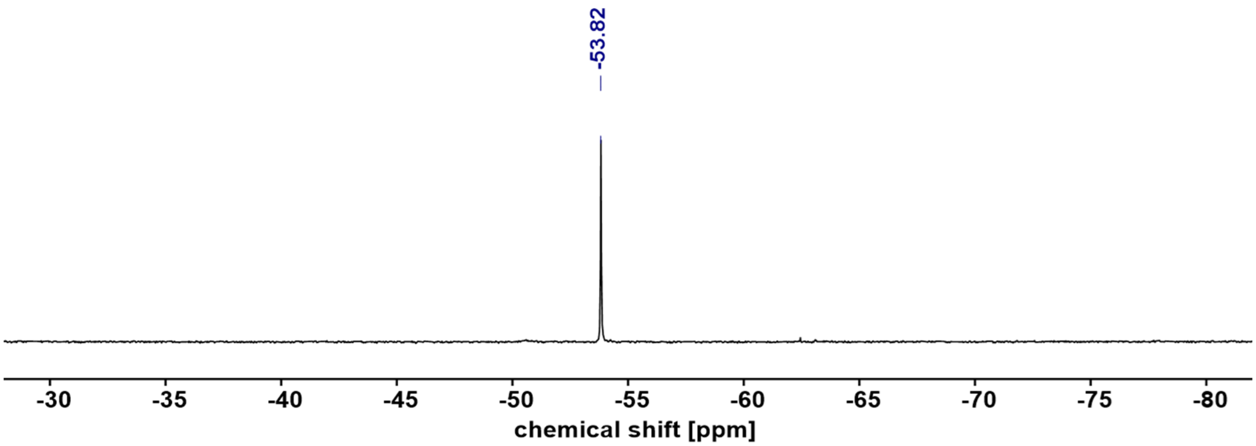

Figure S2. ^{19}F NMR spectrum of (^{Em}L)Fe(κ^2N,O-NHC(O)Ph), (471 MHz, 298 K, C_6D_6).

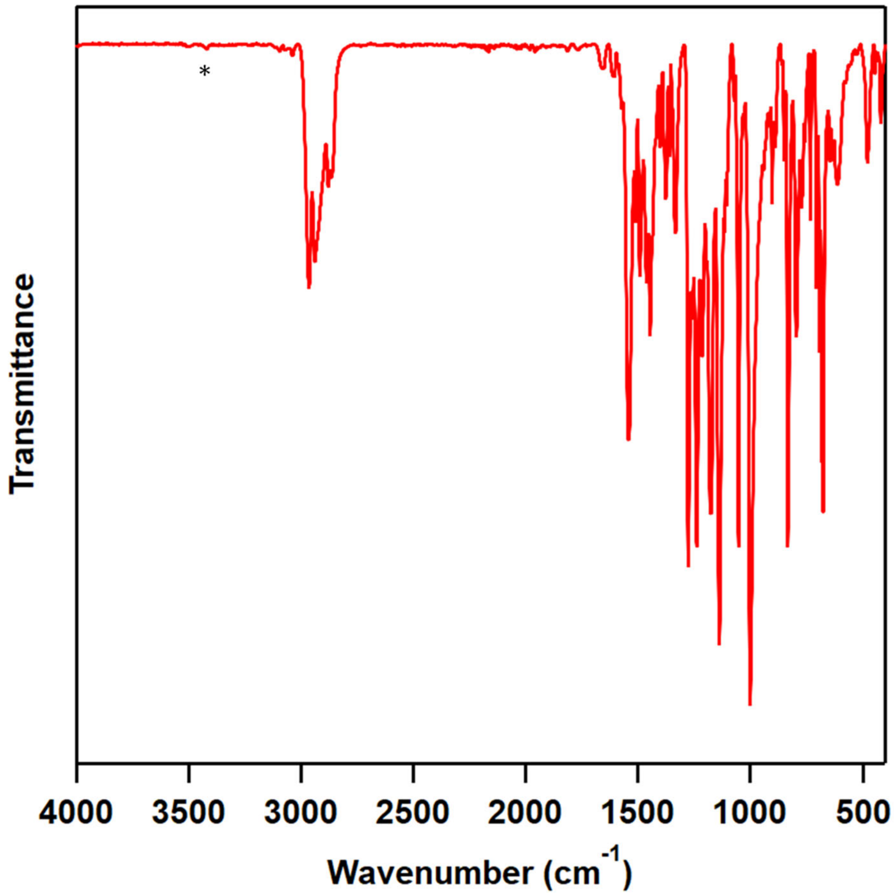

Figure S3. IR spectrum of (^{Em}L)Fe(κ^2N,O-NHC(O)Ph). N–H vibration modes (υ(N–H) = 3418 cm^{-1}) are denoted with asterisks (*).

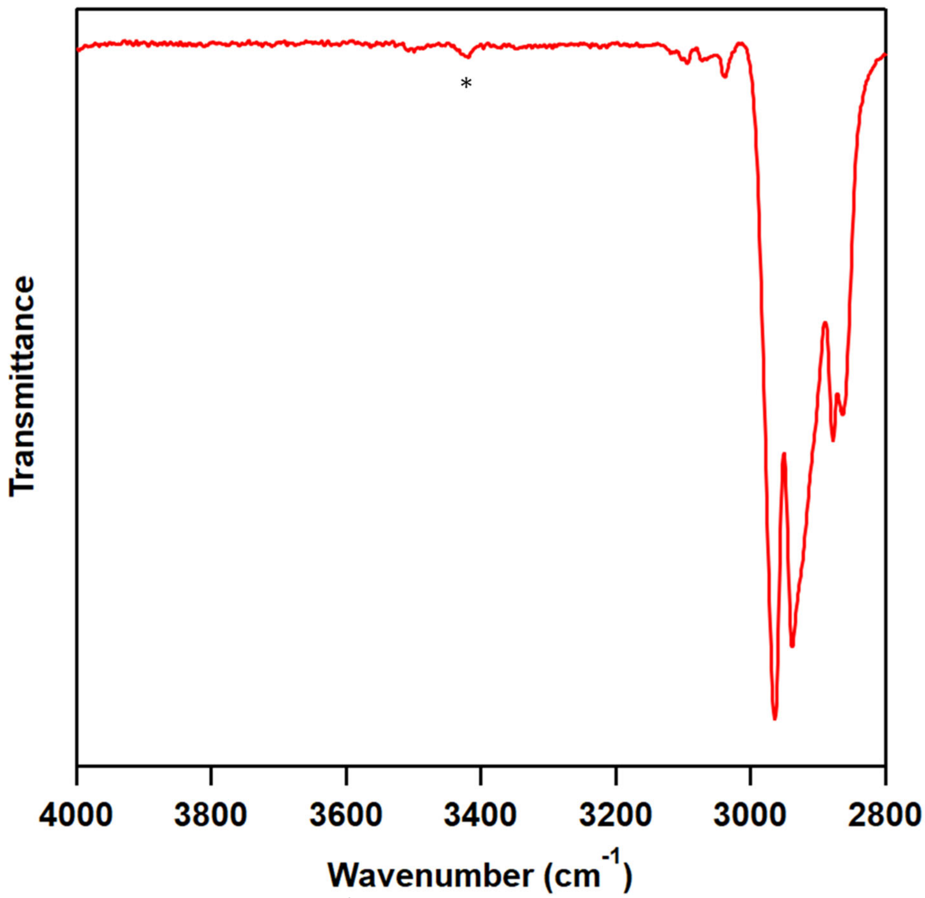

Figure S4. IR spectrum of (^{Em}L)Fe(κ^2N,O-NHC(O)Ph) highlighting N–H vibration. N–H vibration modes (υ(N–H) = 3418 cm^{-1}) are denoted with asterisks (*).

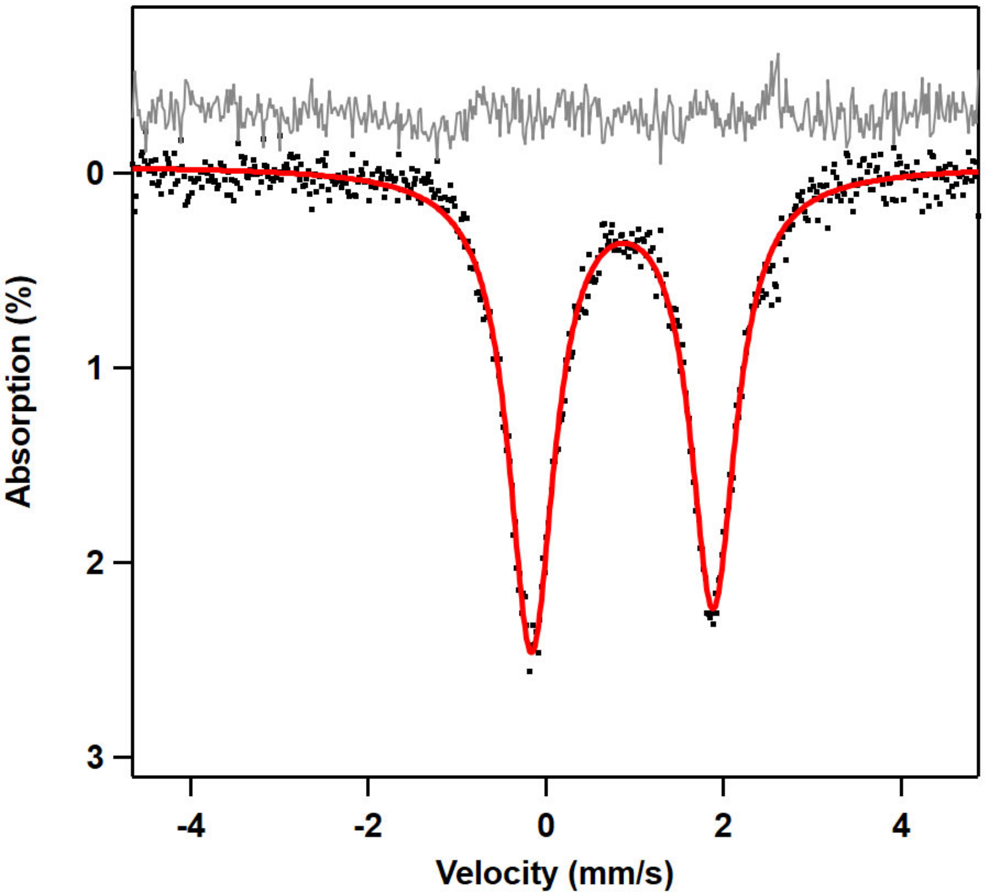

Figure S5. Zero-field ^{57}Fe Mössbauer spectrum of (^{Em}L)Fe(κ^2N,O-NHC(O)Ph) at 90 K. Isomer shift and quadrupole splitting are reported relative to α-iron foil at room temperature. The black dot, red line, and gray line represent the experimental data, fit, and residuals, respectively. δ (mm/s), $|\Delta E_Q|$ (mm/s), γ (mm/s) = 0.86, 2.05, 0.31.

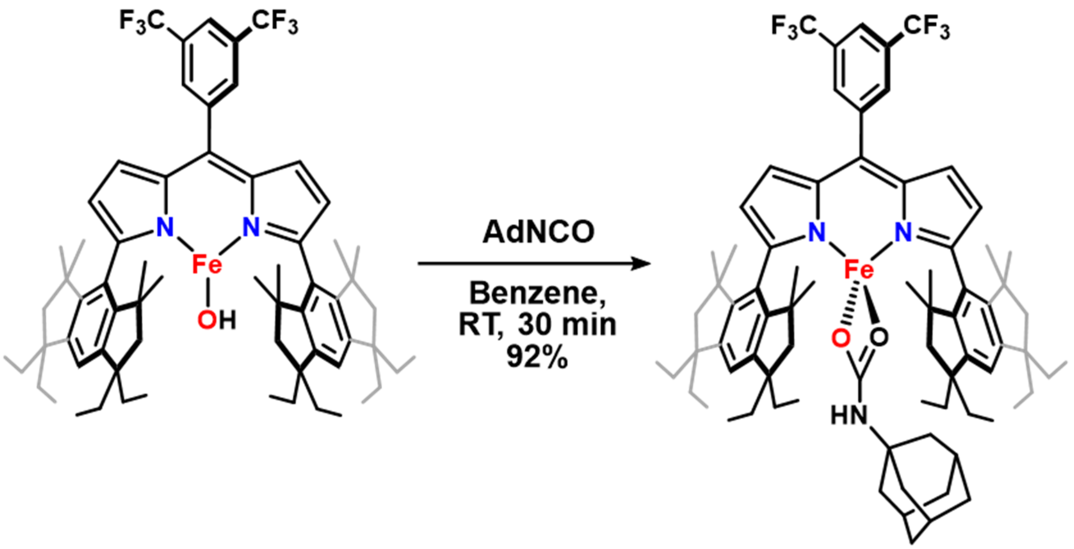

(^{Em}L)Fe(κ^2O,O-O_2CN(H)Ad) (3): A solution of (^{Em}L)Fe(OH) (44.6 mg, 0.0414 mmol) and 1-adamantylisocyanate (7.6 mg, 0.043 mmol) in 3 mL benzene was stirred at room temperature for 30 minutes, and the volatiles were removed *in vacuo*. The resulting solids were dissolved in *n*-pentane and filtered through Celite in *n*-pentane. The product was obtained as red powder after the volatiles were removed *in vacuo* (48.0 mg, 0.0383 mmol, 92%). Crystals suitable for X-ray diffraction were obtained from a concentrated solution of **3** in *n*-pentane. ^{1}H NMR (500 MHz, 298 K, C_6D_6): δ 74.13 (2H), 60.51 (2H), 56.88 (6H), 53.36 (2H), 24.89 (1H), 21.84 (3H), 20.58 (3H), 15.60 (3H), 8.12 (12H+6H), 6.55 (4H), 4.80 (2H), –5.52 (12H), –5.94 (4H), –7.27 (4H), –16.94 (4H), –19.82 (12H+3H), –111.06 (12H) ppm. ^{19}F NMR (471 MHz, 298 K, C_6D_6): δ –48.19 ppm. Zero-field ^{57}Fe Mossbauer (90 K) (δ, $|\Delta E_Q|$ (mm/s)): 0.94, 1.41 (γ = 0.20 mm/s). FTIR (ATR): υ(N–H) 3442 cm^{-1}. HRMS (ESI$^-$): m/z Calc. 1298.6280 [$C_{76}H_{97}F_6FeN_3O_2$+HCOO]$^-$, Found 1298.6798 [M+HCO_2]$^-$.

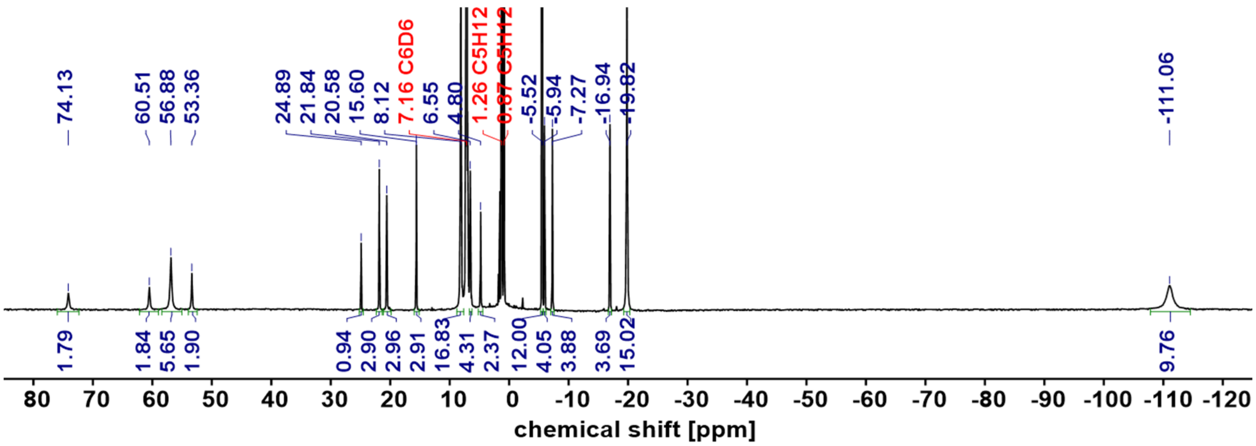

Figure S6. ^{1}H NMR spectrum of (^{Em}L)Fe(κ^2O,O-O_2CN(H)Ad), (500 MHz, 298 K, C_6D_6).

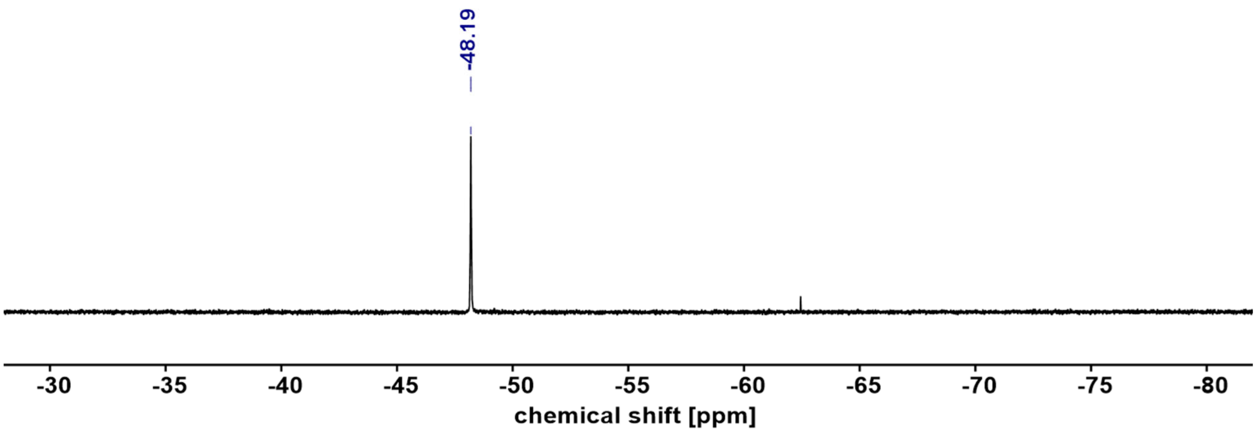

Figure S7. ^{19}F NMR spectrum of (^{Em}L)Fe(κ^2O,O-O_2CN(H)Ad), (471 MHz, 298 K, C_6D_6).

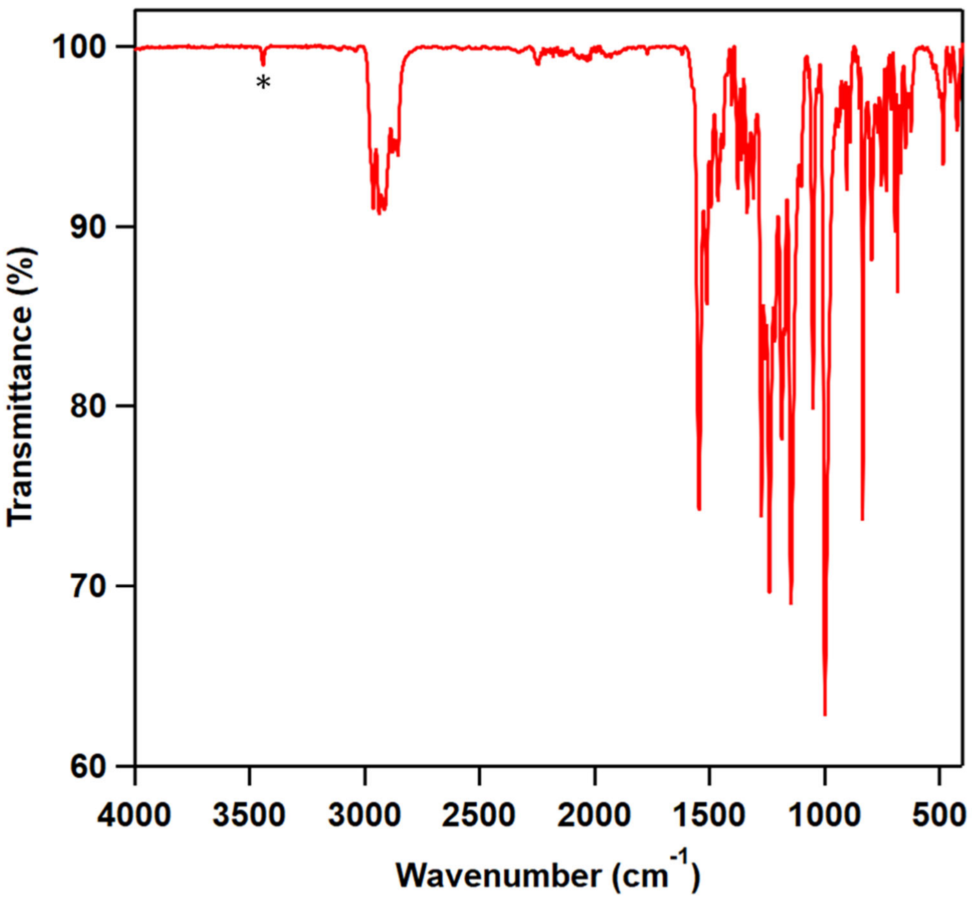

Figure S8. IR spectrum of (^{Em}L)Fe(κ^2O,O-O_2CN(H)Ad). N–H vibration modes (υ(N–H) = 3442 cm^{-1}) are denoted with asterisks (*).

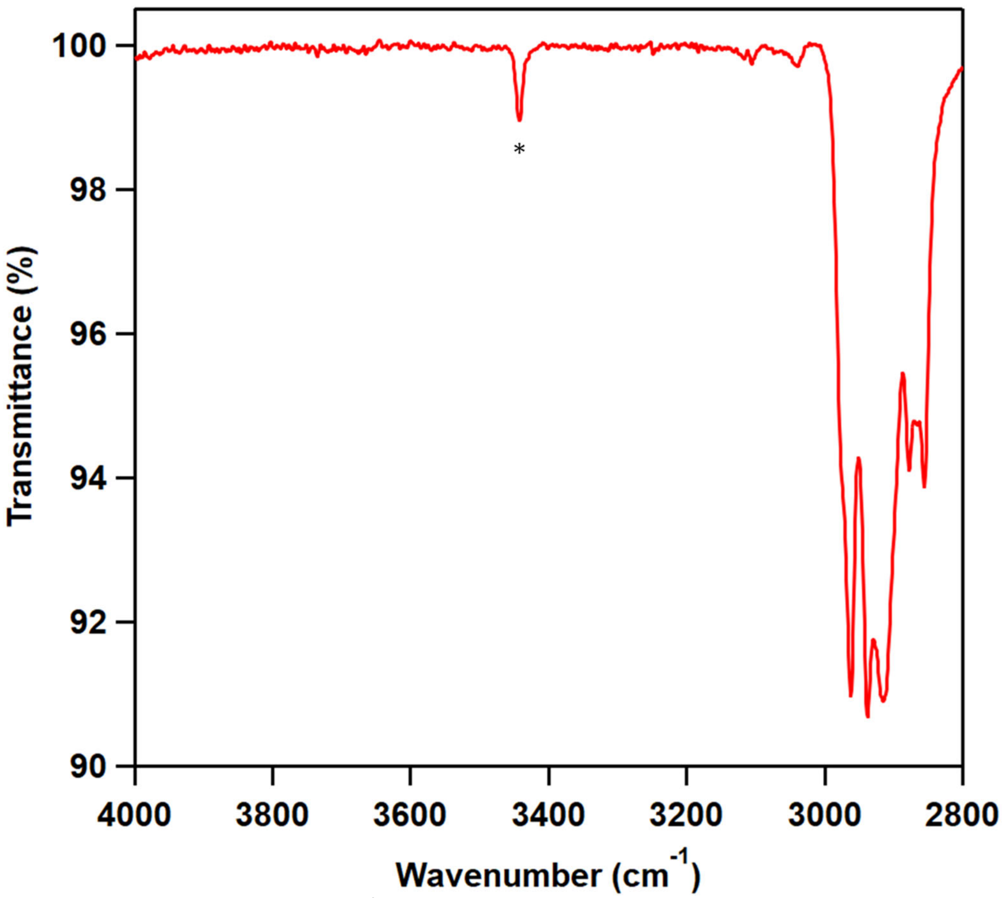

Figure S9. IR spectrum of (^{Em}L)Fe(κ^2O,O-O_2CN(H)Ad) highlighting N–H vibrational mode. N–H vibration modes (υ(N–H) = 3442 cm^{-1}) are denoted with asterisks (*).

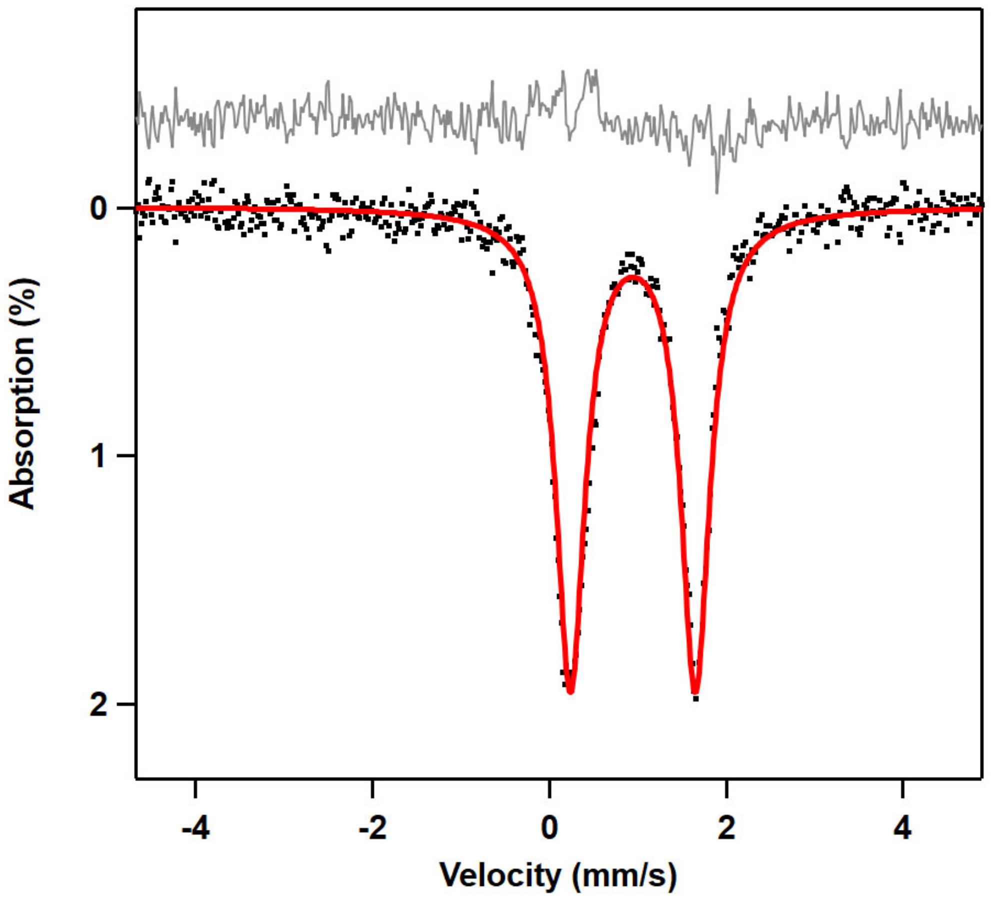

Figure S10. Zero-field ^{57}Fe Mössbauer spectrum of (^{Em}L)Fe(κ^2O,O-O_2CN(H)Ad) at 90 K. Isomer shift and quadrupole splitting are reported relative to α-iron foil at room temperature. The black dot, red line, and gray line represent the experimental data, fit, and residuals, respectively. δ (mm/s), $|\Delta E_Q|$ (mm/s), γ (mm/s) = 0.94, 1.41, 0.20.

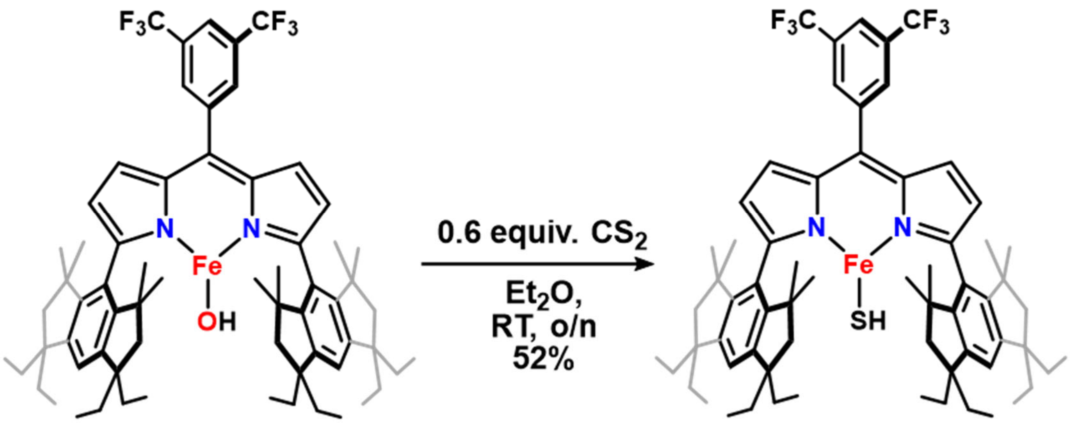

(^{Em}L)Fe(SH) (4): To a solution of (^{Em}L)Fe(OH) (23.6 mg, 0.0219 mmol) in 1 mL diethyl ether was added a solution of carbon disulfide (1.0 mg, 0.013 mmol) in 1 mL diethyl ether. The resulting solution was stirred at room temperature for 24 hours, and the volatiles were removed *in vacuo*. The resulting solids were dissolved in hexanes, and filtered through Celite in hexanes. The product was obtained as red crystals by recrystallization from a concentrated solution of **4** in *n*-pentane at –35 °C (14.2 mg, 0.0130 mmol, 59%). ^{1}H NMR (500 MHz, 298 K, C_6D_6): δ 77.79 (2H), 65.86 (2H), 62.23 (2H), 25.84 (1H), 21.43 (12H), 17.33 (4H), 13.97 (4H), 13.63 (2H), –1.68 (4H), –1.87 (4H), –2.97 (12H), –17.98 (4H), –21.70 (12H), –24.90 (4H), –125.91 (12H) ppm. ^{19}F NMR (471 MHz, 298 K, C_6D_6): δ –47.61 ppm. Zero-field ^{57}Fe Mossbauer (90 K) (δ, $|\Delta E_Q|$ (mm/s)): 0.63, 0.33 (γ = 0.16 mm/s). HRMS (ESI$^-$): m/z Calc. 1137.5436 [$C_{65}H_{82}F_6FeN_2S$+HCOO]$^-$, Found 1137.5417 [M+HCO_2]$^-$.

Note: The reaction of (^{Em}L)Fe(OH) with excess CS_2 generated **4** cleanly without any impurities. The reaction was completed in 12 hours at room temperature when 1.5 equiv of CS_2 was used.

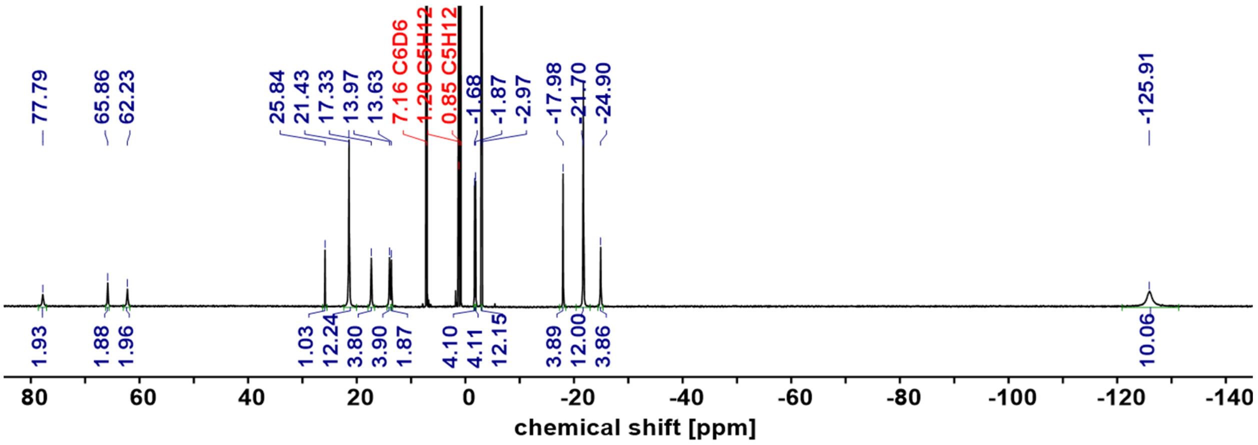

Figure S11. ^{1}H NMR spectrum of (^{Em}L)Fe(SH), (500 MHz, 298 K, C_6D_6).

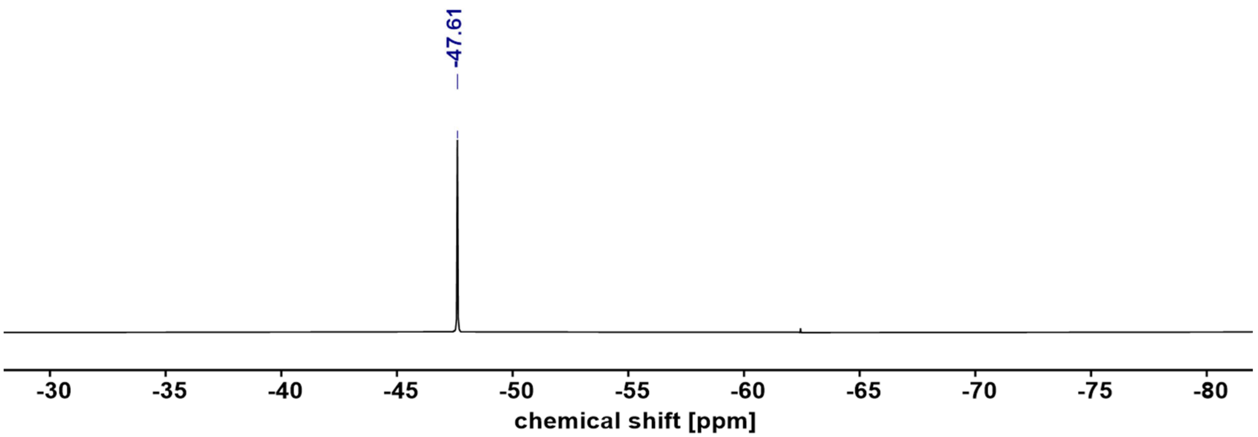

Figure S12. ^{19}F NMR spectrum of (^{Em}L)Fe(SH), (471 MHz, 298 K, C_6D_6).

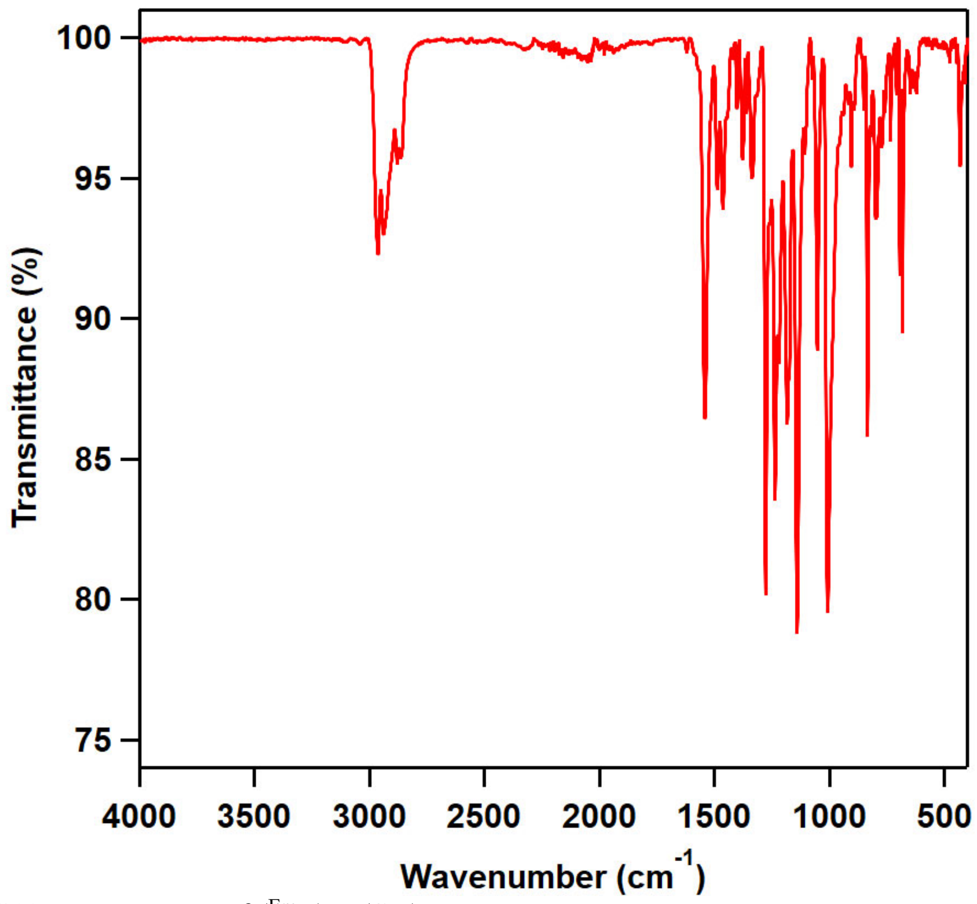

Figure S13. IR spectrum of (^{Em}L)Fe(SH).

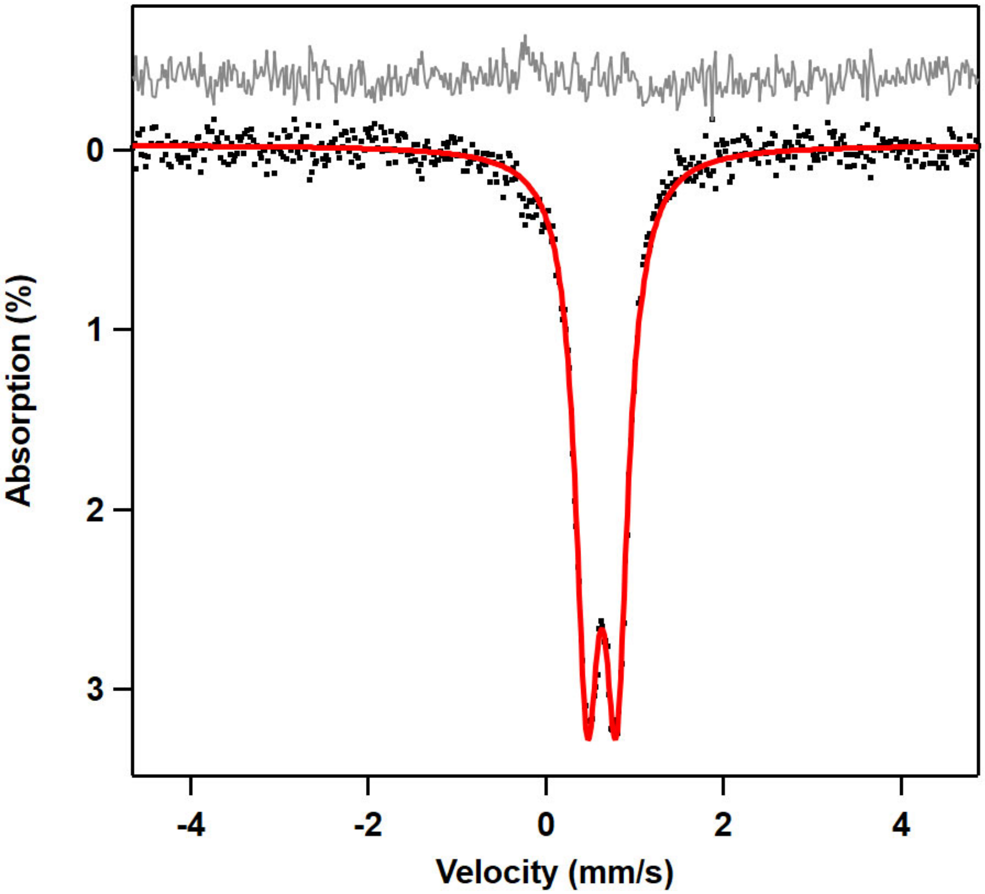

Figure S14. Zero-field ^{57}Fe Mössbauer spectrum of (^{Em}L)Fe(SH) at 90 K. Isomer shift and quadrupole splitting are reported relative to α-iron foil at room temperature. The black dot, red line, and gray line represent the experimental data, fit, and residuals, respectively. δ (mm/s), $|\Delta E_Q|$ (mm/s), γ (mm/s) = 0.63, 0.33, 0.16.

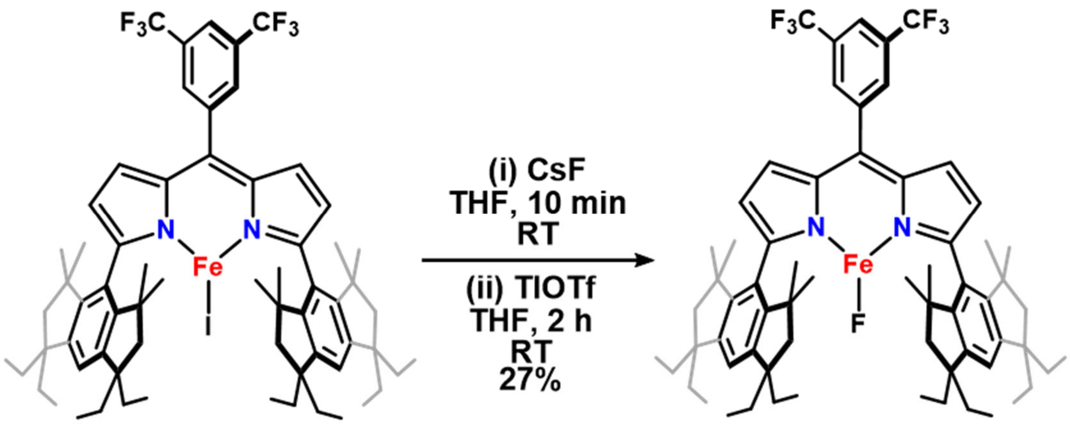

(^{Em}L)Fe(F) (6): A mixture of (^{Em}L)Fe(I) (26.2 mg, 0.0221 mmol) and CsF (3.6 mg, 0.024 mmol) in 1 mL THF were stirred at room temperature for 10 minutes. To the mixture, slowly added a solution of thallium triflate (8.9 mg, 0.025 mmol) in 2 mL THF. The resulting mixture changed color from red to orange in a minute, then turned to red in 5 minutes. The resulting mixture was stirred at room temperature for 1 hour, filtered through Celite in THF, and the volatiles were removed *in vacuo*. The resulting power was triturated with hexanes three times and filtered through Celite in hexanes. The product was obtained as red crystals by recrystallization from a concentrated solution of **6** in *n*-pentane at –35 °C (7.4 mg, 0.0069 mmol, 31%). ^{1}H NMR (500 MHz, 298 K, C_6D_6): δ 85.30 (2H), 74.53 (2H), 53.01 (2H), 42.99 (12H), 29.44 (1H), 24.48 (4H), 20.69 (4H), 16.33 (2H), –2.25 (4H), –3.46 (4H), –5.25 (12H), –22.29 (4H), –27.35 (4H), –31.82 (12H), –125.11 (12H) ppm. ^{19}F NMR (471 MHz, 298 K, C_6D_6): δ –44.29 ppm. Zero-field ^{57}Fe Mossbauer (90 K) (δ, $|\Delta E_Q|$ (mm/s)): 0.82, 1.03 (γ = 0.22 mm/s). HRMS (ESI$^+$): m/z Calc. 1101.5531 $[C_{65}H_{81}F_7FeN_2+Na]^+$, Found 1101.5486 $[M+Na]^+$.

Note. The synthesis of (^{Em}L)FeF is sensitive to the reaction scale in our hands. When the reaction was conducted with more than 60 mg of (^{Em}L)Fe(I), we observed a generation of new ferrous species by ^{57}Mössbauer spectrum (δ, $|\Delta E_Q|$, γ (mm/s) = 1.02, 2.58, 0.22) (Figure S20). The same impurity was observed when TlOTf was added to (^{Em}L)Fe(I) followed by adding fluoride source, CsF. We confirmed that the impurity does not originate from excess CsF by adding CsF to (^{Em}L)FeF as a control. The new ferrous impurity cannot be removed from the reaction mixture, but was cleanly isolated using recrystallization. The molecule was characterized as a thallium salt of ferrous difluoride, (^{Em}L)Fe(F)$_2$(Tl) (**SI-2**) by solid-state structure using single crystal x-ray diffraction (Figure S103). ^{1}H NMR (500 MHz, 298 K, C_6D_6): δ 48.86 (2H), 41.37 (2H), 38.86 (2H), 18.48 (1H), 12.82 (12H), 12.55 (4H), 10.44 (4H), 9.57 (2H), –2.53 (4H), –2.65 (12H), –3.37 (4H), –10.63 (4H), –12.70 (4H), –15.02 (12H), –52.11 (12H) ppm. ^{19}F NMR (471 MHz, 298 K, C_6D_6): δ –53.38 ppm. Zero-field ^{57}Fe Mossbauer (90 K) (δ, $|\Delta E_Q|$ (mm/s)): 1.02, 2.58 (γ = 0.22 mm/s).

Note. To scale up the reaction without generating **SI-2** as an impurity, we screened other fluoride source. Interestingly, hexafluorophosphate salt ([K][PF_6], [TBA][PF_6]) with halide abstraction reagent (TlOTf, AgOTf) or $TlPF_6$ generated (^{Em}L)Fe(F), but these syntheses proceeded with formation of unidentified impurities.

Note. The reaction of (^{Em}L)Fe(I) with [TBA][Ph_3SiF_2] affords (^{Em}L)Fe(F) quantitatively by ^{19}F NMR spectroscopy, but cannot remove organic impurity, Ph_3SiF.

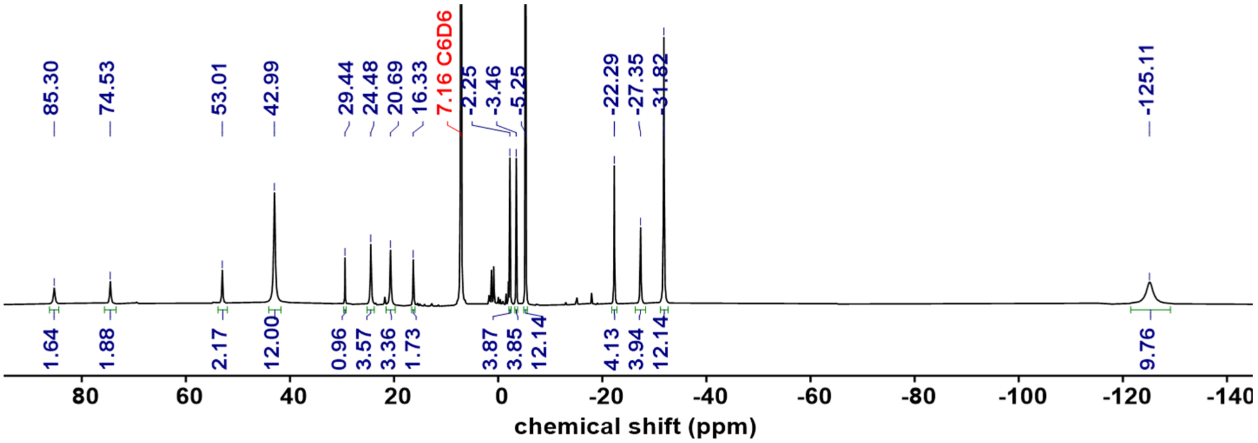

Figure S15. ^{1}H NMR spectrum of (^{Em}L)Fe(F), (500 MHz, 298 K, C_6D_6).

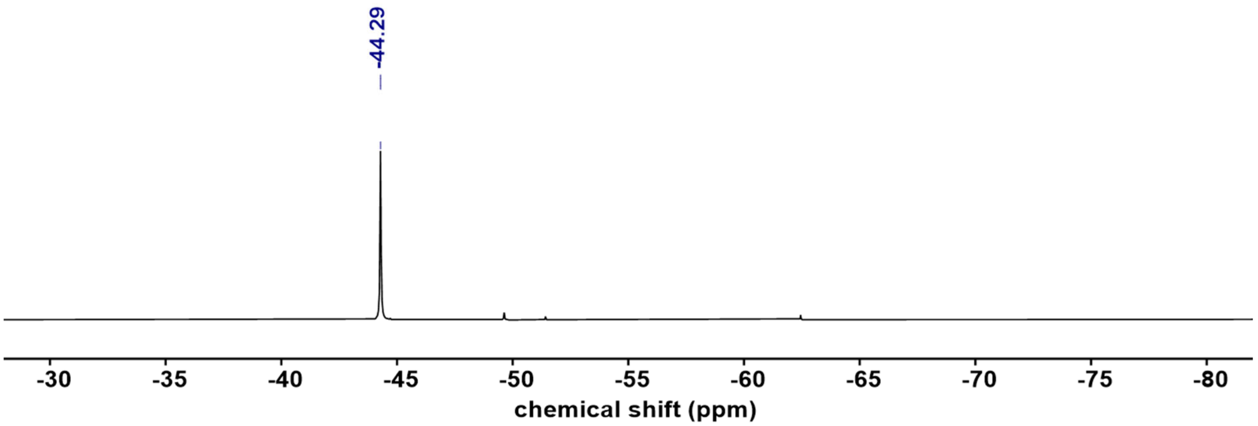

Figure S16. ^{19}F NMR spectrum of (^{Em}L)Fe(F), (471 MHz, 298 K, C_6D_6).

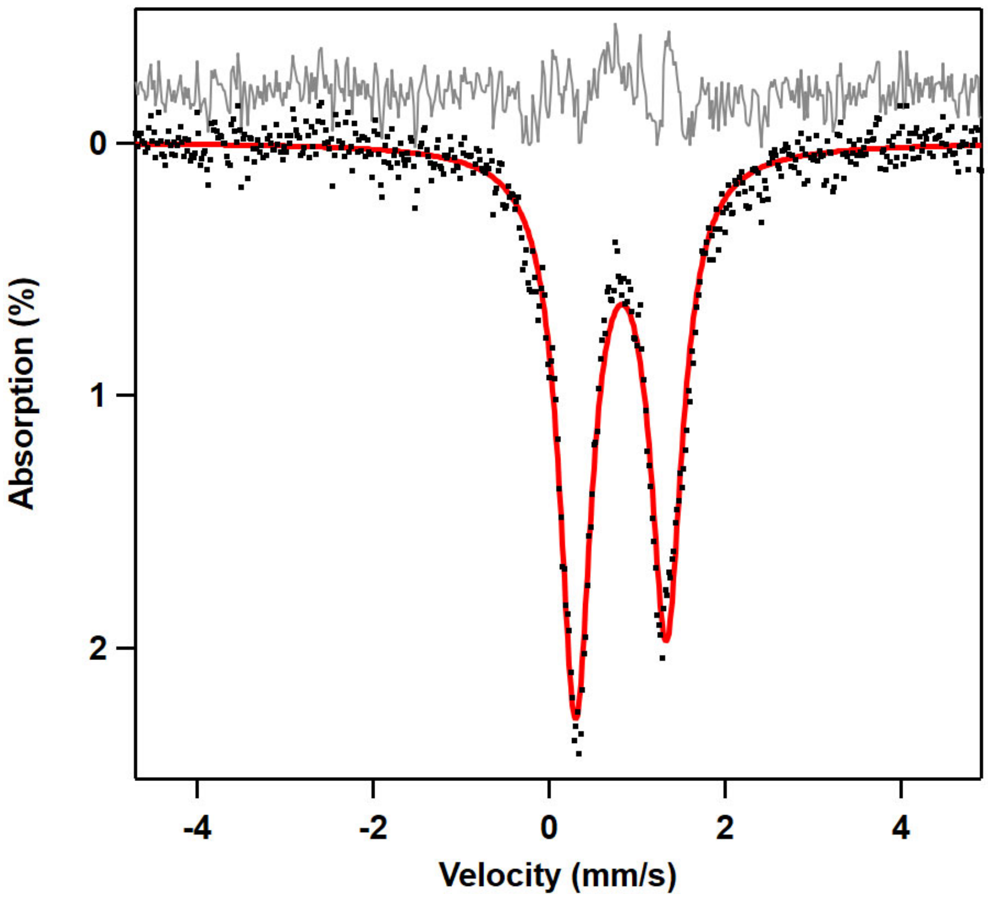

Figure S17. Zero-field ^{57}Fe Mössbauer spectrum of (^{Em}L)Fe(F) at 90 K. Isomer shift and quadrupole splitting are reported relative to α-iron foil at room temperature. The black dot, red line, and gray line represent the experimental data, fit, and residuals, respectively. δ (mm/s), $|\Delta E_Q|$ (mm/s), γ (mm/s) = 0.82, 1.03, 0.22.

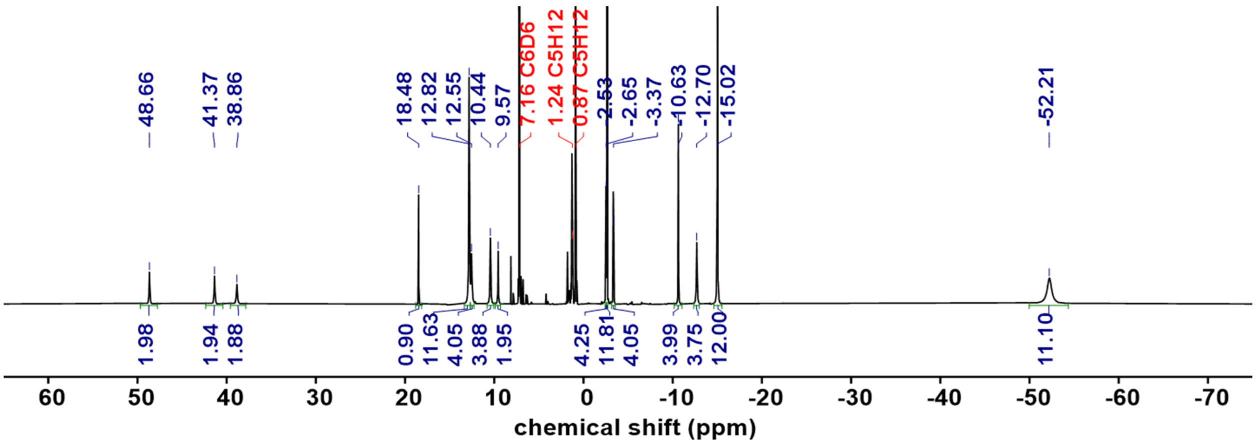

Figure S18. ^{1}H NMR spectrum of (^{Em}L)Fe(F)$_2$(Tl), (500 MHz, 298 K, C_6D_6).

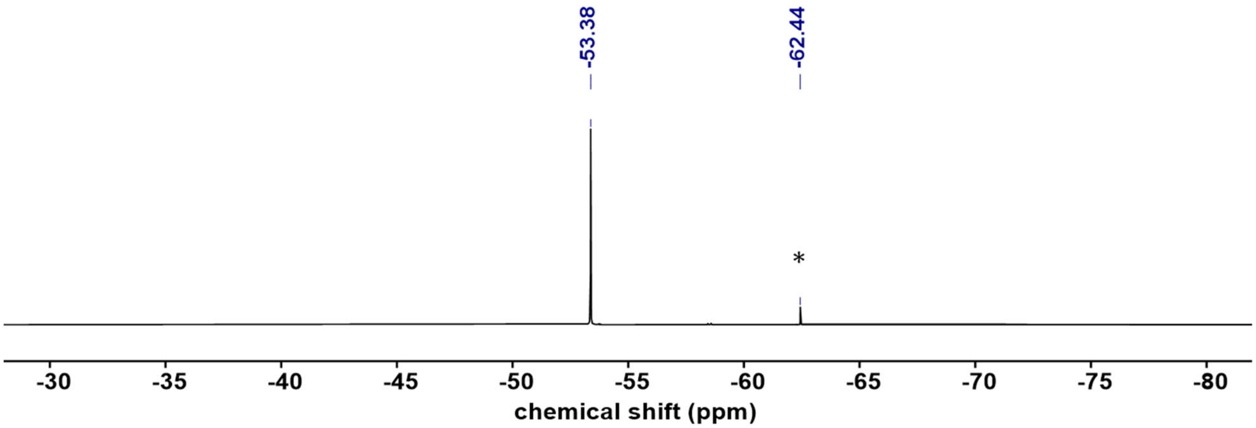

Figure S19. ^{19}F NMR spectrum of (^{Em}L)Fe(F)$_2$(Tl), (471 MHz, 298 K, C_6D_6). (^{Em}L)H were obtained as minor impurity (ca. 3 %) as denoted with asterisk (*), which was unable to purify by recrystallization. No ^{19}F resonances are detected from the fluorides bound to the Fe(II) ion, based on the positioning of the one observed main resonance as relative to other (^{Em}L)Fe(X) complexes.

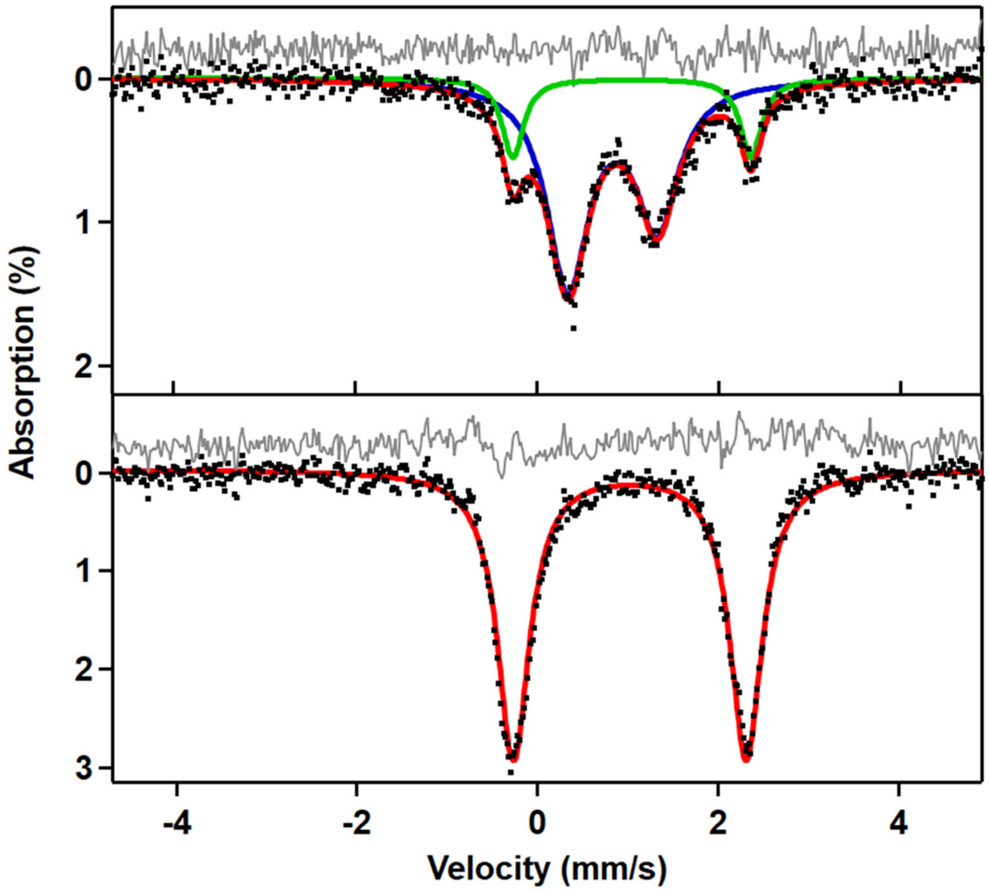

Figure S20. Zero-field ^{57}Fe Mössbauer spectrum of (^{Em}L)FeF synthesis in large scale (top), and (^{Em}L)Fe(F)$_2$(Tl) (bottom) at 90 K. Isomer shift and quadrupole splitting are reported relative to α-iron foil at room temperature. The black dot, red line, and gray line represent the experimental data, fit, and residuals, respectively. The blue and green line on top spectrum represent fitted quadrupole doublets of (^{Em}L)Fe(F) (69 %) (δ, $|\Delta E_Q|$, γ (mm/s) = 0.83, 0.98, 0.28), and (^{Em}L)Fe(F)$_2$(Tl) (31 %) (δ, $|\Delta E_Q|$, γ (mm/s) = 1.05, 2.62, 0.15), respectively. The parameters for the isolated impurity, (^{Em}L)Fe(F)$_2$(Tl), on the bottom spectrum are δ (mm/s), $|\Delta E_Q|$ (mm/s), γ (mm/s) = 1.02, 2.58, 0.22.

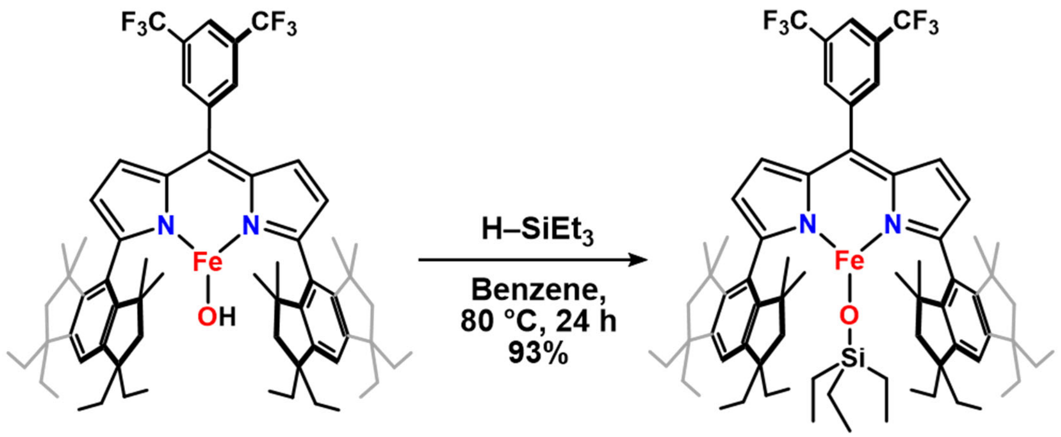

(^{Em}L)Fe(OSiEt$_3$) (7): In the drybox, a solution of (^{Em}L)Fe(OH) (128.4 mg, 0.119 mmol) and triethylsilane (20.0 mg, 0.172 mmol) was transferred to a Schlenk tube. The Schlenk tube was sealed and stirred at 80 °C for 24 hours. The volatiles were removed *in vacuo* and dissolved in hexanes. The resulting solution was filtered through Celite in hexanes. The product was obtained as red powder after the volatiles were removed *in vacuo* (132.0 mg, 0.110 mmol, 93%). Crystals suitable for X-ray diffraction were obtained from a reverse-vapor diffusion of concentrated solution of 7 in *n*-pentane into hexamethyldisiloxane at –35 °C. ^{1}H NMR (500 MHz, 298 K, C_6D_6): δ 75.11 (2H), 74.33 (6H, –Si(CH_2CH$_3$)$_3$), 62.99 (2H), 43.40 (9H, –Si(CH$_2$CH_3)$_3$), 25.84 (1H), 5.98 (4H), 3.42 (4H), 2.79 (12H), –5.64 (12H), –6.50 (4H), –8.27 (4H), –17.31 (4H), –18.38 (4H), –21.37 (12H), –114.63 (12H) ppm. ^{19}F NMR (471 MHz, 298 K, C_6D_6): δ –47.53 ppm. Zero-field ^{57}Fe Mossbauer (90 K) (δ, $|\Delta E_Q|$ (mm/s)): 0.73, 0.52 (γ = 0.16 mm/s). HRMS (ESI$^-$): m/z Calc. 1190.6553 [$C_{71}H_{96}F_6FeN_2OSi$]$^-$, Found 1190.6544 [M]$^-$.

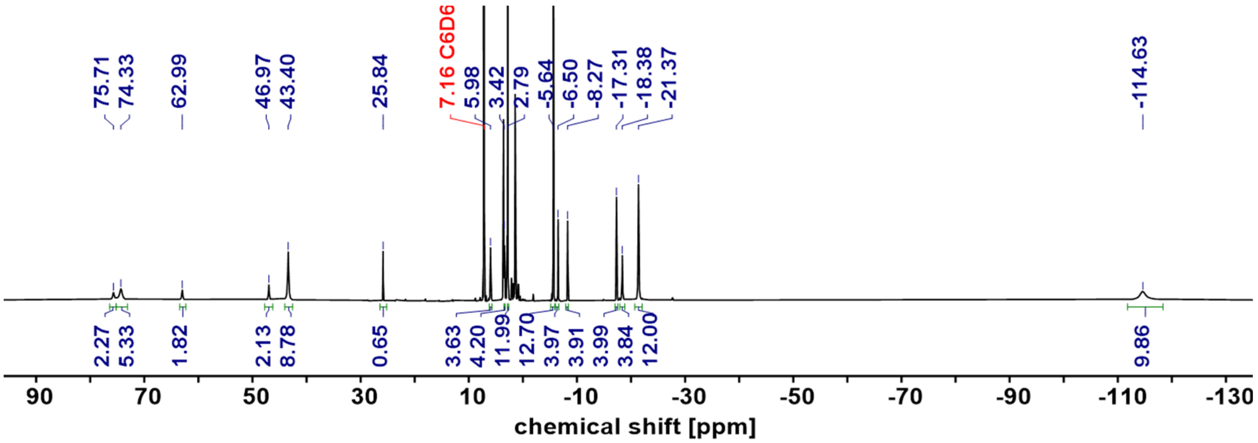

Figure S21. ^{1}H NMR spectrum of (^{Em}L)Fe(OSiEt$_3$), (500 MHz, 298 K, C_6D_6).

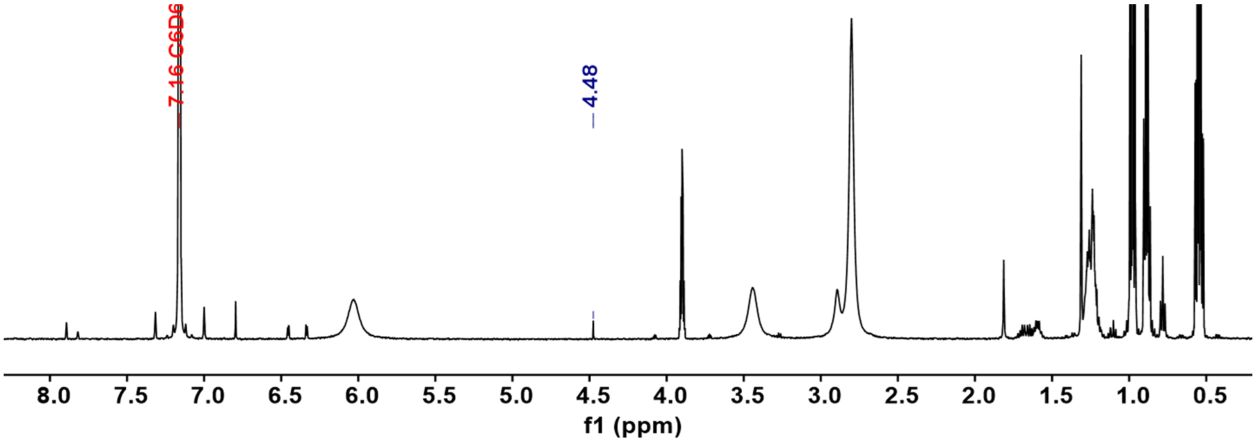

Figure S22. ^{1}H NMR spectrum highlighting H_2 generation (4.48 ppm) during the reaction of (^{Em}L)Fe(OH) with $HSiEt_3$ in a sealed J-Young NMR tube, (500 MHz, 298 K, C_6D_6).

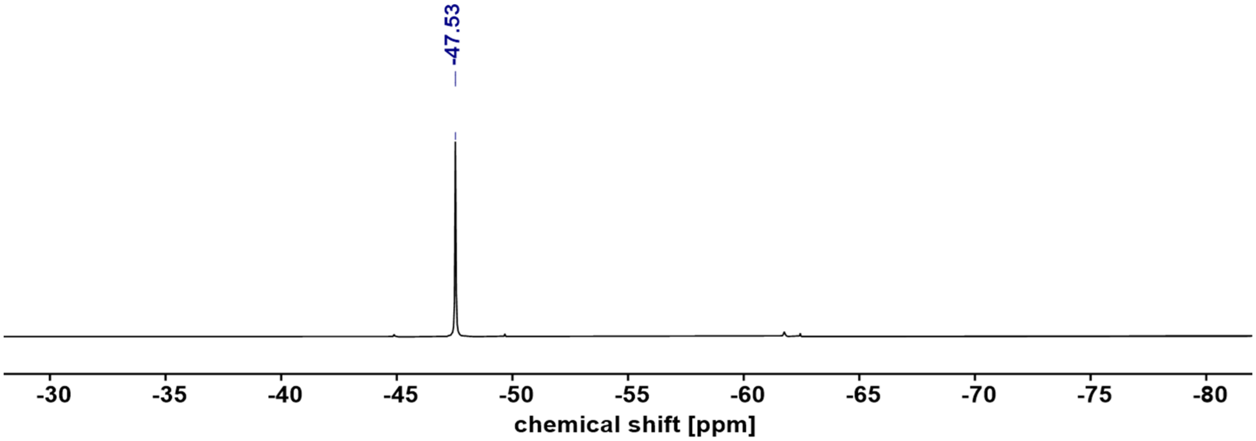

Figure S23. ^{19}F NMR spectrum of (^{Em}L)Fe($OSiEt_3$), (471 MHz, 298 K, C_6D_6).

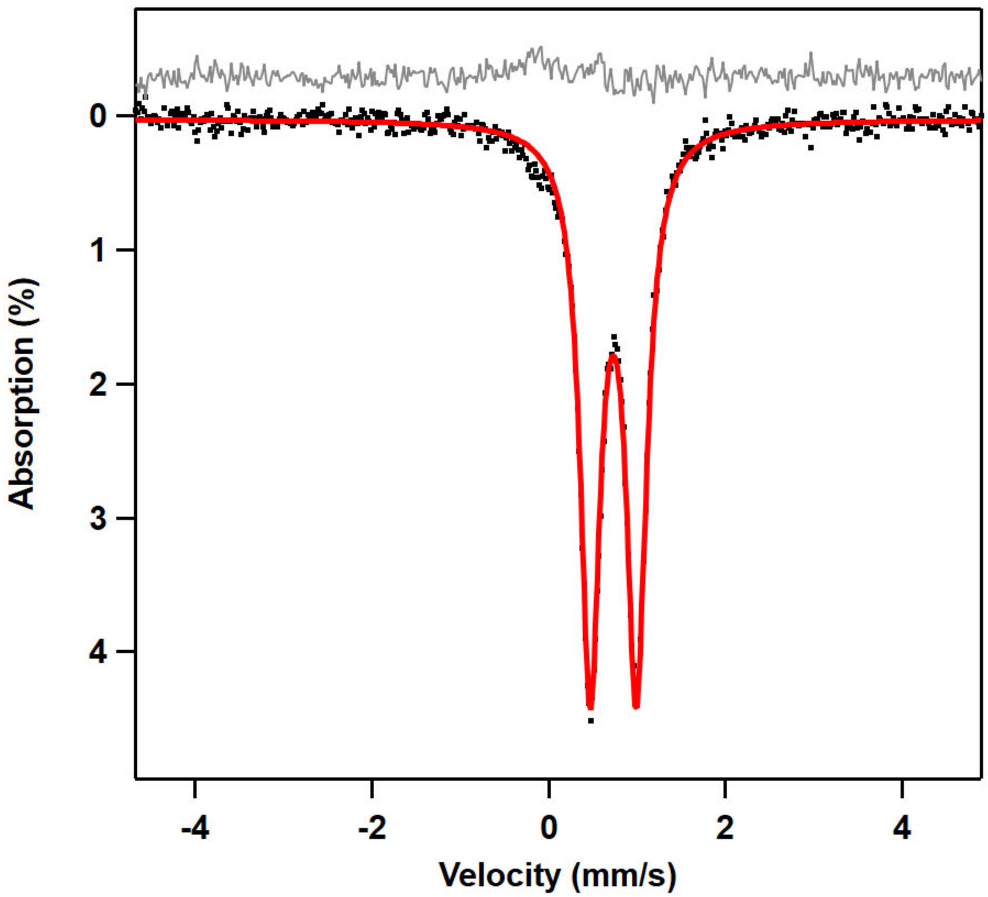

Figure S24. Zero-field ^{57}Fe Mössbauer spectrum of (^{Em}L)Fe(OSiEt$_3$) at 90 K. Isomer shift and quadrupole splitting are reported relative to α-iron foil at room temperature. The black dot, red line, and gray line represent the experimental data, fit, and residuals, respectively. δ (mm/s), $|\Delta E_Q|$ (mm/s), γ (mm/s) = 0.73, 0.52, 0.14.

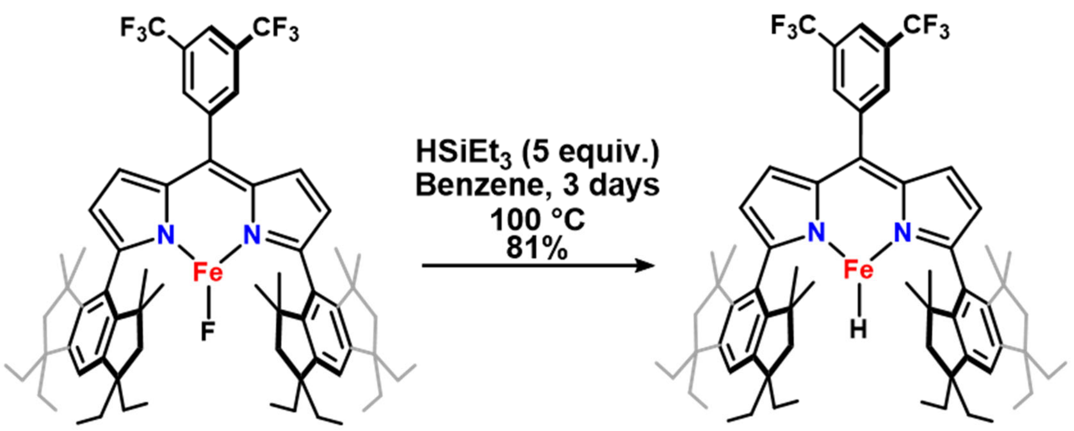

(^{Em}L)Fe(H) (8): All manipulations were performed with silanized glassware. In the drybox, a solution of (^{Em}L)Fe(F) (8.0 mg, 0.0074 mmol) and $HSiEt_3$ (4.3 mg, 0.037 mmol) in 0.7 mL C_6D_6 were transferred to J-Young NMR tube. The tube was sealed and heated to 100 °C for 3 days to generate (^{Em}L)Fe(H). ^{1}H NMR (500 MHz, 298 K, C_6D_6): δ 81.69 (2H), 75.35 (2H), 42.79 (2H), 22.58 (2H), 22.42 (12H), 20.14 (1H), 14.16 (4H), 11.03 (4H), 4.09 (8H), 1.51 (12H), –7.41 (12H), –11.08 (4H), –23.90 (4H), –112.97 (12H) ppm. ^{19}F NMR (471 MHz, 298 K, C_6D_6): δ –52.84 ppm. Zero-field ^{57}Fe Mossbauer (90 K) (δ, $|\Delta E_Q|$ (mm/s)): 0.47, 1.05 (γ = 0.19 mm/s) HRMS (ESI$^-$): m/z Calc. 1105.5716 $[C_{65}H_{82}F_6FeN_2+HCOO]^-$, Found 1105.5727 $[M+HCO_2]^-$.

Note: The *in-situ* generated (^{Em}L)Fe(H) is thermally stable in solution state upto 105 °C. However, work-up of the crude mixture of (^{Em}L)Fe(H) in a drybox led to slow hydrolysis into (^{Em}L)Fe(OH) due to the adventitious water.

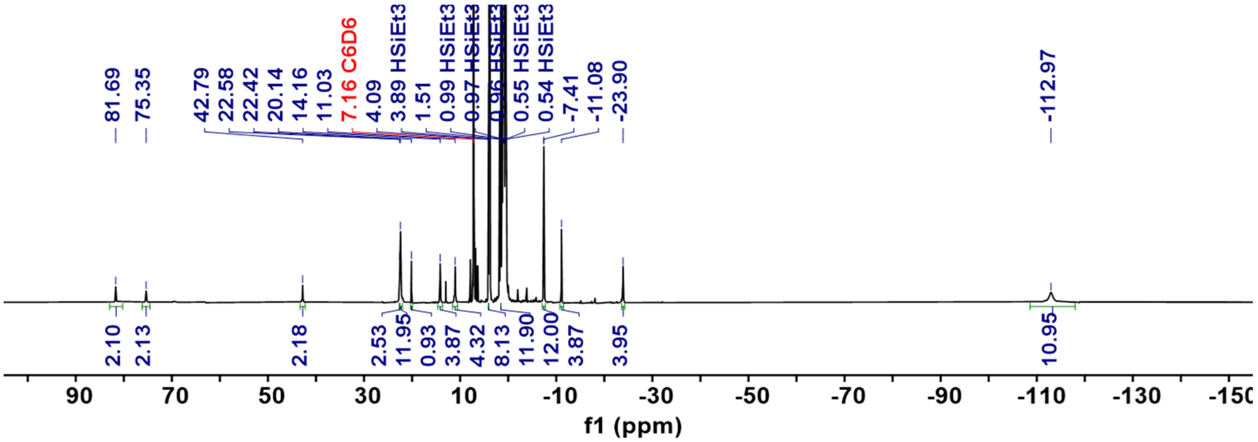

Figure S25. ^{1}H NMR spectrum of *in-situ* generated (^{Em}L)Fe(H) from (^{Em}L)Fe(F) and 5 equivalent of $HSiEt_3$, (500 MHz, 298 K, C_6D_6).

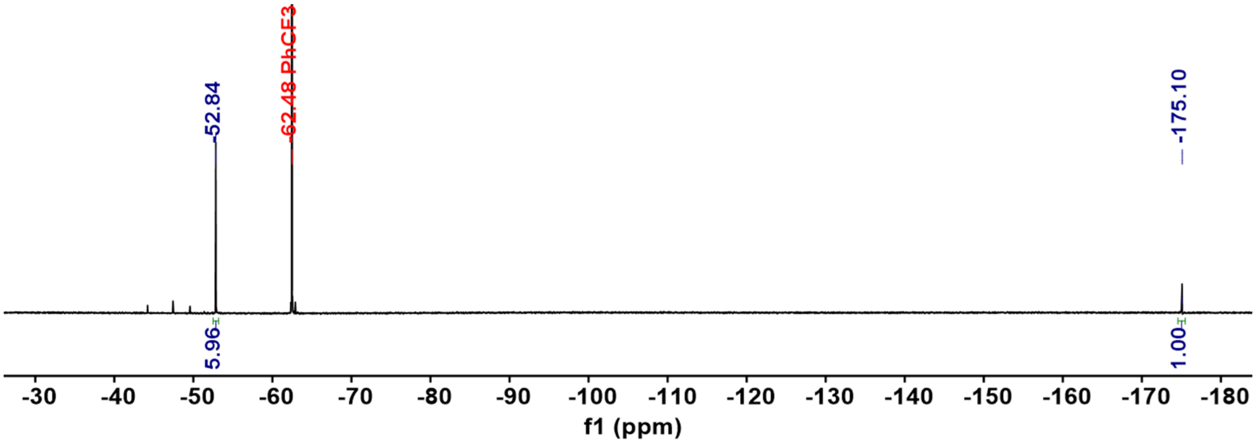

Figure S26. ^{19}F NMR spectrum of *in-situ* generated (^{Em}L)Fe(H) from (^{Em}L)Fe(F) and 5 equivalent of $HSiEt_3$, (471 MHz, 298 K, C_6D_6). 1 equivalent of *F*–$Si(CH_2CH_3)_3$ (–175.10 ppm, 1F) was produced as a byproduct. $PhCF_3$ was used as an internal standard to quantify spectroscopic yield.

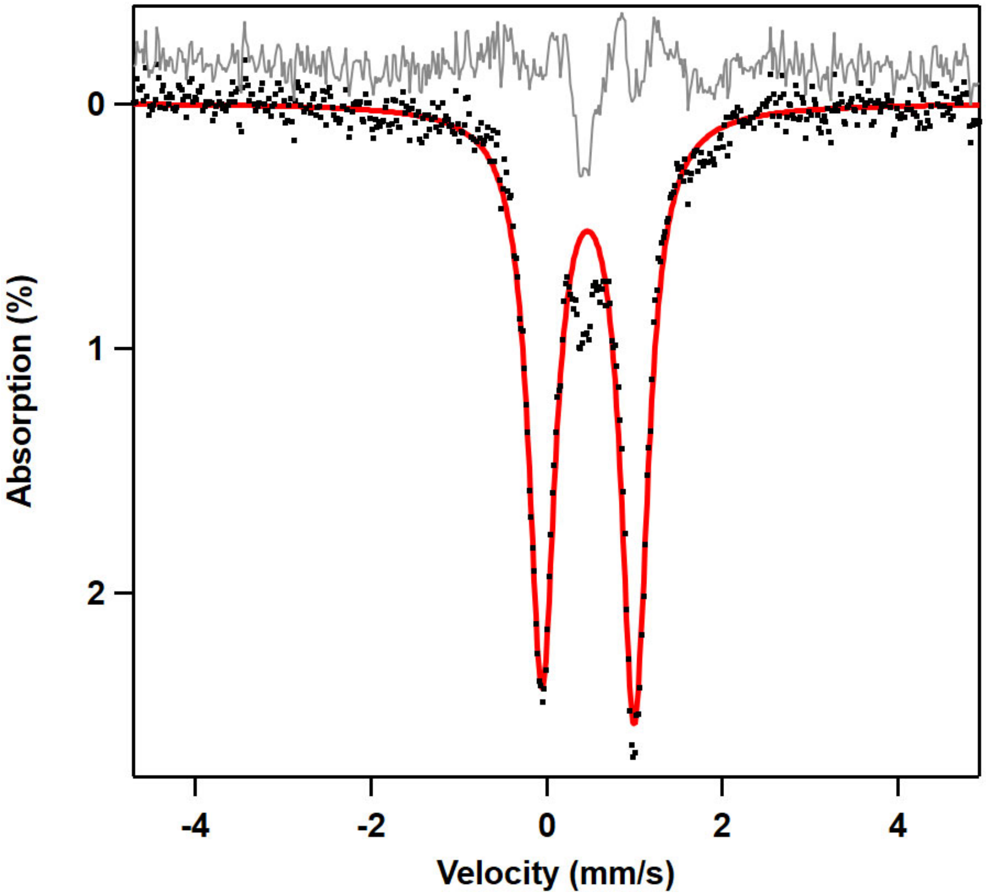

Figure S27. Zero-field ^{57}Fe Mössbauer spectrum of *in-situ* generated (^{Em}L)Fe(H) at 90 K. Isomer shift and quadrupole splitting are reported relative to α-iron foil at room temperature. The black dot, red line, and gray line represent the experimental data, fit, and residuals, respectively. δ (mm/s), $|\Delta E_Q|$ (mm/s), γ (mm/s) = 0.47, 1.05, 0.19

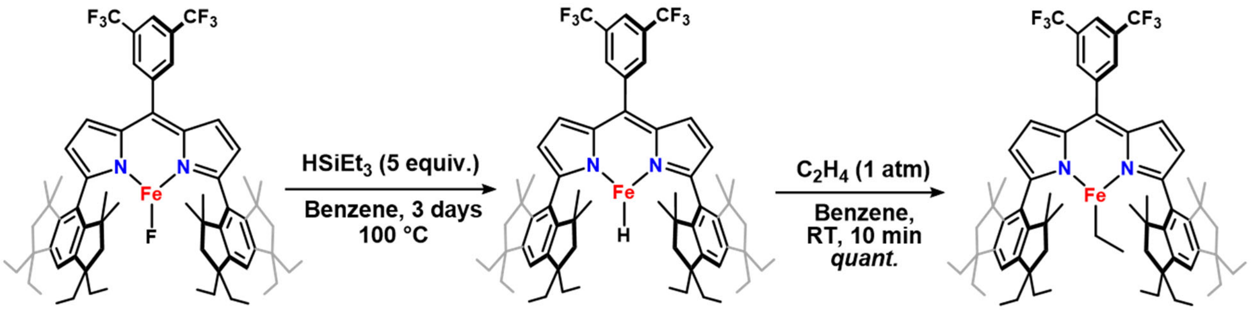

(^{Em}L)Fe(C_2H_5) (9): In the drybox, a solution of (^{Em}L)Fe(F) (25.0 mg, 0.0232 mmol) and $HSiEt_3$ (13.5 mg, 0.116 mmol) in 1 mL benzene was transferred to a J-Young NMR tube, and sealed. After heated to 100 °C for 3 days, the headspace was evacuated, followed by introduction of ethylene gas (1 atm, 3.0 mL, 0.12 mmol) into the headspace at room temperature. Then, the resulting mixture in the J-Young NMR tube was vigorously mixed at room temperature. The resulting solution was degassed, lyophilized, and filtered through Celite in hexanes. The product was obtained as red crystals by recrystallization from a concentrated solution of **9** in *n*-pentane at –35 °C (9.2 mg, 0.0084 mmol, 36%). ^{1}H NMR (500 MHz, 298 K, C_6D_6): δ 86.66 (2H), 57.08 (2H), 55.30 (2H), 23.69 (1H), 17.01 (12H), 11.18 (4H), 9.64 (2H), 9.34 (4H), –1.60 (4H), –1.98 (4H), –2.35 (12H), –15.03 (4H), –16.71 (12H), –21.23 (4H), –117.10 (12H) ppm. ^{19}F NMR (471 MHz, 298 K, C_6D_6): δ –49.48 ppm. Zero-field ^{57}Fe Mossbauer (90 K) (δ, $|\Delta E_Q|$ (mm/s)): 0.43, 1.04 (γ = 0.17 mm/s). HRMS (ESI$^-$): m/z Calc. 1123.5741 [$C_{67}H_{86}F_6FeN_2$+Cl]$^-$, Found 1123.5761 [M+Cl]$^-$.

Note. The conversion of *in-situ* generated (^{Em}L)Fe(H) to (^{Em}L)Fe(Et) is quantitative as determined by ^{19}F NMR using $PhCF_3$ as an internal standard.

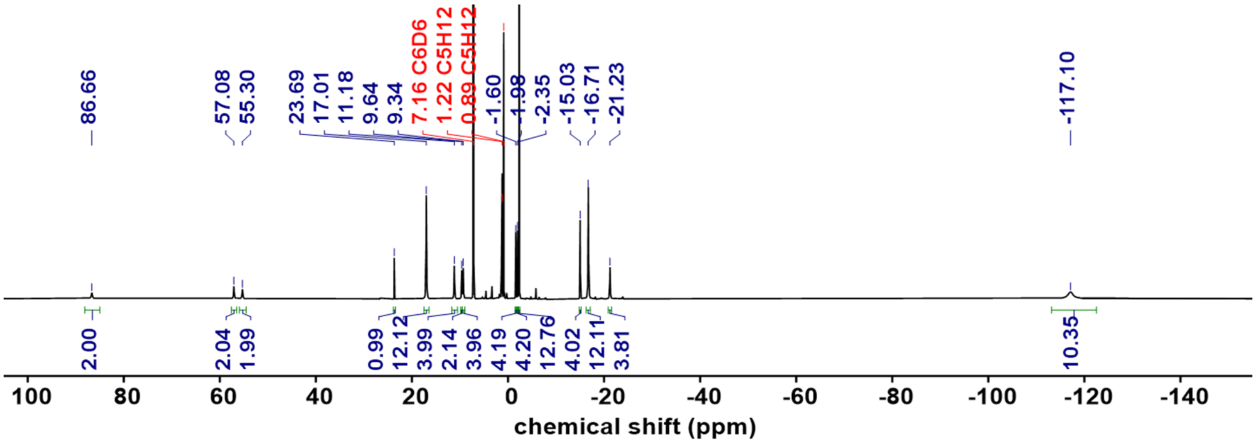

Figure S28. ^{1}H NMR spectrum of (^{Em}L)Fe(C_2H_5), (500 MHz, 298 K, C_6D_6).

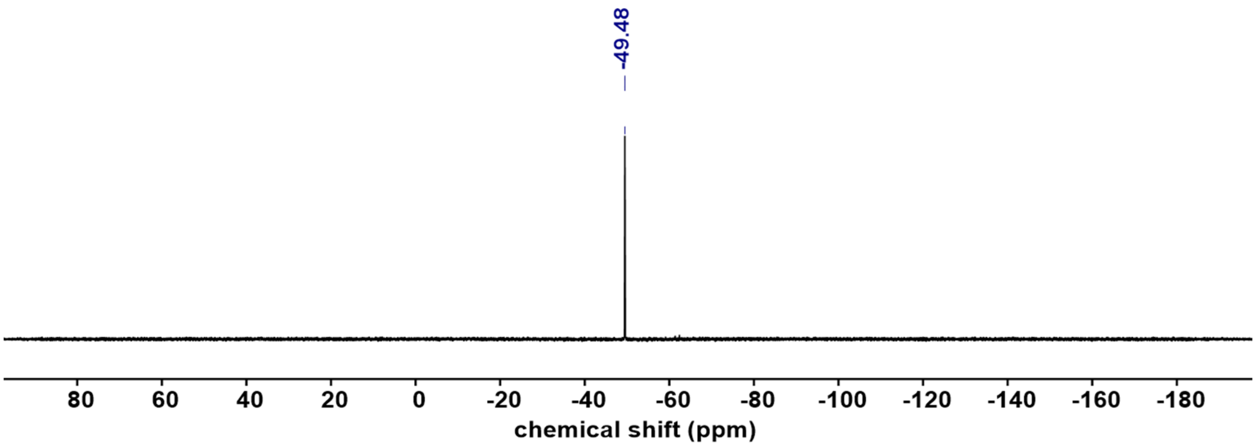

Figure S29. ^{19}F NMR spectrum of (^{Em}L)Fe(C_2H_5), (471 MHz, 298 K, C_6D_6).

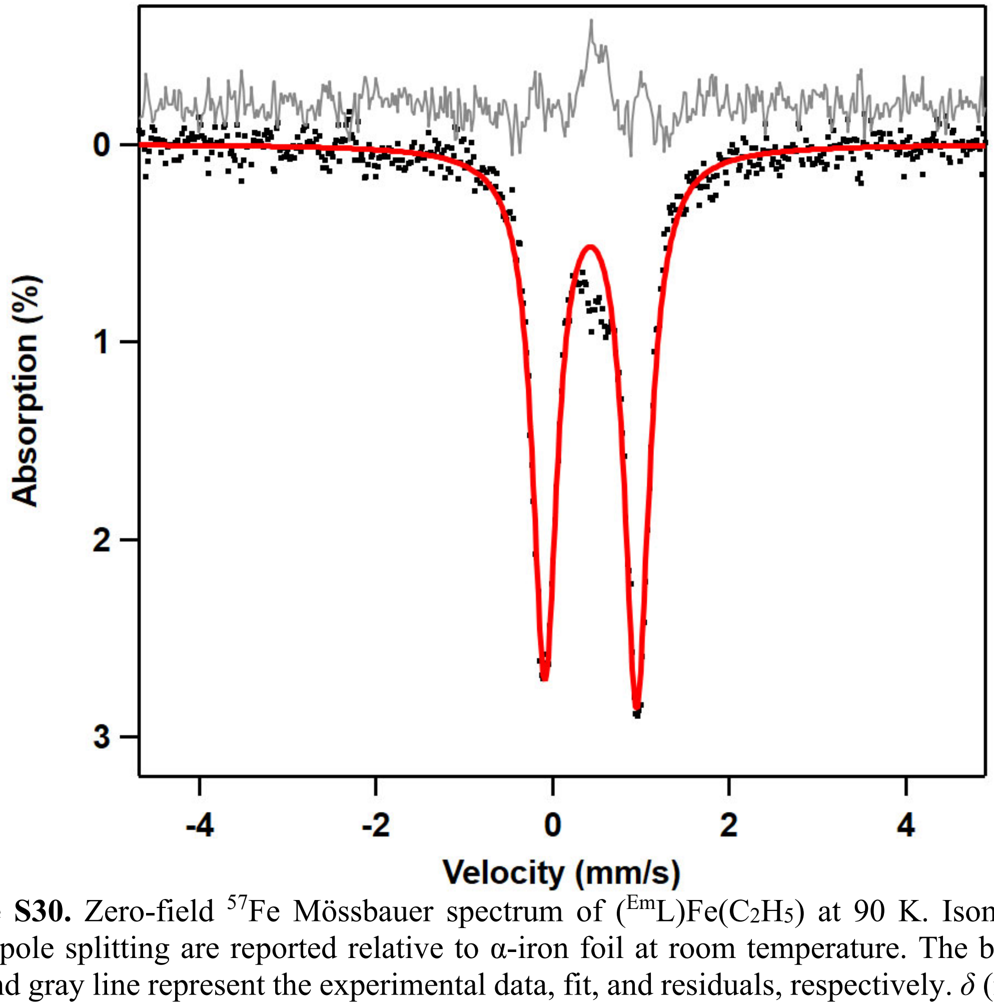

Figure S30. Zero-field ^{57}Fe Mössbauer spectrum of (^{Em}L)Fe(C_2H_5) at 90 K. Isomer shift and quadrupole splitting are reported relative to α-iron foil at room temperature. The black dot, red line, and gray line represent the experimental data, fit, and residuals, respectively. δ (mm/s), $|\Delta E_Q|$ (mm/s), γ (mm/s) = 0.43, 1.04, 0.17

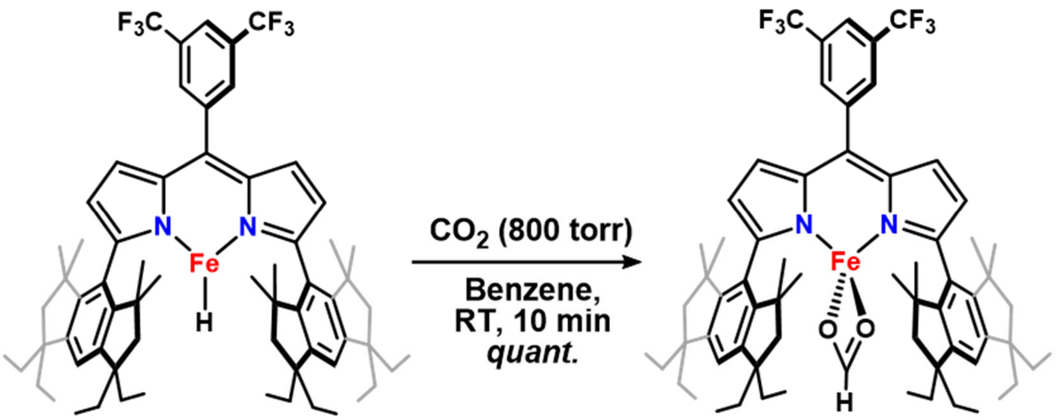

(^{Em}L)Fe(κ^2O,O-O_2CH) (10): To an *in-situ* generated (^{Em}L)Fe(H) in 0.7 mL C_6D_6 in a JYoung NMR tube from (^{Em}L)Fe(F) (8.0 mg, 0.0074 mmol) and $HSiEt_3$ (4.3 mg, 0.37 mmol), 800 torr of CO_2 gas was introduced into the headspace at room temperature following degassed by three freeze-pump-thaw cycles. After 10 minutes at room temperature, full conversion of (^{Em}L)Fe(H) to (^{Em}L)Fe(κ^2O,O-O_2CH) were observed by ^{19}F and ^{1}H NMR spectroscopy. The conversion was quantitative determined by ^{19}F NMR spectrum collected with $PhCF_3$ as an internal standard.

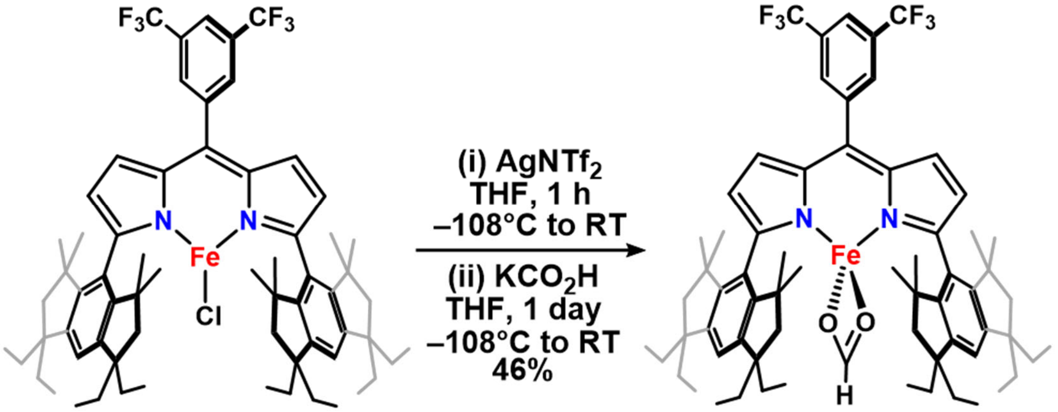

(^{Em}L)Fe(κ^2O,O-O_2CH) (10): To a thawing solution of (^{Em}L)Fe(Cl) (22.8 mg, 0.0208 mmol) in 1 mL tetrahydrofuran, was added a solution of $AgNTf_2$ (8.8 mg, 0.023 mmol). The solution was warmed to room temperature and stirred for an hour. The resulting mixture was filtered through Celite in tetrahydrofuran. Then, a suspension of potassium formate (4.4 mg, 0.052 mmol) was added to the filtrate. The resulting mixture was stirred at room temperature for 2 days. After filtered through Celite in tetrahydrofuran, the volatiles were removed *in vacuo*. The resulting solids were dissolved in hexanes, and filtered through Celite in hexanes. The product was obtained after recrystallization from a concentrated solution of **10** in *n*-pentane at –35 °C (10.6 mg, 0.00959 mmol, 46%). ^{1}H NMR (500 MHz, 298 K, C_6D_6): δ 128.89 (1H, FeO_2C–*H*), 89.27 (2H), 74.23 (2H), 59.38 (2H), 31.30 (12H), 29.29 (1H), 21.69 (4H), 18.06 (4H), 16.76 (4H), –4.10 (4H), –4.45 (12H), –4.64 (4H), –22.75 (4H), –28.90 (12H), –29.11 (4H), –135.56 (12H) ppm. ^{19}F NMR (471 MHz, 298 K, C_6D_6): δ –44.37 ppm. Zero-field ^{57}Fe Mossbauer (90 K) (δ, $|\Delta E_Q|$ (mm/s)): 0.95, 1.38 (γ = 0.27 mm/s). HRMS (ESI$^-$): m/z Calc. 1139.5323 [$C_{66}H_{82}F_6FeN_2O_2$+Cl]$^-$, Found 1139.5335 [M+Cl]$^-$.

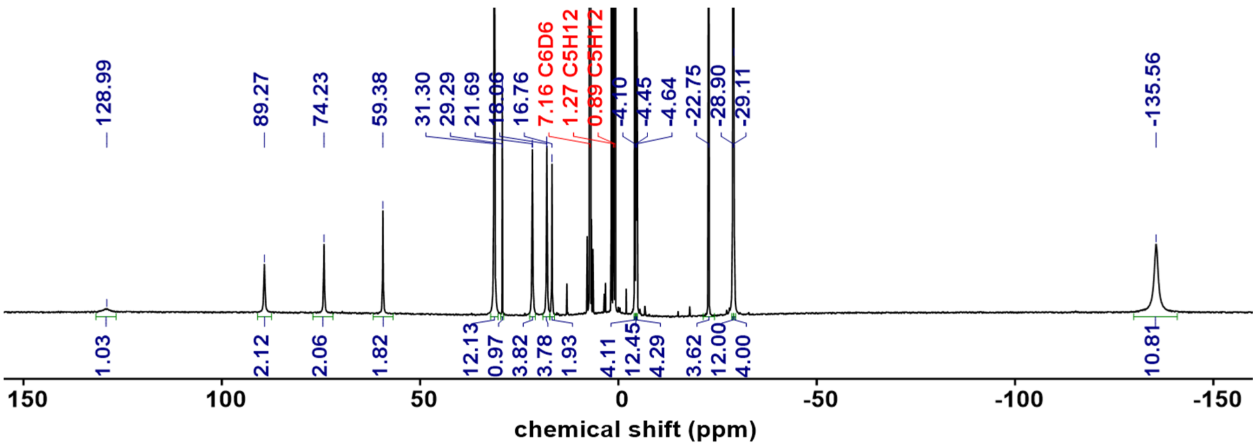

Figure S31. ^{1}H NMR spectrum of (^{Em}L)Fe(κ^2O,O-O_2CH), (500 MHz, 298 K, C_6D_6).

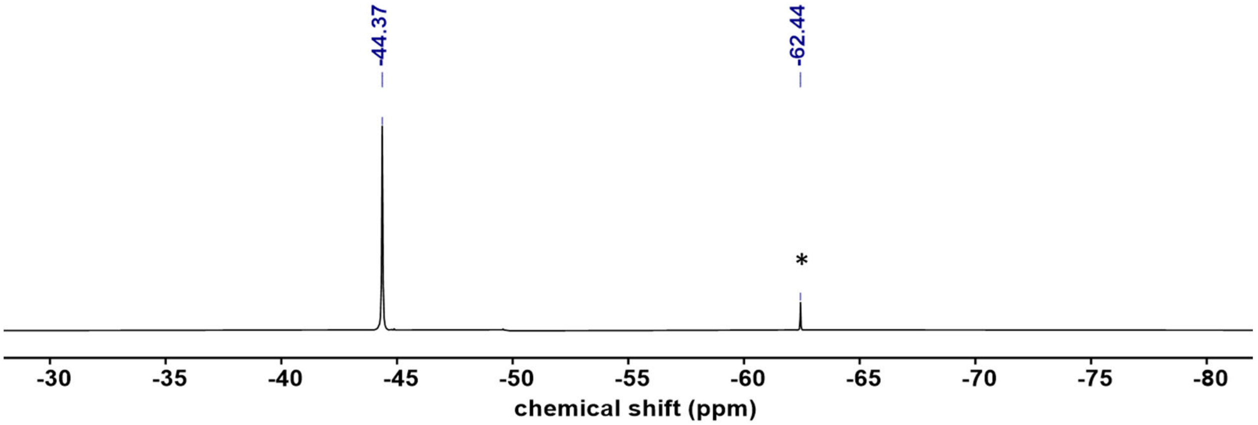

Figure S32. ^{19}F NMR spectrum of (^{Em}L)Fe(κ^2O,O-O_2CH), (471 MHz, 298 K, C_6D_6). Notably, 5% of (^{Em}L)H (*) was generated as an impurity, which cannot be separated from **12** through recrystallization.

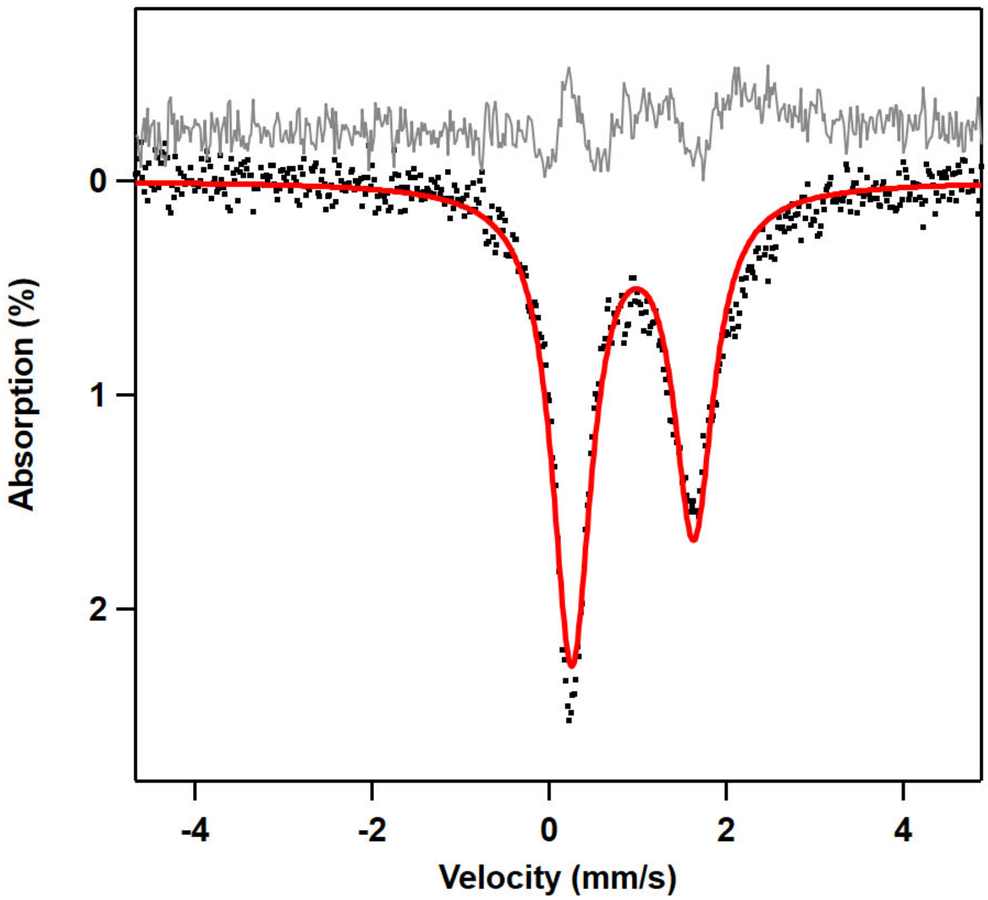

Figure S33. Zero-field ^{57}Fe Mössbauer spectrum of (^{Em}L)Fe(κ^2O,O-O_2CH) at 90 K. Isomer shift and quadrupole splitting are reported relative to α-iron foil at room temperature. The black dot, red line, and gray line represent the experimental data, fit, and residuals, respectively. δ (mm/s), $|\Delta E_Q|$ (mm/s), γ (mm/s) = 0.95, 1.38, 0.27

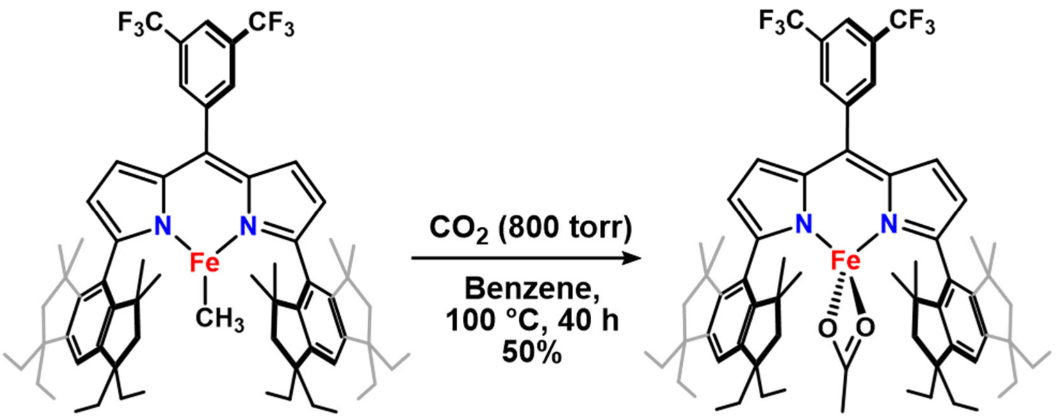

(^{Em}L)Fe(κ^2O,O-O_2CCH_3) (12): In the drybox, a solution of (^{Em}L)Fe(CH_3) (17.2 mg, 0.0160 mmol) in 3 mL of benzene was transferred to a 50 mL Schlenk flask. The solution was degassed by three freeze-pump-thaw cycles. Then, CO_2 (800 torr, 2.10 mmol) was introduced into the headspace at room temperature. The resulting solution was heated to 100 °C. After stirring for 40 hours, the solution was cooled to room temperature, degassed by three freeze-pump-thaw cycles, and lyophilized. The resulting powder was dissolved in hexanes, filtered through Celite in hexanes, and the volatiles were removed *in vacuo*. The product was obtained as red crystals by recrystallization from a concentrated solution of **12** in *n*-pentane at –35 °C (9.0 mg, 0.0080 mmol, 50%). A new broad ^{1}H NMR peak features at +303.55 ppm, which was not observed from other (^{Em}L)Fe(X) complexes. Thus, we assigned this peak as methyl proton of acetate ligand. ^{1}H NMR (500 MHz, 298 K, C_6D_6): δ 303.55 (3H, FeO_2CCH_3), 87.29 (2H), 73.09 (2H), 56.92 (2H), 28.90 (1H), 20.36 (4H), 18.21 (12H), 16.95 (4H), 12.86 (2H), –5.19 (4H), –5.41 (12H), –6.21 (4H), –22.30 (4H), –27.99 (12H), –131.97 (12H) ppm. ^{19}F NMR (471 MHz, 298 K, C_6D_6): δ –44.54 ppm. Zero-field ^{57}Fe Mossbauer (90 K) (δ, $|\Delta E_Q|$ (mm/s)): 0.93, 1.07 (γ = 0.20 mm/s). HRMS (ESI$^-$): m/z Calc. 1163.5770 [$C_{67}H_{84}F_6FeN_2O_2$+HCOO]$^-$, Found 1163.5751 [M+HCO_2]$^-$.

Note. The (^{Em}L)Fe(CH_3) is stable under CO_2 atmosphere in benzene solution at 60 °C.

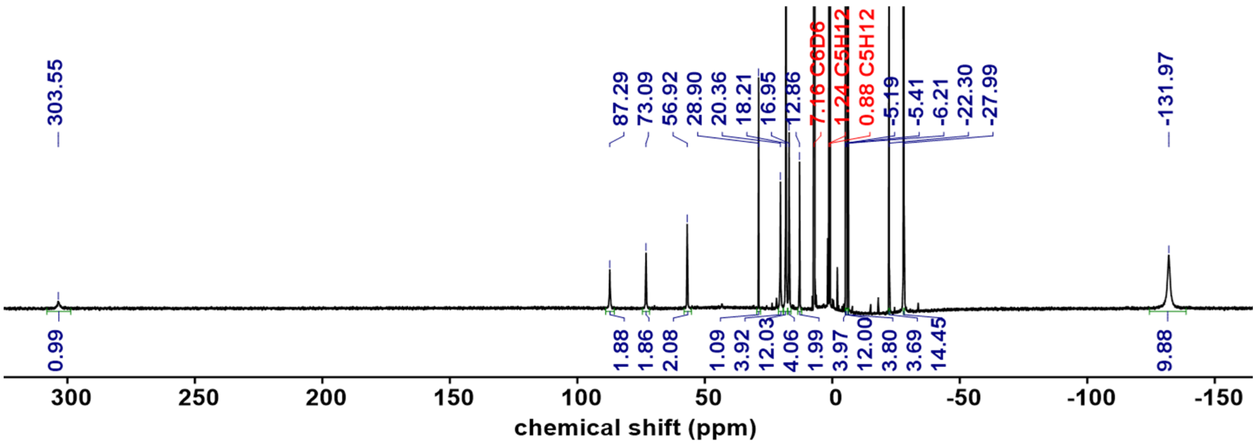

Figure S34. ^{1}H NMR spectrum of (^{Em}L)Fe(κ^2O,O-O_2CCH_3), (500 MHz, 298 K, C_6D_6).

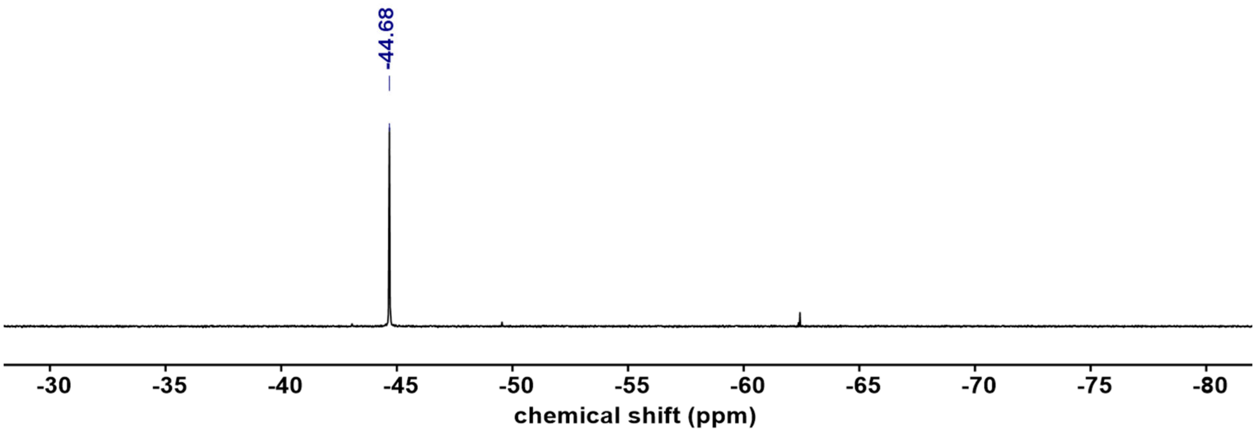

Figure S35. ^{19}F NMR spectrum of (^{Em}L)Fe(κ^2O,O-O_2CCH_3), (471 MHz, 298 K, C_6D_6).

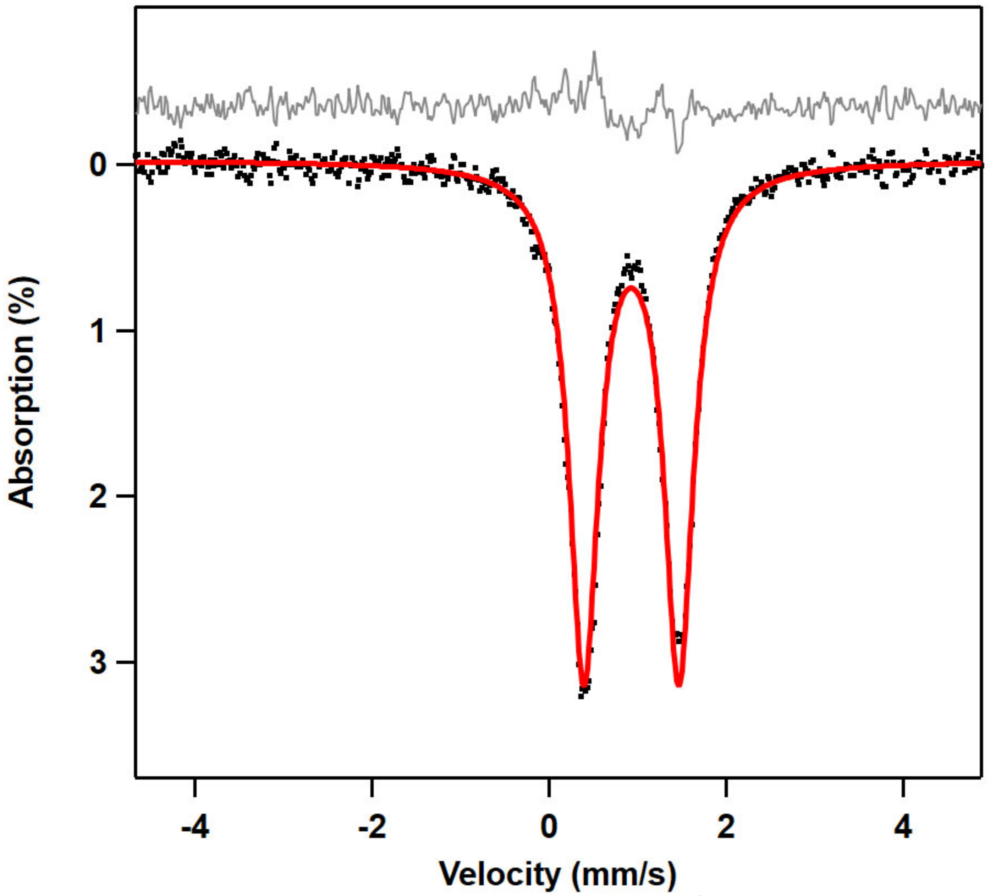

Figure S36. Zero-field ^{57}Fe Mössbauer spectrum of (^{Em}L)Fe(κ^2O,O-O_2CCH_3) at 90 K. Isomer shift and quadrupole splitting are reported relative to α-iron foil at room temperature. The black dot, red line, and gray line represent the experimental data, fit, and residuals, respectively. δ (mm/s), $|\Delta E_Q|$ (mm/s), γ (mm/s) = 0.93, 1.07, 0.20.

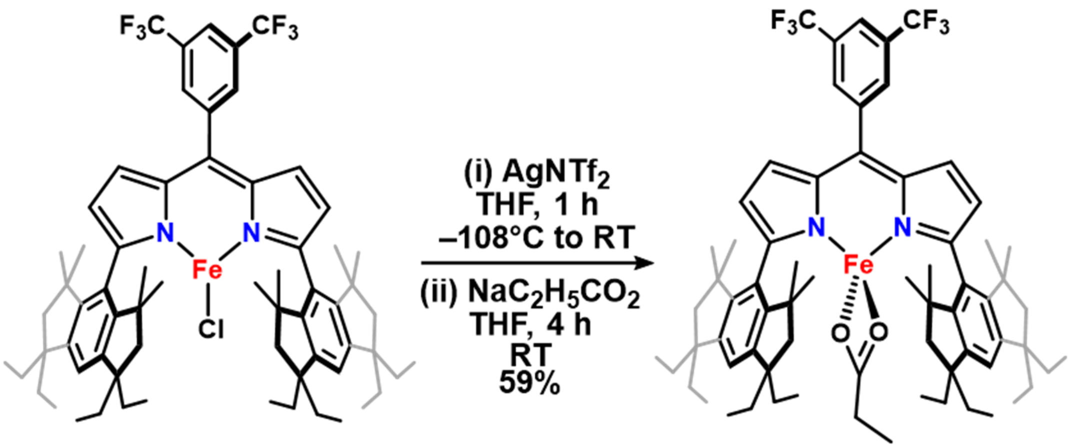

(^{Em}L)Fe(κ^2O,O-$O_2CCH_2CH_3$) (13): To a thawing solution of (^{Em}L)Fe(Cl) (37.4 mg, 0.0341 mmol) in 2 mL of tetrahydrofuran, was added a tetrahydrofuran solution of $AgNTf_2$ (14.3 mg, 0.0369 mmol). The solution was warmed to room temperature and stirred for an hour. The resulting mixture was filtered through Celite in tetrahydrofuran. Then, to the filtrate, was added a suspension of sodium propionate (4.0 mg, 0.042 mmol). The resulting mixture was stirred at room temperature for 4 hours. After filtered through Celite in tetrahydrofuran, the volatiles were removed *in vacuo*. The resulting solids were dissolved in hexanes and filtered through Celite in hexanes. The product was obtained after recrystallization from a concentrated solution of **13** in *n*-pentane at –35 °C (22.9 mg, 0.0202 mmol, 59%). By comparison ^{1}H NMR spectrum with carboxylate complexes ((^{Em}L)Fe(O_2CH) and (^{Em}L)Fe(O_2CCH_3)), we assigned a peak at +288.03 ppm and +95.06 ppm to $O_2CCH_2CH_3$, and $O_2CCH_2CH_3$, respectively. ^{1}H NMR (500 MHz, 298 K, C_6D_6): δ 288.03 (2H, $FeO_2CC\mathit{H}_2CH_3$), 95.06 (3H, $FeO_2CCH_2C\mathit{H}_3$), 84.12 (2H), 69.96 (2H), 56.28 (2H), 27.90 (1H), 17.09 (4H), 14.20 (4H), 13.15 (12H), 10.17 (2H), –5.36 (4H), –5.60 (12H), –6.77 (4H), –20.89 (4H), –25.64 (4H), –25.93 (12H), –127.80 (12H) ppm. ^{19}F NMR (471 MHz, 298 K, C_6D_6): δ –45.51 ppm. Zero-field ^{57}Fe Mossbauer (90 K) (δ, $|\Delta E_Q|$ (mm/s)): 0.84, 1.10 (γ = 0.19 mm/s). HRMS (ESI$^-$): m/z Calc. 1167.5639 $[C_{68}H_{86}F_6FeN_2O_2+Cl]^-$, Found 1167.5621 $[M+Cl]^-$.

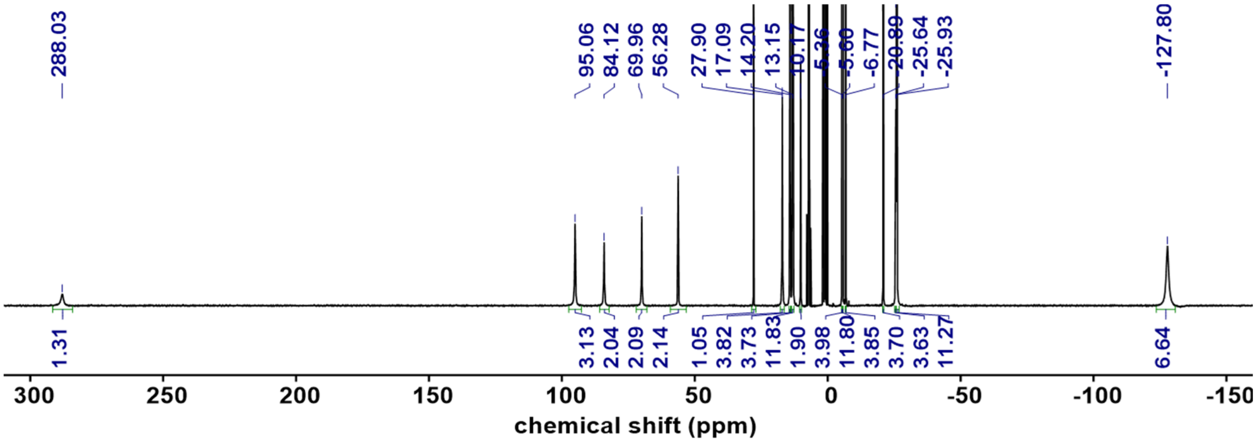

Figure S37. ^{1}H NMR spectrum of (^{Em}L)Fe(κ^2O,O-$O_2CCH_2CH_3$), (500 MHz, 298 K, C_6D_6).

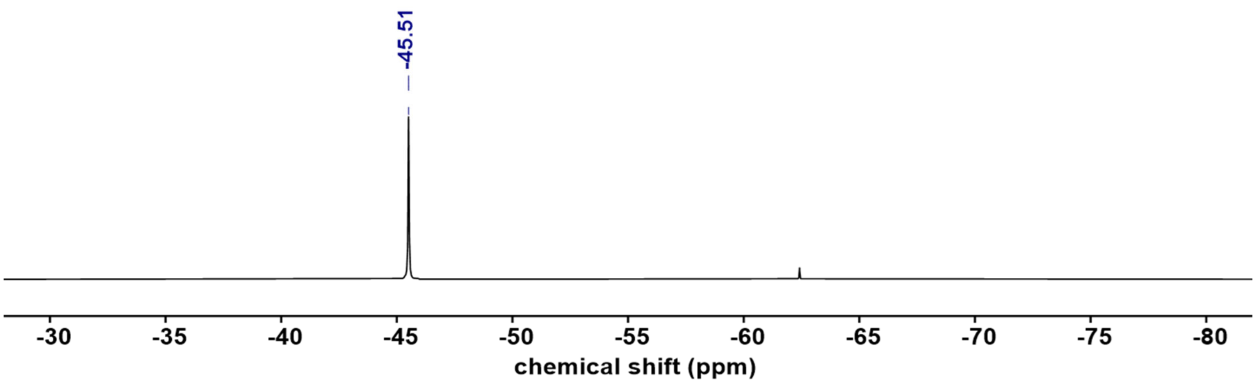

Figure S38. ^{19}F NMR spectrum of (^{Em}L)Fe(κ^2O,O-$O_2CCH_2CH_3$), (471 MHz, 298 K, C_6D_6).

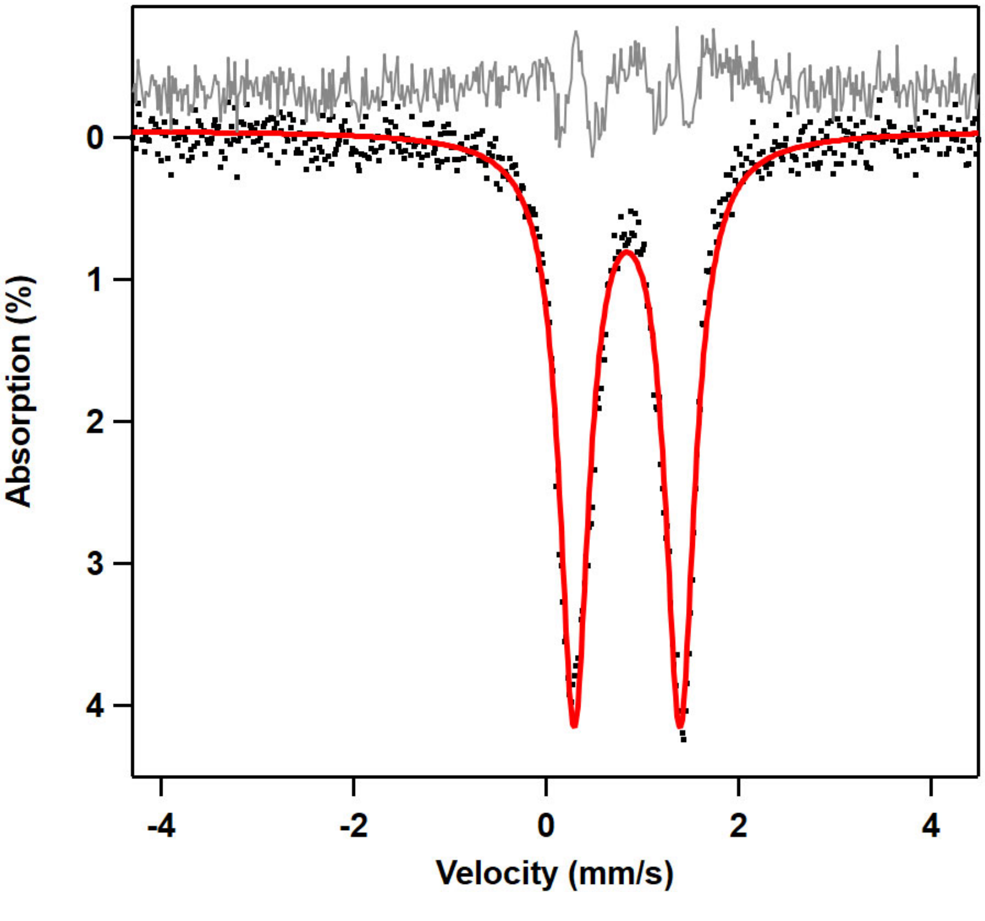

Figure S39. Zero-field ^{57}Fe Mössbauer spectrum of (^{Em}L)Fe(κ^2O,O-$O_2CCH_2CH_3$) at 90 K. Isomer shift and quadrupole splitting are reported relative to α-iron foil at room temperature. The black dot, red line, and gray line represent the experimental data, fit, and residuals, respectively. δ (mm/s), $|\Delta E_Q|$ (mm/s), γ (mm/s) = 0.84, 1.10, 0.19.

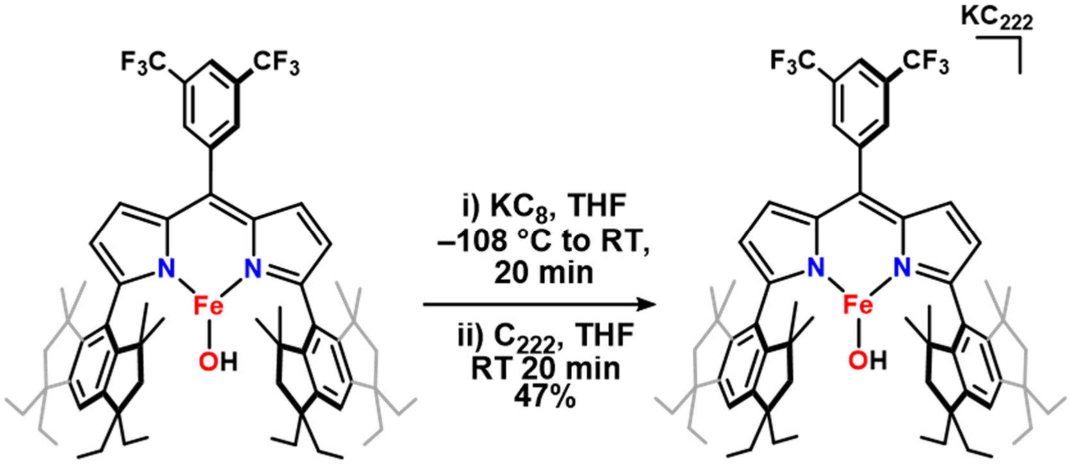

[KC_{222}][(^{Em}L)Fe(OH)] (14):
Route A: To a thawing solution of (^{Em}L)Fe(OH) (35.2 mg, 0.0327 mmol) in 1 mL tetrahydrofuran, a suspension of KC_8 (4.6 mg, 0.0340 mmol) in 2 mL tetrahydrofuran was added. The resulting mixture was warmed to room temperature and stirred for 20 minutes. The color changed from red to purple. The resulting mixture was filtered through Celite in tetrahydrofuran. To the filtrate, was added a solution of [2.2.2]Cryptand (13.8 mg, 0.0367 mmol) at room temperature. The resulting solution was stirred at room temperature for 5 minutes, and the volatiles were removed *in vacuo*. The resulting powder were washed with hexanes, dissolved in diethyl ether, and filtered through Celite in diethyl ether. The product was obtained as purple crystal after recrystallized from a concentrated solution of **14** in diethyl ether at –35 °C (23.0 mg, 0.0154 mmol, 47%).

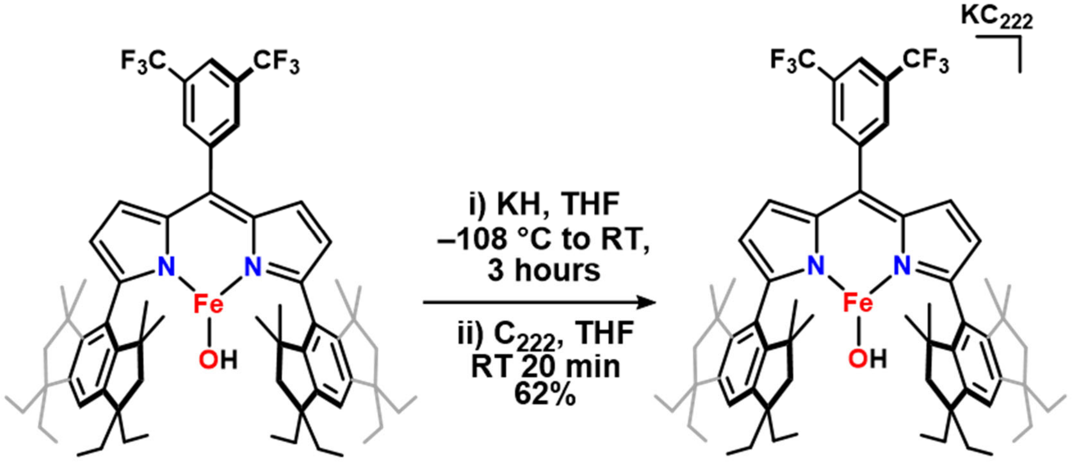

Route B: To a thawing solution of (^{Em}L)Fe(OH) (53.5 mg, 0.0497 mmol) in 2 mL tetrahydrofuran, a suspension of potassium hydride (6.0 mg, 0.150 mmol) in 2 mL tetrahydrofuran was added. The resulting mixture was warmed to room temperature and stirred for 3 hours. The color changed from red to purple slowly. The resulting mixture was filtered through Celite in tetrahydrofuran. To the filtrate, was added a solution of [2.2.2] Cryptand (20.2 mg, 0.0537 mmol) at room temperature. The resulting solution was stirred at room temperature for 5 minutes, and the volatiles were removed *in vacuo*. The resulting powder were washed with hexanes, dissolved in diethyl ether, and filtered through Celite in diethyl ether. The product was obtained as purple crystal after recrystallized from a concentrated solution of **14** in diethyl ether at –35 °C (45.8 mg, 0.0307 mmol, 62%). ^{1}H NMR (500 MHz, 298 K, C_6D_6): δ 115.95 (2H), 63.26 (2H), 34.87 (2H), 30.49 (12H),

21.98 (1H), 16.26 (4H), 14.64 (2H), 14.01 (4H), 5.16 (12H), 4.69 (12H), 2.96 (12H), –0.12 (4H), –0.91 (4H), –1.91 (12H), –13.19 (4H), –17.61 (4H), –18.10 (12H), –89.65 (12H) ppm. ^{19}F NMR (471 MHz, 298 K, C_6D_6): δ –49.04 ppm. FTIR (ATR): υ(O–H) = 3637 cm^{-1}. Zero-field ^{57}Fe Mossbauer (90 K) (δ, $|\Delta E_Q|$ (mm/s)): 0.59, 0.68 (γ = 0.16 mm/s). HRMS (ESI$^-$): m/z Calc. 1121.5664 [$C_{65}H_{82}F_6FeN_2O$+HCOO]$^-$, Found 1121.5656 [M+HCO_2]$^-$. HRMS (ESI$^+$): m/z Calc. 415.2205 [$C_{18}H_{36}KN_2O_6$]$^+$, Found 415.2221 [M]$^+$.

Note: We could not get a good fitting for zero-field ^{57}Fe Mössbauer spectrum collected at 90 K. The spectra were obtained reproducibly with different conditions (frozen C_6D_6 solution and crystalline powder). To check any NMR silent iron impurity in the recrystallized material, we oxidized the recrystallized [(^{Em}L)Fe(OH)][KC_{222}] with 1 equivalent of [Fc][$BArF_{24}$] in thawing THF solution, and collected the zero-field ^{57}Fe Mössbauer spectrum of the crude mixture in frozen THF solution (Figure S25). In the spectrum, only two species, (^{Em}L)Fe(OH) (45%) and ferrocene (55%), exist, which confirms the purity of [(^{Em}L)Fe(OH)][KC_{222}].

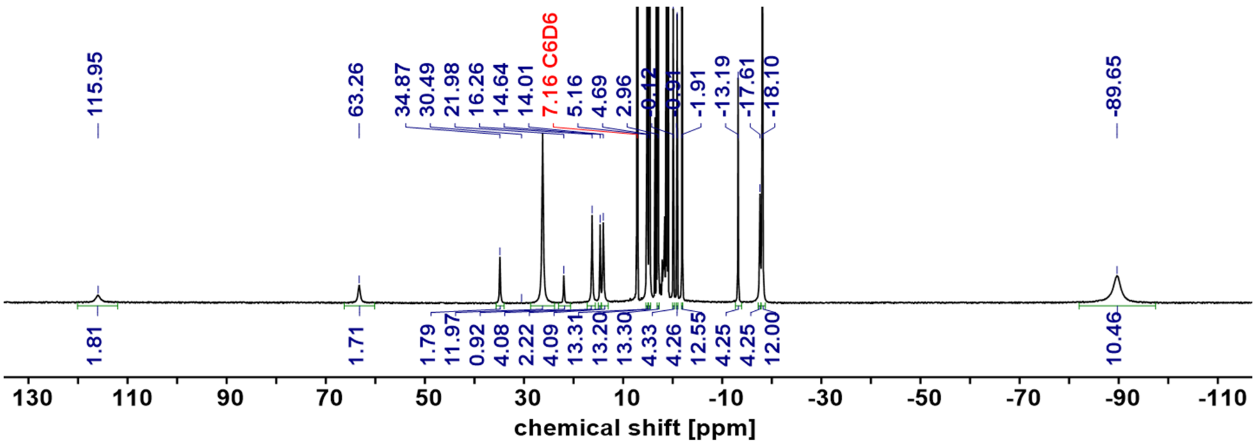

Figure S40. ^{1}H NMR spectrum of [(^{Em}L)Fe(OH)][KC_{222}], (500 MHz, 298 K, C_6D_6).

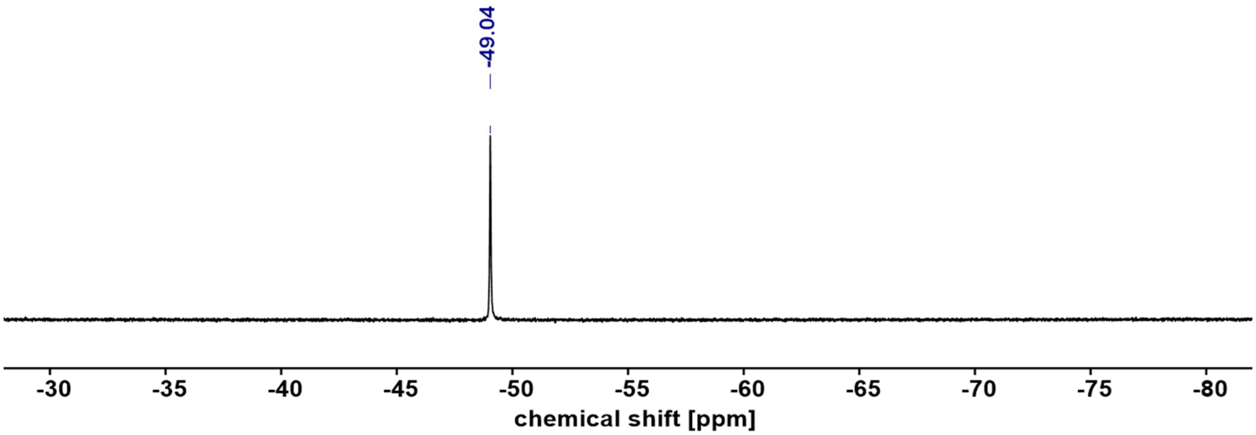

Figure S41. ^{19}F NMR spectrum of [(^{Em}L)Fe(OH)][KC_{222}], (471 MHz, 298 K, C_6D_6).

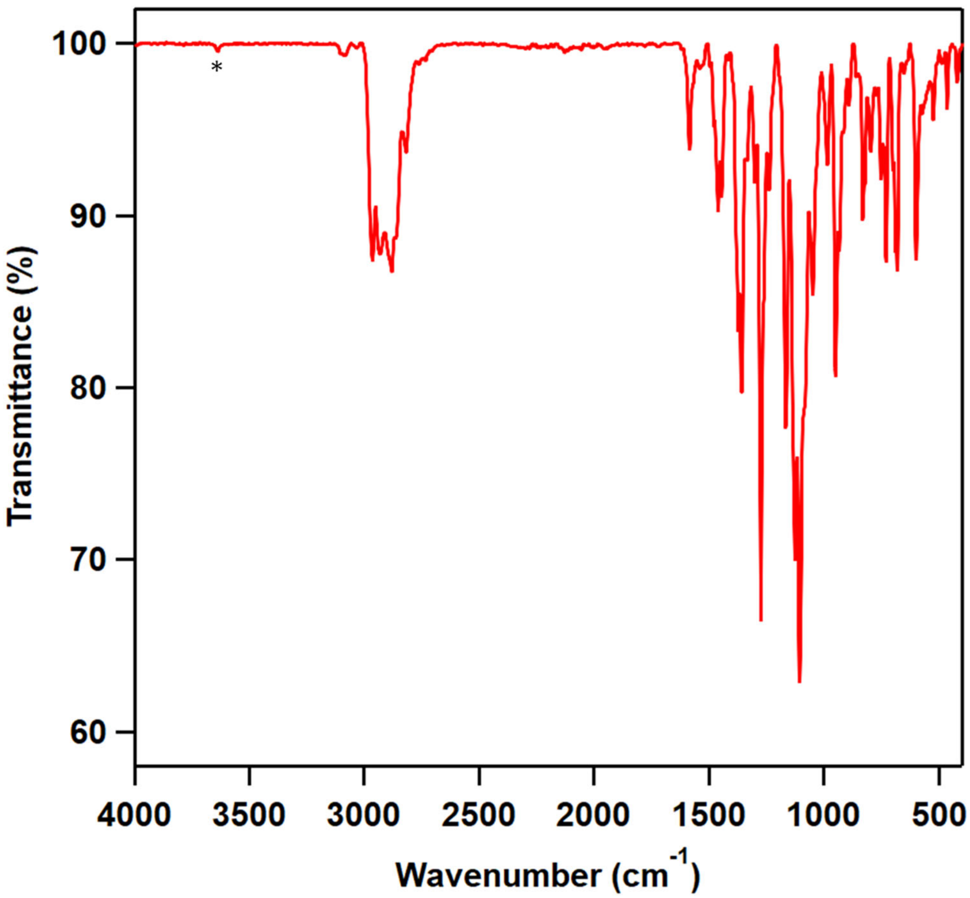

Figure S42. IR spectrum of [(^{Em}L)Fe(OH)][KC_{222}]. O–H vibration modes (υ(O–H) = 3637 cm^{-1}) are denoted with asterisks (*).

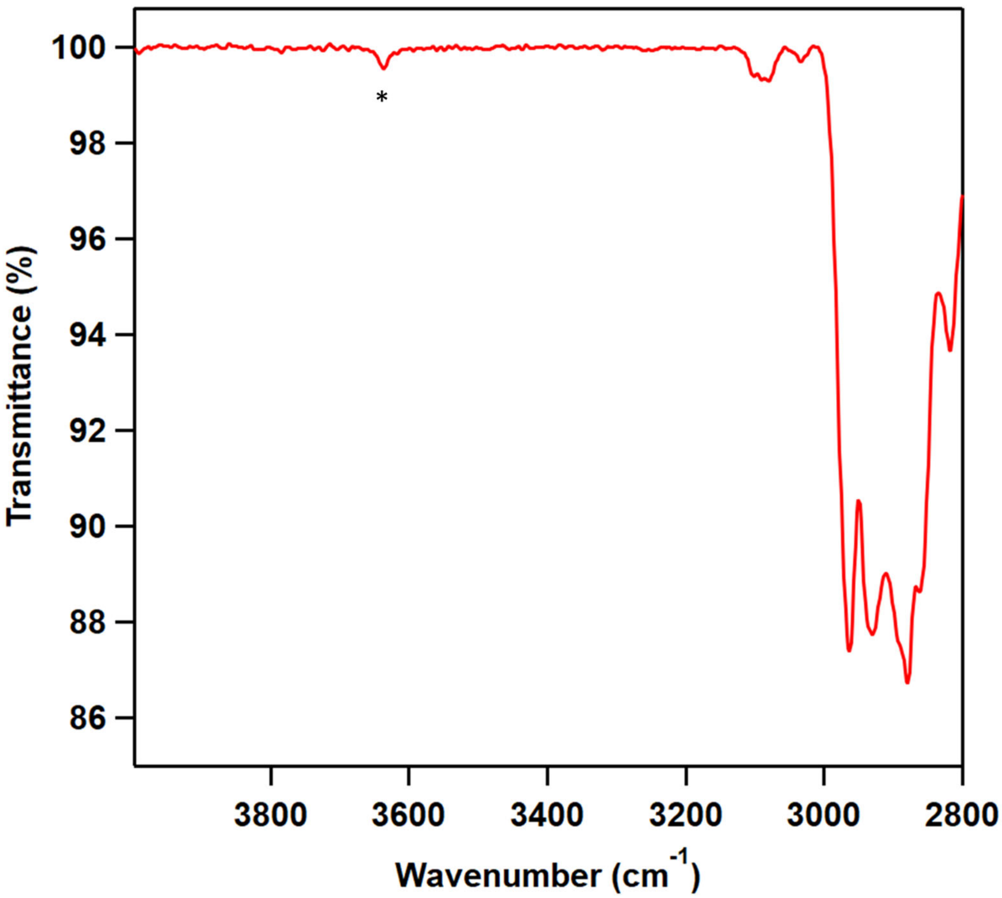

Figure S43. IR spectrum of [(^{Em}L)Fe(OH)][KC222] highlighting O–H vibration. O–H vibration modes (υ(O–H) = 3637 cm^{-1}) are denoted with asterisks (*).

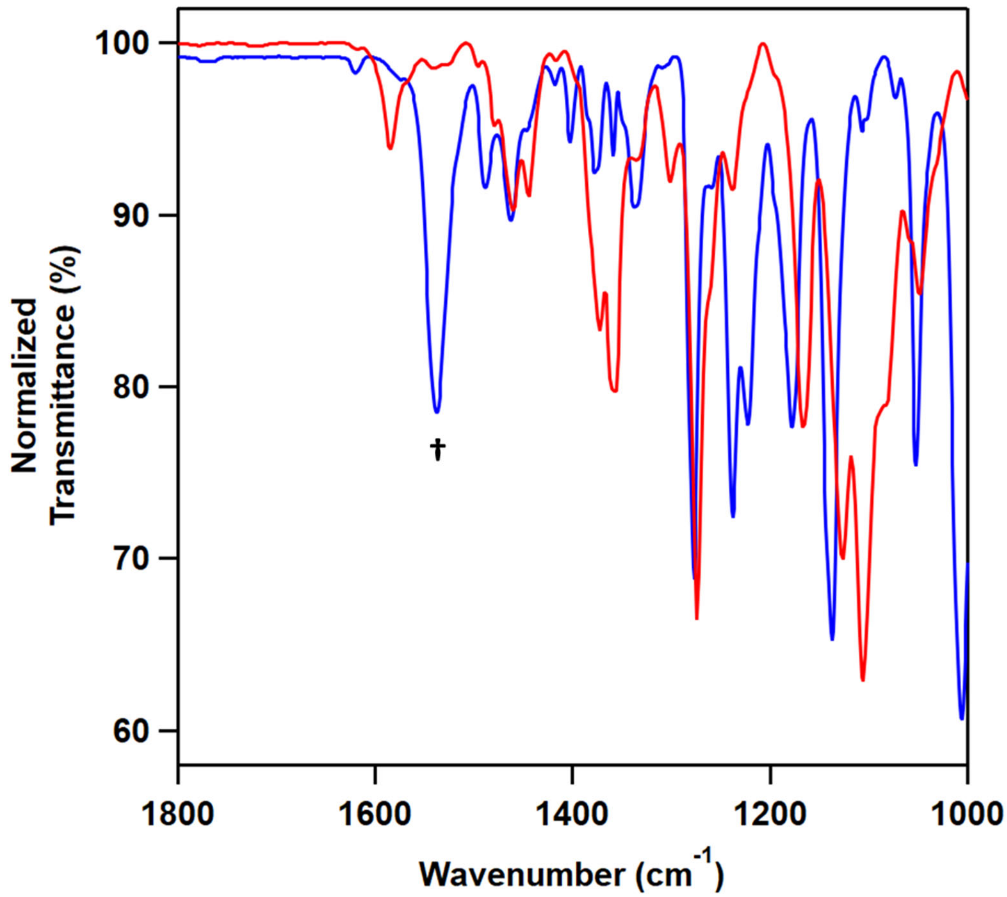

Figure S44. Stacked IR spectra of [(^{Em}L)Fe(OH)][KC222] (*red*) and (^{Em}L)Fe(OH) (*blue*). One electron reduction of (^{Em}L)Fe(OH) removes dipyrromethene vibration by changing delocalization over two pyrrole subgroup. Dipyrromethene vibration modes from (^{Em}L)Fe(OH) (υ(dipyrr) = 1538 cm^{-1}) are denoted with obelisk (†).

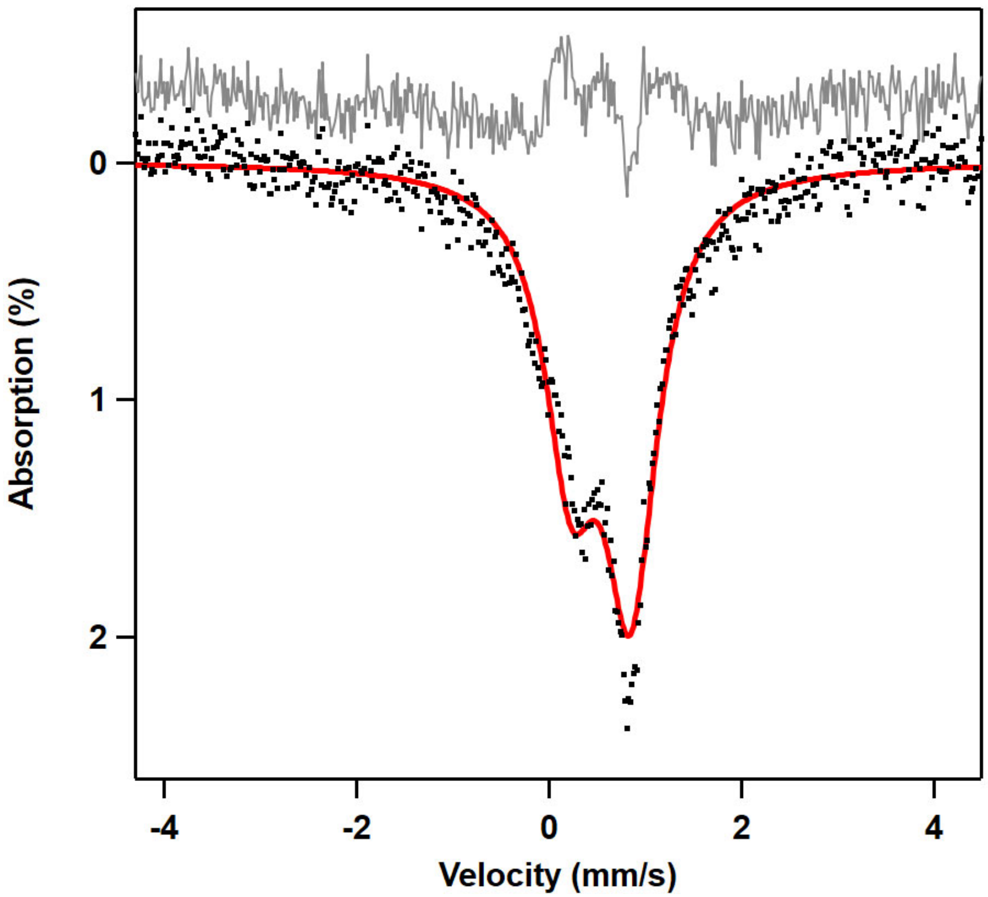

Figure S45. Zero-field ^{57}Fe Mössbauer spectrum of [(^{Em}L)Fe(OH)][KC_{222}] at 90 K. Isomer shift and quadrupole splitting are reported relative to α-iron foil at room temperature. The black dot, red line, and gray line represent the experimental data, fit, and residuals, respectively. δ (mm/s), $|\Delta E_Q|$ (mm/s), γ (mm/s) = 0.59, 0.68, 0.36.

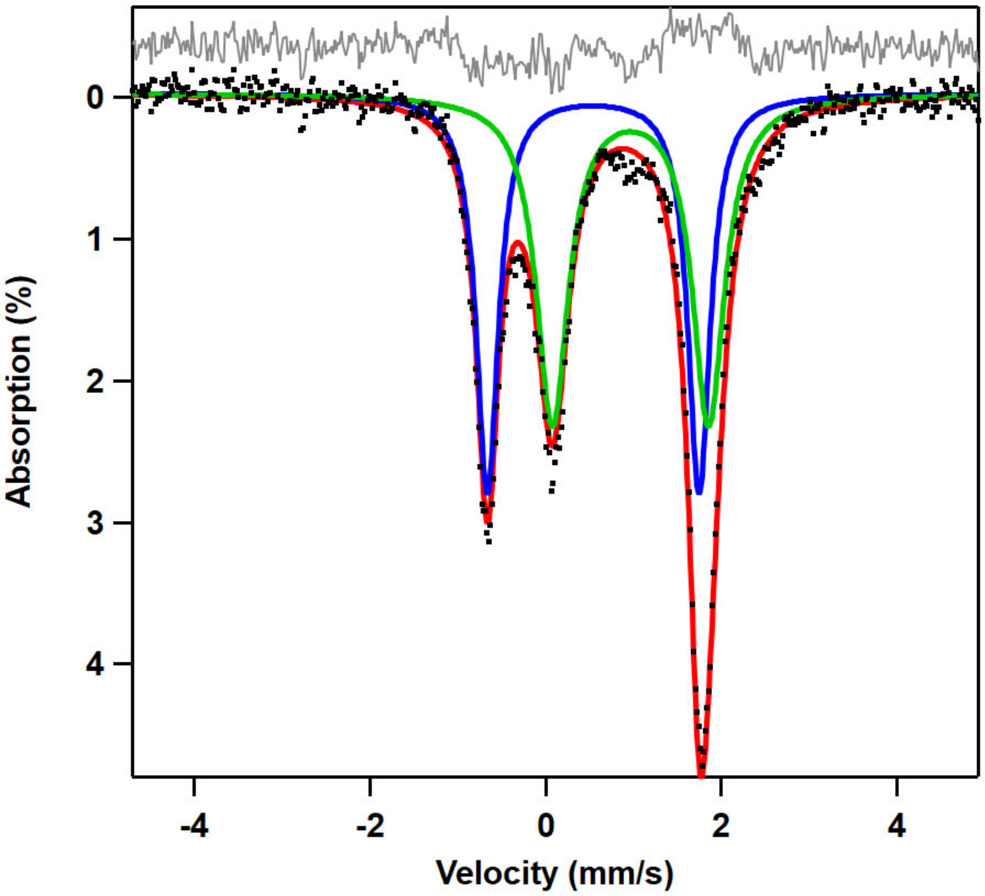

Figure S46. Zero-field ^{57}Fe Mössbauer spectrum of the crude mixture of the oxidation of [(^{Em}L)Fe(OH)][KC$_{222}$] with 1 equivalent of [Fc][BArF$_{24}$] at 90 K in frozen THF. Isomer shift and quadrupole splitting are reported relative to α-iron foil at room temperature. The black dot, red line, blue line, green line, and gray line represent the experimental data, fit, ferrocene (55%), (^{Em}L)Fe(OH) (45%) and residuals, respectively. δ (mm/s), $|\Delta E_Q|$ (mm/s), γ (mm/s) = 0.54, 2.41, 0.15 (*blue*, FeCp$_2$), and 0.96, 1.78, 0.22 (*green*, (^{Em}L)Fe(OH)).

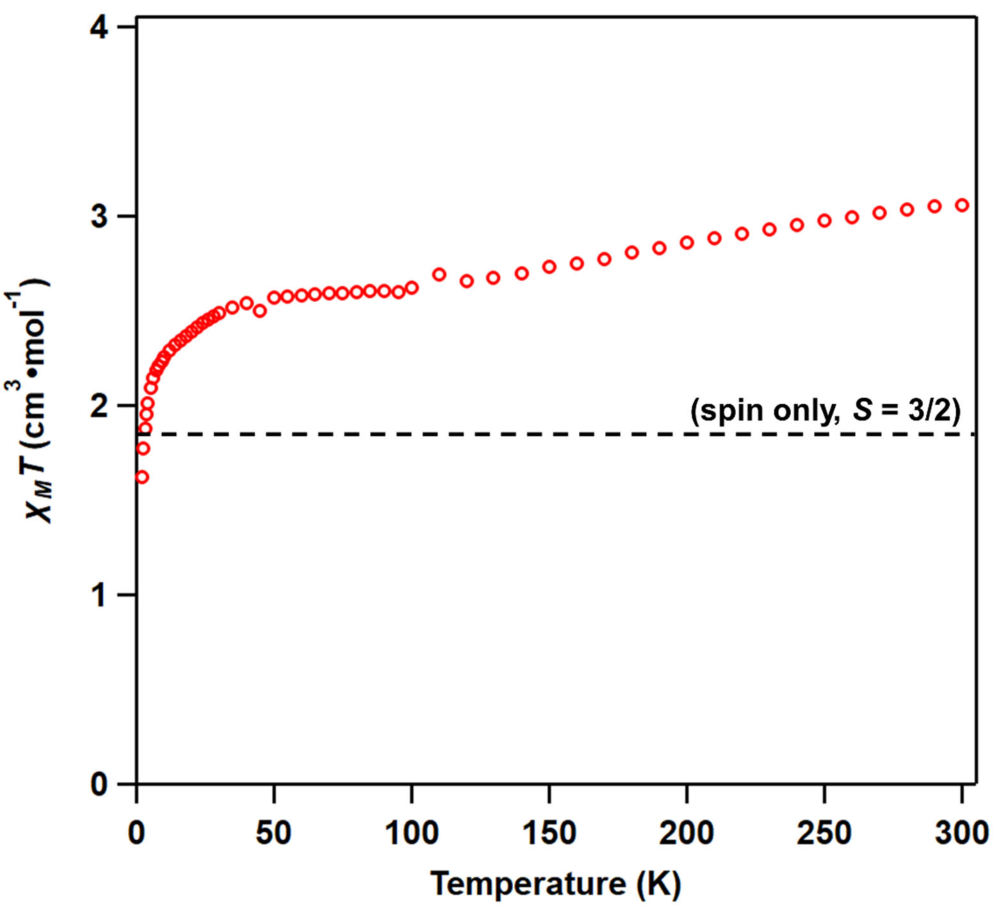

Figure S47. Temperature dependence of the molar magnetic susceptibility temperature product (χ_MT) of [(^{Em}L)Fe(OH)][KC_{222}] (**14**) (T = 300 K to 2 K; H = 1 T) (χ_MT (spin-only) = 1.875 (S = 3/2), 4.375 (S = 5/2)).

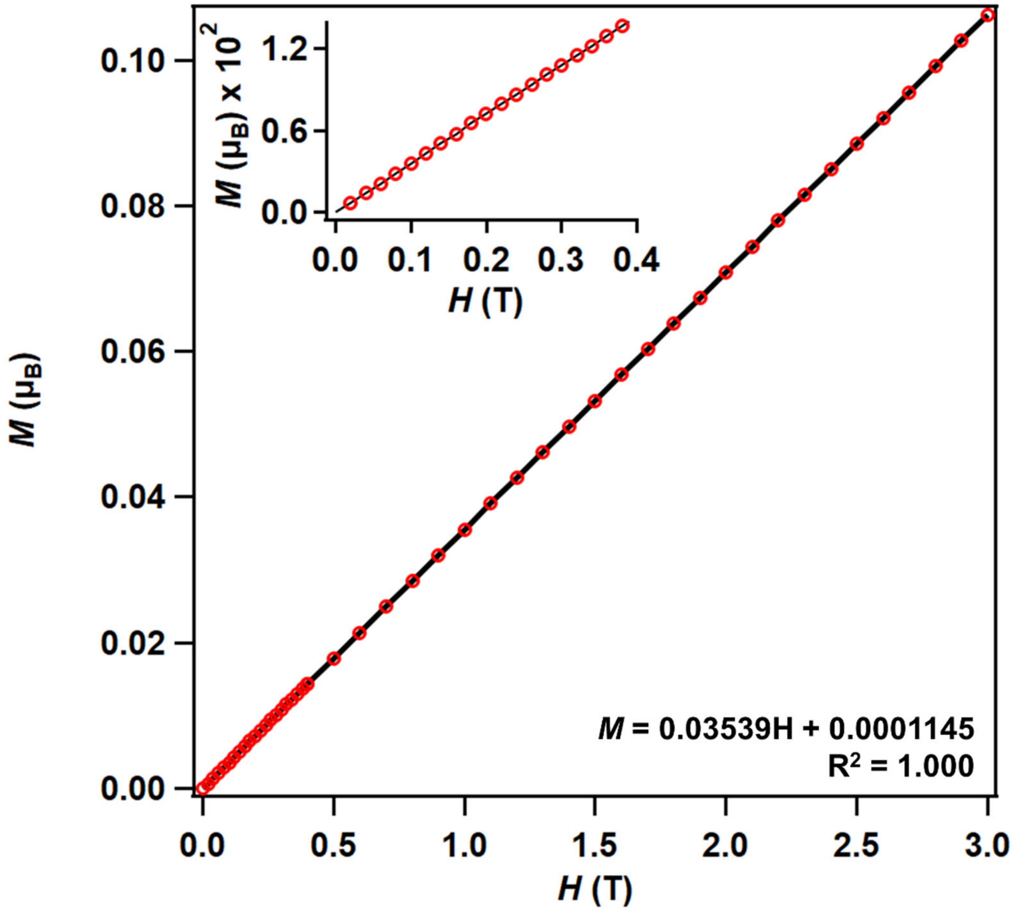

Figure S48. Field dependence of the magnetization of [(^{Em}L)Fe(OH)][KC222] (**14**) (T = 100 K; *H* = 0 – 3 T). The linearity of the relationship between *H* (T) and *M* (μ_B) indicates that the sample is free from ferromagnetic impurities.

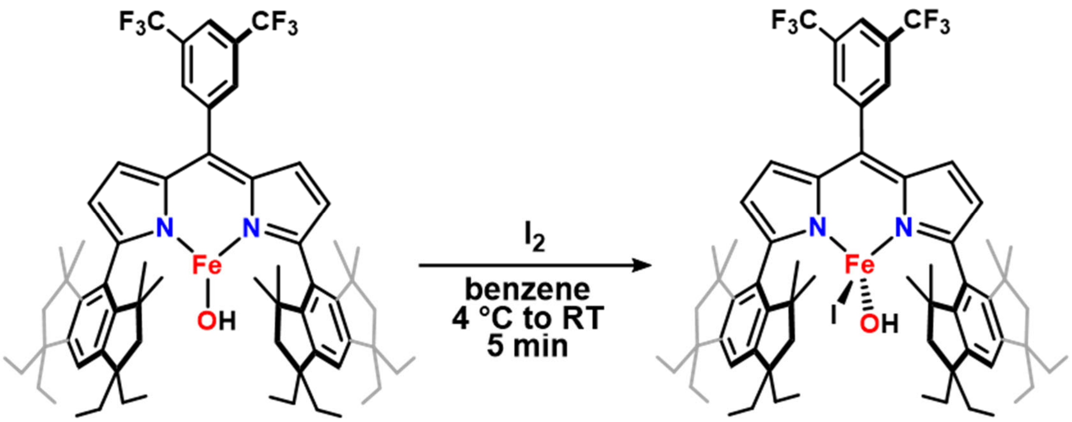

(^{Em}L)Fe(OH)(I) (15): To a thawing solution of excess I_2 (39.0 mg, 0.154 mmol) in 1 mL of benzene, was slowly added a solution of (^{Em}L)Fe(OH) (80.4 mg, 0.0746 mmol) in 3 mL of benzene. The solution was warmed up to room temperature, and the solution turned to deep red immediately. After being stirred for 5 minutes at room temperature, the solution was frozen and volatiles were removed *in vacuo*. The resulting powder was dissolved in hexanes and filtered through Celite in hexanes. The product was obtained as deep red powder after the volatiles were removed *in vacuo* (72.8 mg, 0.0605 mmol, 80%). ^{1}H NMR (500 MHz, 298 K, C_6D_6): δ 100.23, 17.27, 4.86, 8.98, 8.47, 6.25, 6.12, 5.92, 5.38, 4.97, 4.61, 3.92, 3.59, 2.30, 2.02, 1.14, –0.17 ppm. ^{19}F NMR (471 MHz, 298 K, C_6D_6): δ –62.84 (3F), –65.70 (3F) ppm. FTIR (ATR): υ(O–H) = 3642 cm^{-1}. Zero-field ^{57}Fe Mossbauer (90 K) (δ, $|\Delta E_Q|$ (mm/s)): 0.36, 1.33 (γ = 0.52 mm/s). HRMS (ESI^{-}): m/z Calc. 1248.4698 [$C_{65}H_{82}F_6FeIN_2O$+HCOO]$^{-}$, Found 1248.4709 [M+HCO_2]$^{-}$.

Note. We observed the generation of (^{Em}L)Fe(I) along with a new ferric species as a byproduct during the synthesis of (^{Em}L)Fe(OH)(I). We cannot fully prevent the generation of (^{Em}L)Fe(I), but the addition (^{Em}L)Fe(OH) into the excess I_2 is crucial to minimize the byproduct generation. The detailed investigation of the ferric byproduct is described below (Figure S65).

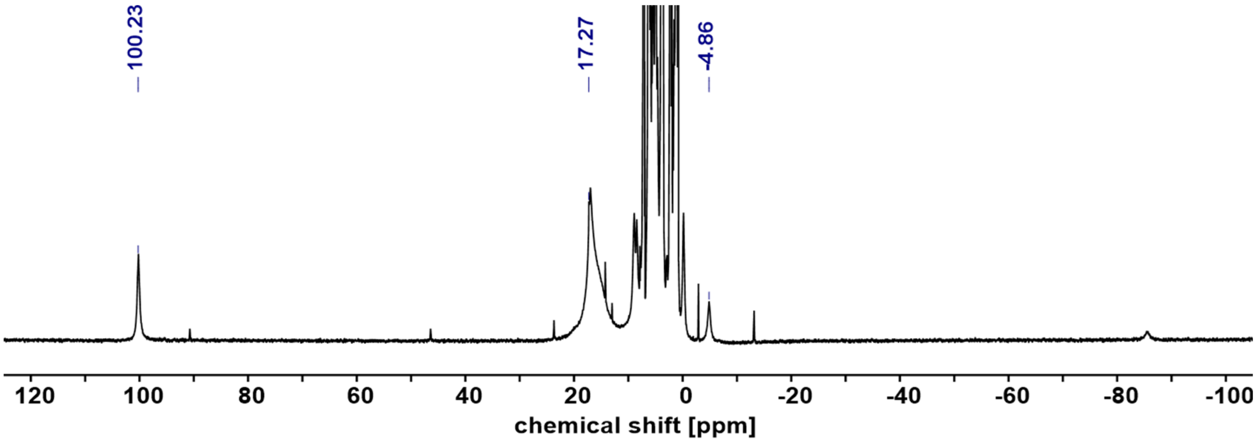

Figure S49. ^{1}H NMR spectrum of (^{Em}L)Fe(OH)(I), (500 MHz, 298 K, C_6D_6).

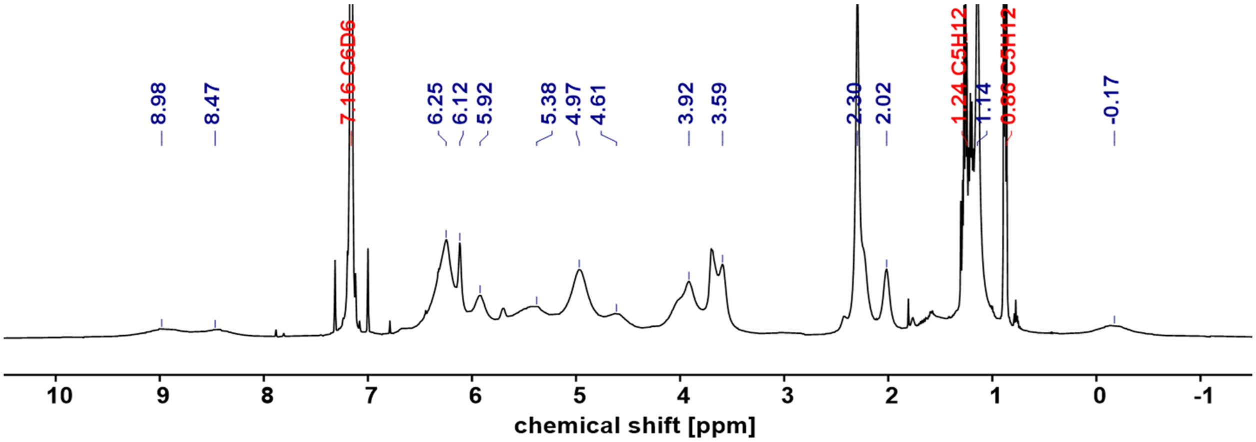

Figure S50. ^{1}H NMR spectrum of (^{Em}L)Fe(OH)(I), (500 MHz, 298 K, C_6D_6).

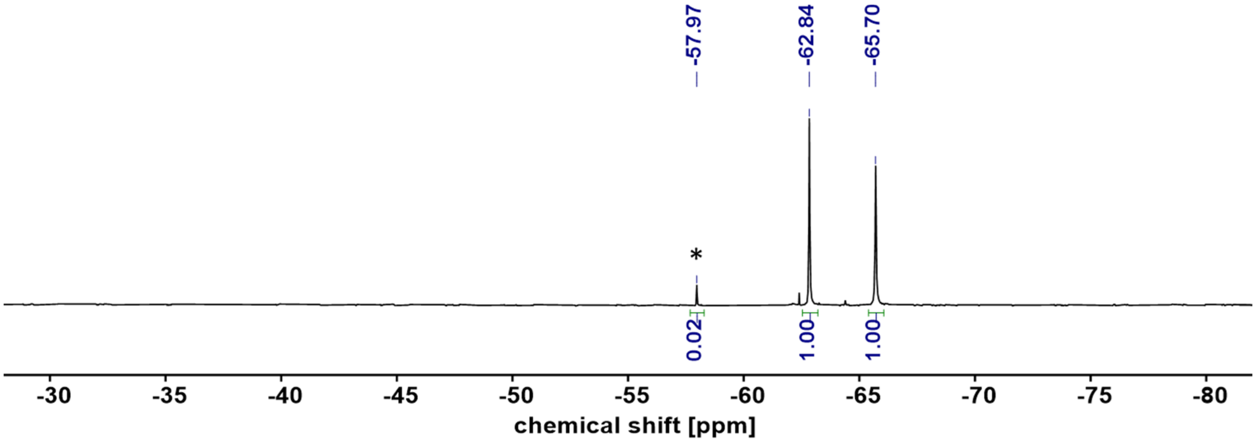

Figure S51. ^{19}F NMR spectrum of (^{Em}L)Fe(OH)(I), (471 MHz, 298 K, C_6D_6). (^{Em}L)Fe(I) were obtained as minor impurity (ca. 1 %) as denoted with asterisk (*), which was unable to purify by recrystallization.

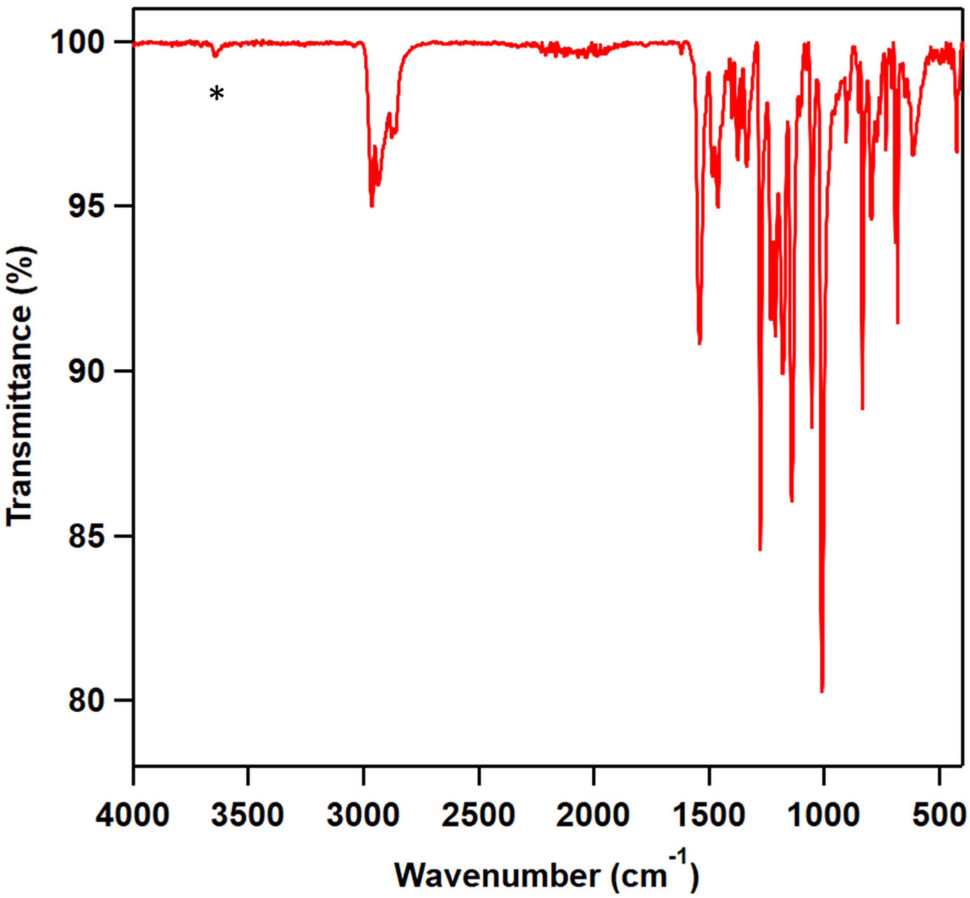

Figure S52. IR spectrum of (^{Em}L)Fe(OH)(I). O–H vibration modes (υ(O–H) = 3642 cm^{-1}) are denoted with asterisks (*).

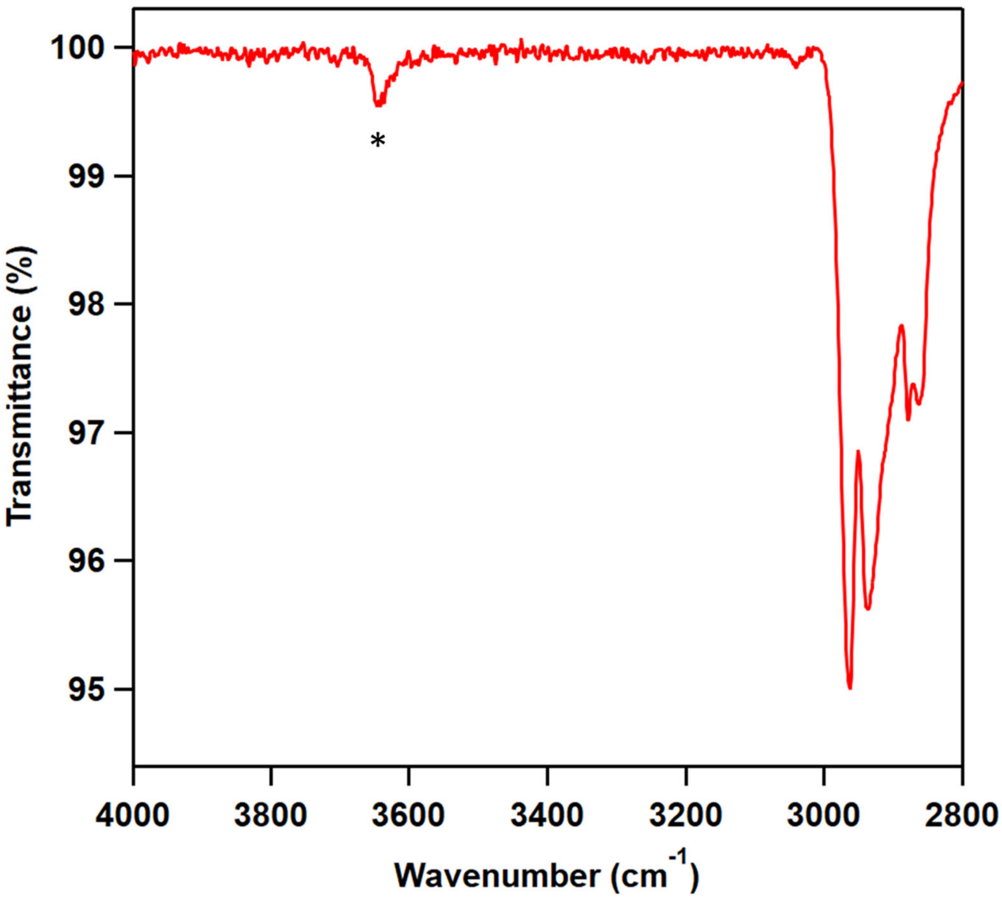

Figure S53. IR spectrum of (^{Em}L)Fe(OH)(I) highlighting O–H vibration. O–H vibration modes (υ(O–H) = 3642 cm^{-1}) are denoted with asterisks (*).

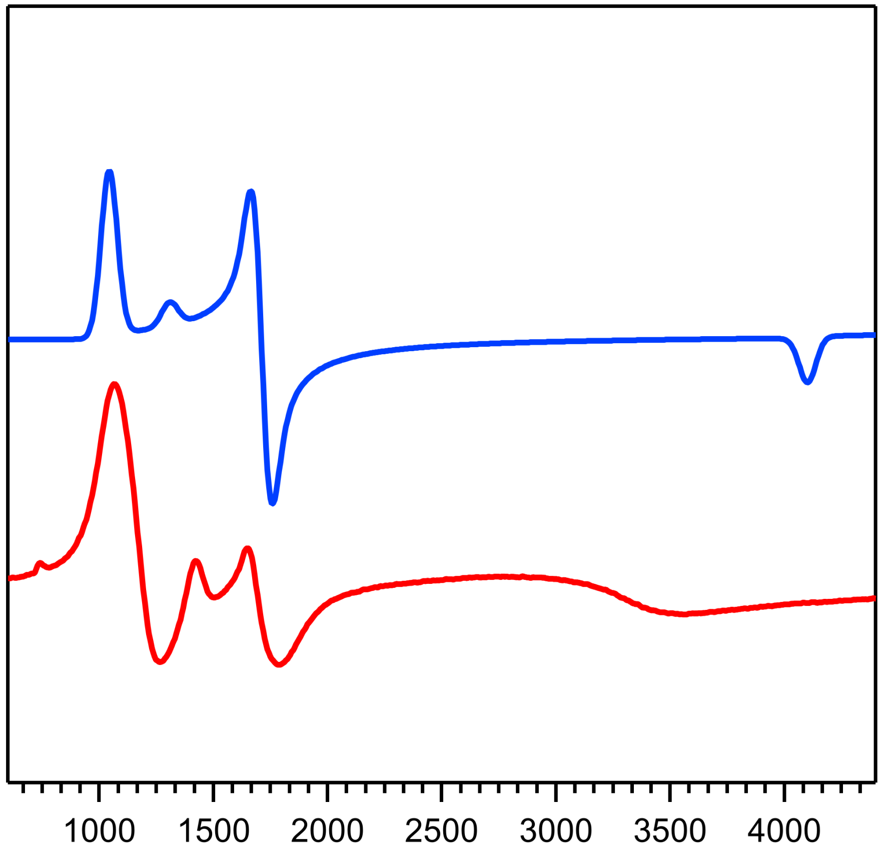

Figure S54. EPR spectrum of (^{Em}L)Fe(OH)(I) (red) in frozen toluene matrix at 77 K. The blue line represents a simulation with VisualRhombo[10] (E/D = 0.062, S = 5/2). Though the experimental spectrum shows a non-zero baseline at low field, we did not take account baseline for this broad spectrum as there is a risk causing spectral distortion.[11]

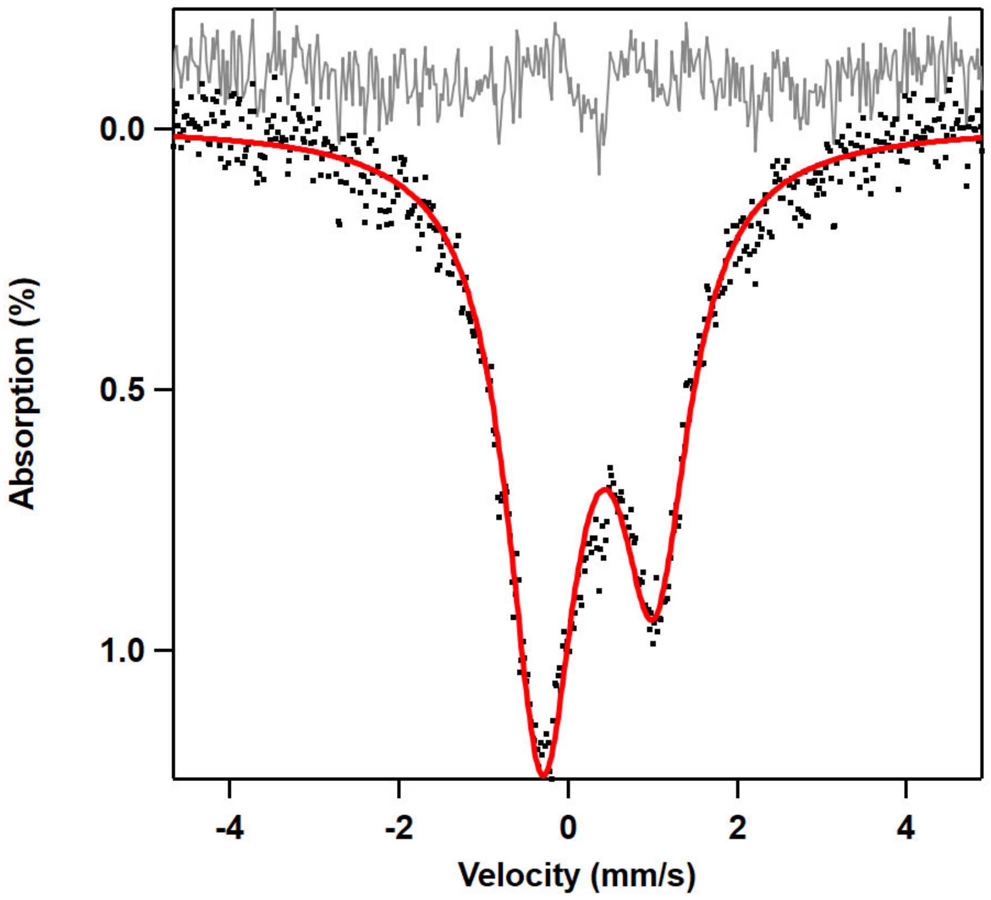

Figure S55. Zero-field ^{57}Fe Mössbauer spectrum of (^{Em}L)Fe(OH)(I) at 90 K. Isomer shift and quadrupole splitting are reported relative to α-iron foil at room temperature. The black dot, red line, and gray line represent the experimental data, fit, and residuals, respectively. δ (mm/s), $|\Delta E_Q|$ (mm/s), γ (mm/s) = 0.36, 1.33, 0.52

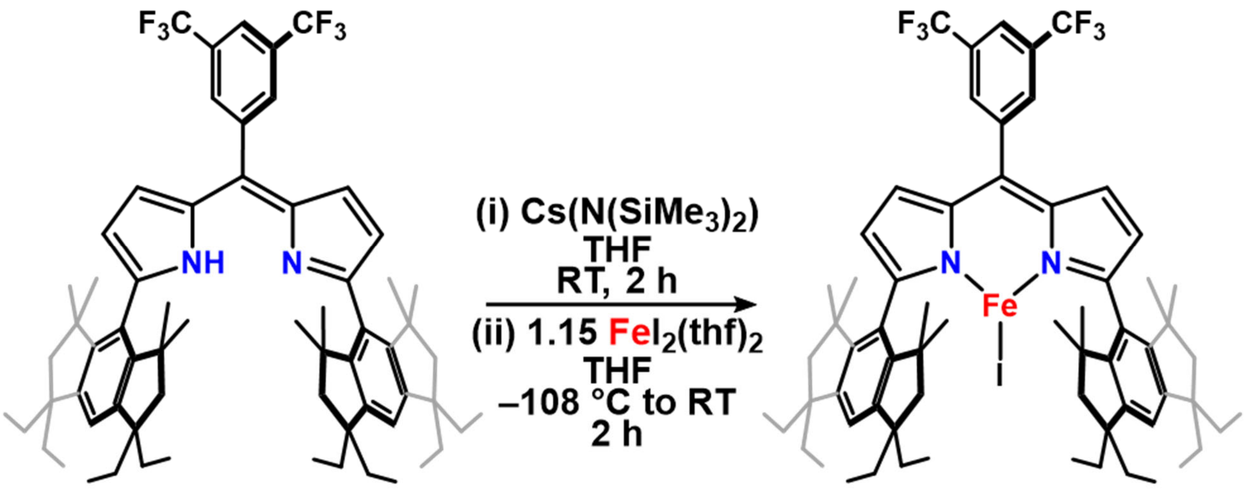

(^{Em}L)Fe(I) (16): In a dinitrogen-filled drybox, addition of solid cesium bis(trimethylsilyl) amide (0.095 g, 0.324 mmol, 1.03 equiv.) to (^{Em}L)H (0.316 g, 0.314 mmol, 1.00 equiv.) in tetrahydrofuran (3 mL) was accompanied by a rapid color change from orange to deep red-pink, indicative of formation of (^{Em}L)Cs.[12] After stirring for 2 h, the mixture was frozen in a liquid-nitrogen chilled cold well, followed by addition of crystalline $FeI_2(thf)_2$ (0.165 g, 0.362 mmol, 1.15 equiv.) upon thawing, resulting in a graduate color change to red-pink with concomitant precipitation of cesium iodide. After stirring for 3 h, the suspension was filtered over Celite, followed by removal of solvent *in vacuo*. The residual solid was dissolved in benzene and lyophilized to remove residual tetrahydrofuran. The residual solids were suspended in minimal pentane and allowed to stand at –35 ºC for 1 h. The suspension was filtered over Celite and rinsed with minimal cold pentane (*ca.* 2 mL), followed by elution with warm benzene and lyophilization to afford (^{Em}L)FeI (1) as a maroon solid (0.275 g, 74 %). Crystals suitable for single-crystal X-ray diffraction were obtained by allowing a concentrated solution of 1 in pentane to stand at –35 ºC for 48 h. ^{1}H NMR (500 MHz, 298 K, C_6D_6): δ 91.06, 46.68, 23.92, 17.32, 14.34, 6.72, 6.33, 5.71, 5.40, 3.70, 2.37, –2.95, –13.25, –86.23 ppm. ^{19}F NMR (470 MHz, 298 K, C_6D_6): δ –57.95 ppm. Zero-field ^{57}Fe Mössbauer (90 K) (δ, $|\Delta E_Q|$ (mm/s)): 0.67, 0.84 (γ = 0.16 mm/s). Anal. Calc. for $C_{65}H_{81}F_6FeIN_2{\bullet}C_5H_{12}$: C 66.77, H 7.44, N 2.22; Found: C 66.95, H 7.09, N 2.42 (one molecule of pentane is present from bulk recrystallization and is located in the unit cell).

Note. The isolated yield of **16** is increased by allowing the *n*-pentane fraction from filtration to stand at –35 ºC over several days, affording crystalline **16**.

Note. Using non-crystalline, powder $FeI_2(thf)_2$ as a metal source resulted in impure metalation.

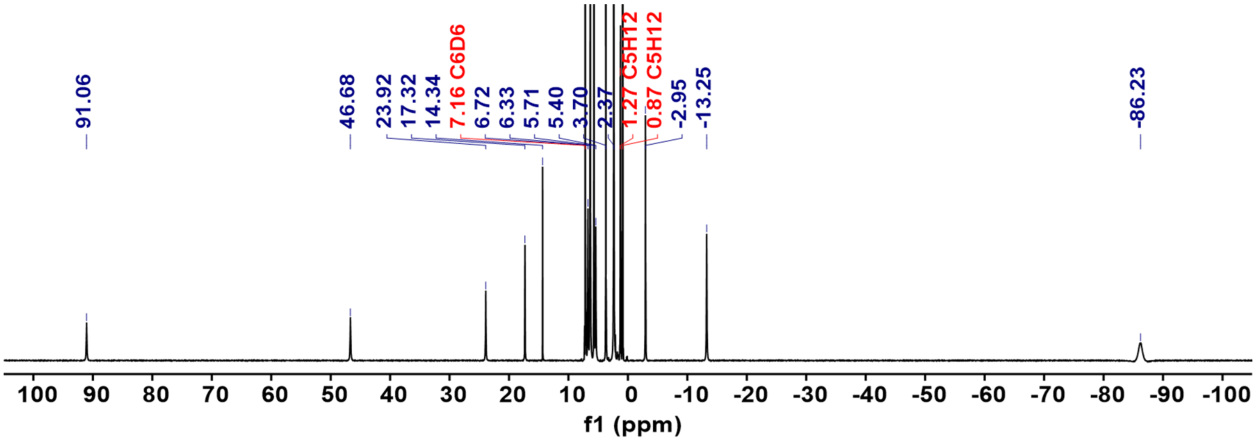

Figure S56. ^{1}H NMR spectrum of (^{Em}L)Fe(I), (500 MHz, 298 K, C_6D_6).

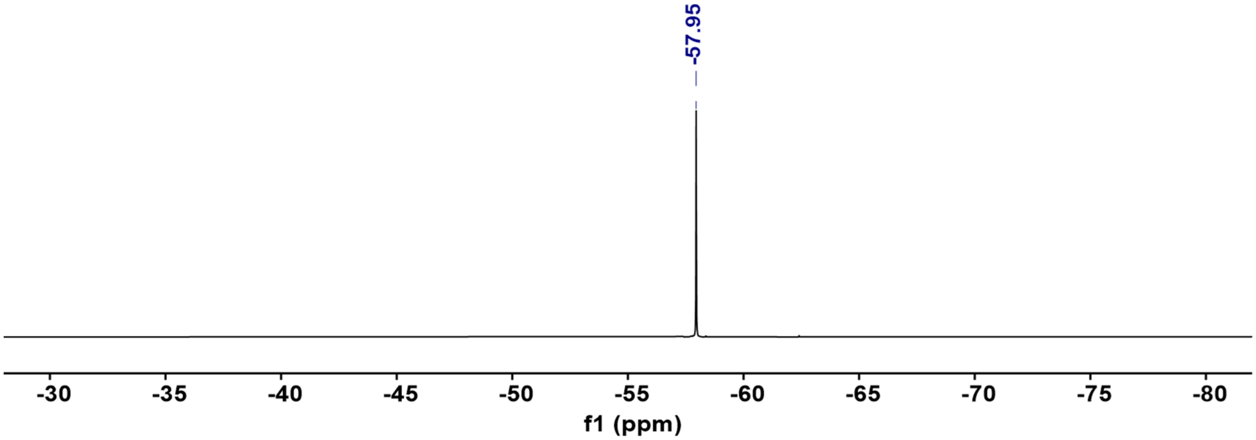

Figure S57. ^{19}F NMR spectrum of (^{Em}L)Fe(I), (471 MHz, 298 K, C_6D_6).

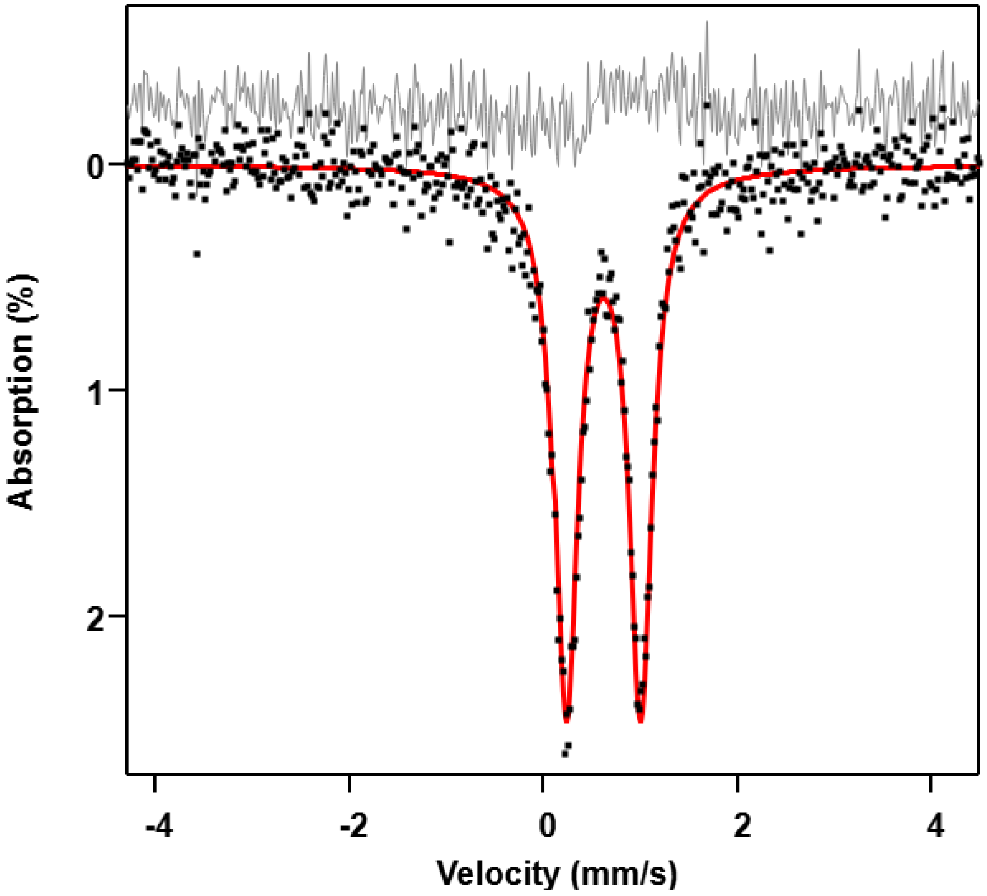

Figure S58. Zero-field ^{57}Fe Mössbauer spectrum of (^{Em}L)Fe(I) at 90 K. Isomer shift and quadrupole splitting are reported relative to α-iron foil at room temperature. The black dot, red line, and gray line represent the experimental data, fit, and residuals, respectively. δ (mm/s), $|\Delta E_Q|$ (mm/s), γ (mm/s) = 0.61, 0.77, 0.15

Additional characterization of (^{Em}L)Fe(OH)

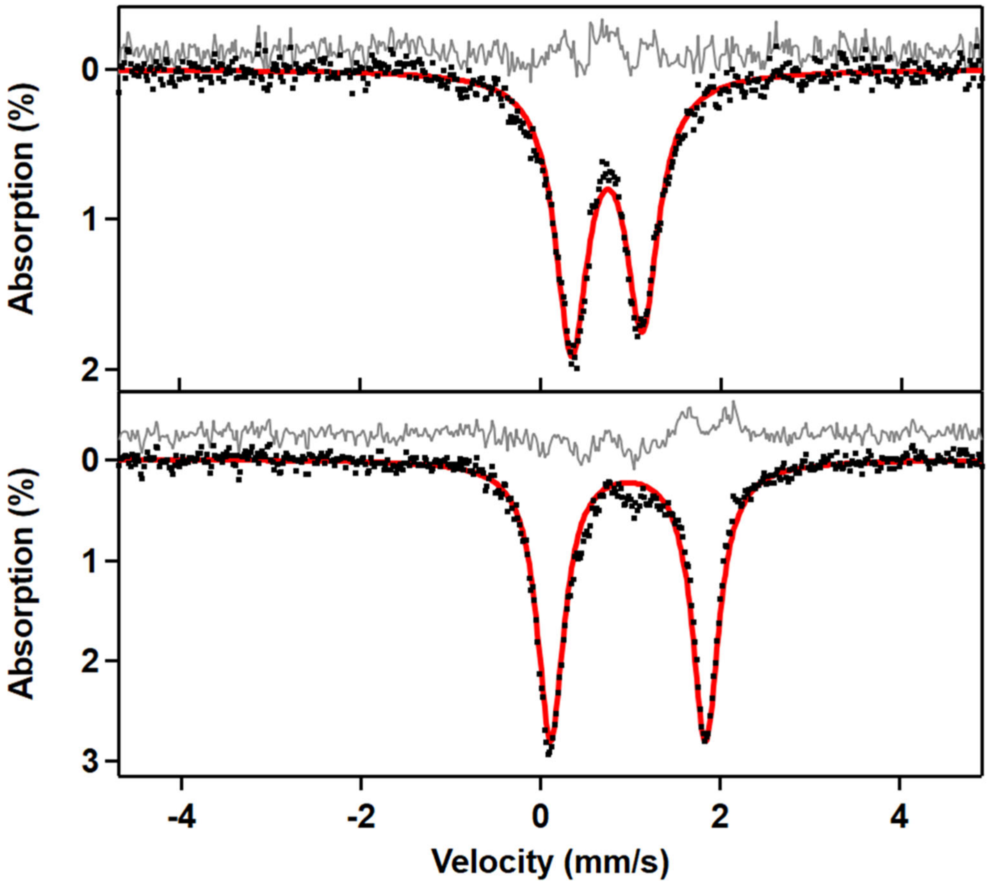

Figure S59. Zero-field ^{57}Fe Mössbauer spectrum of (^{Em}L)Fe(OH) at 90 K in frozen benzene (*top*) and THF (*bottom*) matrix. Isomer shift and quadrupole splitting are reported relative to α-iron foil at room temperature. The black dot, red line, and gray line represent the experimental data, fit, and residuals, respectively. δ (mm/s), $|\Delta E_Q|$ (mm/s), γ (mm/s) = 0.73, 0.78, 0.22 (*top*) and δ (mm/s), $|\Delta E_Q|$ (mm/s), γ (mm/s) = 0.97, 1.72, 0.18 (*bottom*).

Characterization of (^{Em}L)Fe(κ^2O,O-HCO_3)

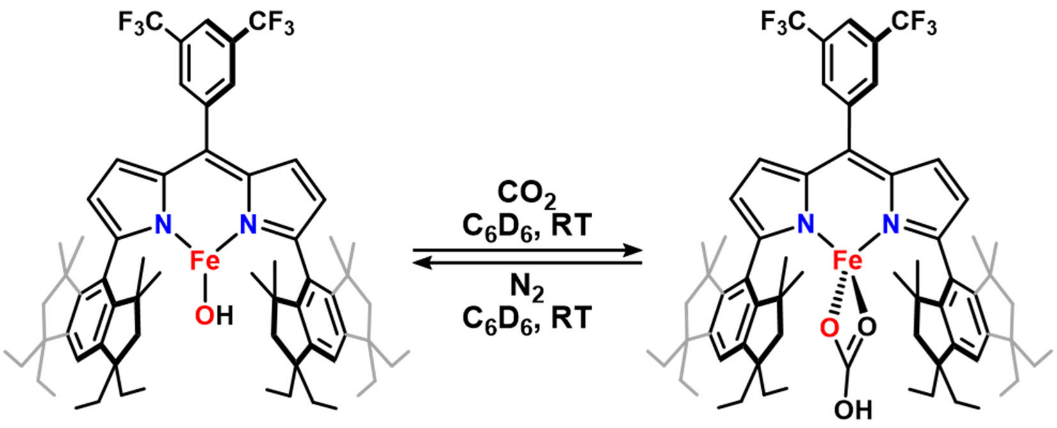

Determination of equilibrium constant by NMR spectroscopy

In the drybox, a solution of (^{Em}L)Fe(OH) (6.12 mM) in 1.00 mL C_6D_6 was transferred to J-Young NMR tube, and sealed. The tube was degassed with three freeze-pump-thaw cycles, and CO_2 gas (0, 70, 162, 415,821 torr) was introduced into the headspace (1.84 mL) at room temperature. The solution was mixed vigorously, then ^{1Em}L)Fe(κ^2O,O-HCO_3), [**5**], were obtained by integration by peaks in ^{19}F NMR spectrum.

The pressure of CO_2 at the equilibrium (p_{CO2}(equilibrium)) were different from the initially introduced into the headspace (p_{CO2}(initial)) due to (*i*) dissolving in the C_6D_6 solution (solubility of CO_2 in benzene, $S_{CO2/PhH}$, = 0.1088 mol^{-1} • L^{-1} • bar^{-1}), and (*ii*) consumption by equilibrium with CO_2. To reflect the pressure change, we calculated the p_{CO2}(equilibrium) by using the relation S1 (Table S1).

$$\begin{aligned} & n_{CO2}(\text{total}) \\ &= p_{CO2}(\text{initial}) \cdot \text{V(gas)} \\ &= p_{CO2}(\text{equilibrium}) \cdot (\text{V(gas)} + S_{CO2/PhH} \cdot \text{V(solution)}) + \text{V(solution)} \cdot [\mathbf{5}] \end{aligned} \quad (S1)$$

Table S1. Pressure of CO_2 in equilibrium converted from the initially introduced pressure.

p_{CO2}(initial) (torr)	p_{CO2}(equilibrium) (torr)	p_{CO2}(equilibrium) (atm)
0	0	0
70	12	0.016
162	44.1	0.0581
415	81.3	0.107
821	299	0.393

Then, the equilibrium constant was obtained from the slope of a plot ([**5**]/[**1**] vs p_{CO2}(equilibrium)) following relation S2 (Figure S57), and the free energy was obtained by equation S3.

$$K_{eq} = [\mathbf{5}] \bullet [\mathbf{1}]^{-1} \bullet p_{CO2}^{-1}(\text{equilibrium}) \quad (S2)$$
$$\Delta G° = -R \bullet T \bullet \ln(K_{eq}) \quad (S3)$$

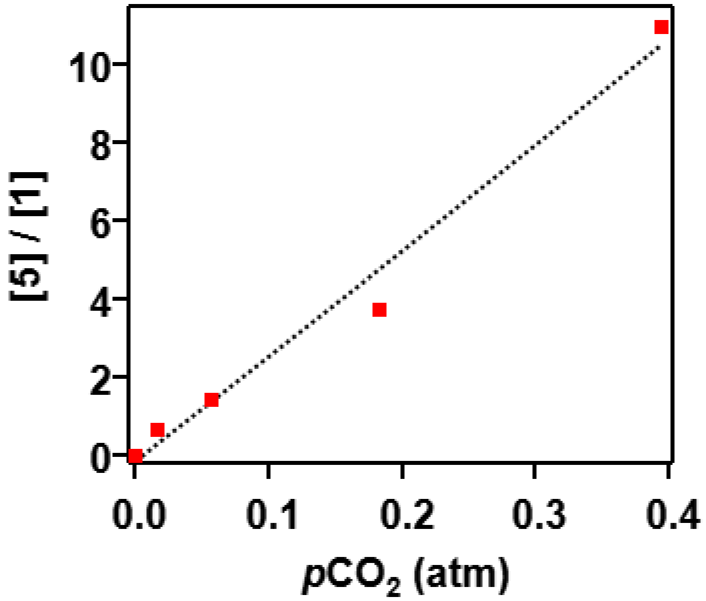

Figure S60. Plot of [**5**]/[**1**] vs p_{CO2}(equilibrium). The slope for the regression ($R^2 = 0.982$) is K_{eq} following the relation *S2*.

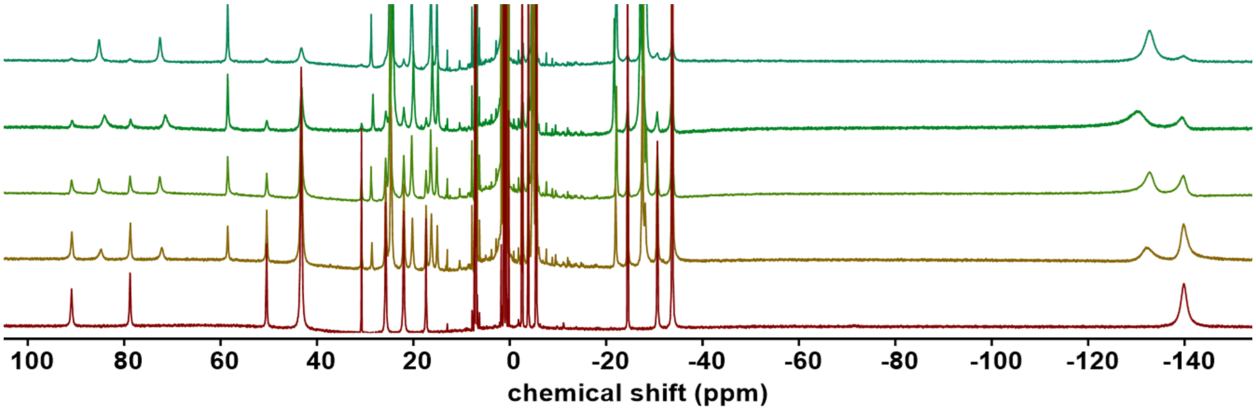

Figure S61. Stacked ^{1}H NMR spectra of the (^{Em}L)Fe(OH) under varying initial CO_2 pressure (0 torr (*maroon*, bottom), 70 torr (*golden*), 162 torr (*yellow-green*), 415 torr (*green*), and 821 torr (*forest-green*, top)) (500 MHz, 298 K, C_6D_6).

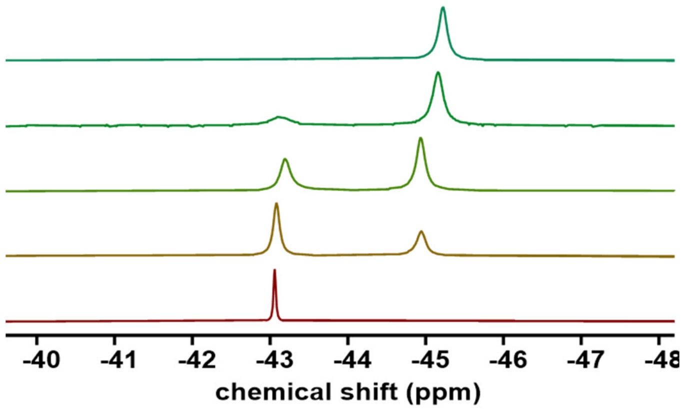

Figure S62. Stacked ^{19}F NMR spectra of the (^{Em}L)Fe(OH) under varying initial CO_2 pressure (0 torr (*maroon*, bottom), 70 torr (*golden*), 162 torr (*yellow-green*), 415 torr (*green*), and 821 torr (*forest-green*, top)) (471 MHz, 298 K, C_6D_6).

Characterization of 5 with Zero-field ^{57}Fe Mössbauer spectroscopy

In the drybox, a solution of (^{Em}L)Fe(OH) (42.2 mg, 0.0392 mmol) in 0.7 mL benzene was transferred to the Mössbauer sample holder placed in a Schlenk flask. After the flask was degassed by three freeze-pump-thaw cycles carefully not to spill the sample in the holder, the headspace (~50 mL) was charged with CO_2 gas (800 torr) at room temperature. The solution was equilibrated with CO_2 gas at room temperature for 10 minutes. Then, the flask was placed into the liquid nitrogen to quickly freeze the solution. The frozen solution was mounted at Mössbauer spectrometer, and the spectrum was collected at 90 K.

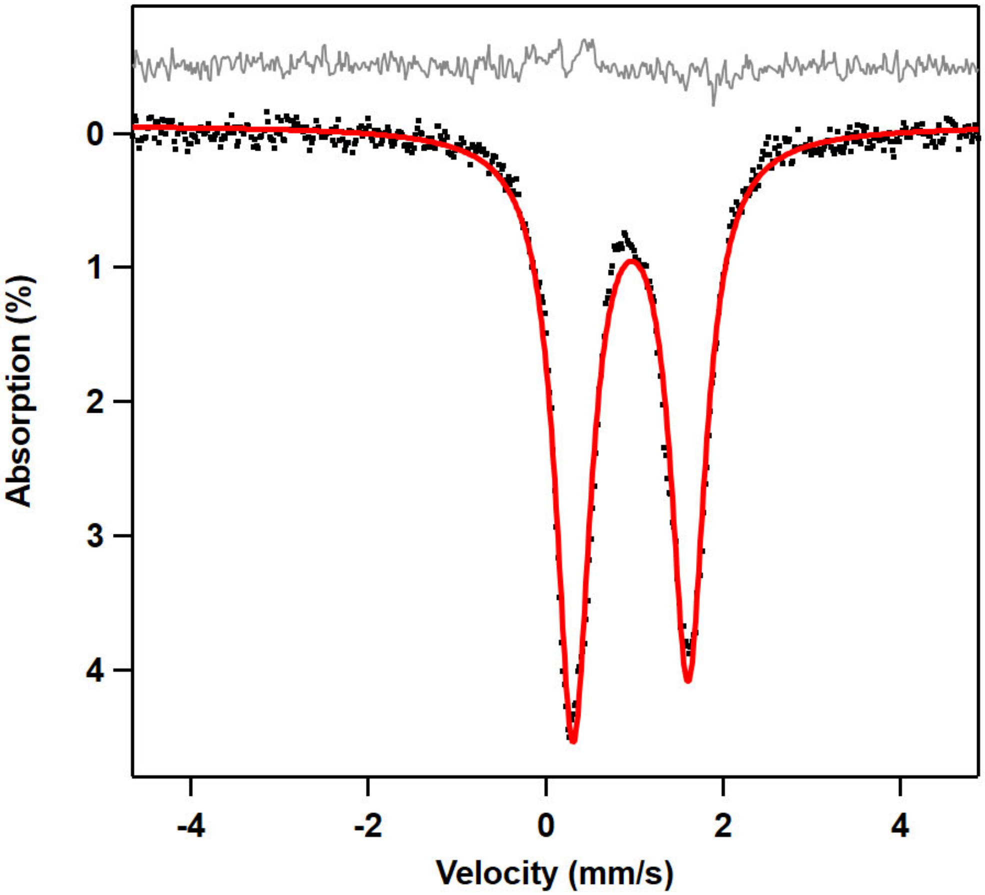

Figure S63. Zero-field ^{57}Fe Mössbauer spectrum of (^{Em}L)Fe(HCO_3) at 90 K. Isomer shift and quadrupole splitting are reported relative to α-iron foil at room temperature. The black dot, red line, and gray line represent the experimental data, fit, and residuals, respectively. δ (mm/s), $|\Delta E_Q|$ (mm/s), γ (mm/s) = 0.96, 1.29, 0.24.

Characterization of 5 with IR spectroscopy

In the drybox, a crystalline powder of **1** was transferred to the Schlenk flask. After the flask was evacuated under vacuum, the headspace (~50 mL) was charged with CO_2 gas (800 torr) at room temperature. After the crystalline powder was equilibrated with CO_2 gas at room temperature for 1 day, IR spectrum was collected using ATR-FTIR spectrometer in an Argon drybox to minimize sample preparation time (The solid was exposed to Argon environment less than 10 seconds). IR spectrum was repeatedly measured after 10 minutes to monitor CO_2 release from the crystalline solid.

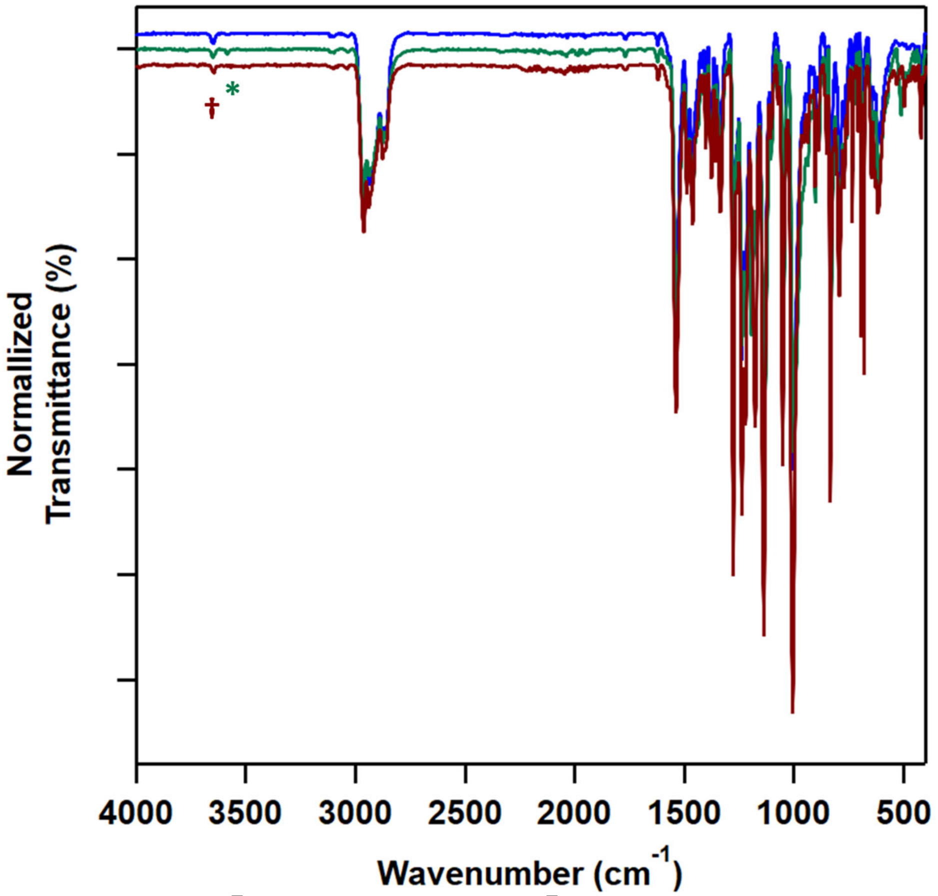

Figure S64. IR spectra of (^{Em}L)Fe(OH) (*maroon*), (^{Em}L)Fe(OH) under CO_2 for 1 day (*forest-green*), and (^{Em}L)Fe(OH) under CO_2 for 1 day followed by under Argon for 10 minutes at room temperature (*blue*). ν(OH) from (^{Em}L)Fe(OH) and (^{Em}L)Fe(κ^2O,O-HCO_3) are denoted with obelisk (†) and asterisk (*), respectively.

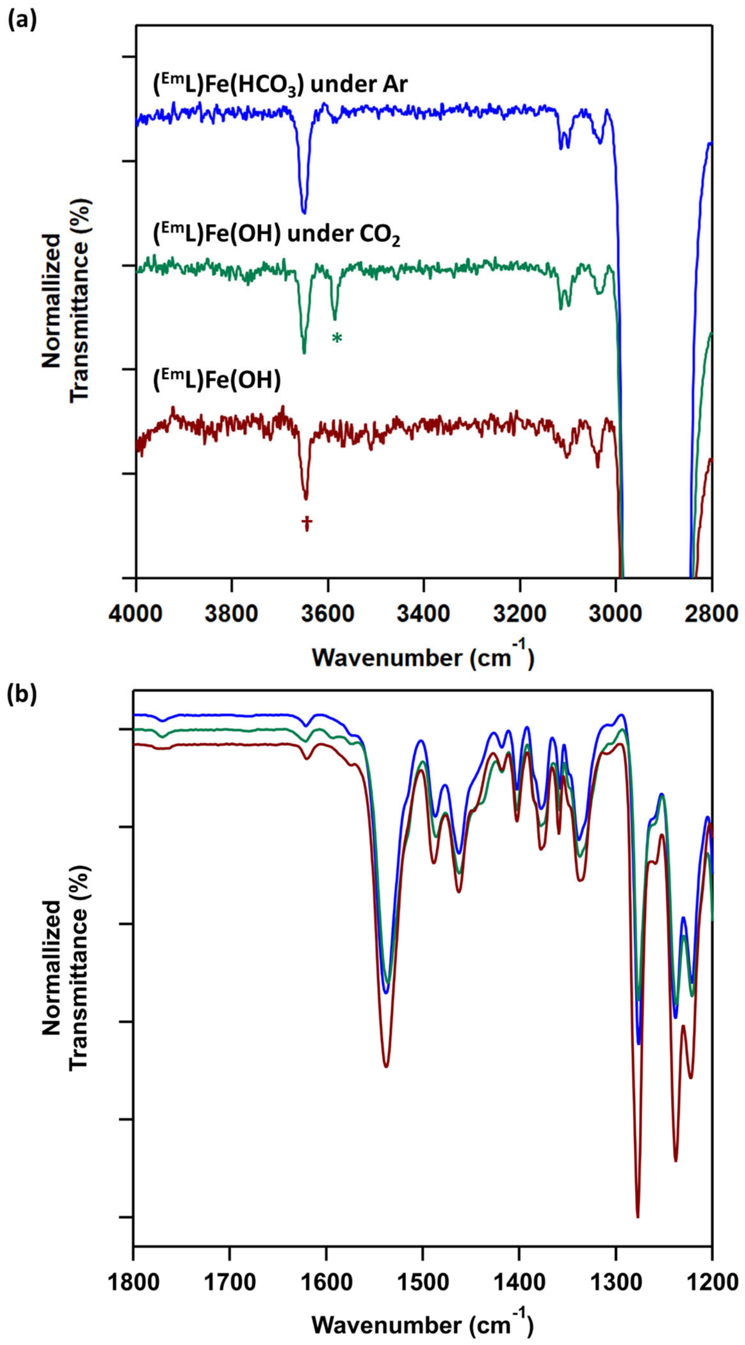

Figure S65. IR spectra highlighting O–H vibrational modes (a) and the region of asymmetric C=O stretch (b) of (^{Em}L)Fe(OH) (*maroon*), (^{Em}L)Fe(OH) under CO_2 for 1 day (*forest-green*), and (^{Em}L)Fe(OH) under CO_2 for 1 day followed by under Argon for 10 minutes at room temperature (*blue*). ν(OH) from (^{Em}L)Fe(OH) and (^{Em}L)Fe(κ^2O,O-HCO_3) are denoted with obelish (†) and asterisk (*), respectively.

Reactivity study of (^{Em}L)Fe(OH)(I) (15)

To a solution of (^{Em}L)Fe(OH)(I) (**15**) (10.0 mg, 0.00830 mmol) in 0.5 mL of C_6D_6, added a solution of Gomberg's dimer (7.7 mg, 0.0158 mmol) in 0.5 mL C6D6 at room temperature. After stirred at room temperature for 1 hour, the reaction was completed as monitored by ^{1}H NMR spectroscopy. We observed the generation of Ph_3COH from ^{1}H NMR.

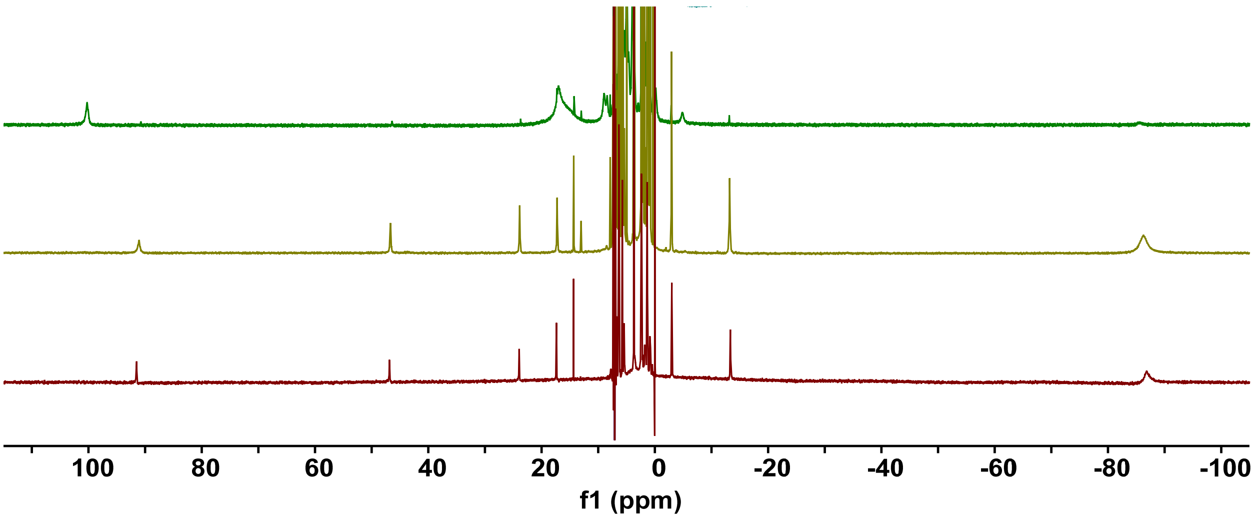

Figure S66. Stacked ^{1}H NMR spectra of the (^{Em}L)Fe(OH)(I) (*green*, top), the reaction between (^{Em}L)Fe(OH)(I) with 2 equivalent of Gomberg's dimer at room temperature for 1 hour (*golden*, middle), and (^{Em}L)Fe(I) (*maroon*, bottom), (500 MHz, 298 K, C_6D_6).

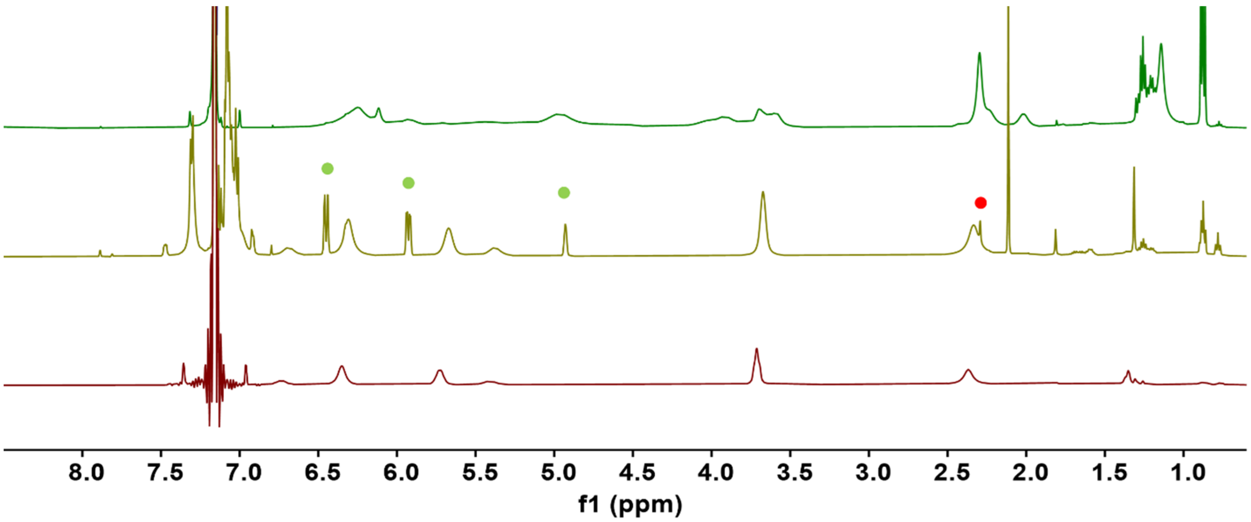

Figure S67. Stacked ^{1}H NMR spectra highlighting diamagnetic complexes of the (^{Em}L)Fe(OH)(I) (*green*, top), the reaction between (^{Em}L)Fe(OH)(I) with 2 equivalent of Gomberg's dimer at room temperature for 1 hour (*golden*, middle), and (^{Em}L)Fe(I) (*maroon*, bottom), (500 MHz, 298 K, C_6D_6). The consumption and generation of Gomberg's dimer and Ph_3COH are confirmed by comparing with reference ^{1}H NMR spectrum.[13] Remaining Gomberg's dimer and generated Ph_3COH are denoted with green (6.46 ppm (2H, d, 10.3 Hz), 5.93 ppm (2H, dd, 10.3 Hz, 4.0 Hz), 4.94 ppm (1H, br)) and red dot (2.29 ppm, Ph_3CO–*H*), respectively. The yield of Ph_3COH is 91(1)% as determined by ^{1}H NMR spectroscopy using $(Me_3Si)_2O$ as an internal standard.

Investigation of (^{Em}L)Fe(OH) (1) oxidation

An attempt to synthesize (^{Em}L)Fe(OH)(I) (**15**) by adding I_2 to (^{Em}L)Fe(OH) (**1**) generated an unidentified high-spin ferric impurity as well as **15** as determined by EPR spectroscopy (Figure S63). However, the reversal the order of addition can prevent the ferric byproduct generation. Thus, we hypothesized that the hydroxyl radical on **15** transferred to **1**, as **15** transfers OH$^•$ to carboradical.

Notably, the same ferric species was observed as a sole ferric species by EPR spectrum from the reaction mixture of (^{Em}L)Fe(OH) (**1**) with [Fc][$BArF_{24}$]. However, we cannot cleanly isolate the ferric product. Since OH$^•$ transfer to **1** was observed from dipyrrinato ferric hydroxo **8**, we hypothesized that the oxidation of **1** immediately induces OH$^•$ transfer to **1** in the mixture. Thus, we estimated the amount of the generated ferric species by titrating with KC_8 as followed: To a frozen solution of **1** (23.0 mg, 0.0214 mmol) in 1.5 mL THF, added a suspension of [Fc][$BArF_{24}$] (24.8 mg, 0.0236 mmol) in 2 mL THF. After the reaction mixture was stirred at room temperature for 30 minutes, the resulting solution was filtered through Celite in benzene to remove remaining [$FeCp_2$][$BArF_{24}$]. The ^{19}F NMR spectrum of the product (Figure S66, bottom) was fully overlapped with CF_3 from [$BArF_{24}$]$^-$. The filtrated was frozen, and a suspension of KC_8 (1.8 mg, 0.013 mmol) in 1 mL THF. Generation of (^{Em}L)Fe(OH) (**1**) was observed by ^{19}F NMR spectrum (Figuse S66, middle). Then, addition of a suspension of KC_8 (1.6 mg, 0.012 mmol) and cryptand [2.2.2] (4.8 mg, 0.013 mmol) resulted in complete conversion of (^{Em}L)Fe(OH) (**1**) to [(^{Em}L)Fe(OH)][KC_{222}] (**7**) as determined by ^{19}F NMR spectroscopy (Figure S65, top). The titration results imply that a half equivalent of the high spin ferric species was generated from **1**. Additionally, an IR spectrum showed strong OH vibration (ν(OH) = 3646 cm^{-1}). In sum, we concluded the OH$^•$ transfer from ferric hydroxo species to ferrous hydroxo generated high-spin ferric dihydroxo, (^{Em}L)Fe$(OH)_2$ (**SI-3**).

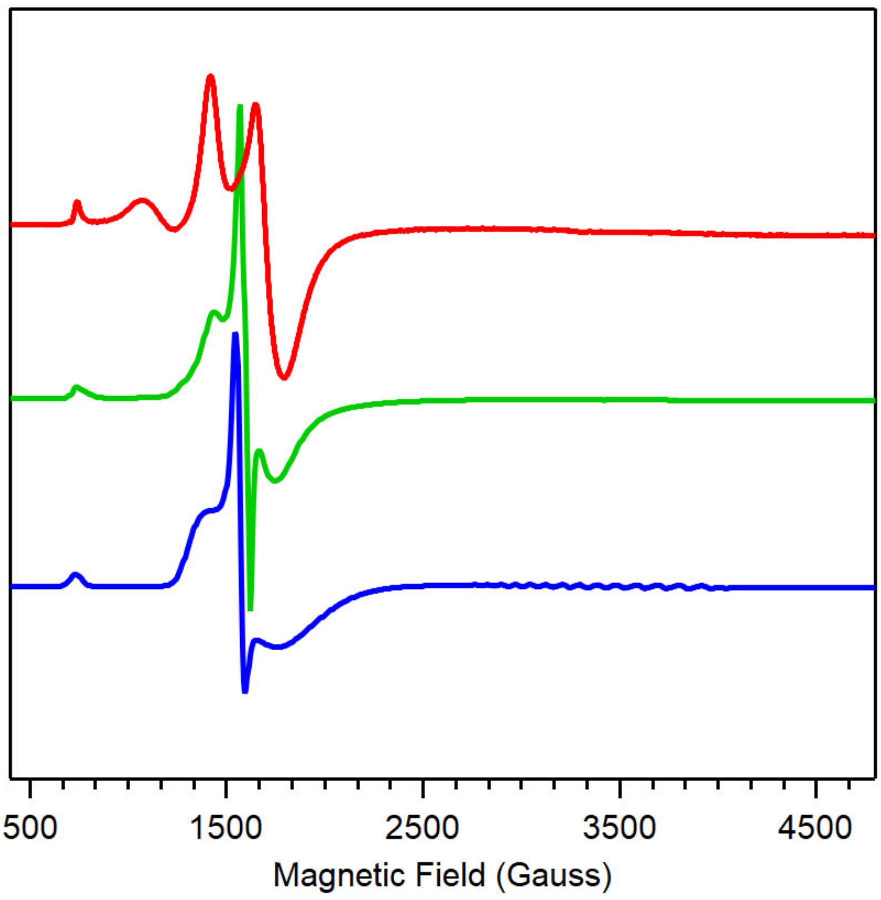

Figure S68. Stacked EPR spectra of the crude mixture of I_2 addition into (^{Em}L)Fe(OH) (red), (^{Em}L)Fe(OH)$_2$ (**SI-3**) (green) in 2-MeTHF at 77 K, and a simulation of green spectrum with VisualRhombo[10] (E/D = 0.22, S = 5/2) (blue).

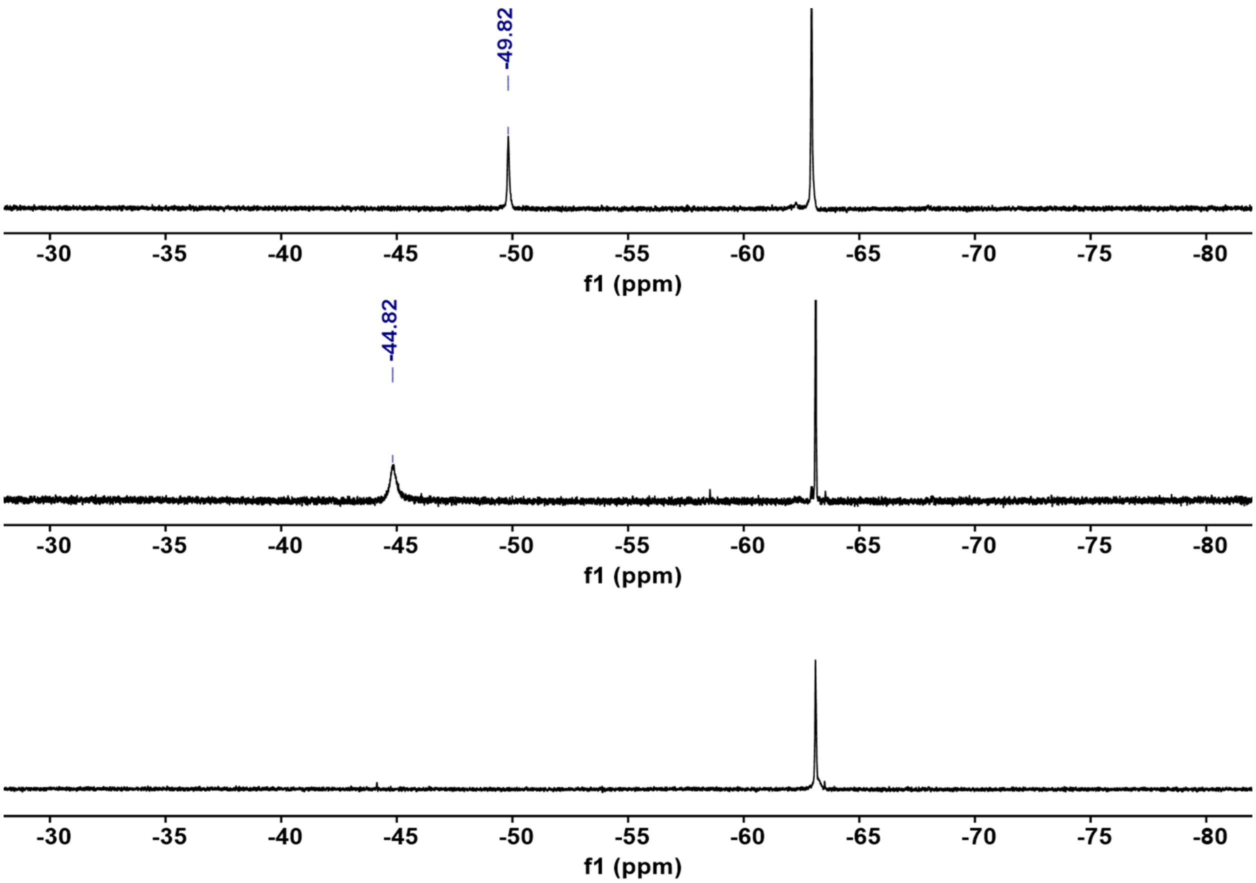

Figure S69. Stacked ^{19}F NMR spectra of the crude mixture of (^{Em}L)Fe(OH) and [$FeCp_2$][$BArF_{24}$] (bottom), addition of 0.5 equivalent of KC_8 to the crude mixture (middle), and addition of 1.0 equivalent of KC_8 to the crude mixture (top), (471 MHz, 298 K, C_6D_6+THF).

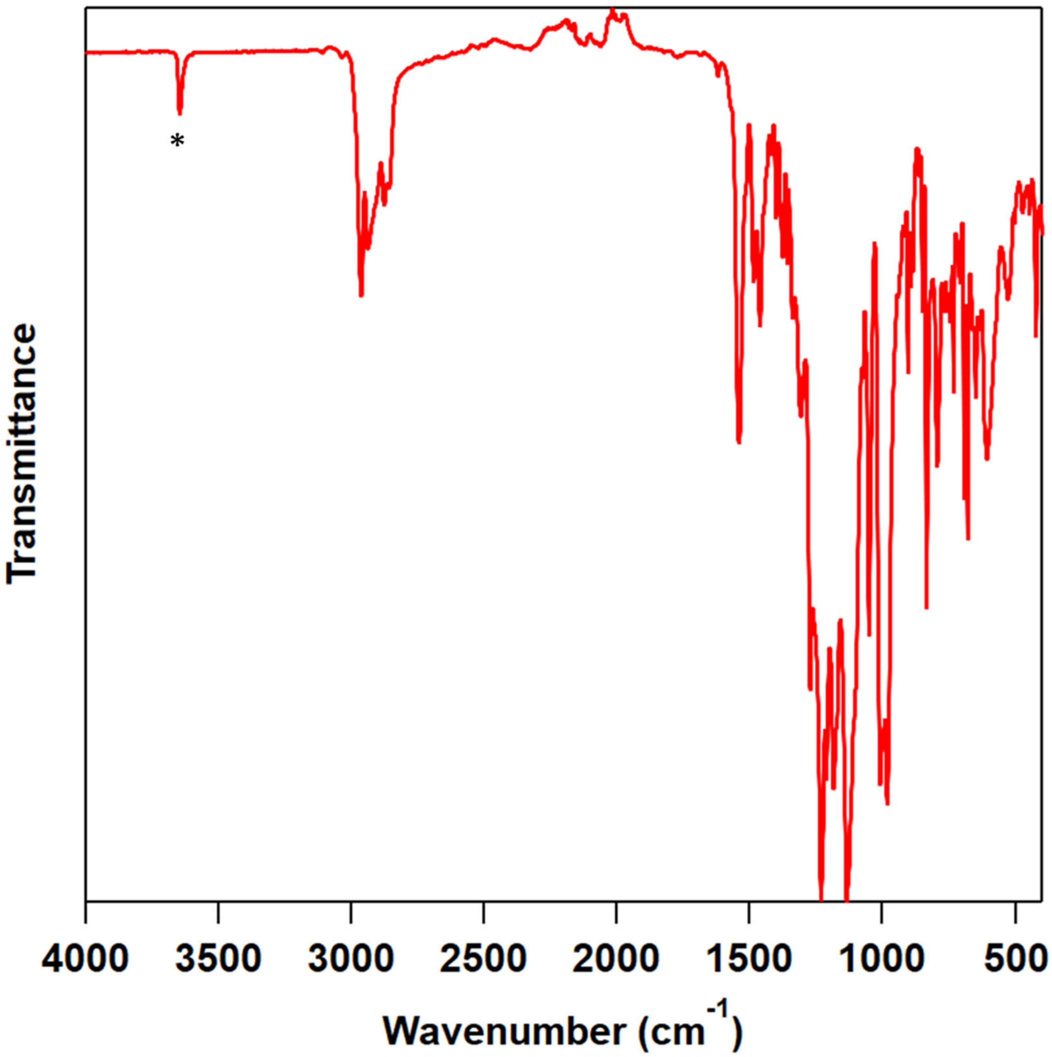

Figure S70. IR spectrum of (^{Em}L)Fe(OH)$_2$. O–H vibration mode (υ(O–H) = 3646 cm^{-1}) is denoted with asterisks (*).

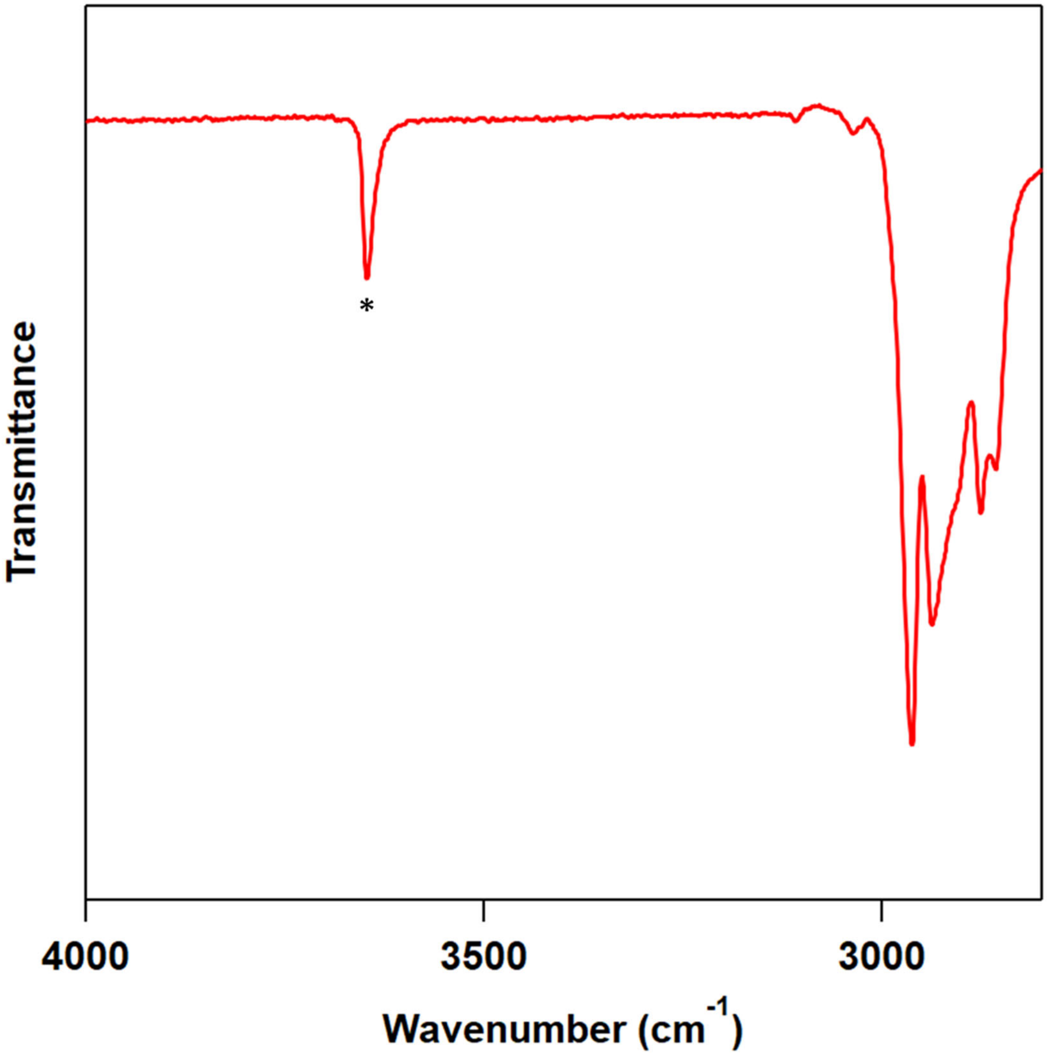

Figure S71. IR spectrum of (^{Em}L)Fe(OH)$_2$ highlighting O–H vibration. O–H vibration mode (υ(O–H) = 3646 cm^{-1}) is denoted with asterisks (*).

Reaction of (^{Em}L)Fe(C_2H_5) towards CO_2

We observed CO_2 insertion into ferrous methide, (^{Em}L)Fe(CH_3), generating ferrous acetato at elevated temperature. To investigate the effect of β-hydride on ferrous alkyl species on a reaction with CO_2, we monitored the reaction of (^{Em}L)Fe(C_2H_5) under CO_2 atmosphere.

In the drybox, a solution of (^{Em}L)Fe(C_2H_5) (4.0 mg, 0.0037 mmol) and $PhCF_3$ (0.20 µL, 0.0016 mmol) in 0.7 mL C_6D_6 were transferred to a J-Young NMR tube. The tube was sealed, degassed by three freeze-pump-thaw cycle, and CO_2 gas was introduced into the headspace (840 torr, 2.0 mL, 0.090 mmol) at room temperature. After heated to 100 °C for 24 hours, the reaction was monitored by ^{19}F and ^{1}H NMR spectroscopy (Figures S69–70). Interestingly, two paramagnetic species, (^{Em}L)Fe(O_2CH) (**10**) and (^{Em}L)Fe($O_2CC_2H_5$) (**13**) were observed in 40% and 53% spectroscopic yield. Two compounds are CO_2 insertion product to (^{Em}L)Fe(H) and (^{Em}L)Fe(C_2H_5). The generation of **13** implies the existence of competing β-hydride elimination pathway with CO_2 insertion to the ferrous alkyl complex (Figure S106).

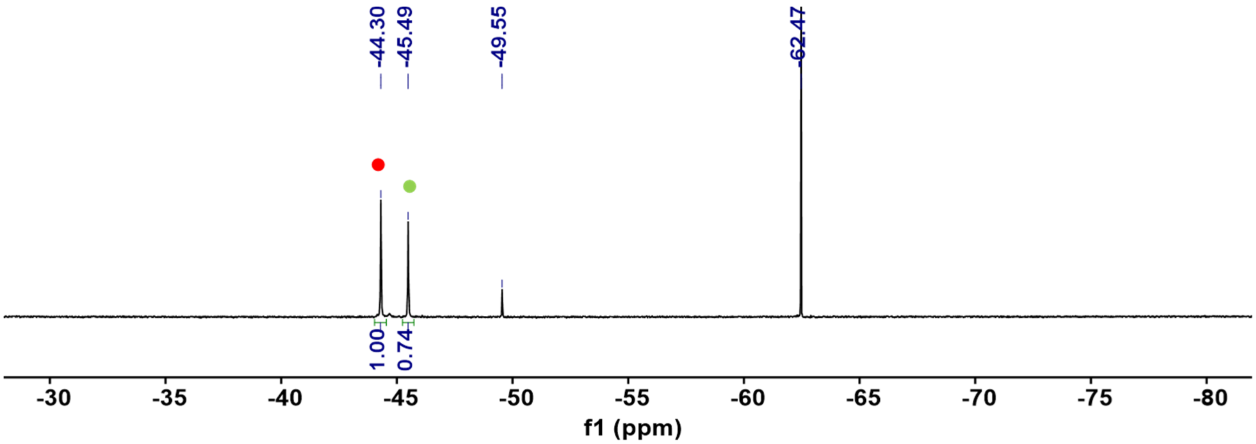

Figure S72. ^{19}F NMR spectrum of the reaction of (^{Em}L)Fe(C_2H_5) with CO_2 at 100 °C for 24 hours (471 MHz, 298 K, C_6D_6). The Red and green dots represent (^{Em}L)Fe(O_2CH) (**10**) and (^{Em}L)Fe($O_2CC_2H_5$) (**13**), respectively. The resonance at −62.47 ppm is from the added internal standard of $PhCF_3$.

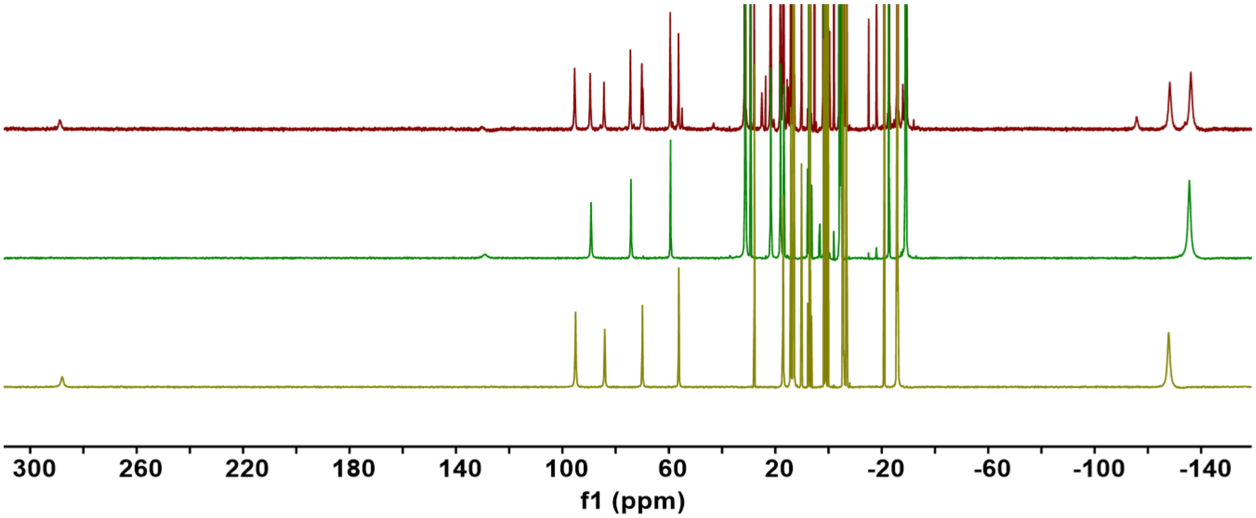

Figure S73. Stacked ^{1}H NMR spectra of the reaction of (^{Em}L)Fe(C_2H_5) with CO_2 at 100 °C for 24 hours (*maroon*, top), (^{Em}L)Fe(O_2CH) (*green*, middle), and (^{Em}L)Fe($O_2CC_2H_5$) (*golden*, bottom), (500 MHz, 298 K, C_6D_6).

X-ray diffraction techniques

Structures of **2–7**, **9–10**, **12–14**, **16** and **SI1** were collected on a Bruker three-circle platform goniometer equipped with an Apex II CCD and an Oxford cryostream cooling device. The incident radiation was from a graphite fine focus sealed tube Mo Kα (0.7107 Å) or Cu Kα (1.5418 Å) source. Crystals were mounted on a cryoloop or glass fiber pin using Paratone N oil, Fomblin Y LVAC 06/6, or Fluorolube. Structures were collected at 100 K. Data were collected as a series of φ and/or ω scans.

Data were integrated using SAINT[14] and scaled with either a numerical or multi-scan absorption correction using SADABS.[15] The structures were solved by intrinsic phasing, direct methods or Patterson maps using SHELXL-2014[16] and SHELXT-2014[17] refined against F^2 on all data by full matrix least squares with SHELXL-2019/2 with the OLEX2[18] interface. All non-hydrogen atoms were refined anisotropically. Hydrogen atoms on C atoms (except FeO_2C–*H* of **12**) were placed at idealized positions and refined using a riding model unless otherwise stated. The isotropic displacement parameters of all hydrogen atoms were constrained to be 1.2 times the parameter of the atoms they were linked to (1.5 times for methyl groups). Further details on each structure are noted below. The restraints on bond lengths and constraints of the atomic displacement parameters on each pair of disorder fragments (SADI/SAME and EADP instructions of SHELXL-2019/2), as well as the restraints of the atomic displacement parameters (SIMU/RIGU instructions of SHELXL-2019/2) if necessary, have been applied for the disorder refinement.[19] Further details on particular structures are noted below. Please refer to the cif files for exact details of disorder refinement on each structure.

(^{Em}L)Fe(NHC(O)Ph) (2): The structure was solved in the triclinic space group $P\bar{1}$ with two molecules of **1** and four molecules of diethyl ether solvent per unit cell and one molecule of **1** and two molecules of diethyl ether solvent in the asymmetric unit. The H atoms located on N3 (FeN*H*C(O)) were identified in the Fourier difference map. Positional disorder of the dipyrrin ligand (C2–C8, C10–C17, F1–F6), and diethyl ether solvent (C11S–C22S, O11S–O13S) were observed, which were modelled using similarity constraints and restraints. (CCDC 2485261)

(^{Em}L)Fe(O_2CN(H)Ad) (3): The structure was solved in the monoclinic space group $P2_1/c$ with four molecules of **3** and one in the asymmetric unit. Positional and rotational disorder of whole molecule were observed, which were modelled using similarity constraints and restraints. A disordered solvent molecule was unable to be modeled and was squeezed out by using PLATON/SQUEEZE.[20] (CCDC 2485263)

(^{Em}L)FeSH (4): The structure was solved in the triclinic space group $P\bar{1}$ with two molecules of **4** along with two *n*-pentane solvent molecules per unit cell and one of each in the asymmetric unit. The H atoms located on S1 (FeS–*H*) were identified in the Fourier difference map. Positional and rotational disorder of the trifluoromethyl (C17, F4–F6) fragment and within the hydrindacene motif (C26, C38–C41, C62–C62) were observed, which were modelled using similarity constraints and restraints. (CCDC 2485262)

(^{Em}L)Fe(HCO_3) (5): The structure was solved in the triclinic space group $P\bar{1}$ with two molecules of **5** along with two *n*-pentane solvent molecules per unit cell and one of each in the asymmetric

unit. The H atoms located on O1A (FeO–*H*) and O3 (FeO_2CO–*H*) were identified in the Fourier difference map. Positional and rotational disorder of the trifluoromethyl (C17, F4–F6) fragment and within the hydrindacene motif (C20–C22, C30–C31, C34–C39, C50–C52, C56–C57, C62–C65) were observed, which were modelled using similarity constraints and restraints. The detailed refinements are described in a "Single Crystal to Single Crystal" section below. (CCDC 2485255-2485260)

(^{Em}L)Fe(F) (6): The structure was solved in the triclinic space group $P\bar{1}$ with two molecules of **6** and two molecules of *n*-pentane solvent per unit cell and one of each molecule in the asymmetric unit. Rotational disorder of trifluoromethyl (C17, F4–F6) fragment, and positional disorder of *n*-pentane molecules (C1S–C5S) were observed, which were modelled using similarity constraints and restraints. (CCDC 2485266)

(^{Em}L)Fe($OSiEt_3$) (7): The structure was solved in the monoclinic space group $C2/c$ with eight molecules of **7** along with eight toluene and four *n*-pentane solvent molecules per unit cell and one molecule of **7** along with one toluene and 0.5 *n*-pentane solvent molecules in the asymmetric unit. Positional and rotational disorder of the trifluoromethyl (C16–C17, F1–F6) fragment and within the triethylsilyl (C71–C76) were observed, which were modelled using similarity constraints and restraints. 0.5 toluene solvent molecule located at the inversion center (special position) was modelled using similarity constraints and restraints in PART –1. (CCDC 2485264)

(^{Em}L)Fe(C_2H_5) (9): The structure was solved in the triclinic space group $P\bar{1}$ with two molecules of **9** and two molecules of *n*-pentane solvent per unit cell and one of each molecule in the asymmetric unit. Rotational disorder of trifluoromethyl (C17, F4–F6) fragment, positional disorder of hydrindacene motif (C27, C38–C41, C51, C62–C65), and iron ethyl fragment (C71–C72) were observed, which were modelled using similarity constraints and restraints. (CCDC 2485267)

(^{Em}L)Fe(O_2CH) (10): The structure was solved in the triclinic space group $P\bar{1}$ with two molecules of **10** and two molecules of *n*-pentane solvent per unit cell and one of each molecule in the asymmetric unit. The H atoms located on C71 (FeO_2C–*H*) were identified in the Fourier difference map. Rotational disorder of trifluoromethyl (C16, F1–F3) fragment, and positional disorder of hydrindacene motif (C50–C51, C62–C65) were observed, which were modelled using similarity constraints and restraints. The crystal is a non-merohedral twin, and the structure was modeled by using reflections in hkl5 format that was created by Cell_Now/Twinabs.[21] (CCDC 2485268)

(^{Em}L)Fe(O_2CCH_3) (12): The structure was solved in the triclinic space group $P\bar{1}$ with two molecules of **12** and two molecules of *n*-pentane solvent per unit cell and one of each molecule in the asymmetric unit. Positional disorder of hydrindacene motif (C62–C65) were observed, which were modelled using similarity constraints and restraints. (CCDC 2485269)

(^{Em}L)Fe(O_2CCH_3) (13): The structure was solved in the monoclinic space group $P2_1/c$ with four molecules of **13** and four molecules of *n*-pentane solvent per unit cell and one of each molecule in the asymmetric unit. Positional disorder of *n*-pentane molecule (C1S–C5S) were observed, which were modelled using similarity constraints and restraints. (CCDC 2485270)

[KC_{222}][(^{Em}L)Fe(OH)] (14): The structure was solved in the orthorhombic space group $P2_12_12_1$ with four molecules of **14** along with four diethyl ether solvent molecules per unit cell and one of each in the asymmetric unit. The H atoms located on O1 (FeO–*H*) were identified in the Fourier difference map. Positional and rotational disorder of the trifluoromethyl (C16–C17, F1–F6) fragment and within hydrindacene motif (C56–C57, C62–C65), and diethyl ether solvent molecule (O1S, C1S–C4S) were observed, which were modelled using similarity constraints and restraints. The crystal is a non-merohedral twin, and the structure was modeled by using reflections in hkl5 format that was created by Cell_Now/Twinabs.[21] (CCDC2485265)

(^{Em}L)Fe(I) (16): The structure was solved in the triclinic space group $P\bar{1}$ with two molecules of **16** and one molecule of *n*-pentane solvent per unit cell and one of each molecule in the asymmetric unit. Positional disorder of hydrindacene motif (C58, C62–C65) were observed, which were modelled using similarity constraints and restraints. (CCDC 2493268)

{(^{Em}L)Fe}$_2$(OH)$_2$(μ^2–OH)K (SI1): The structure was solved in the triclinic space group $P\bar{1}$ with two molecules of **SI1** per unit cell and one of **SI1** in the asymmetric unit. The H atoms located on O1–O3 (FeO–*H*) were identified in the Fourier difference map. Rotational and positional disorder of trifluoromethyl fragment (C16–C17, F1–F6, C81–C82, F7–F12), dipyrrin and hydrindacene motif (C66–C69, C83–C106, C83A–C99A, C200–C206) were observed, which were modelled using similarity constraints and restraints. A disordered solvent molecule was unable to be modeled and was squeezed out by using PLATON/SQUEEZE.[20] (CCDC 2485271)

Single Crystal to Single Crystal conversion between 1 and 5

X-ray diffracting quality single crystals were dispersed in Fluorolube® to maximize gas diffusion while protecting crystal from air exposure. The crystals were transferred to Schlenk flask under N_2 atmosphere, and the headspace was evacuated. CO_2 gas was introduced into the headspace at room temperature, and the crystal was placed under CO_2 atmosphere at room temperature for 1 day.

A single crystal was mounted on the goniometer quickly to minimize CO_2 release, and the diffraction data (**5_LT1**) was collected at 100 K under N_2 cryostream. The structure of **5** was solved and modelled as described above. After collecting data, the temperature of N_2 cryostream was warmed to 298 K with the ratio of 5 K/min to prevent crystal cracking. At 298 K, ω scans were conducted to achieve homogeneous irradiation over the surface and monitor the electron density change over time by the Fourier difference map (F_o–F_c(**5_LT1**)). The data sets were collected at 100 K after incubating the crystal at 298 K for 2 hours (**5_LT2**), 4 hours (**5_LT3**), 16 hours (**5_LT4**), 60 hours (**5_LT5**), and 144 hours (**5_LT6**). After incubating at 298 K, the space group did not change ($P\bar{1}$), and the unit cell parameters were changed less than 2% (Table S2). Over the course of CO_2 releasing, the electron density at bicarbonate (O1–O3, C71, H3) decreased. The gas released product was modeled by locating all non-H atoms of the product in the electron density difference map (F_o*(irradiated)* – F_c*(initial,* ***5_LT1****)*), and converged to **1** after 144 hours (**5_LT6**). The change of electron density after gas releasing was as follows (*i*) appearance of (^{Em}L)Fe(OH), and (*ii*) rotation of ethyl group within hydrindacene motif of **5**. The conversion ratio

of **5** to **1** was treated as a variable in the refinements. (CCDC deposition number for **5_LT1** – **5_LT6**: 2485255 – and 2485260)

Table S2. Unit cell parameters of **5** throughout the *in-crystallo* conversion to **1**.

**Incubating time (hours)**	**Unit cell parameters**						
	a **(Å)**	***b*** **(Å)**	***c*** **(Å)**	***α*** **(°)**	***β*** **(°)**	***γ*** **(°)**	***V*** **($Å^3$)**
0	10.4755(4)	13.8067(5)	22.3894(8)	82.6564(13)	89.1725(13)	82.2681(13)	3182.5(2)
2	10.4685(4)	13.7920(5)	22.3737(7)	82.6124(12)	89.2675(12)	82.2444(12)	3174.20(19)
4	10.4636(4)	13.7863(5)	22.3647(8)	82.5889(13)	89.2954(13)	82.2145(13)	3169.7(2)
16	10.4479(4)	13.7679(5)	22.3323(8)	82.5470(13)	89.4133(14)	82.0358(14)	3154.4(2)
64	10.4295(5)	13.7384(6)	22.2893(10)	82.5239(15)	89.5048(16)	81.7745(16)	3133.8(2)
144	10.4070(12)	13.8012(15)	22.008(2)	85.013(3)	93.150(4)	82.778(4)	3117.1(6)

Table S3. Conversion ratio of **5** to **1** as determined by the occupancy of O1A.

**Irradiation time (hours)**	**Conversion (%)**
0	14.7(4)
2	27.4(4)
4	34.2(4)
16	56.1(3)
64	79.8(3)
144	100

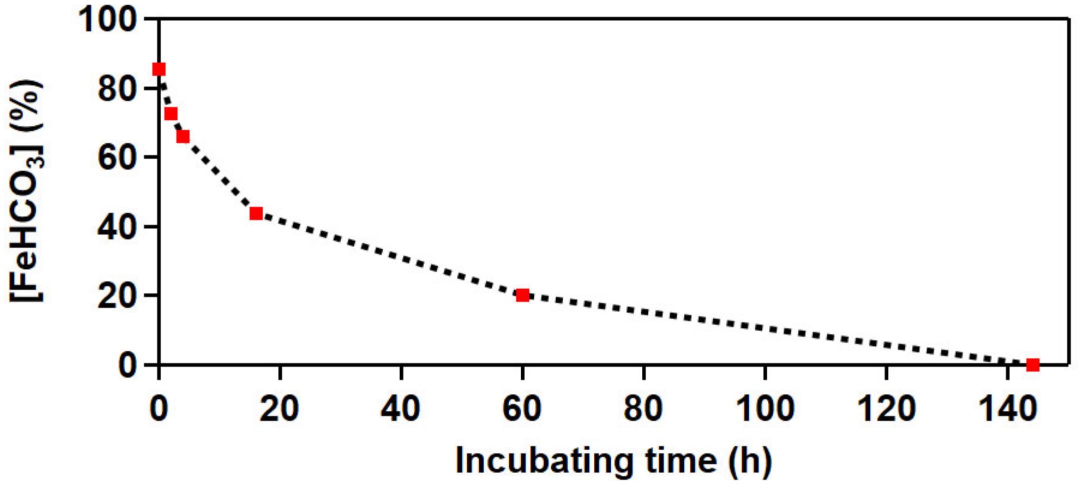

Figure S74. Single crystal conversion ratio of **5** to **1**.

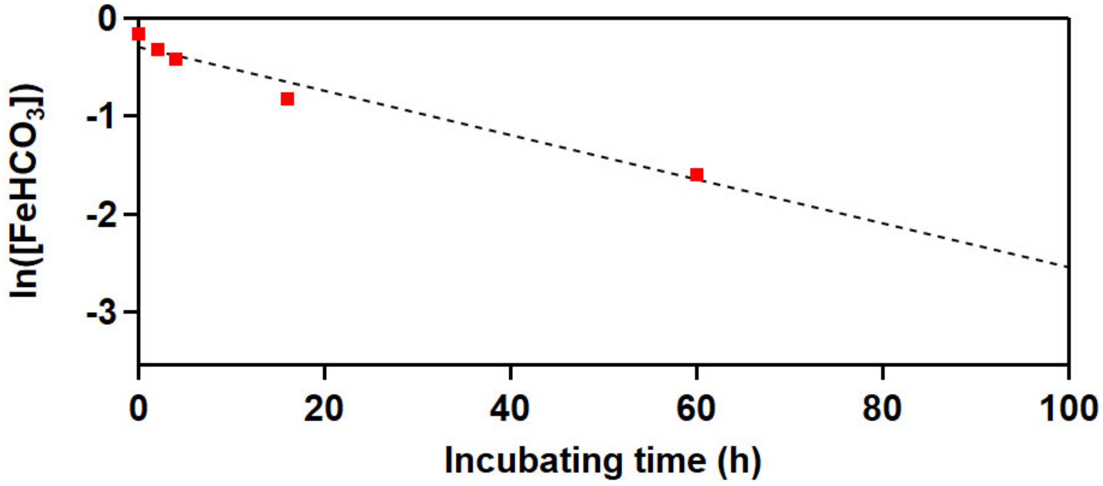

Figure S75. Single crystal conversion ratio of **5** to **1**. Logarithmic of [**5**] and time shows linear relationship ($R^2 = 0.962$), which suggests first order kinetics of CO_2 releasing from a solid-state **5**.

Table S4. X-ray diffraction experimental details

	(^{Em}L)Fe(NHC(O)Ph) (2)	(^{Em}L)Fe(O$_2$CNHAd) (3)	(^{Em}L)FeSH (4)	(^{Em}L)Fe(HCO$_3$) (5-LT1)	(^{Em}L)Fe(HCO$_3$) (5-LT2)
**Moiety Formula**	$C_{80}H_{107}F_6FeN_3O_3$	$C_{76}H_{97}F_6FeN_3O_2$	$C_{70}H_{94}F_6FeN_2S$	$C_{70.85}H_{94}F_6FeN_2O_{2.71}$	$C_{70.72}H_{94}F_6FeN_2O_{2.45}$
**CCDC Deposit**	2485261	2485263	2485262	2485255	2485256
**FW**	1328.53	1254.41	1165.38	1186.86	1181.22
**λ (Å)**	0.71073	0.71073	0.71073	0.71073	0.71073
**T (K)**	100	100	100	100	100
**Crystal System**	Triclinic	Monoclinic	Triclinic	Triclinic	Triclinic
**Space Group**	$P\bar{1}$ (2)	$P2_1/c$ (14)	$P\bar{1}$ (2)	$P\bar{1}$ (2)	$P\bar{1}$ (2)
**a (Å)**	10.8139(9)	10.6004(10)	10.490(3)	10.4755(4)	10.4685(4)
**b (Å)**	14.6223(11)	28.413(3)	13.838(3)	13.8067(5)	13.7920(5)
**c (Å)**	23.5735(18)	26.282(2)	22.334(5)	22.3894(8)	22.3737(7)
**α (°)**	94.169(3)	90	82.671(5)	82.6564(13)	82.6124(12)
**β (°)**	94.221(3)	100.038(3)	89.717(5)	89.1725(13)	89.2675(12)
**γ (°)**	92.774(3)	90	82.450(5)	82.2681(13)	82.2444(12)
**Volume (Å^3)**	3702.2(5)	7794.6(12)	3187.4(13)	3182.5(2)	3174.20(19)
**Z**	2	4	2	2	2
**Calc. ρ (mg/mm^3)**	1.192	1.069	1.214	1.239	1.236
**μ (mm^{-1})**	0.27	0.25	0.33	0.3	0.3
**Crystal Size (mm)**	0.16 × 0.12 × 0.08	0.14 × 0.12 × 0.06	0.18 × 0.16 × 0.10	0.20 × 0.16 × 0.15	0.20 × 0.16 × 0.15
**Reflections**	13033	13761	11288	11278	11285
**Completeness (to 2θ)**	0.994	0.993	0.996	0.996	0.996
**GOF on F^2**	1.06	1.04	1.03	1.09	1.06
**R$_1$, wR$_2$^{a} [$I > 2\sigma(I)$]**	0.087, 0.256	0.104, 0.298	0.057, 0.158	0.045, 0.102	0.046, 0.103

^{a} $1 = \sum\left||F_o| - |F_c|\right|/\sum|F_o|$, $wR2 = \{\sum[w(F_o^2 - F_c^2)^2]/\sum[w(F_o^2)^2]\}^{1/2}$

	$(^{Em}L)Fe(HCO_3)$ (5-LT3)	$(^{Em}L)Fe(HCO_3)$ (5-LT4)	$(^{Em}L)Fe(HCO_3)$ (5-LT5)	$(^{Em}L)Fe(HCO_3)$ (5-LT6)	$(^{Em}L)Fe(F)$ (6)
**Moiety Formula**	$C_{70.66}H_{94}F_6FeN_2O_{2.32}$	$C_{70.44}H_{94}F_6FeN_2O_{1.88}$	$C_{70.20}H_{94}F_6FeN_2O_{1.40}$	$C_{70}H_{94}F_6FeN_2O$	$C_{70}H_{93}F_{7.01}FeN_2$
**CCDC deposit**	2485257	2485258	2485259	2485260	2485266
**FW**	1178.28	1168.6	1158.2	1149.32	1151.4
**λ (Å)**	0.71073	0.71073	0.71073	0.71073	0.71073
**T (K)**	100	100	100	100	100
**Crystal System**	Triclinic	Triclinic	Triclinic	Triclinic	Triclinic
**Space Group**	$P\bar{1}$ (2)	$P\bar{1}$ (2)	$P\bar{1}$ (2)	$P\bar{1}$ (2)	$P\bar{1}$ (2)
**a (Å)**	10.4636(4)	10.4479(4)	10.4295(5)	10.4070(12)	10.4274(3)
**b (Å)**	13.7863(5)	13.7679(5)	13.7384(6)	13.8012(15)	13.8284(4)
**c (Å)**	22.3647(8)	22.3323(8)	22.2893(10)	22.008(2)	22.0395(7)
**α (°)**	82.5889(13)	82.5470(13)	82.5239(15)	85.013(3)	94.7255(11)
**β (°)**	89.2954(13)	89.4133(14)	89.5048(16)	93.150(4)	93.5144(11)
**γ (°)**	82.2145(13)	82.0358(14)	81.7745(16)	82.778(4)	97.3318(11)
**Volume (Å^3)**	3169.7(2)	3154.4(2)	3133.8(2)	3117.1(6)	3132.97(16)
**Z**	2	2	2	2	2
**Calc. ρ (mg/mm^3)**	1.235	1.23	1.227	1.225	1.221
**μ (mm^{-1})**	0.3	0.3	0.3	0.3	0.3
**Crystal Size (mm)**	0.20 × 0.16 × 0.15	0.20 × 0.16 × 0.15	0.20 × 0.16 × 0.15	0.20 × 0.16 × 0.15	0.26 × 0.18 × 0.12
**Reflections**	11276	11191	11141	11121	11081
**Completeness (to 2θ)**	0.996	0.998	0.997	0.99	0.998
**GOF on F^2**	1.06	1.06	1.05	1.15	1.05
**R_1, wR_2[a] [$I > 2\sigma(I)$]**	0.046, 0.105	0.05, 0.116	0.049, 0.119	0.091, 0.237	0.06, 0.182

[a] $1 = \sum \left| |F_o| - |F_c| \right| / \sum |F_o|$, $wR2 = \{\sum[w(F_o^2 - F_c^2)^2]/\sum[w(F_o^2)^2]\}^{1/2}$

	$(^{Em}L)Fe(OSiEt_3)$ **(7)**	$(^{Em}L)Fe(C_2H_5)$ **(9)**	$(^{Em}L)Fe(O_2CH)$ **(10)**	$(^{Em}L)Fe(O_2CCH_3)$ **(12)**	$(^{Em}L)Fe(O_2CCH_2CH_3)$ **(13)**
**Moiety Formula**	$C_{78.75}H_{108}F_6FeN_2OSi$	$C_{72}H_{98}F_6FeN_2$	$C_{71}H_{94}F_6FeN_2O_2$	$C_{72}H_{96}F_6FeN_2O_2$	$C_{73}H_{98}F_6FeN_2O_2$
**CCDC deposit**	2485264	2485267	2485268	2485269	2485270
**FW**	1296.6	1161.37	1177.33	1191.35	1205.38
**λ (Å)**	0.71073	0.71073	0.71073	0.71073	0.71073
**T (K)**	100	100	100	100	100
**Crystal System**	Monoclinic	Triclinic	Triclinic	Triclinic	Monoclinic
**Space Group**	$C2/c$ (15)	$P\bar{1}$ (2)	$P\bar{1}$ (2)	$P\bar{1}$ (2)	$P2_1/c$ (14)
**a (Å)**	45.8100(15)	10.5242(7)	10.482(4)	10.4797(17)	10.5213(12)
**b (Å)**	15.2921(5)	13.9515(8)	13.802(5)	13.903(2)	21.045(2)
**c (Å)**	20.8657(6)	22.3358(13)	22.312(8)	22.552(3)	29.428(3)
**α (°)**	90	83.323(2)	82.542(12)	82.445(5)	90
**β (°)**	93.8331(17)	89.855(2)	89.493(13)	89.239(5)	92.363(2)
**γ (°)**	90	81.448(2)	82.572(15)	81.834(5)	90
**Volume ($Å^3$)**	14584.4(8)	3220.7(3)	3173.5(19)	3224.2(9)	6510.3(13)
**Z**	8	2	2	2	4
**Calc. ρ (mg/mm^3)**	1.181	1.198	1.232	1.227	1.23
**μ (mm^{-1})**	0.28	0.29	0.3	0.3	0.3
**Crystal Size (mm)**	0.22 × 0.18 × 0.10	0.34 × 0.16 × 0.14	0.18 × 0.16 × 0.12	0.18 × 0.16 × 0.14	0.18 × 0.16 × 0.12
**Reflections**	12889	11449	11296	11442	11606
**Completeness (to 2θ)**	0.999	0.997	0.995	0.994	0.996
**GOF on F^2**	1.05	1.04	1.11	1.04	1.14
**R_1, wR_2[a] [$I > 2\sigma(I)$]**	0.044, 0.104	0.042, 0.100	0.073, 0.182	0.055, 0.131	0.056, 0.142

[a] $1 = \sum||F_o| - |F_c|| / \sum|F_o|$, $wR2 = \{\sum[w(F_o^2 - F_c^2)^2] / \sum[w(F_o^2)^2]\}^{1/2}$

	$[(^{Em}L)Fe(OH)][KC_{222}]$ (14)	$(^{Em}L)Fe(I)$ (16)	$\{(^{Em}L)Fe\}_2(OH)_2(\mu^2–OH)(K)$ (SI1)
**Moiety Formula**	$C_{87}H_{128}F_6FeKN_4O_8$	$C_{70}H_{93}F_6FeIN_2$	$C_{130}H_{165}F_{12}Fe_2KN_4O_3$
**CCDC deposit**	2485265	2493268	2485271
**FW**	1566.88	1259.21	2210.45
**λ (Å)**	0.71073	0.71073	0.71073
**T (K)**	100	100	100
**Crystal System**	Orthorhombic	Triclinic	Triclinic
**Space Group**	$P2_12_12_1$ (19)	$P\bar{1}$ (2)	$P\bar{1}$ (2)
**a (Å)**	20.1072(11)	10.4886(5)	16.7683(5)
**b (Å)**	25.0400(14)	13.8820(6)	18.0737(5)
**c (Å)**	16.6795(10)	22.5055(9)	24.0646(7)
**α (°)**	90	83.508(2)	89.568(1)
**β (°)**	90	89.636(2)	73.784(1)
**γ (°)**	90	81.829(2)	72.999(1)
**Volume (Å^3)**	8397.9(8)	3222.6(2)	6675.1(3)
**Z**	4	2	2
**Calc. ρ (mg/mm^3)**	1.239	1.298	1.1
**μ (mm^{-1})**	0.3	0.771	0.31
**Crystal Size (mm)**	0.26 × 0.20 × 0.18	0.32 × 0.19 × 0.19	0.24 × 0.18 × 0.14
**Reflections**	14453	9831	23742
**Completeness (to 2θ)**	0.97	0.990	0.998
**GOF on F^2**	1.13	1.05	1.06
**R_1, wR_2[a] [$I > 2\sigma(I)$]**	0.079, 0.156	0.022, 0.071	0.052, 0.114

[a] $1 = \sum||F_o| - |F_c||/\sum|F_o|$, $wR2 = \{\sum[w(F_o^2 - F_c^2)^2]/\sum[w(F_o^2)^2]\}^{1/2}$

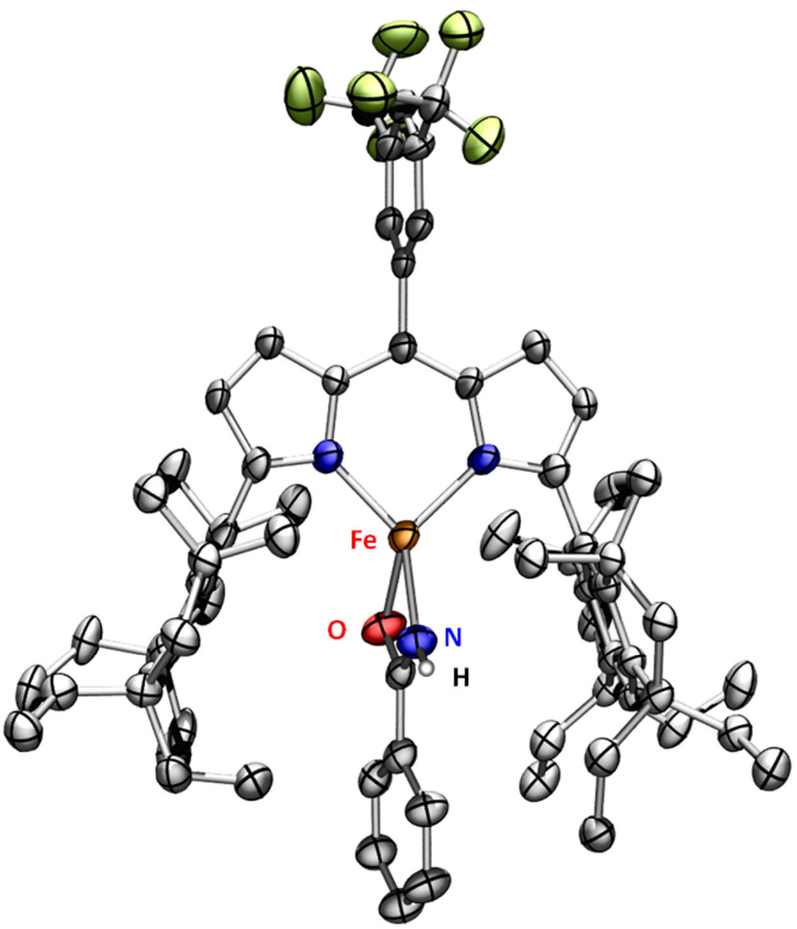

Figure S76. Solid-state structure of (^{Em}L)Fe(NHC(O)Ph) (**2**) with thermal ellipsoids at 50% probability level. Hydrogens and solvent molecules in the unit cell are omitted for clarity, except for the *H*–NC(O)Ph. Color scheme: Fe (*orange*), O (*red*), F (*yellow-green*), N (*blue*), C (*gray*), H (*white*).

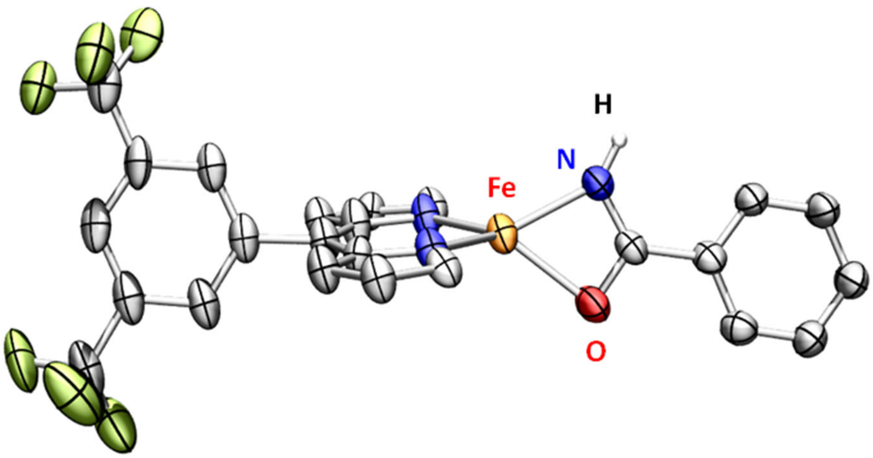

Figure S77. Truncated solid-state structure of (^{Em}L)Fe(NHC(O)Ph) (**2**) with thermal ellipsoids at 50% probability level, highlighting the connectivity of the primary coordination sphere. Hydrogens and solvent molecules in the unit cell are omitted for clarity, except for the *H*–NC(O)Ph. Color scheme: Fe (*orange*), O (*red*), F (*yellow-green*), N (*blue*), C (*gray*), H (*white*).

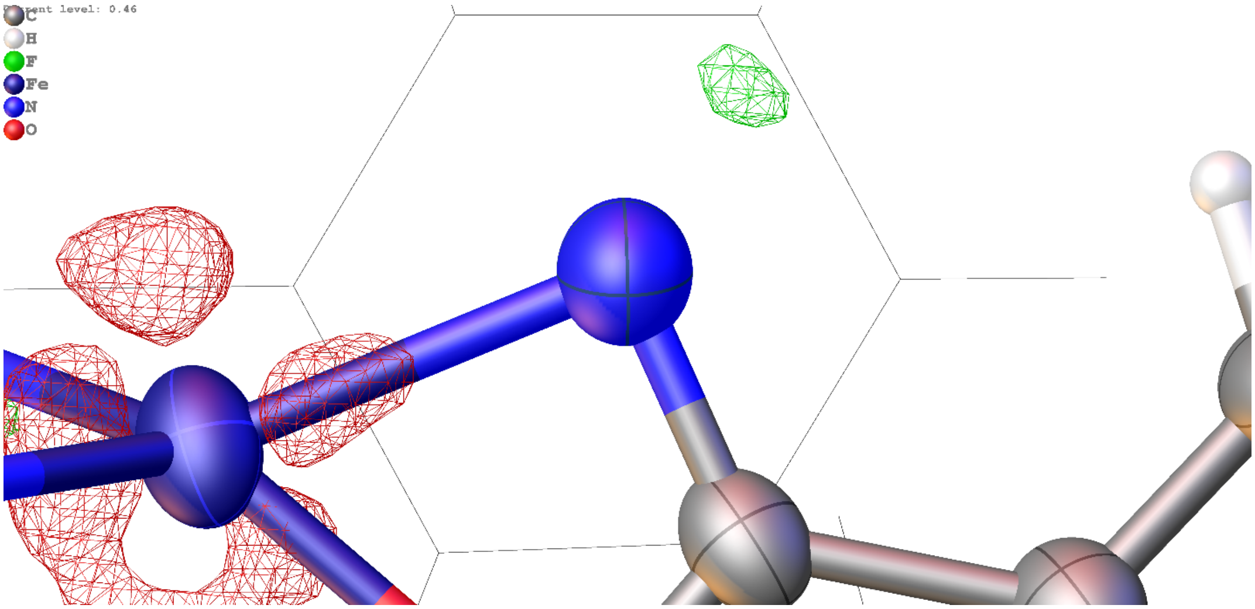

Figure S78. Fourier-difference map for (^{Em}L)Fe(NHC(O)Ph) (**2**) in which *H*–NC(O)Ph was located. The electron density map was generated at the 0.46 *e* Å^{-3} level.

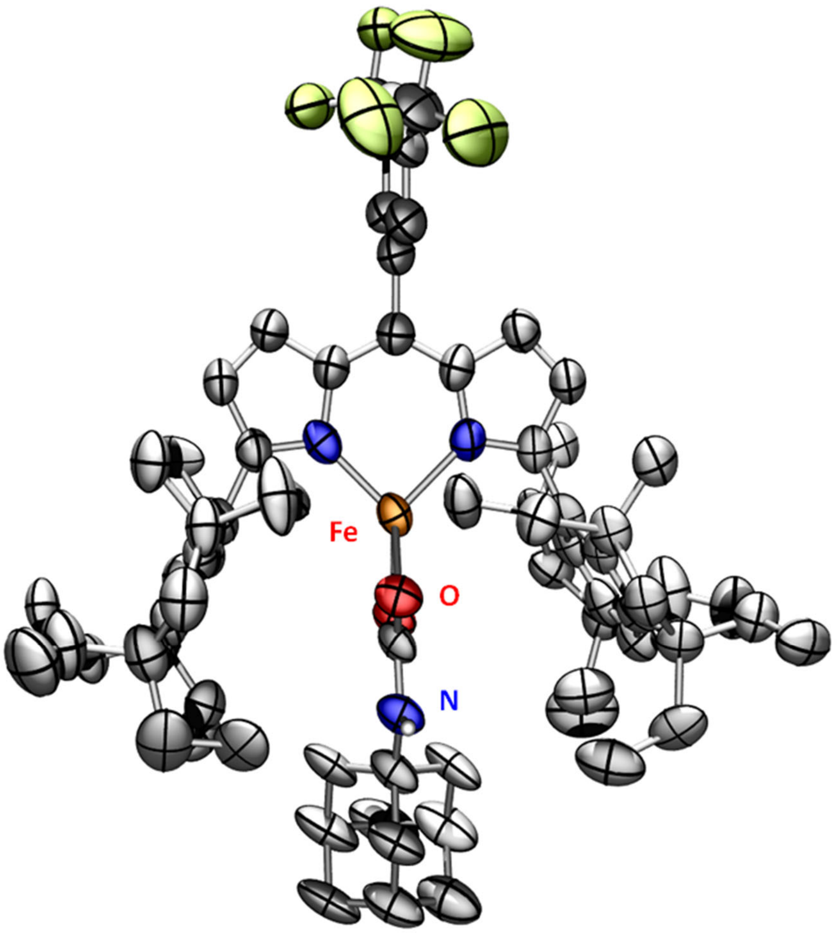

Figure S79. Solid-state structure of (^{Em}L)Fe(O_2CNHAd) (**3**) with thermal ellipsoids at 50% probability level. Hydrogens and solvent molecules in the unit cell are omitted for clarity. Color scheme: Fe (*orange*), O (*red*), F (*yellow-green*), N (*blue*), C (*gray*), H (*white*).

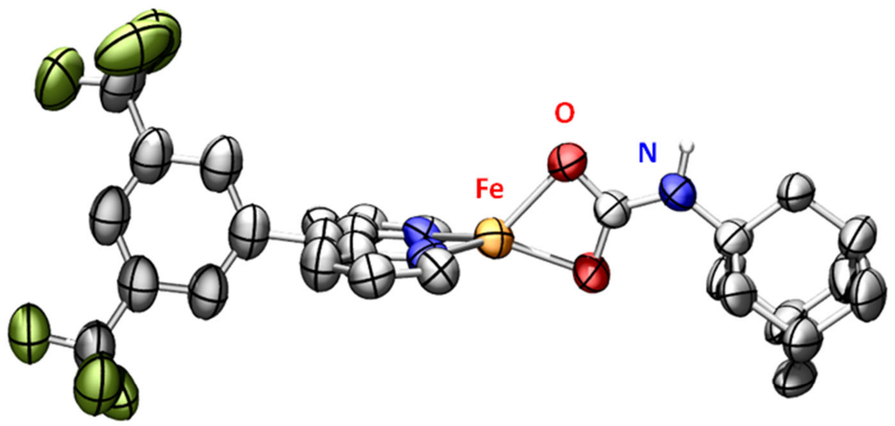

Figure S80. Truncated solid-state structure of (^{Em}L)Fe(O_2CNHAd) (**3**) with thermal ellipsoids at 50% probability level, highlighting the connectivity of the primary coordination sphere. Hydrogens and solvent molecules in the unit cell are omitted for clarity. Color scheme: Fe (*orange*), O (*red*), F (*yellow-green*), N (*blue*), C (*gray*), H (*white*).

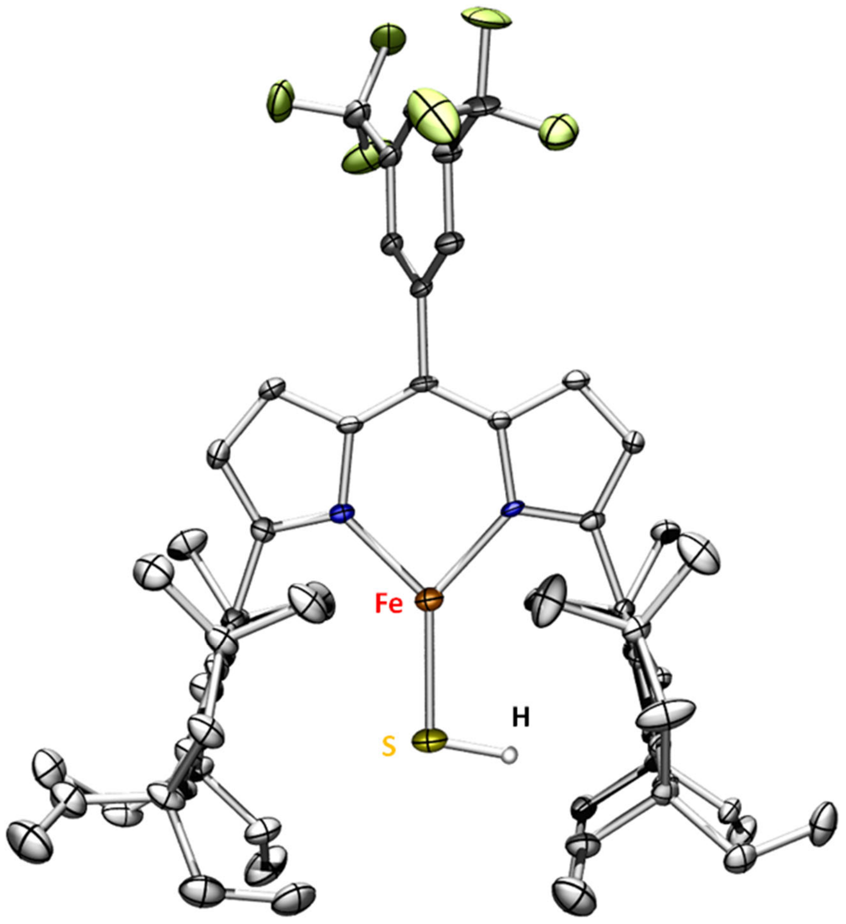

Figure S81. Solid-state structure of (^{Em}L)Fe(SH) (**4**) with thermal ellipsoids at 50% probability level. Hydrogens and solvent molecules in the unit cell are omitted for clarity, except for the FeS–*H*. Color scheme: Fe (*orange*), S (*yellow*), F (*yellow-green*), N (*blue*), C (*gray*), H (*white*).

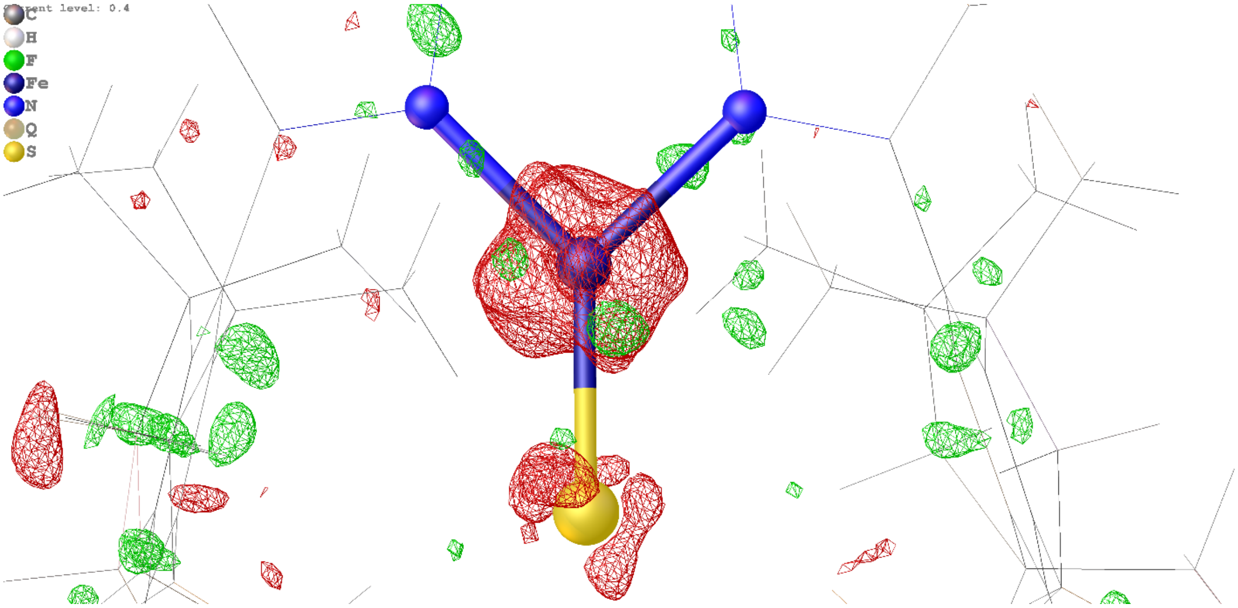

Figure S82. Fourier-difference map for (^{Em}L)Fe(SH) (**4**) in which FeS–*H* was located. The electron density map was generated at the 0.40 *e* Å^{-3} level.

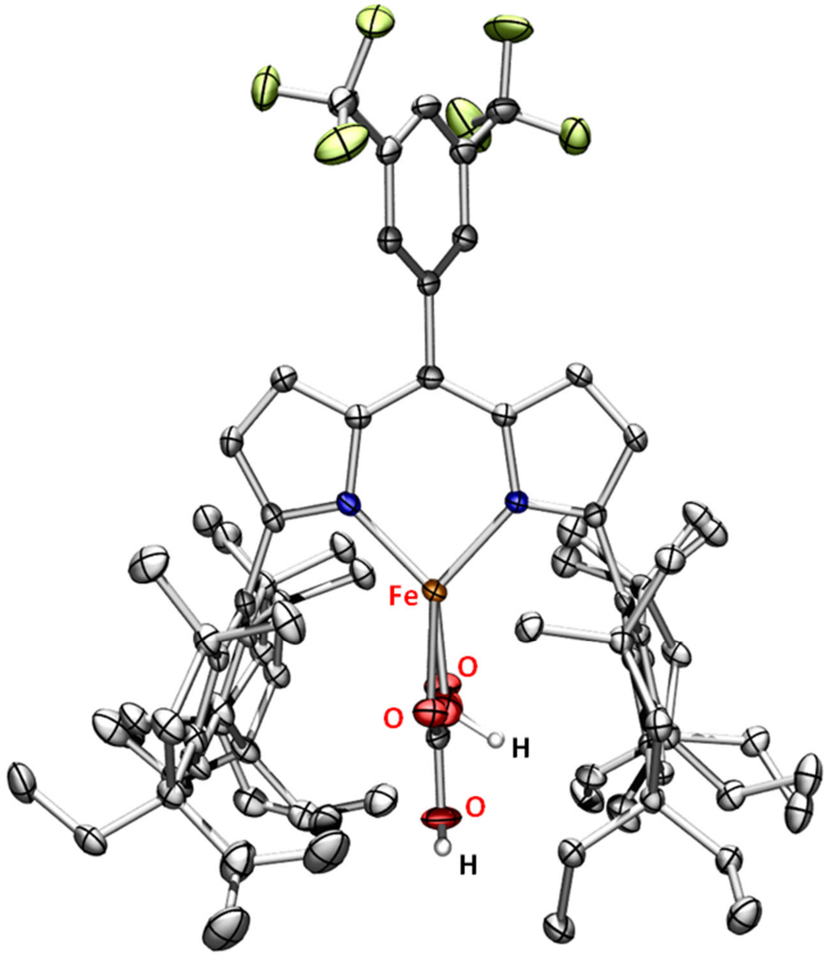

Figure S83. Solid-state structure of (^{Em}L)Fe(OH) incubated under CO_2 (**5_LT1**) with thermal ellipsoids at 50% probability level, reflecting partial consumption of (^{Em}L)Fe(OH). Hydrogens and solvent molecules in the unit cell are omitted for clarity, except FeO–*H*, and FeO_2CO–*H*. Color scheme: Fe (*orange*), O (*red*), F (*yellow-green*), N (*blue*), C (*gray*), H (*white*).

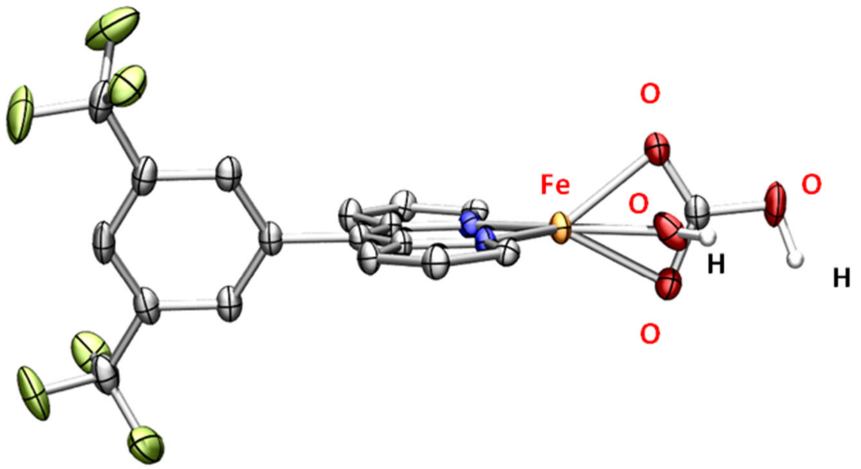

Figure S84. Truncated solid-state structure of (^{Em}L)Fe(OH) incubated under CO_2 (**5_LT1**) with thermal ellipsoids at 50% probability level, highlighting the connectivity of the primary coordination sphere. Hydrogens and solvent molecules in the unit cell are omitted for clarity, except FeO–*H*, and FeO_2CO–*H*. Color scheme: Fe (*orange*), O (*red*), F (*yellow-green*), N (*blue*), C (*gray*), H (*white*).

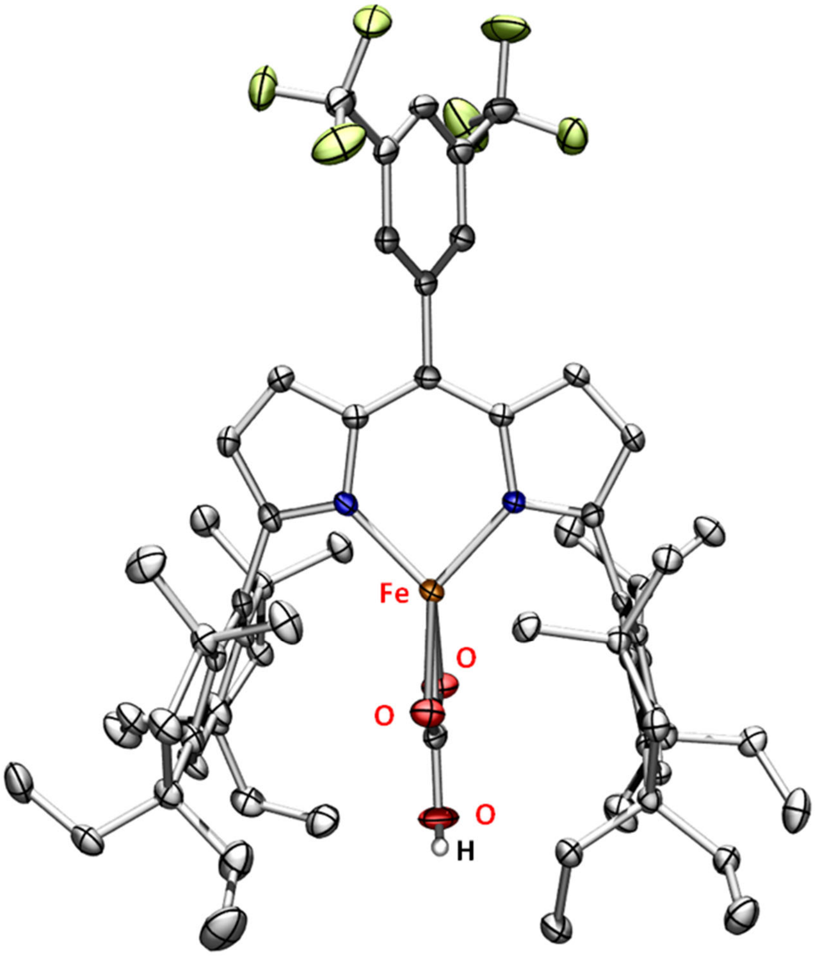

Figure S85. Solid-state structure of (^{Em}L)Fe(HCO_3) obtained from **5_LT1** with thermal ellipsoids at 50% probability level, highlighting the connectivity of the primary coordination sphere. Hydrogens and solvent molecules in the unit cell are omitted for clarity, except FeO_2CO–*H*. Color scheme: Fe (*orange*), O (*red*), F (*yellow-green*), N (*blue*), C (*gray*), H (*white*).

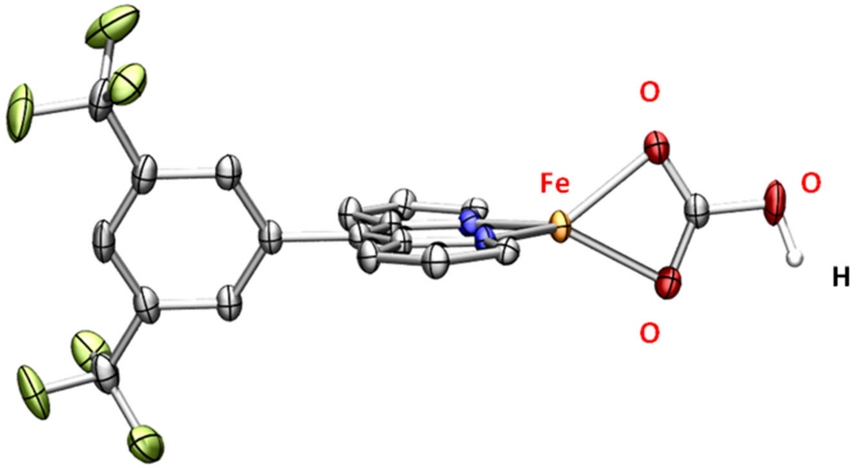

Figure S86. Truncated solid-state structure of (^{Em}L)Fe(HCO_3) obtained from **5_LT1** with thermal ellipsoids at 50% probability level, highlighting the connectivity of the primary coordination sphere. Hydrogens and solvent molecules in the unit cell are omitted for clarity, except FeO_2CO–*H*. Color scheme: Fe (*orange*), O (*red*), F (*yellow-green*), N (*blue*), C (*gray*), H (*white*).

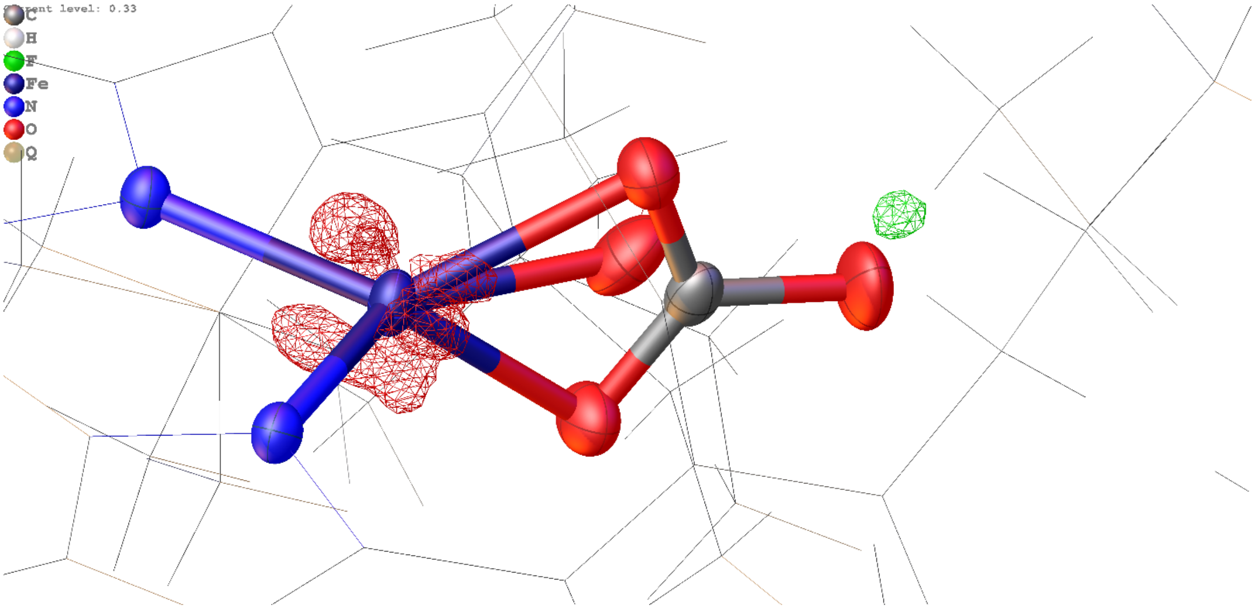

Figure S87. Fourier-difference map for **5_LT1** in which FeO_2CO–*H* was located. The electron density map was generated at the 0.33 e Å^{-3} level.

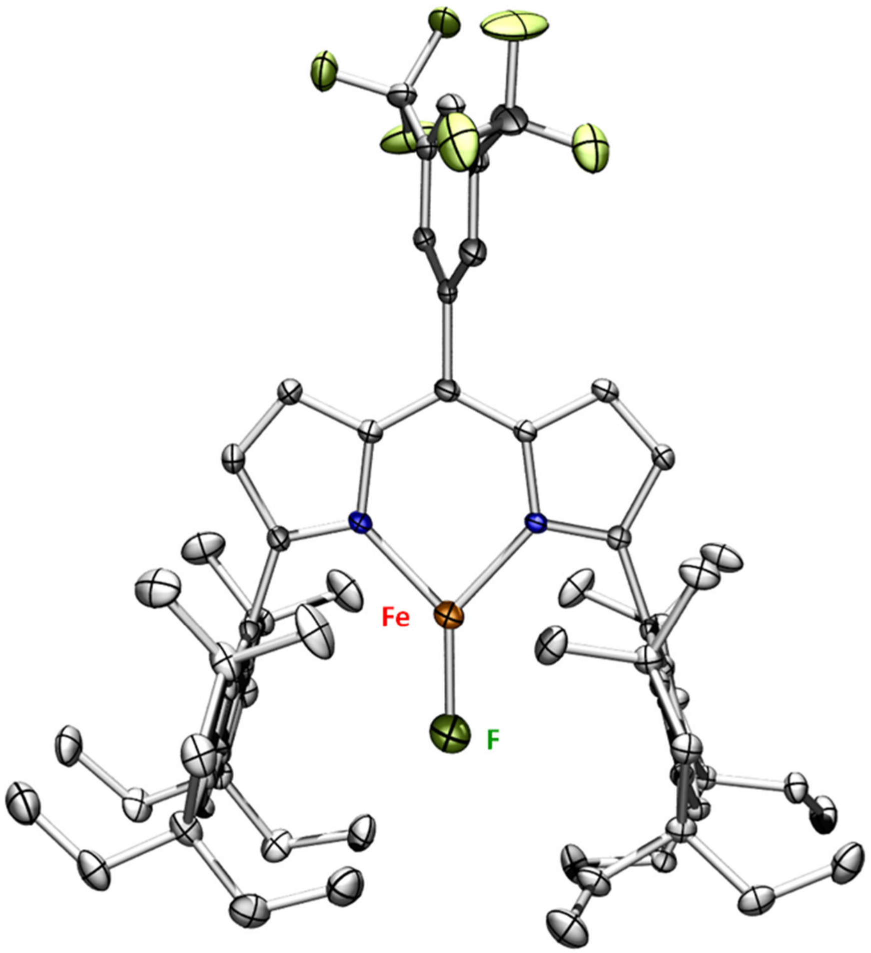

Figure S88. Solid-state structure of (^{Em}L)Fe(F) (**6**) with thermal ellipsoids at 50% probability level. Hydrogens and solvent molecules in the unit cell are omitted for clarity. Color scheme: Fe (*orange*), O (*red*), F (*yellow-green*), N (*blue*), C (*gray*), H (*white*).

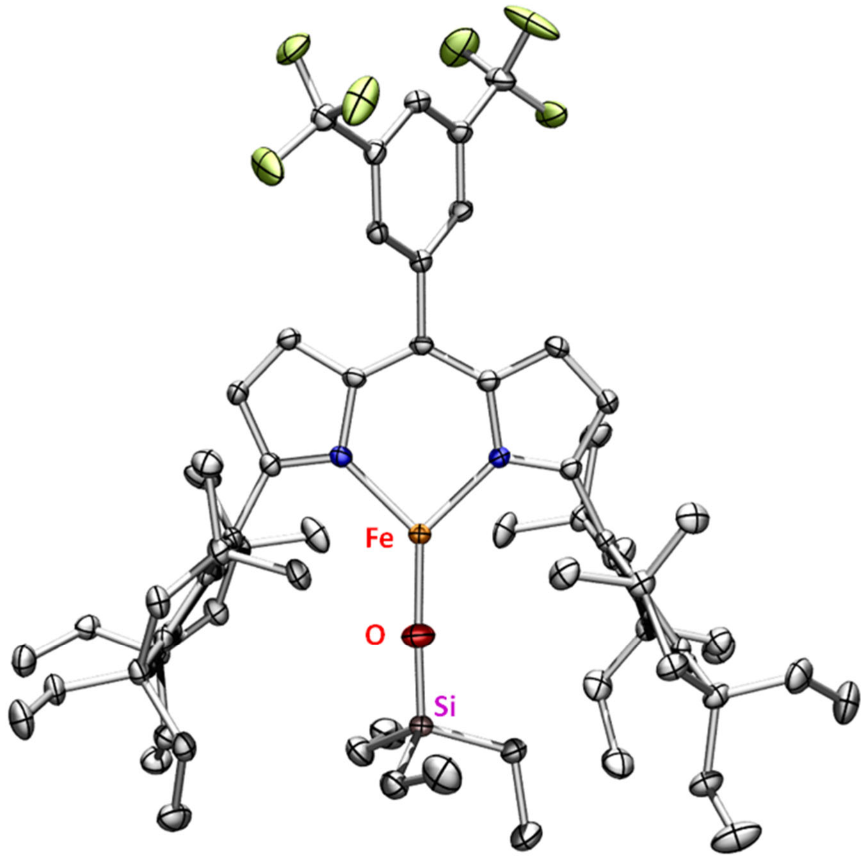

Figure S89. Solid-state structure of (^{Em}L)Fe(OSi(CH_2CH_3)$_3$) (**7**) with thermal ellipsoids at 50% probability level. Hydrogens and solvent molecules in the unit cell are omitted for clarity. Color scheme: Fe (*orange*), O (*red*), Si (*pink*), F (*yellow-green*), N (*blue*), C (*gray*), H (*white*).

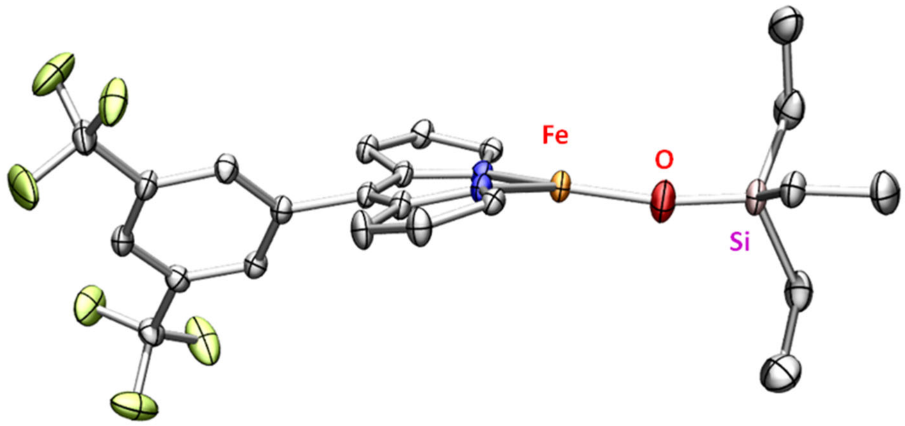

Figure S90. Truncated solid-state structure of (^{Em}L)Fe(OSi(CH_2CH_3)$_3$) (**7**) with thermal ellipsoids at 50% probability level, highlighting the connectivity of the primary coordination sphere. Hydrogens and solvent molecules in the unit cell are omitted for clarity. Color scheme: Fe (*orange*), O (*red*), Si (*pink*), F (*yellow-green*), N (*blue*), C (*gray*), H (*white*).

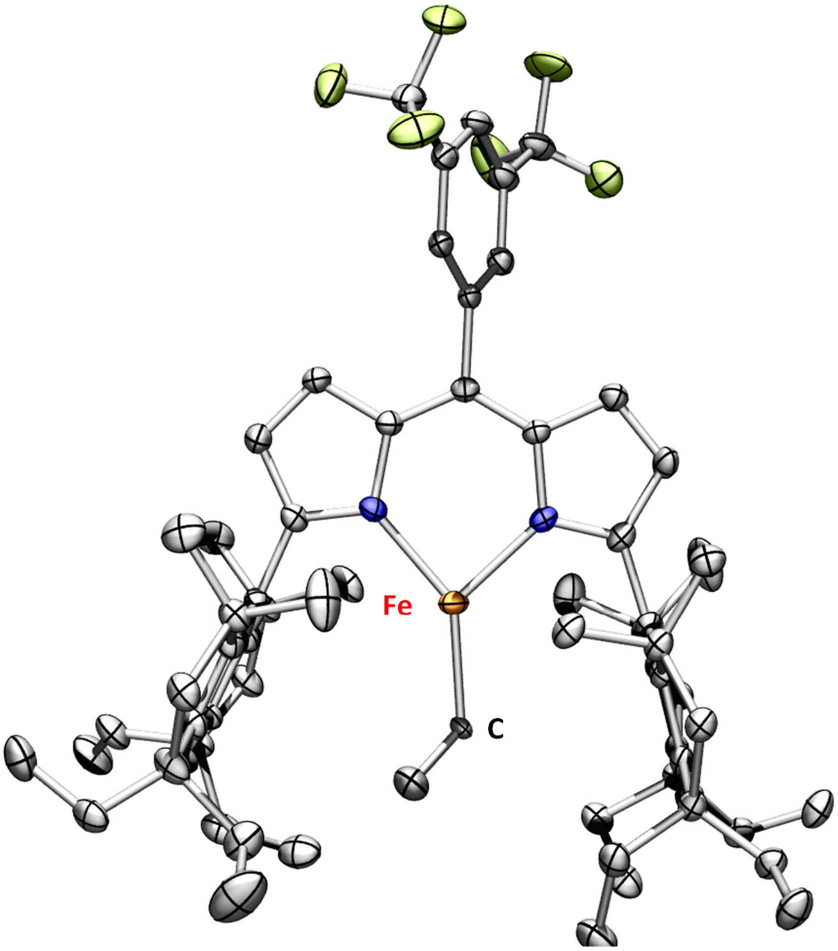

Figure S91. Solid-state structure of (^{Em}L)Fe(CH_2CH_3) (**9**) with thermal ellipsoids at 50% probability level. Hydrogens and solvent molecules in the unit cell are omitted for clarity. Color scheme: Fe (*orange*), O (*red*), F (*yellow-green*), N (*blue*), C (*gray*), H (*white*).

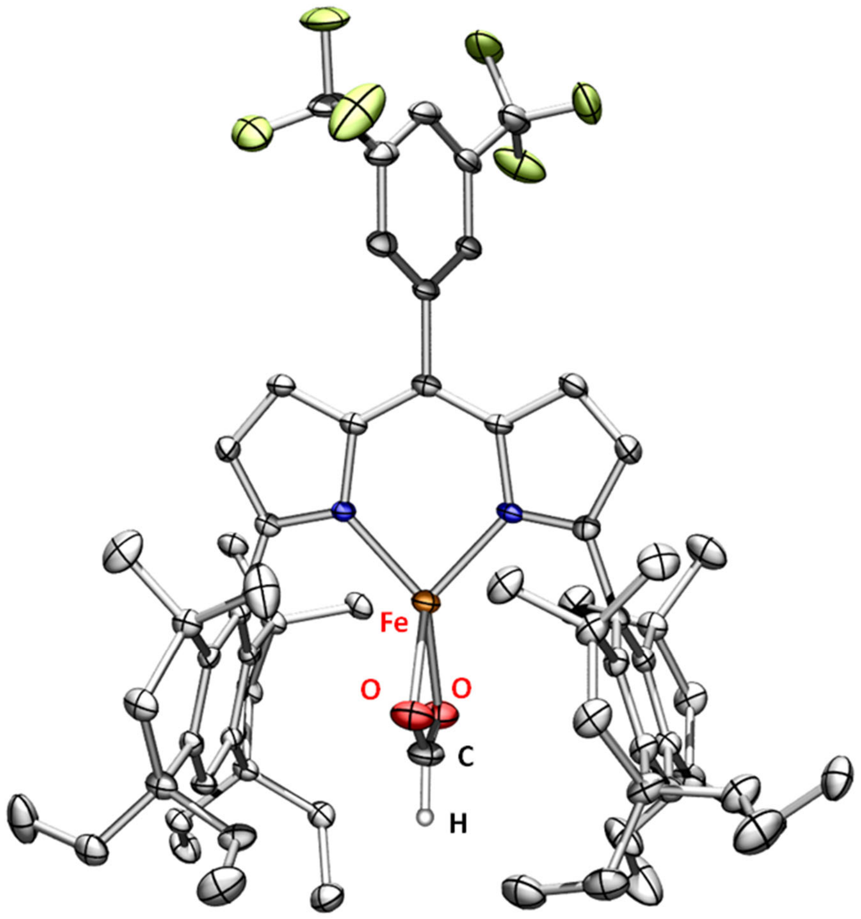

Figure S92. Solid-state structure of (^{Em}L)Fe(O_2CH) (**10**) with thermal ellipsoids at 50% probability level. Hydrogens and solvent molecules in the unit cell are omitted for clarity, except FeO_2C–*H*. Color scheme: Fe (*orange*), O (*red*), F (*yellow-green*), N (*blue*), C (*gray*), H (*white*).

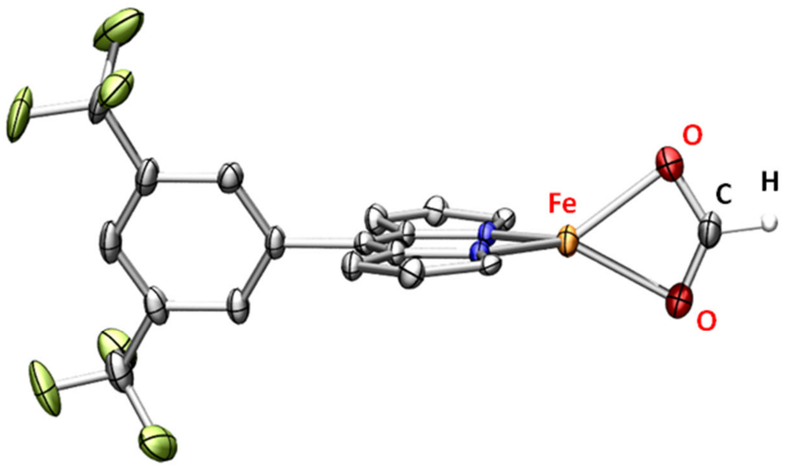

Figure S93. Truncated solid-state structure of (^{Em}L)Fe(O_2CH) (**10**) with thermal ellipsoids at 50% probability level, highlighting the connectivity of the primary coordination sphere. Hydrogens, and solvent molecules in the unit cell are omitted for clarity, except FeO_2C–*H*. Color scheme: Fe (*orange*), O (*red*), F (*yellow-green*), N (*blue*), C (*gray*), H (*white*).

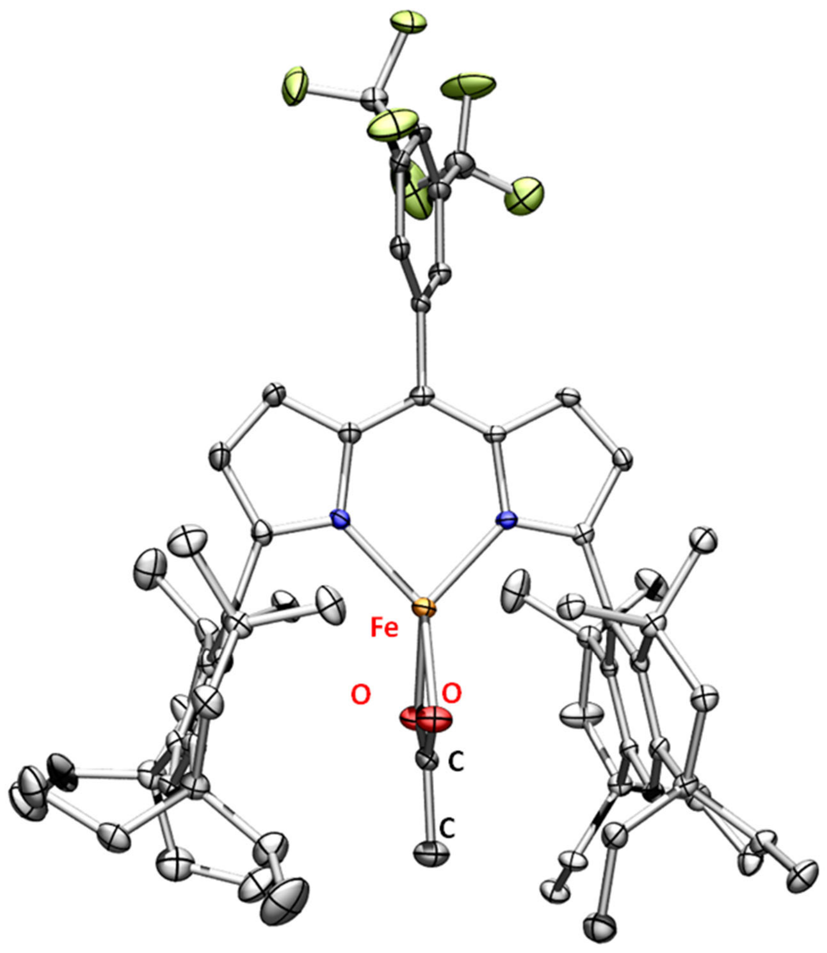

Figure S94. Solid-state structure of (^{Em}L)Fe(O_2CCH_3) (**12**) with thermal ellipsoids at 50% probability level. Hydrogens and solvent molecules in the unit cell were omitted for clarity, except FeO_2C–*H*. Color scheme: Fe (*orange*), O (*red*), F (*yellow-green*), N (*blue*), C (*gray*), H (*white*).

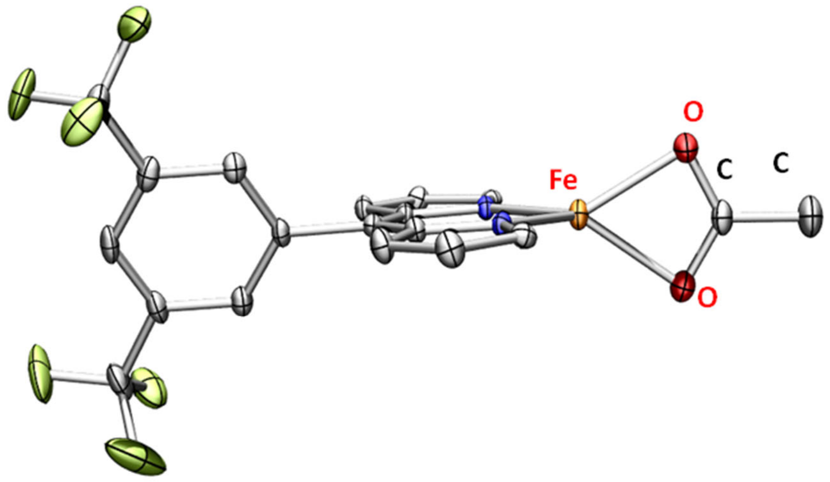

Figure S95. Truncated solid-state structure of (^{Em}L)Fe(O_2CCH_3) (**12**) with thermal ellipsoids at 50% probability level, highlighting the connectivity of the primary coordination sphere. Hydrogens, and solvent molecules in the unit cell are omitted for clarity. Color scheme: Fe (*orange*), O (*red*), F (*yellow-green*), N (*blue*), C (*gray*), H (*white*).

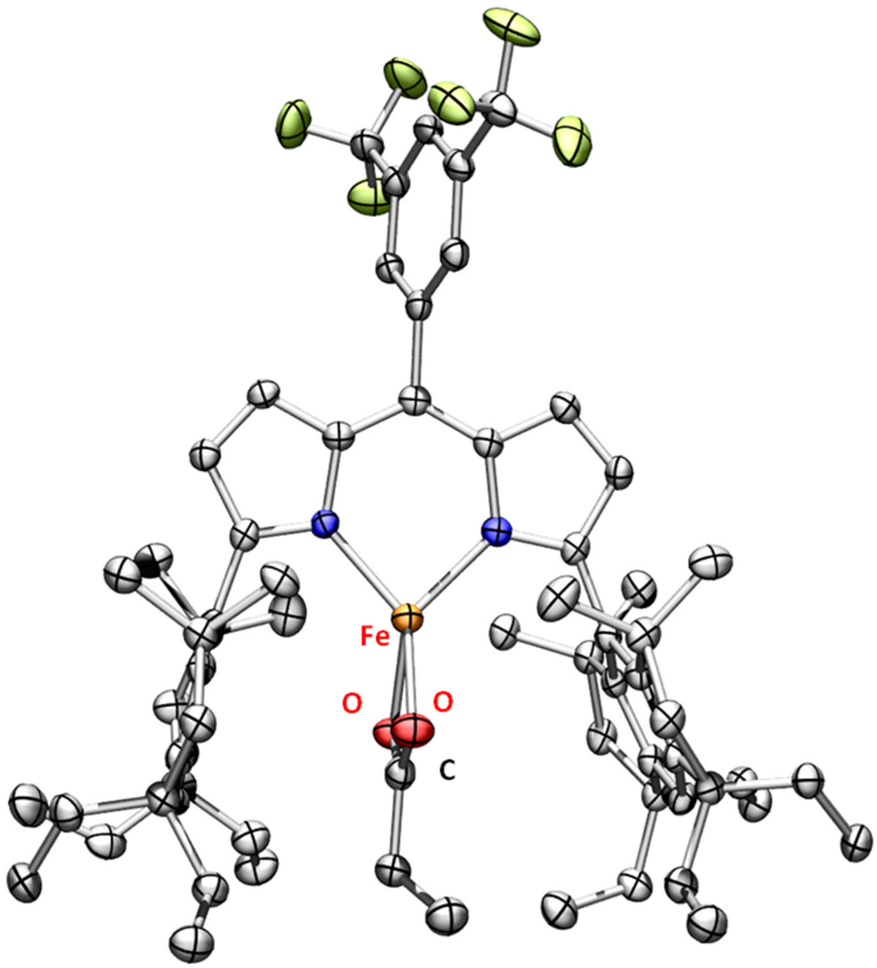

Figure S96. Solid-state structure of ([Em]L)Fe($O_2CC_2H_5$) (**13**) with thermal ellipsoids at 50% probability level. Hydrogens and solvent molecules in the unit cell are omitted for clarity. Color scheme: Fe (*orange*), O (*red*), F (*yellow-green*), N (*blue*), C (*gray*), H (*white*).

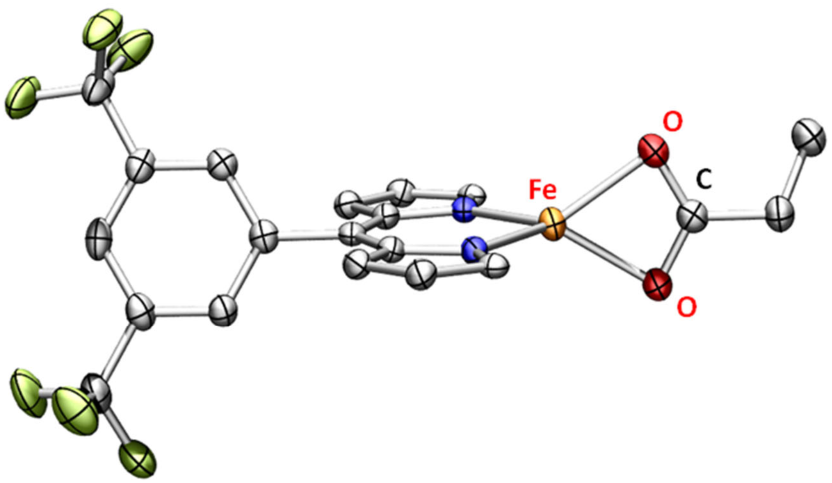

Figure S97. Truncated solid-state structure of ([Em]L)Fe($O_2CC_2H_5$) (**13**) with thermal ellipsoids at 50% probability level, highlighting the connectivity of the primary coordination sphere. Hydrogens, and solvent molecules in the unit cell are omitted for clarity. Color scheme: Fe (*orange*), O (*red*), F (*yellow-green*), N (*blue*), C (*gray*), H (*white*).

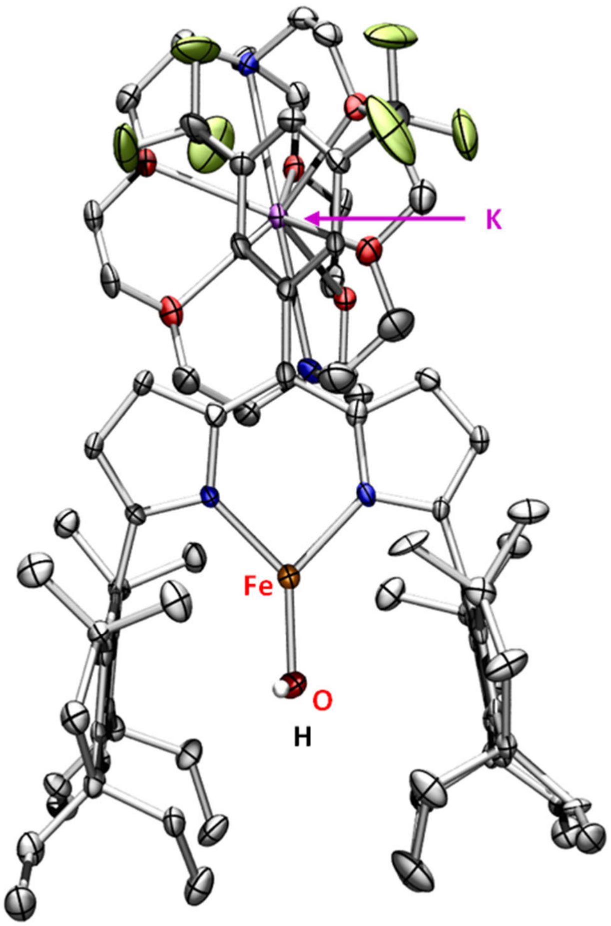

Figure S98. Solid-state structure of [KC222][(^{Em}L)Fe(OH)] (**14**) with thermal ellipsoids at 50% probability level. Hydrogens and solvent molecules in the unit cell are omitted for clarity, except FeO–*H*. Color scheme: Fe (*orange*), O (*red*), K (*orchid*), F (*yellow-green*), N (*blue*), C (*gray*), H (*white*).

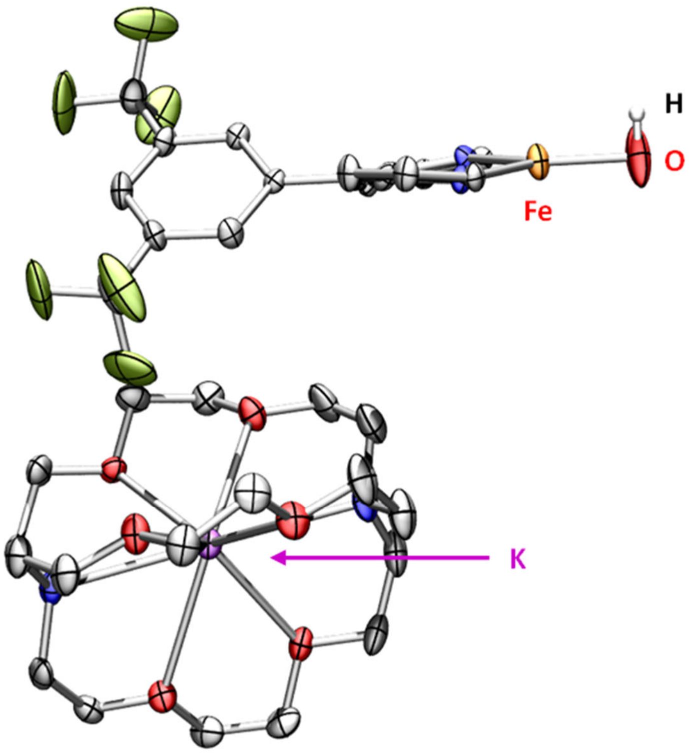

Figure S99. Truncated solid-state structure of [KC222][(^{Em}L)Fe(OH)] (**14**) with thermal ellipsoids at 50% probability level, highlighting the connectivity of the primary coordination sphere. Hydrogens, and solvent molecules in the unit cell are omitted for clarity, except FeO–*H*. Color scheme: Fe (*orange*), O (*red*), K (*orchid*), F (*yellow-green*), N (*blue*), C (*gray*), H (*white*).

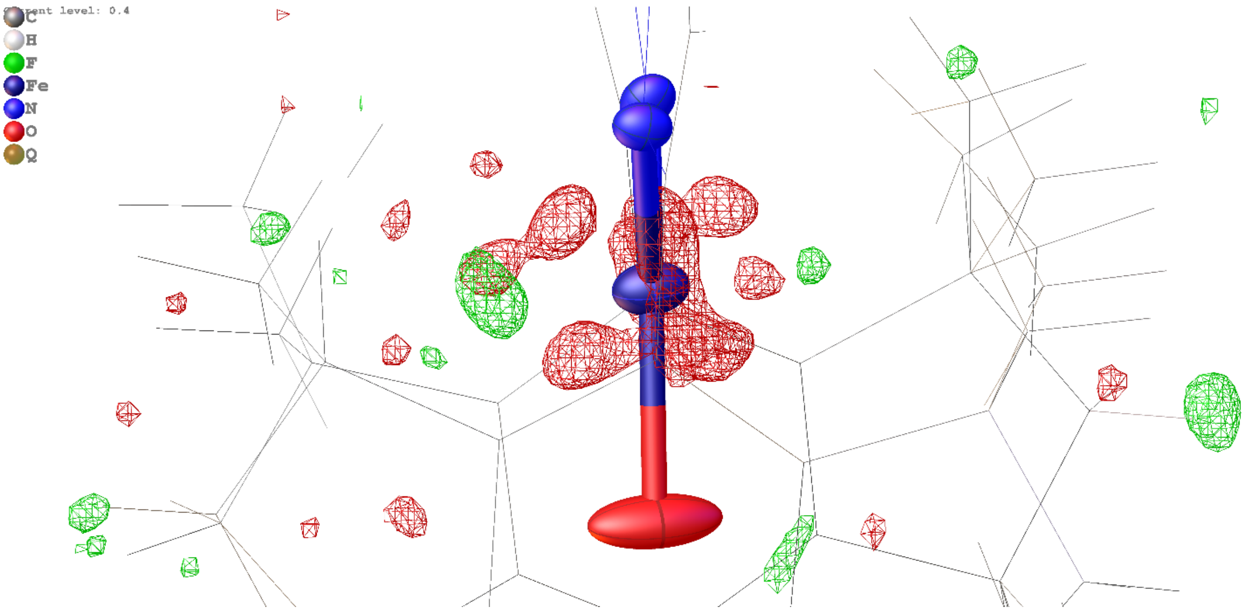

Figure S100. Fourier-difference map for [KC222][(^{Em}L)Fe(OH)] (**14**) in which FeO–*H* was located. The electron density map was generated at the 0.40 e Å^{-3} level.

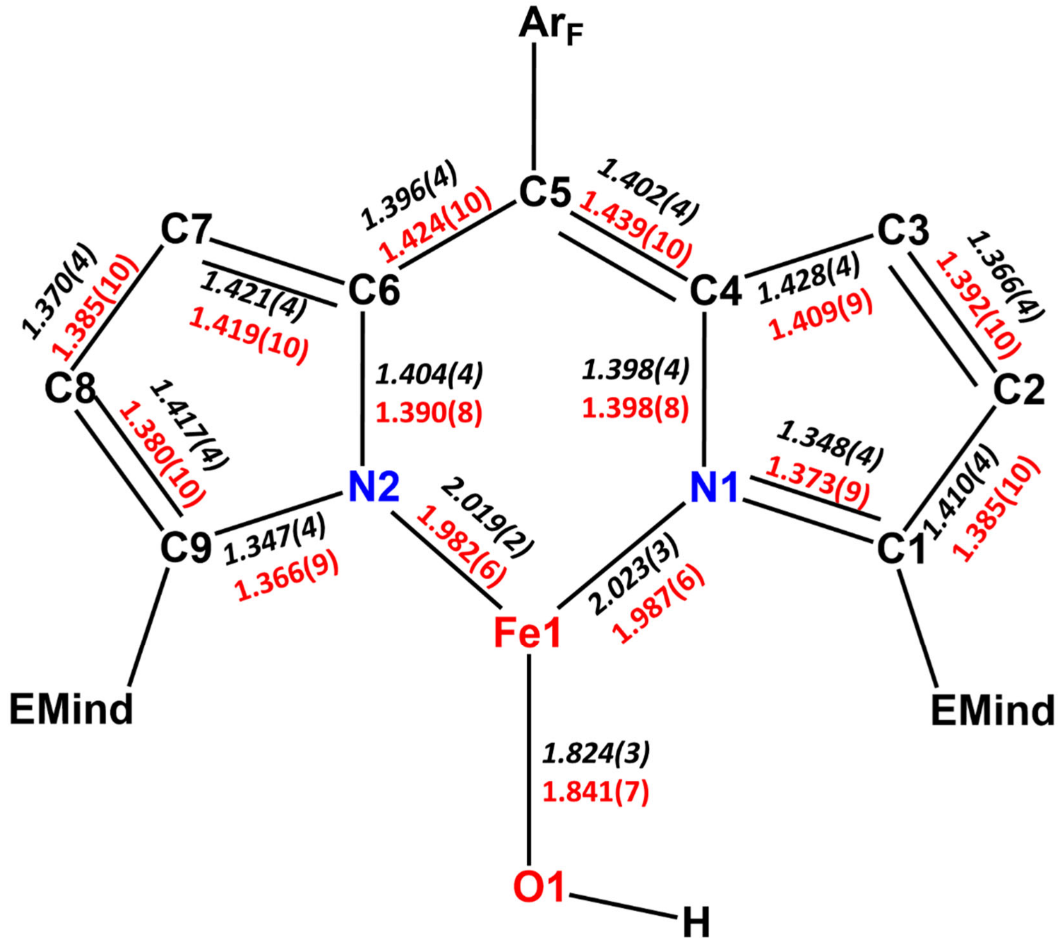

Figure S101. Bond distances (Å) of (^{Em}L)Fe(OH) (**1**) (*black)*, and [KC_{222}][(^{Em}L)Fe(OH)] (**14**) (red) at the dipyrrin iron hydroxo moieties. The elongation around dipyrrin meso position (*d*(C4–C5), *d*(C5–C6)) reveals the ligand radical character of **14**.

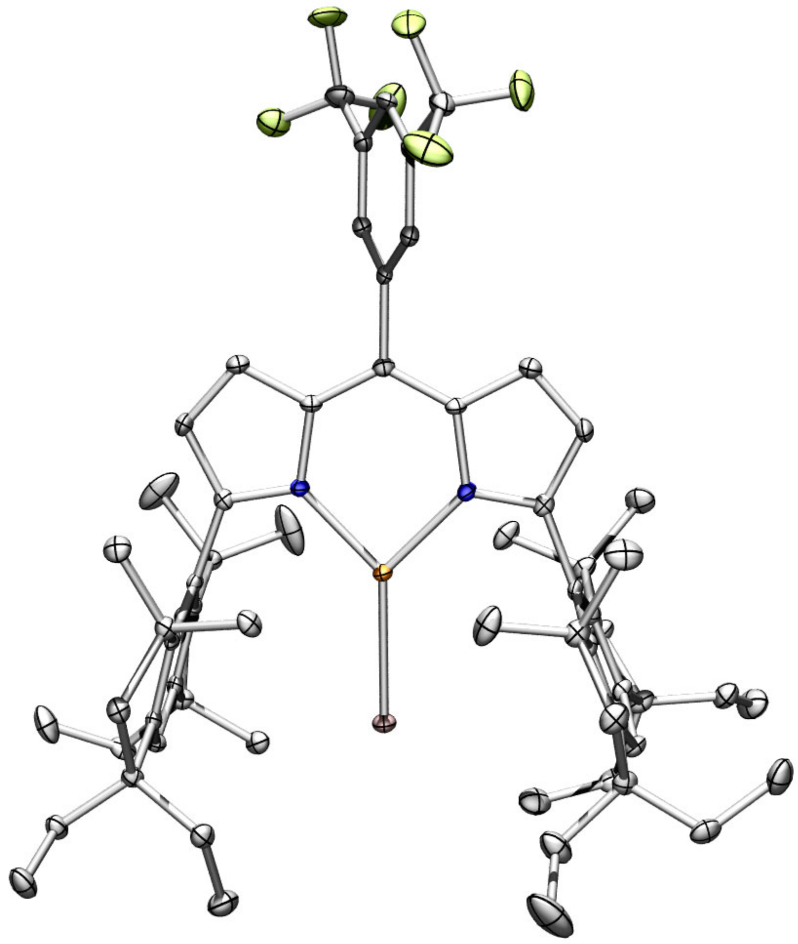

Figure S102. Solid-state structure of (^{Em}L)Fe(I)] (**16**) with thermal ellipsoids at 50% probability level. Hydrogens and solvent molecules in the unit cell are omitted for clarity. Color scheme: Fe (*orange*), F (*yellow-green*), N (*blue*), C (*gray*), I (*pink*).

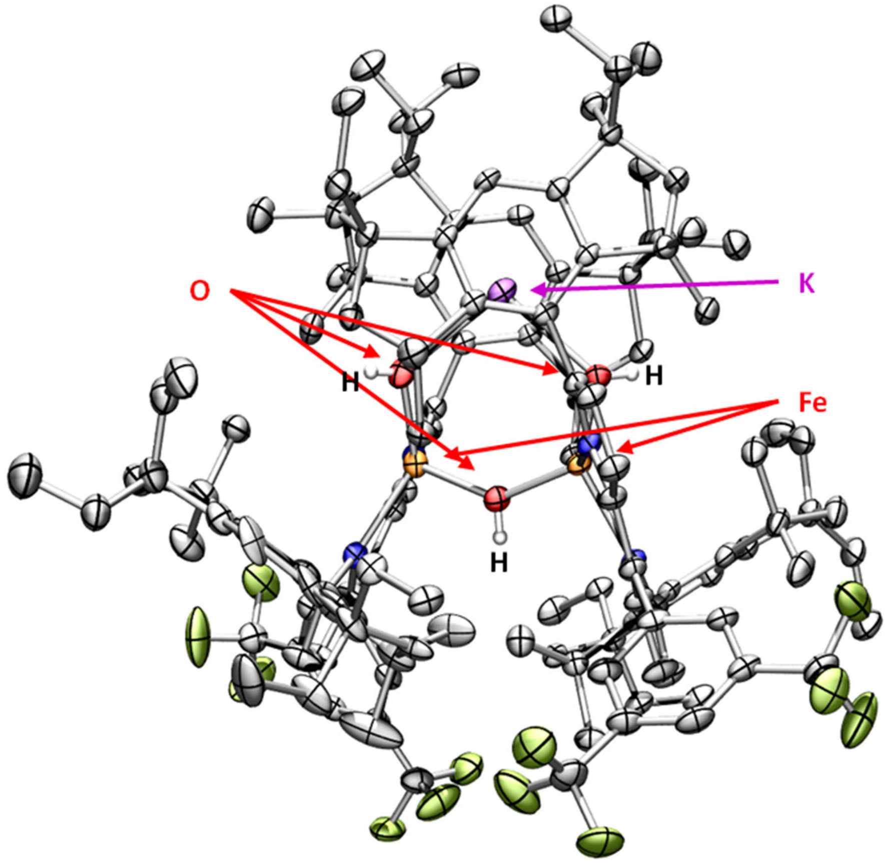

Figure S103. Solid-state structure of $\{(^{Em}L)Fe\}_2(\mu\text{-}OH)(\mu\text{-}OH)_2(K)$ (**SI-1**) with thermal ellipsoids at 50% probability level. Hydrogens and solvent molecules in the unit cell are omitted for clarity, except Fe_2O–*H* and FeO–*H*. Color scheme: Fe (*orange*), O (*red*), K (*orchid*), F (*yellow-green*), N (*blue*), C (*gray*), H (*white*).

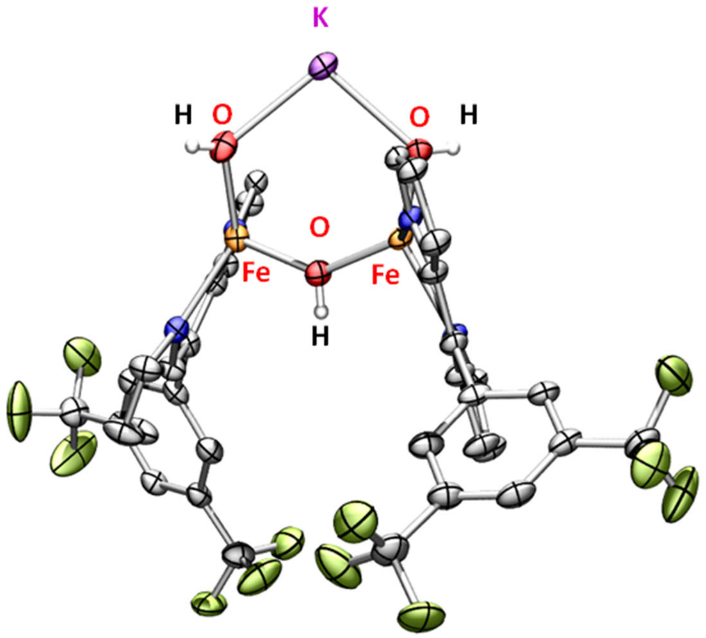

Figure S104. Truncated solid-state structure of $\{(^{Em}L)Fe\}_2(\mu\text{-}OH)(\mu\text{-}OH)_2(K)$ (**SI-1**) with thermal ellipsoids at 50% probability level, highlighting the connectivity of the primary coordination sphere. Hydrogens, and solvent molecules in the unit cell are omitted for clarity, except Fe_2O–*H* and FeO–*H*. Color scheme: Fe (*orange*), O (*red*), K (*orchid*), F (*yellow-green*), N (*blue*), C (*gray*), H (*white*).

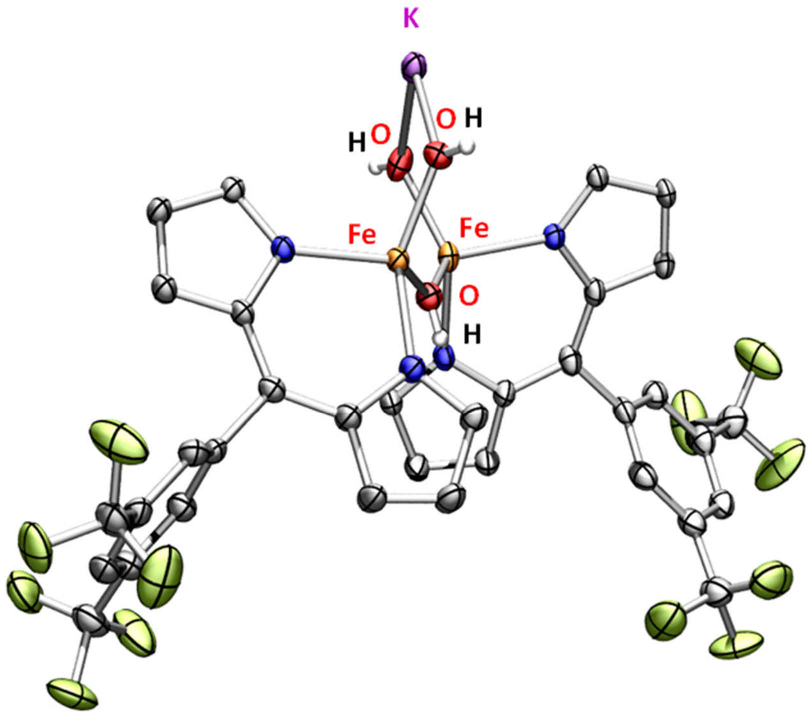

Figure S105. Truncated solid-state structure of {(^{Em}L)Fe}$_2$(μ-OH)(μ-OH)$_2$(K) (**SI-1**) with thermal ellipsoids at 50% probability level, highlighting the connectivity of the primary coordination sphere. Hydrogens, and solvent molecules in the unit cell are omitted for clarity, except Fe$_2$O–*H* and FeO–*H*. Color scheme: Fe (*orange*), O (*red*), K (*orchid*), F (*yellow-green*), N (*blue*), C (*gray*), H (*white*).

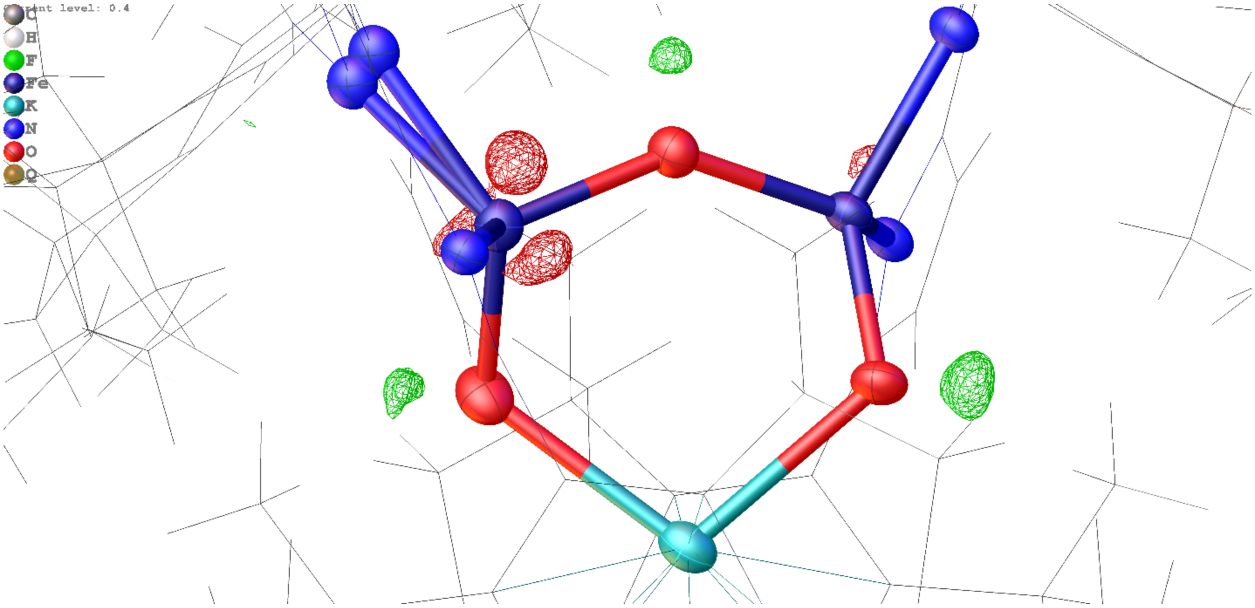

Figure S106. Fourier-difference map for {(^{Em}L)Fe}$_2$(μ-OH)(μ-OH)$_2$(K) (**SI-1**) in which FeO–*H* were located. The electron density map was generated at the 0.40 *e* Å^{-3} level.

Computational Details

All calculations including geometry optimizations and frequency calculations were carried out using ORCA5 program package.[22] All calculations employed unrestricted Kohn-Sham theory (UKS) except closed-shell singlet calculations. The B3LYP functional[23] was used with def2-TZVP[24] (Fe, N, O), def2-SVP[25] (H, C, F, S) basis set, RIJCOSX[26] approximation, and D3BJ[27] dispersion correction. All optimized structures possess non-imaginary frequency, and all transition state structures possess one-imaginary frequency which is stretching mode of generating/breaking bond during reaction. All free-energy calculations assumed 298.15 K and 1 atm. Frequency of O–H vibrational mode were scaled by a factor (0.967) obtained from the ratio v(O–H) of (^{Em}L)Fe(OH) between experimental and calculated. The exchange coupling constant (J of **14** was calculated from the energy difference between broken symmetry solution and high spin configuration as shown in equation *S4*.

$$J = -((E_{HS} - E_{BS}))/(<S^2_{max}>) \quad (S4)$$

The zero-field ^{57}Fe Mössbauer parameters were calculated using the same level of theory with increased radial grid on Fe (IntAcc 7). The benzene matrix was included using a dielectric continuum model.[28] The isomer shift was calculated *via* linear regression of DFT calculated electron density (ρ) at the nucleus to experimental value[29,20] determined by dipyrrin iron complexes (δ = b(ρ–a)+c, a = 11580, b = –0 .355, c = 1.418).[1,31]

p*K*a of the hydroxo complexes were calculated with the same level of theory following the previously reported methods[32,33] including a dielectric continuum model to describe THF solution.[28] The structure was optimized including solvation, and the free energy were obtained by numerical frequencies calculation. The organic molecules with well defined acidity (picric acid, benzoic acid, pyridinium, DBU, TBD, and triethylamine)[34] were also calculated with the same level of theory and calibrated to minimize the discrepancy by the level of theory.

Table S5. Comparison of observables between experimental and DFT calculated with geometry optimized structure of Fe(OH) and Fe(HCO_3) with (^{Em}L) and truncated (^{Me}L) scaffold.

	**Experiment**		**Geometry optimized**			
	**(^{Em}L)Fe (OH)**	**(^{Em}L)Fe (κ^2-HCO_3)**	**(^{Em}L)Fe (OH)**	**(^{Em}L)Fe (κ^2-O,O-HCO_3)**	**(^{Me}L)Fe (OH)**	**(^{Me}L)Fe (κ^2-O,O-HCO_3)**
d(Fe–O) (Å)	1.824(3)	2.085(2), 2.080(3)	1.82	2.13, 2.11	1.82	2.13, 2.12
d(Fe–N_{dipyrr}) (Å)	2.023(3), 2.019(2)	2.019(2), 2.024(2)	2.04, 2.04	2.03, 2.03	2.01, 1.99	1.98, 1.99
v(O–H)	3650	3586	3650[a]	3578[a]	3698[a]	3593[a]
Relative energy (kcal/mol)	0.0	−2.0[b]	0.0[c]	+0.8	0.0[c]	+0.9

a) Scaled by 0.967
b) Determined using K_{eq}
c) Relative energy of (^{Em}L)Fe(OH)+CO_2

Table S6. Geometry of (^{Em}L)Fe(OH) (solid-state structure, DFT optimized structure) and ligand truncated (^{Me}L)Fe(OH).

	**(^{Em}L)Fe(OH)**		**(^{Me}L)Fe(OH)**				**(^{Me}L)Fe(OH) (thf)**
	**Solid-state structure**	**Optimized (*S* = 2)**	***S* = 2**	***S* = 1**	***S* = 0 (open shell)**	***S* = 0 (closed shell)**	***S* = 2**
d(Fe–OH) (Å)	1.824(3)	1.82	1.82	1.78	1.78	1.75	1.85 *d*($Fe–O_{thf}$) = 2.25 Å
d($Fe–N_{dipyrr}$) (Å)	2.023(3), 2.019(2)	2.04, 2.04	2.01, 1.99	1.93, 1.92	1.94, 1.92	1.88, 1.88	2.04, 2.06
Spin density (Fe)	N/A	3.74	3.74	2.01	0.03	0.00	3.76
Relative free energy (kcal/mol)	N/A	N/A	0.0	+22.2	+33.0	+45.7	N/A

Table S7. Zero-field ^{57}Fe Mössbauer spectrum parameters of 1 in frozen benzene and THF solution, and DFT predicted parameters.

	**(^{Em}L)Fe(OH)** (δ, $|\Delta E_Q|$) (mm/s)	**(^{Em}L)Fe(OH)(thf)** (δ, $|\Delta E_Q|$) (mm/s)
Experiment (90 K)	0.73, 0.78	0.97, 1.72
DFT calculated (truncated ^{Me}L ligand)	0.64, 0.36	0.95, 1.43

Table S8. Geometry of (^{Em}L)Fe(κ^2-O,O-HCO_3) (solid-state structure, DFT optimized structure) and (^{Me}L)Fe(OH).

	**(^{Em}L)Fe(κ^2-O,O-HCO_3)**		**(^{Me}L)Fe(κ^2-O,O-HCO_3)**			
	**Solid-state structure**	**Optimized (*S* = 2)**	***S* = 2**	***S* = 1**	***S* = 0 (open shell)**	***S* = 0 (closed shell)**
d(Fe–OH) (Å)	2.085(2), 2.080(3)	2.13, 2.11	2.13, 2.12	2.01, 2.02	2.01, 2.03	1.96, 2.08
d(Fe–N_{dipyrr}) (Å)	2.019(2), 2.024(2)	2.03, 2.03	1.98, 1.99	1.93, 1.93	1.93, 1.93	1.90, 1.91
Spin density (Fe)	N/A	3.80	3.79	2.06	0.00	N/A
Relative free energy (kcal/mol)	N/A	N/A	0.0	+11.4	+24.6	+43.6

Table S9. Comparison of optimized structure of (^{Em}L)Fe(κ^2-O,O-HCO_3) and (^{Em}L)Fe(κ^2-O,OH-HCO_3).

	**(^{Em}L)Fe(OH)**	**(^{Em}L)Fe(κ^2-O,O-HCO_3)**	**(^{Em}L)Fe(κ^2-O,OH-HCO_3)**
d(Fe–O) (Å)	1.82	2.13, 2.11	1.97, 2.27
d(Fe–N_{dipyrr}) (Å)	2.04, 2.04	2.03, 2.03	2.02, 2.04
v(O–H) (cm^{-1})	3650[a]	3578[a]	3543[a]
Relative free energy (kcal/mol)	0.0[b]	+0.15	+6.40

a) Scaled by 0.967
b) (^{Em}L)Fe(OH)+CO_2

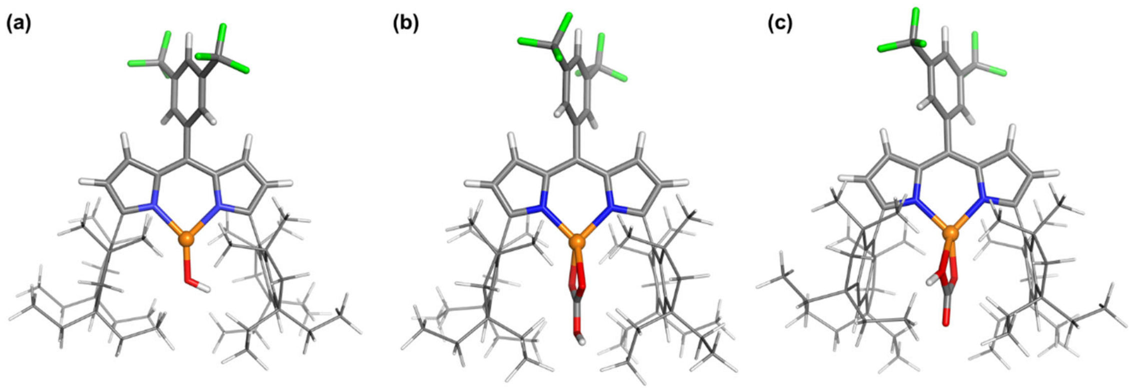

Figure S107. Optimized structure of (^{Em}L)Fe(OH) (a), (^{Em}L)Fe(κ^2-O,O-HCO$_3$) (b), and (^{Em}L)Fe(κ^2-O,OH-HCO$_3$) (c).

Table S10. Geometry of [(^{Em}L)Fe(OH)][KC$_{222}$] (solid-state structure, DFT optimized structure).

	**Solid-state structure**	**Geometry optimized**			
		***S* = 5/2**	***S* = 3/2**	***S* = 1/2[a]**	***S* = 3/2 (BS4,1)**
d(Fe–OH) (Å)	1.841(6)	1.85	1.85	1.81	1.85
d(Fe–N$_{dipyrr}$) (Å)	1.983(6), 1.986(6)	2.00, 2.00	1.99, 2.00	1.91, 1.93	2.00, 2.00
d(C–C$_{meso}$) (Å)	1.433(10), 1.438(10)	1.44, 1.44	1.44, 1.44	1.43, 1.43	1.44, 1.44
ν(O–H)	3637	3651[b]	3650[b]	3689[b]	3651[b]
Spin density (Fe)	N/A	3.74	2.02	1.99	3.72
Spin density (C$_{meso}$)	N/A	0.49	0.47	–0.46	–0.47
Relative free energy (kcal/mol)	N/A	+0.9	+27.0	+18.8	0.0

a) the same solution with BS(2,1)
b) Scaled by 0.967

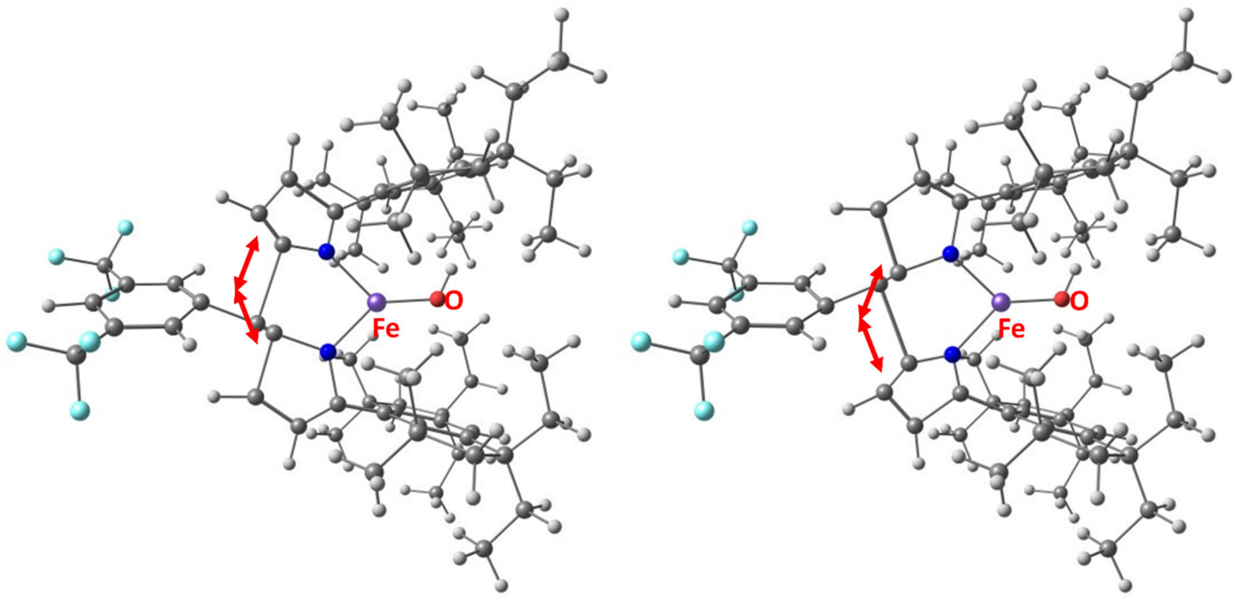

Figure S108. Visualization of dipyrrin vibrational mode (v = 1551 cm^{-1} from optimized structure) of (^{Em}L)Fe(OH), which disappears by one-electron reduction to **14**. The displaced geometry were visualized by multiplying displacement scale factor of ±1 to the corresponding vibrational mode using Chemcraft.[35]

Table S11. DFT calculated p*K*a of [(^{Me}L)Fe(H_2O)]$^+$, (^{Me}L)Fe(OH), [(^{Me}L)Fe(H_2O)(I)]$^+$, and (^{Me}L)Fe(OH)(I).

Equilibrium			p*K*a (calculated)
[(^{Me}L)Fe^{II}(OH$_2$)]$^+$	⇌	**(^{Me}L)Fe^{II}(OH)** + H$^+$	4.0
**(^{Me}L)Fe^{II}(OH)**	⇌	[(^{Me}L)Fe^{II}(O)]$^-$ + H$^+$	53.8
[(^{Me}L)Fe^{III}(OH$_2$)(I)]$^+$	⇌	**(^{Me}L)Fe^{III}(OH)(I)** + H$^+$	–0.4
**(^{Me}L)Fe^{III}(OH)(I)**	⇌	[(^{Me}L)Fe^{III}(O)(I)]$^-$ + H$^+$	34.9

Table S12. Geometry of transition state during the reaction between CO_2 and (^{Me}L)Fe(X) (X = H, CH_3, CH_2CH_3, NH_2, OH (*S* = 2, 1, 0 (open-shell), 0 (closed-shell)), F, SH.

X	H	CH_3	C_2H_5	NH_2	OH (*S* = 2)	OH (*S* = 1)	OH (*S* = 0, open shell)	OH (*S* = 0, closed shell)	F	SH
d(Fe–X) of (^{Me}L)Fe(X) (Å)	1.63	2.03	2.03	1.87	1.82	1.78	1.78	1.75	1.80	2.26
d(Fe–X) (Å)	1.68	2.21	2.16	1.97	1.94	1.83	1.87	1.84	3.04	2.42
d(Fe–O_{CO2}) (Å)	2.32	3.75[a]	3.86[a]	3.35[a]	2.28	2.29	2.21	2.18	1.89	2.13
d(FeO–CO) (Å)	1.20	1.24	1.22	1.20	1.21	1.18	1.20	1.20	1.29	1.23
d(FeOC–O) (Å)	1.17	1.22	1.21	1.20	1.17	1.15	1.16	1.16	1.19	1.18
∠(O–C–O) (°)	159	136	142	145	152	172	159	159	131	146
∠(Fe–X–C) (°)	93.9	137	137	100	92.3	87.1	89.8	88.4	71.5	72.4
d(X–CO_2) (Å)	1.93	1.67	1.88	1.83	1.90	2.41	2.04	2.06	1.39	2.29
d(Fe–N_{dipyrr}) (Å)	1.98, 1.99	1.94, 1.95	1.96, 1.97	1.97, 1.98	2.00, 2.01	1.94, 1.95	1.95, 1.95	1.90, 1.92	1.97, 1.99	1.98, 1.99
Imag. Freq. (cm^{-1})	–343	–41	–402	–169	–270	–46	–238	–235	–25	–111
Spin density (Fe)	3.81	3.74	3.74	3.79	3.79	2.02	–0.03	N/A	3.77	3.77

a) Shortest Fe–O distance while Fe–O distance is too long to interact.

Table S13. p*K*a[36] and calculated Δ*G*° of the reaction between (^{Em}L)Fe(X) CO_2.

X	H	CH_3	CH_2CH_3	NH_2	OH	F	SH
p*K*a of conjugate acid (HX)[a] (kcal/mol)	400.4	416.6	420.1	399.6	390.8	371.5	351.2
Calucated Δ*G*° (kcal/mol)	–22.1	–22.6	–27.1	–15.1	+0.9	+19.0	+97.5

a) gas phase

Table S14. Geometry of transition state during the reaction between C_2H_4 and (^{Me}L)Fe(H).

***d*(Fe–H)** of (^{Me}L)Fe(H) (Å)	1.63
***d*(Fe–C_2H_5)** of (^{Me}L)Fe(C_2H_5) (Å)	2.03
***d*(Fe–H)** (Å)	1.66
***d*(Fe–C_{C2H4})** (Å)	2.12
***d*(C–C_{C2H4})** (Å)	1.43
***d*(H_{FeH}–C)** (Å)	1.62
***d*(Fe–N_{dipyrr})** (Å)	1.99, 1.98
**Imag. Freq.** (cm^{-1})	–843
**Spin density** (Fe)	3.64

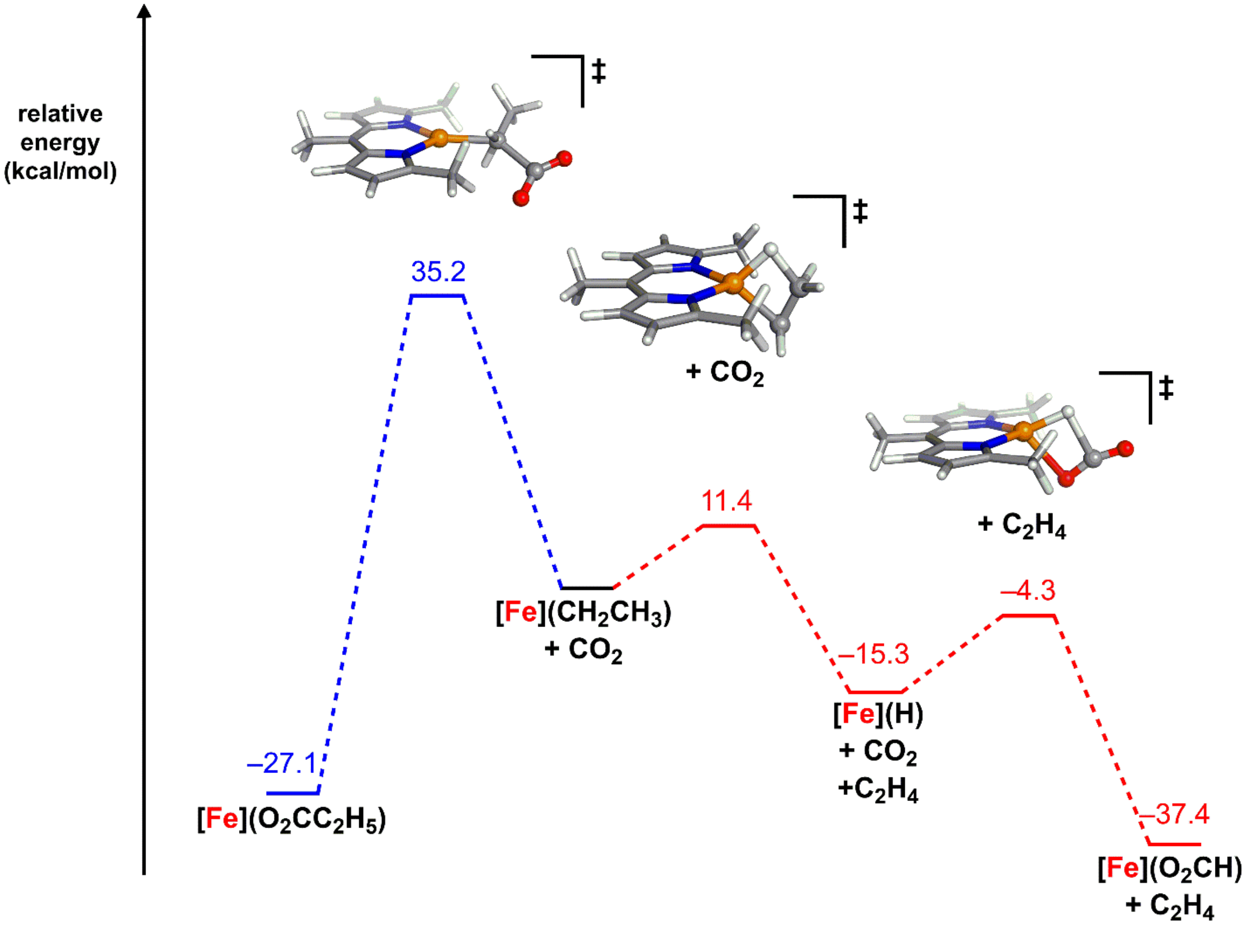

Figure S109. DFT calculated competitive reaction pathway for (^{Me}L)Fe(CH_2CH_3) towards CO_2, and β-hydride abstraction to generate (^{Me}L)Fe(H) followed by formato generation.

Table S15. Coordinates of the optimized molecular structure for the (^{Em}L)Fe(OH).

Fe	1.79672464312898	7.04595753548165	16.67056352086111
O	1.88233506674064	6.16040957239969	18.26276208731911
N	2.11862961090373	6.63438150914983	14.70190205100655
N	1.47891026395302	8.99958248678774	16.18530613879897
H	1.76744125065143	6.67598528278250	19.07026973941178
C	2.31409074681930	5.39654345329380	14.22350561481463
C	2.05700201793896	7.51521115857581	13.62113378579036
C	1.29805290498000	9.96285594010303	17.10203471764636
C	1.60456908533996	9.60457532115495	14.93814697851218
C	2.38864505910455	4.24935844736288	15.16468542330014
C	2.39350108046720	5.42759391514864	12.80011698785761
C	1.83260701092093	8.89889906181307	13.73955253402587
C	2.22531672078653	6.75056424527423	12.42052456847330
C	1.30484720660292	11.24234757129773	16.47829408568666
C	1.12519175666731	9.59155768815477	18.52920386774057
C	1.49076808010421	11.02042757447495	15.12016668011340
C	3.61559161559531	3.88190098872237	15.76470566349378
C	1.21542511327843	3.54956388546929	15.53629714842014
H	2.55658063877931	4.56591884915826	12.15688715958822
C	1.81180955946177	9.69766804210092	12.47986710002550
H	2.21608128770768	7.14503737506391	11.40683223090649
H	1.16770904996648	12.19550684243163	16.98393562766909
C	2.23632935675683	9.53680693939448	19.40115815304968
C	-0.14047810514578	9.20441970770547	19.02415029778789
H	1.55363505606549	11.77158390681812	14.33587509015752
C	5.01454068617263	4.48145496437123	15.55156084715440
C	3.65088332349457	2.84936568695306	16.71104292194230
C	-0.21529130939411	3.70256594499964	14.99561153078230
C	1.27704719111945	2.54949733606900	16.51547439650148
C	0.63879825059909	10.36794829982905	12.10135216761698
C	2.94449832485091	9.79641563415719	11.66207742109109
C	3.69678419073348	9.92797154592275	19.13450224741916
C	2.07322761032616	9.10512113651392	20.72665965596815
C	-1.49215079471967	9.14492969824969	18.29544621515214
C	-0.27312672184132	8.75746893652631	20.34824368062386
C	5.93062012911900	3.55698354220822	16.42497621314689
C	5.09139344979168	5.93436700954210	16.06206818750320
C	5.46565152146039	4.43134662548844	14.08141528690637
C	5.05026336070601	2.54195530208259	17.20922561513005
C	2.48721419559022	2.19467292198237	17.10549995733322
C	-1.02724601975300	2.65955004531570	15.83815718954143
C	-0.78797758446155	5.11514824610560	15.20908712533106
C	-0.29546833341087	3.34131098419195	13.49985399181364
C	-0.07538210562917	1.94102980290172	16.83647565386205
C	0.59754033149768	11.11082887169342	10.91928059780965
H	-0.24435151827249	10.30272888695399	12.73871643701681
C	2.90195768922704	10.55364564630224	10.48729172207577
H	3.86838895914341	9.29749761243048	11.95990798616416
C	4.41779441963093	9.37192451704556	20.40055675458947
C	3.83661891434267	11.46129578084637	19.03887390750485
C	4.31008836248593	9.28905177176455	17.87711435757026
C	3.36692630028900	9.16098596482898	21.52291104037675
C	0.82524507378189	8.70716783981583	21.20422965097302
C	-2.48141954251041	8.88898540433789	19.47338006110505
C	-1.86310519197156	10.44955088228608	17.57205819609196
C	-1.54154971122464	7.97946289682098	17.28680737557222
C	-1.68652486883037	8.29799131483024	20.66690062607580
H	6.64258185410281	3.02266243219850	15.77965998069672
H	6.54170432136053	4.15865237006239	17.11296681300372
H	4.69598836067493	6.01012273551256	17.08474474643194
H	4.52080364197928	6.62093686159010	15.42207984353873
H	6.13884891231818	6.27782996556189	16.07132659095296
H	6.52173351131400	4.73798621659990	14.00049874788193
H	4.87268236961445	5.10498983621600	13.44742796065411
H	5.37468570351448	3.41176762974235	13.67470531655031
C	5.14863386105070	2.72608372080896	18.74278383234734
C	5.43586857425976	1.07468076744511	16.88345709237771
H	2.52359898288596	1.40688784977419	17.86246251250390

H	-1.84560969567045	3.15859435404814	16.37606354381231
H	-1.50811374902031	1.93054867918491	15.16994351928757
H	-1.86544892657607	5.12659441845808	14.97719080364155
H	-0.30141797683121	5.85921876312252	14.56506620601376
H	-0.66318924829972	5.43045616673002	16.25453654831839
H	0.14007033717643	2.34868262323557	13.30856576705558
H	0.23398262222385	4.07519060652034	12.87708888873167
H	-1.34857840968547	3.32159095893743	13.17351835507032
C	-0.45857195477581	2.22061690375531	18.31336658936200
C	-0.06489689964831	0.40477166130306	16.63308185163133
C	1.72948609906055	11.20935149163687	10.10683205551401
C	-0.69950733228608	11.76421839269234	10.50565084157339
C	4.13099430502306	10.62281693598548	9.61384803298572
H	5.24320044139845	10.02747648939240	20.71550592287248
H	4.86966431973294	8.40146709316734	20.14645568250093
H	3.31094410053465	11.84727858396793	18.15373201215243
H	4.89907858909191	11.74223535939384	18.95117935262380
H	3.42261959211690	11.96292452525718	19.92584122897845
H	4.12829358819400	8.20366906479435	17.86072128733307
H	5.40130238420693	9.44604083940626	17.87840629836678
H	3.91364886694563	9.72047327087684	16.94836480769063
C	3.28756061192222	10.37127124125600	22.49490330312368
C	3.63023308545841	7.86422413540427	22.32586319810009
H	0.71213071975920	8.35448855850255	22.23294438516547
H	-2.92179158834047	9.85078807570529	19.77530747453306
H	-3.31894210694848	8.24727191181035	19.16265800102191
H	-2.91606890302626	10.40887623020338	17.24706185761841
H	-1.24398788915211	10.61643182766682	16.68007425995143
H	-1.74507366639826	11.31928149427324	18.23784382080393
H	-1.23355252246860	7.03312659060437	17.75102580399190
H	-0.88071765548350	8.16454849467992	16.42924128265331
H	-2.56556178889474	7.85255719845439	16.89982133155804
C	-2.19782688360978	8.82277400110217	22.02942192751606
C	-1.68347765985629	6.74244298563954	20.67001883785683
C	4.77379978948769	4.10581943760169	19.27876652839734
H	4.49669477643636	1.97002673752693	19.21473546225606
H	6.17766414688981	2.46920241193712	19.05363488886539
C	5.25610679709100	0.64470515176003	15.42867041079602
H	6.48614175676120	0.92433621520509	17.19180497537382
H	4.83537897558924	0.40956071471248	17.52874999425998
H	-1.42856278289422	1.72892174836254	18.51193962329714
H	0.27696929411831	1.70394212585691	18.95468455530884
C	-0.52782902223272	3.69054008860806	18.72351179331542
C	0.42256577352763	-0.08463131536092	15.27086515833159
H	0.56638649105518	-0.04204787653901	17.42116033999609
H	-1.08699259512859	0.03041220035890	16.82143111540364
H	1.70216075500719	11.80209061356413	9.19323957981438
F	-1.56718290777384	10.85708468824559	10.02036630129361
F	-0.51349275156725	12.68996330444186	9.55078999223131
F	-1.30149047259727	12.36709958292879	11.54603961719109
F	5.23714599791901	10.85871907920287	10.34081530348966
F	4.33029281638861	9.46306676495535	8.96082498802149
F	4.03453914521586	11.59088353498054	8.68892444140453
H	3.08062913949799	11.27770288655655	21.90378386203530
H	2.40105122423188	10.22826420860260	23.13745051246481
C	4.51485288146532	10.60925781399365	23.37438794602520
C	3.42670639070377	6.55910868522412	21.55851396941618
H	4.66500193199055	7.90455719815760	22.70602938207114
H	2.98522831433405	7.86322958143916	23.22326788197867
C	-2.00460449859796	10.31755221134989	22.28570174884504
H	-3.26971875305484	8.57592343702078	22.11282039236483
H	-1.69963209514025	8.25191642319426	22.83364967681572
C	-3.02653775965559	6.06056396500384	20.92608427750866
H	-1.27229648117254	6.39106765876435	19.71153148955061
H	-0.95756371824006	6.41197493936932	21.43348781398432
H	5.48420779586417	4.88080474757494	18.95006044845255
H	4.78848905877059	4.10523284439134	20.37937166715096
H	3.76898575765714	4.42084699025829	18.95509318980358
H	5.86370644928526	1.25268923128549	14.73983613816847
H	4.20553739318547	0.73710275822990	15.11124251690475

H	5.55875264679903	-0.40563295826658	15.29161764482098
H	-1.34418226228946	4.22144418067108	18.20844724000578
H	0.40656224878763	4.23327648479622	18.51031753390826
H	-0.72408179494414	3.77789684651028	19.80419500611444
H	-0.20736363838651	0.29027547278129	14.44889234322815
H	0.40368973140781	-1.18497764373595	15.22123704272125
H	1.45519609214236	0.24387039562174	15.07374099052121
H	5.43021440378004	10.73840890203859	22.77447032546229
H	4.69143895266536	9.77824563885223	24.07452838657658
H	4.38661405022052	11.52321183288531	23.97607927167908
H	3.98604674977441	6.54155344666205	20.61157349589170
H	2.36823154389920	6.39037644383690	21.30980728516719
H	3.76482374836972	5.69775942316587	22.15542855208921
H	-2.49674394180889	10.93468163593694	21.51726010564941
H	-0.93923660246391	10.59432102740345	22.29335425935489
H	-2.43425256897425	10.60329543726942	23.25905811780548
H	-3.79177179685933	6.37308030661385	20.19728606074476
H	-3.41748412090820	6.27595012492920	21.93259537519556
H	-2.92158501449675	4.96744254709295	20.83982919391629

Table S16. Coordinates of the optimized molecular structure for the (^{Em}L)Fe(κ^2-O,O-HCO_3).

Fe	9.92583636470984	7.20044765819117	16.74211716047932
O	8.94611138734922	8.59044275622023	18.01754039068000
O	10.99973404928858	7.90817165801843	18.41562143931509
N	10.20929549455750	7.51114191501266	14.75884913744828
N	9.56424817808832	5.23419634765088	16.37103282378441
C	9.98695301581917	8.60984169370732	18.72599914893738
C	10.44370276144720	8.71925294705321	14.22651267165221
C	10.08661869420535	6.58984109743110	13.72166490672270
C	9.53637378099680	4.58918656469521	15.13653114330418
C	9.33732518580802	4.32351208458766	17.32948815658007
O	10.01546195493596	9.38053675046507	19.81856930356175
C	10.49446396001910	8.62518329994899	12.80510384919521
C	10.55527125040847	9.88764887428300	15.13676182977985
C	10.26851344838380	7.29386833193010	12.48628804385267
C	9.77970608768185	5.22833275176753	13.90642241945614
C	9.27032399875864	3.20020656248908	15.37427120396607
C	9.13991287696594	3.03861324195022	16.74518924132163
C	9.35245772612198	4.78057803131005	18.74350398916977
H	10.87510136211337	9.24896963368466	20.24976864838746
H	10.68177556204235	9.44998294179488	12.12159797164745
C	9.41146155024394	10.65439944414877	15.45376820319331
C	11.77552391595759	10.19461895552194	15.78150254790168
H	10.22311407657694	6.85519294831843	11.49195895274553
C	9.71877477040296	4.37490783025701	12.68330226178916
H	9.19936652966215	2.42652817588150	14.61325517627462
H	8.93731494790201	2.12006893272363	17.29072625637254
C	10.55991453966895	4.80084677672493	19.47642085925615
C	8.19135383085951	5.33189616769433	19.33436243222307
C	9.49026354054352	11.68181215725851	16.40752782672695
C	7.99999310551212	10.57362226858819	14.85357826554858
C	11.81821732565989	11.19587089140618	16.76267953101755
C	13.16944154927215	9.59144924512371	15.54822969747024
C	10.87901964087254	4.14165433221759	11.93069639720618
C	8.51768692811255	3.77522324667166	12.28589502719377
C	11.92574183753822	4.20807571241662	19.09856772759335
C	10.61870783321580	5.43398366259340	20.73072891101701
C	8.26821240299377	5.94012095076223	20.59405204053998
C	6.75193779808646	5.35737600133104	18.79726326206873
C	10.67993682356788	11.93478997263397	17.08841290106951
C	8.19861617052650	12.48415100282065	16.49597377488184
C	7.19886902477590	11.54288320392237	15.77603572368551
C	7.36612362169664	9.17178911501567	14.90601857535118
C	7.99285982697721	11.06758762712517	13.39178947718003
C	13.20043871131209	11.33730894044234	17.38350607697462
C	14.08282126174977	10.54594210725363	16.38104348180391
C	13.29219552585434	8.14373726189959	16.06215584769237
C	13.58635736955244	9.63445710274489	14.06797272959618
H	11.82189199891960	4.59246613153056	12.24275117858166

C	10.83673656866396	3.31494351222833	10.80654400142517
C	8.47943467783804	2.95413678214794	11.15355626843744
H	7.61118161568481	3.94936507654179	12.86812673651468
C	12.70394911058717	4.32940762651336	20.44449398143857
C	12.61729825494565	5.00331981356533	17.97256831123995
C	11.86036851047364	2.72614329123625	18.68963879083575
C	12.02676942199128	5.41844373184190	21.31749732287826
C	9.47706950188437	6.00936184135039	21.28871355021179
C	6.93499598068366	6.51626418576409	21.04551897825274
C	5.94714517769014	5.87939557402128	20.03061116490598
C	6.58214531901656	6.31353581436223	17.59898513703470
C	6.24690608419393	3.95878329311467	18.40254200534373
H	10.72574709475993	12.71900064473989	17.84886292688459
C	7.75445758562515	12.82138123536397	17.93770562474119
C	8.43303930229409	13.81085035466368	15.71355602560005
H	6.43220424773081	12.09426133612453	15.21147820586406
H	6.66653425257524	10.94534505585293	16.52995894886795
H	7.83310862165086	8.47450033782896	14.19748576503772
H	6.29749030447244	9.24329370698199	14.64450080129118
H	7.43710176359167	8.74592890743073	15.91695388043911
H	8.43219124166887	12.07110078195491	13.29597167083562
H	6.95854843912280	11.11169579985734	13.01274551651229
H	8.55942494737779	10.38530957133914	12.74283467485004
C	13.17455760466123	10.66653091517438	18.78662344707544
C	13.64298984221235	12.81403055450581	17.51717440771520
H	14.88746465914292	9.98902309835606	16.88317812014983
H	14.57625172047865	11.25052901001917	15.69597239675850
H	14.33135335129031	7.79402433482901	15.95019107091605
H	12.64747650246562	7.45885465138358	15.49314008588276
H	13.01461297865901	8.06942927089796	17.12047656864549
H	13.44534837523323	10.64218389527858	13.64539301337513
H	13.01018248725025	8.92407837418912	13.45874182364980
H	14.65272133644464	9.37024044153786	13.97184184114663
C	9.63673138927252	2.71630564914268	10.41168149528645
C	12.08966552680669	3.09431969066189	9.99389788264953
C	7.16275297542253	2.34001450730311	10.74179891816624
H	12.63264101005817	3.36513134347293	20.97264831947226
H	13.77403975889568	4.52402368147075	20.27643752282837
H	12.70082420557020	6.06821603266657	18.21789691706681
H	12.06027499767814	4.91722078938707	17.02847739256772
H	13.63104635065070	4.60719548272972	17.79938346607980
H	12.87785407110921	2.30089749161380	18.67744873914026
H	11.43301217614637	2.59217136358718	17.68725732419246
H	11.25692822964324	2.14348250692306	19.40380870866805
C	12.73125615649781	6.78731575461388	21.09461434633425
C	12.01220947749059	5.02192473788994	22.81031073326982
H	9.51327526682460	6.48803106113931	22.26957636088091
C	7.00034699500029	8.06177626972711	20.89483334107244
C	6.59584645004372	6.13805280610608	22.50721123576954
H	5.16457132136020	6.58715977579305	19.72101386136882
H	5.51896005290747	6.36980109613843	17.31415480903641
H	6.73934748688066	3.58722089395221	17.49337325691987
H	5.16250223381631	3.99476242998839	18.20595446597395
H	6.42324902337053	3.23208231103086	19.21176014381155
H	5.42818535173590	5.03217568536011	20.50268664522171
H	6.92862083340289	7.32567078220247	17.84374626055064
H	7.14276268803097	5.96150465602151	16.72123915059256
H	8.42326871626214	13.60799359889497	18.33296339660768
H	6.75582094810848	13.28676383911705	17.88121267067437
C	7.71414683094252	11.65761161998605	18.92465140311257
H	9.28306759194186	14.33041226752180	16.18985438285387
H	8.77551993534323	13.55928666112142	14.69758059486188
C	7.24436091817086	14.76788023899254	15.62370623929631
H	7.07431356880020	10.83459640484024	18.57239092846599
H	7.31982889905134	11.99080929683519	19.89844410989909
H	12.38644216947144	11.16585540360315	19.37838017644429
H	12.84361766817700	9.62414117495826	18.65991661957903
C	14.48735815237809	10.69182595434364	19.56962984942627
H	13.07120384501650	13.27668188694223	18.34193944789681
H	14.69722966810412	12.83123138854469	17.84115028516594

C	13.49150932849944	13.67357106363300	16.26194122648289
H	9.60674400855326	2.06111400708179	9.54156027022071
F	12.07228264521226	1.90610759049835	9.36754633374801
F	12.23296290893873	4.04073190418600	9.04713384253110
F	13.19124199010664	3.13526694043576	10.76196369177774
F	7.31546918284090	1.42609334697452	9.77067147679383
F	6.30828177493415	3.27341896786432	10.28582595643927
F	6.56116004729067	1.73362219591407	11.78141235943793
H	13.80833819419955	6.65931615866894	21.29419918952858
H	12.64300397993519	7.04453091376245	20.03053201471305
C	12.20528276975612	7.95645166593333	21.92623708953967
H	11.41283606619601	5.75159544278071	23.38002911981635
H	11.47161579273553	4.06376816064221	22.89730290515337
C	13.39107504278432	4.89264676507998	23.45787639853404
H	7.86074136075162	8.42583046647956	21.48177448285761
C	5.74757780613273	8.83333383726227	21.30880171555381
C	6.74854545875167	4.65861211603891	22.86193916503577
H	7.24652634805610	8.29783321477687	19.84880372494238
H	7.23189395140632	6.73917275958078	23.18209409832201
H	5.56064020673108	6.45721545313334	22.71429035170051
H	8.70942278969722	11.22949301262715	19.09409566430383
H	7.49386441993266	15.63204354413929	14.98717214832448
H	6.95180757395310	15.16120972437551	16.60932817451567
H	6.35843946322274	14.28219168749152	15.18343319284394
H	15.31341480553602	10.24165111315565	18.99596528017916
H	14.39221522345944	10.11651476628565	20.50475620510914
H	14.79034340701369	11.71394863352130	19.84371440812136
H	14.06128508089749	13.26487948630824	15.41222222755427
H	13.86374809923448	14.69441888678009	16.44408469738033
H	12.44037159897611	13.74910655083181	15.94464213325961
H	11.11224408678418	8.05583403368200	21.84069791043127
H	12.66737053902334	8.90333740885213	21.59814771171277
H	12.43133167977588	7.84938214954768	22.99850996368862
H	14.02470646551155	4.16688829097490	22.92296953311131
H	13.30384436882665	4.54864163881044	24.50080822771878
H	13.93052844109493	5.85293967607641	23.47394054769477
H	4.85036245469734	8.47693045566287	20.77640223264581
H	5.86507422259504	9.90257433993697	21.07129371254201
H	5.54454089796249	8.75575081138561	22.38845704582334
H	6.43939153031510	4.47273356192877	23.90298164216548
H	7.79073524992977	4.32137743815605	22.75390647172813
H	6.12885531728643	4.01598515590007	22.21616236785334

Table S17. Coordinates of the optimized molecular structure for the (^{Em}L)Fe(κ^2-O,OH-HCO_3).

Fe	3.71595337567480	-0.23921998122575	16.77776579751127
O	2.54820749274755	-1.78759878748474	17.95891393156976
O	4.50799637241884	-0.96147458989411	18.42714430372630
C	3.64468029530485	-1.81594778303291	18.85006490140768
N	3.70268599025124	-0.72054084232760	14.80044721998361
N	3.29084595678729	1.68008972519385	16.30598470144871
O	3.65027769254950	-2.55688392757396	19.80170898061724
C	3.95919243089264	-1.94823701989019	14.31824544140324
C	3.67002261987303	0.17100661555417	13.72820996795917
C	3.19147748812235	2.25772960571377	15.04379304682525
C	3.01234548535282	2.61447672950993	17.22860896979719
C	4.13010182568052	-1.89331681259453	12.90616440206788
C	4.01596485746168	-3.10080037477643	15.25366204599592
C	3.95148639479889	-0.56875187290389	12.53390514692067
C	3.39231268848104	1.55004943161927	13.84335555558910
C	2.82609387777651	3.63329290180594	15.22372965126191
C	2.72193002794767	3.85386190830303	16.58947619694893
C	3.03098847338979	2.19852190650158	18.65490306782944
H	4.35378571337385	-2.73988807862321	12.26142331696293
C	2.85981929423451	-3.87017928072802	15.52564199147504
C	5.20937643532908	-3.40187760201776	15.94931209796366
H	4.01920658560772	-0.15828036861137	11.52929810375661
C	3.26391117115128	2.32143542092938	12.57443535789319
H	2.65269097236180	4.35756359348060	14.43137869726933
H	2.46722805951924	4.78262746899115	17.09446166124544

C	1.86125376254208	1.68444521862070	19.26506791671387
C	4.24345071744180	2.15709806376221	19.37660821616846
C	1.47143011576361	-3.78636756022784	14.87040297538373
C	2.90428579631889	-4.88355573532942	16.49447740232163
C	5.21390887271562	-4.38723847638633	16.94822813482106
C	6.62813252545727	-2.87057655399467	15.69533361592215
C	2.31565678770584	1.94065671878143	11.61395233264776
C	4.06565912680640	3.44372143384120	12.32416249265025
C	0.41744619578462	1.67521310001101	18.73941580934250
C	1.93970187277398	1.08339344086987	20.52747312594149
C	4.29765571217786	1.54690594839034	20.64229450908172
C	5.62102147673210	2.70454561242392	18.97634573176853
C	0.67484050490545	-4.92536507863391	15.59544939986407
C	0.77853788343802	-2.42942563079691	15.10660564053660
C	1.52892125398727	-4.04673192520369	13.35241515414439
C	4.06373640481499	-5.12153574373365	17.23315406914619
C	1.62204114973243	-5.69722428863878	16.56036941151169
C	6.57095166173152	-4.55608225182197	17.61227886821487
C	7.47119585866166	-3.56459314659943	16.81670566619701
C	6.78301518297597	-1.34428012830967	15.80663349379540
C	7.10553757812283	-3.32257740205513	14.30013508159327
H	1.67516628741180	1.07882738655532	11.80640378916328
C	2.17939552299081	2.66320842990306	10.42669037199522
C	3.91403388466945	4.17168303677546	11.14062208142123
H	4.81143726733639	3.74592067833579	13.06105917640605
C	-0.38667926279495	1.16206454023726	19.97793291794747
C	0.23848815629827	0.72182569983845	17.54051628407959
C	-0.08577724948661	3.07608916549272	18.34931266051318
C	0.60110782995272	0.53970371582762	21.00172622710732
C	3.15255849986377	0.99476909833925	21.21254772055983
C	5.70221297552835	1.56373588479910	21.23172724219005
C	6.39510361973286	2.61877103537499	20.32674381317413
C	6.30113887929152	1.84226345681785	17.89298567941222
C	5.58061970032252	4.16481548301820	18.49556189519067
H	-0.16348543527450	-4.48920633000027	16.15876447800757
H	0.21871123753321	-5.60919817290312	14.86472157963046
H	0.68824064025146	-2.19784327619550	16.17753560214080
H	-0.23923245533738	-2.45299144056713	14.68420498117286
H	1.32383110603737	-1.60166050525791	14.63393175469622
H	1.99613072223609	-3.20895791335490	12.81846881137329
H	0.50834511762705	-4.16896395961335	12.95404672669426
H	2.09775908519466	-4.95865099188288	13.12235755991360
H	4.07881924663523	-5.89529250532834	18.00495486427285
C	1.02877755321306	-5.80100856999527	17.98741567220236
C	1.89530052833135	-7.15082147684795	16.08338583779611
C	7.05727642807566	-6.02298457011142	17.51913881726021
C	6.46011072965597	-4.12626684179940	19.09950364028612
H	8.34570623073204	-4.07146091713914	16.38260637636212
H	7.87074209044026	-2.79965732418596	17.49776697865965
H	6.41414644624953	-0.98881637160145	16.77903553661370
H	6.25533825977576	-0.80902147958543	15.00611522895597
H	7.84965630313583	-1.07566545117097	15.73649503143676
H	8.16666662459257	-3.05826972006863	14.15922066321741
H	6.52755209139634	-2.83212622802709	13.50404791103286
H	7.00305267961760	-4.41075987313350	14.17849108642189
C	1.16936003278086	2.19147161710847	9.40962316122807
C	2.97390348406168	3.78518269364879	10.18355749513753
C	4.78915810032787	5.38137448816285	10.91987778468917
H	-0.91218332705398	2.01094063745643	20.43915013069556
H	-1.16523242035931	0.44683528790158	19.67412263408439
H	0.80023025108173	1.06693012147966	16.66086303215283
H	-0.82539038463214	0.66640827766943	17.25780929419339
H	0.58262775352826	-0.29123359941400	17.78581617378553
H	0.11473283842569	3.80410737284457	19.15152851371819
H	-1.17485856237637	3.04732222865543	18.17876513933431
H	0.38608513694676	3.43966490371316	17.42681497715145
C	0.63529077247721	-1.00927078312547	20.89260100644052
C	0.28251585647628	0.96363989244802	22.45575162315568
H	3.19368620220002	0.51481478975407	22.19197625692946
C	6.39238199998016	0.17852967384670	21.06953172247126

C	5.67822942336212	2.02025909169697	22.70843129606679
H	6.33448970967139	3.60118905189338	20.82231550000252
H	7.46287976323397	2.40493630588288	20.16787013159605
H	7.32790476623763	2.20045083692222	17.71444560850207
H	5.75916904275787	1.90353176259749	16.93761340690261
H	6.35516175442436	0.78850026742831	18.19234097396507
H	5.01280778969813	4.79682225601049	19.19670353149093
H	5.12344750411699	4.25987290867594	17.50088162873662
H	6.60647345131412	4.56421533088837	18.43109597387565
H	0.04710554029869	-6.29881125550069	17.89919916138729
H	1.65650569237149	-6.48614437952123	18.58261154953777
H	1.83214533193722	-6.96681461391652	13.90062344844221
H	3.45146574086308	-6.74749724321975	14.60461327811335
C	0.87653696271751	-4.49268448376040	18.75970235210729
H	0.94284554791849	-7.70844126276782	16.13447251296924
H	2.56328243657712	-7.62428128754441	16.82377691480020
C	2.51004764538267	-7.31215274546563	14.69572273178119
H	8.07115146266563	-6.08091139139606	17.94817963773666
H	6.41796909753880	-6.63822901812784	18.17732799469928
C	7.07135510222790	-6.63559073151982	16.11960059546137
C	7.74528031584939	-4.22981576458263	19.92034010270374
F	1.50563616995281	0.98157617541317	8.92346213009409
F	-0.05317657246411	2.06615160634458	9.95496128455963
F	1.06764791944901	3.03133717076129	8.36885044398429
H	2.85935665295486	4.35348860544367	9.26154535391254
F	6.08644841156780	5.03901348355011	10.83125085040665
F	4.68188671433962	6.24611017985617	11.94581584138757
F	4.46723796266023	6.04187909893697	9.79716639974039
H	0.82977631079598	-1.26771108294747	19.84083214588996
H	1.51292740255218	-1.37965030773606	21.44759780871731
C	-0.61688517946661	-1.74326469354634	21.37185314768012
H	-0.75589035792021	0.66893291492177	22.68267176394481
H	0.91622133832546	0.37198917996940	23.14073191683056
C	0.46294708032905	2.44992391998712	22.76537200155963
H	-0.15024971003188	3.08351753295117	22.10441656927238
H	6.33292798882040	-0.11202411891453	20.01138368988012
H	7.46441416330341	0.31082887465862	21.29586024667065
C	5.82977352742281	-0.96568500081460	21.90903292401441
H	5.15683941923218	2.99235804100304	22.75076584873976
H	5.05808267853196	1.32487127288612	23.29737420620911
C	7.05088733862818	2.14547549635707	23.36914068135557
H	0.36207760227554	-3.71986080714191	18.16241196733802
H	1.85102743554869	-4.11101944582702	19.10175837743306
H	0.26881783007545	-4.63734621884880	19.66625387060452
H	2.73328541435678	-8.37182428338686	14.49468875489406
H	7.76549136710786	-6.10522324327334	15.44905331886713
H	6.07332690580922	-6.60988959766698	15.65391963845448
H	7.39606038201006	-7.68764595059405	16.15794536101134
H	8.08082039955515	-5.27073987074037	20.04923327365550
H	7.58733747936471	-3.81049319740024	20.92665260659214
H	8.57460847098569	-3.66789861183629	19.45923024055622
H	-1.52729746392039	-1.37843318113868	20.86797423364355
H	-0.77275008473376	-1.63496004259881	22.45624490177396
H	-0.53373477234386	-2.82114397110522	21.16048966885546
H	1.50994363306819	2.76598935298697	22.64183249217254
H	0.16328433463286	2.67115084550337	23.80223355228328
H	4.78773800212729	-1.19074120417493	21.64308507135440
H	5.88329387671739	-0.75896213626463	22.99010190770305
H	6.40211280071043	-1.88776855891176	21.72225752876633
H	7.71083128926119	2.82954501855694	22.81113104369558
H	7.56287252603088	1.17294300342764	23.43696674819082
H	6.95784260350073	2.53976264944167	24.39380036877638
H	5.66902110134767	-4.72365018474829	19.58263290715805
H	6.09406570780143	-3.08922945956410	19.12619997517182
H	2.01396972578248	-2.59178578403123	18.05890705964429

Table S18. Coordinates of the optimized molecular structure for the (^{Em}L)Fe(OH)(I).

Fe	7.96033794019213	9.36944498293377	5.18010820109777
N	7.70901606674544	8.60778832449573	7.03522640350485

N	8.78757418911230	11.08442097247989	5.86779890655795
O	6.41339806589202	9.70273075748866	4.28588914161321
I	9.26860855603841	8.00810667384228	3.48138320891269
C	7.36459549767899	7.35198405895167	7.39200565576339
C	7.80283564449284	9.37553682268950	8.20328789881752
C	9.31832093599422	12.09496402137206	5.15051308464173
C	8.68262197263256	11.50999877285173	7.19701513228185
H	6.39432609018896	9.46418903516259	3.34920274234625
C	7.25864421800933	6.25717379925977	6.39878744193883
C	7.20488074319497	7.27367254771249	8.80122661373557
C	8.23663392094436	10.71314971275086	8.26649998238192
C	7.48215564266967	8.53208322376237	9.31149763186116
C	9.58626211467391	11.95050460233097	3.70192862503076
C	9.55805240893921	13.21634702979924	5.98946167566998
C	9.15170252828531	12.85908668510874	7.26559368568253
C	8.38486441661963	5.45526677804277	6.11018222222731
C	6.04763832287745	5.99830091696469	5.71949984392496
H	6.93279170264469	6.37777802880327	9.35325980805215
C	8.28858202412036	11.33015118164624	9.62665796958425
H	7.48489901754371	8.82914971899150	10.35714300619251
C	10.82168470728829	11.44421045415807	3.24486968724926
C	8.59372978286078	12.27977205336500	2.75504253220326
H	9.97616003778657	14.16596610691143	5.66459548964898
H	9.17634329272542	13.47826677493171	8.15864816194386
C	8.30617583242064	4.46556477094881	5.11546713858846
C	9.74812661154457	5.39813660445023	6.82161733074920
C	6.01390589773141	5.04951218534414	4.68782240074254
C	4.63120625178306	6.48253315307853	6.06142247725348
C	7.12334834557452	11.51846140630918	10.37823189766706
C	9.52261504536775	11.71915126376852	10.17234583015758
C	11.02908380979695	11.22440310150097	1.87213408851571
C	12.10600225546484	11.16792102092276	4.04193528608841
C	8.78734715628221	11.96402392153382	1.40186845173160
C	7.33642845505644	13.14486012280731	2.92046576515621
C	7.13961361586129	4.28753289516141	4.37361164625931
C	9.56600091735851	3.61132681806579	5.05255748559337
C	9.59898763996119	4.74037080104285	8.21263700163159
C	10.56292196764262	4.48981890972415	5.85324329470038
C	10.46913902202135	6.74285086792801	7.01460791061035
C	4.65788013015602	4.97503689036097	4.00579473074377
C	3.78245471977730	5.93497787451767	4.86722447467793
C	4.20162994615096	5.80318493920360	7.38153223914928
C	4.44342808104656	7.99878052799429	6.21104148961048
C	7.19247135643609	12.08576557332469	11.65652744086355
H	6.15820684949996	11.22797268135257	9.95954638880207
C	9.58407033866892	12.29223436088940	11.44231698291135
H	10.43607763911406	11.57108236112210	9.59614768690235
C	9.99478300781025	11.42536213757069	0.95631040559126
C	12.46574426016014	10.82024376398770	1.56752149359463
C	12.74324211895050	12.50351655755887	4.49017751700837
C	11.94785515754889	10.28355054629917	5.29057315340165
C	12.97551576108683	10.42692518189474	2.97929143077988
C	7.58195369384698	12.32516263749920	0.54238593094685
C	6.44417338609406	12.89696694575223	4.14402158682270
C	6.56461544199272	12.83378506510935	1.60576548282828
C	7.79634113479252	14.62215799175559	2.95323060333051
H	7.09924579902904	3.53827023942965	3.57811620913195
C	10.05476289154300	3.30028905135949	3.61831283722349
C	9.24152734462048	2.25982971732750	5.75923388808213
H	9.05322166457020	5.40227635646104	8.89950774950187
H	10.59388955931730	4.55178211029507	8.64840495440889
H	9.06054184876970	3.78413184576267	8.16780262616947
H	11.10138593680611	5.14087032956960	5.14847820153785
H	11.32184299878075	3.89951943575843	6.38813129000768
H	10.53479722617060	7.30591564098226	6.07471638518701
H	11.49517706611506	6.55570905423055	7.37227965129645
H	9.97265595054188	7.37677893510065	7.76212909373734
C	4.10776736711564	3.52884416843636	3.95728645167768
C	4.76974600834936	5.47631076138832	2.54025451693014
H	2.87843056966541	5.42756961150510	5.23567921243880

H	3.42931408833573	6.77488909590754	4.25211184513364
H	4.33271628274720	4.71325925209447	7.33580273844068
H	3.13988544787059	6.01636797312807	7.58954891806204
H	4.79317028569118	6.18244961063887	8.22728727564144
H	4.97222976834116	8.39055426230201	7.09198952501892
H	3.37215373588890	8.22197693232413	6.34674133037377
H	4.80782400584538	8.54588437441756	5.33277685961218
C	8.41800380293391	12.47927956241118	12.19215345139960
C	5.92178427029485	12.23179143143893	12.45931015209734
C	10.91630750223255	12.68065858482800	12.03873463876854
H	10.14006423999301	11.19102999518839	-0.10104130055326
C	13.27973620543249	12.04256739667527	1.04022755054675
C	12.61005651750650	9.67370187861131	0.53930995753439
H	12.83179321251584	13.22285101951676	3.66460988115798
H	13.74976642202138	12.32662003208427	4.90349704829858
H	12.13470370862402	12.97270672939210	5.27679275943108
H	11.41663566530960	10.80000647580912	6.10189283789555
H	12.94638543114105	10.01082201502506	5.67049563073250
H	11.40615738408721	9.35708936024948	5.06094280635671
H	14.04934030098755	10.63642254404763	3.10728794369965
H	12.83837872308244	9.34467656537741	3.12283330468089
C	7.05925564571651	11.08781510941920	-0.23039935032955
C	7.98467715836301	13.43224002801434	-0.46996262677885
H	6.16142486952995	11.84114975293222	4.23291842030376
H	5.52505838199313	13.49844742028065	4.04499810822983
H	6.93404108683650	13.20520227348140	5.07882568037334
H	5.99819168083114	13.71122158002130	1.25936676736500
H	5.82377985414879	12.04916253691261	1.81788786919254
H	8.37554459943361	14.82509409361672	3.86670083056909
H	6.92176601670136	15.29352848750745	2.95049849759382
H	8.43104764937936	14.87713986170906	2.09247736304772
H	11.02635104303329	2.78375952418082	3.69647363635467
H	9.36458104248076	2.56034921551247	3.17416589466293
C	10.19563750054676	4.47948780000176	2.65984874756460
H	8.79994508844115	2.47127156433954	6.74402847023478
H	8.43714433395822	1.77351942894537	5.18000307641947
C	10.40315868105236	1.28108885697189	5.92987312065873
H	3.11792453430976	3.55790808617073	3.46778212343025
H	4.75505536204644	2.94186440714764	3.28220288779182
C	3.99909621645989	2.79847627733225	5.29319530965687
H	5.35793532153416	4.73610624132839	1.96949521761025
H	3.75637412380344	5.46257046712076	2.09957979793142
C	5.39915805186373	6.85445559888524	2.35371302569956
H	8.46344044299850	12.93346610978615	13.18204329513807
F	6.11001539767445	12.95643908502542	13.57266075643481
F	4.95134026661305	12.82537391040259	11.74453470863312
F	5.45158094634158	11.02775540808077	12.83635752626288
F	11.85774885168386	12.83951833209750	11.09421151822194
F	11.36297829757843	11.74519001177696	12.89600608207535
F	10.82833389890819	13.83237180939009	12.72581927809777
H	13.37347088888523	12.78072539516995	1.84918050046767
H	14.30560181420935	11.68368971092247	0.84177127694439
C	12.74155773374360	12.75816385526024	-0.19827158103751
H	12.35748897793165	10.06266191761017	-0.46107346464430
H	13.68301976879675	9.41450955498387	0.49006743402381
C	11.79071803620871	8.40999615618912	0.78326003358307
H	6.13959165012446	11.37168043493547	-0.76866843917710
H	7.79984482476239	10.83604328104076	-1.01069354811166
C	6.78175553760969	9.84075322889677	0.60791543512921
H	8.37508001413590	14.29265526839195	0.09611784030732
H	8.83614922948362	13.05795553837407	-1.06486553943556
C	6.88512275249223	13.91101745507827	-1.41889308500910
H	10.56731061564863	4.13651259664398	1.68088314449824
H	10.90375793091826	5.23524576041874	3.03225906003057
H	9.23491672385154	4.98909193281019	2.49655341772548
H	10.81221552907551	0.94624193464144	4.96424532307185
H	10.07004643397080	0.38228438248574	6.47344692655477
H	11.23109114151978	1.72298442704360	6.50732045513430
H	3.65401754078589	1.76297408381014	5.14340522268228
H	3.28334601384435	3.28644470048129	5.97220799761890

H	4.97320153914590	2.75686366965321	5.80585872071361
H	5.42559509559225	7.12709146206583	1.28745223227232
H	6.43157732177883	6.87888501100588	2.73302884224447
H	4.83540377589323	7.63978292001389	2.88134002823063
H	12.63057472922178	12.08311691957763	-1.06154535669974
H	13.43038980683433	13.56264767445789	-0.50239233117627
H	11.76034387448733	13.21806638367860	-0.00538368193733
H	11.99584329262769	7.66175923344375	0.00080951069616
H	10.71156002175830	8.62085357003377	0.77279540565651
H	12.02032961111369	7.94233411029920	1.75257435008617
H	6.45937021682181	9.00624738668188	-0.03390117865772
H	5.97131300753699	10.01221878479137	1.33525155752971
H	7.67315652261089	9.51018725141977	1.16223485821882
H	6.56508743163552	13.12091446059546	-2.11525029825554
H	7.24120387438008	14.75795291795602	-2.02691286183211
H	5.99197545686999	14.25331308608271	-0.87152425782835

Table S19. Coordinates of the optimized molecular structure for the $[(^{Em}L)Fe(OH)]^{-}$ by spin-state.

S = 5/2

Fe	1.85636269246603	7.11331367255095	16.59976757560689
O	1.92865646508056	6.21489645763673	18.21780574697461
N	2.17438212808794	6.66386666765868	14.67747200487964
N	1.46224749167728	9.03375238264452	16.17364191182970
H	1.74416489978268	6.75626212867594	18.99448562009965
C	2.32875320381377	5.38791313454381	14.20277578984478
C	2.05642774695590	7.52583267344425	13.59345833009161
C	1.31724959555695	10.00403544646895	17.13021718502259
C	1.63287767285900	9.65261352633755	14.94311707161301
C	2.42894177348467	4.25699371741934	15.16210439269862
C	2.31679268246685	5.40035934318666	12.80450297451924
C	1.84415159655833	8.94572524383993	13.70544635940040
C	2.13540554047926	6.74160476285474	12.41121442826018
C	1.40136008828657	11.26787856656684	16.53769172414512
C	1.10851461406108	9.60972242165360	18.54758982275239
C	1.60146231830624	11.05581117044350	15.15695868662216
C	3.66596441317161	3.89406370307175	15.74539051663547
C	1.27207252901831	3.55095421189385	15.57192892487037
H	2.42985488445219	4.53322784464721	12.15573101567737
C	1.81016676998860	9.72561846648142	12.46079735025599
H	2.04375973957401	7.10349885468995	11.39030785734465
H	1.29914326830680	12.22360105704201	17.04972780164548
C	2.19291783473481	9.53060789366898	19.45220577432325
C	-0.16833470333608	9.22359410994530	19.01796045488551
H	1.72921371989238	11.82458195658354	14.39815089931946
C	5.05978481173270	4.48292144074318	15.47659826650551
C	3.72833577566125	2.87519628005181	16.70521178878747
C	-0.17069752505106	3.69604822197280	15.06180402972325
C	1.35471826455554	2.56511818219641	16.56432000378184
C	0.78165531512702	10.65948883974538	12.20427947433626
C	2.78892260783842	9.56154721553187	11.45152017750216
C	3.66335696513510	9.90447646040055	19.21999317997367
C	2.00055087073296	9.08001342657662	20.76789572921368
C	-1.50577290602400	9.19459638568245	18.26090742860033
C	-0.33616954579489	8.75946953579378	20.33256852457965
C	5.99827558246251	3.59273529941301	16.36208917507156
C	5.15946032659457	5.95644076916153	15.91692169774371
C	5.46217522644170	4.36824639525647	13.99516516426165
C	5.13923739220738	2.59093510279690	17.18659807755135
C	2.57707875076728	2.22033818323158	17.13557764989280
C	-0.96819531972373	2.68162058150343	15.95194875584063
C	-0.72740069679579	5.11883161375968	15.24302673210523
C	-0.28806164368711	3.29290900393367	13.57905290076515
C	0.00786455480436	1.96969077610264	16.93199899738851
C	0.72715203322616	11.37231815197533	11.00587527551198
H	0.00397178494293	10.80409292102036	12.95482182912764
C	2.72750669796983	10.27464118651010	10.25636227356904
H	3.61487146132509	8.87320212102883	11.62591160921268

C	4.35517291488713	9.30806512971112	20.48387311632038
C	3.82577808492887	11.43769166547439	19.16651925889369
C	4.28371716494468	9.29188134663745	17.95344371929916
C	3.28295703361739	9.09100380748303	21.58550738341636
C	0.74022948952935	8.68553224729959	21.21472147488522
C	-2.52349244638445	8.91478300926510	19.40977244122290
C	-1.84418550296690	10.52578056678679	17.57027737444680
C	-1.54817583880661	8.06359433010836	17.21442708431139
C	-1.75735118538027	8.29302262320041	20.60627812140444
H	6.71085418846243	3.04921465700522	15.72367692366133
H	6.60928346974235	4.22068441438511	17.02693859558881
H	4.80892388117835	6.08102419736097	16.95152278317102
H	4.55385424382919	6.60995796570628	15.27649037281920
H	6.20840620201811	6.29437020693532	15.86350011001863
H	6.51045919004488	4.68691183592891	13.86285641008460
H	4.82645989750140	4.99936054211896	13.35959821771780
H	5.37280699354843	3.32796000965889	13.64272559356358
C	5.26005514076346	2.82215680169808	18.71265374503259
C	5.53479205825635	1.11938945282722	16.89848649480658
H	2.63114871531460	1.44575553203517	17.90597038359056
H	-1.75853273310727	3.20500642555425	16.50864642623556
H	-1.48419237518329	1.94613554393869	15.31662055788987
H	-1.80731451031267	5.13456148791890	15.01820571942409
H	-0.23054768845252	5.83908579038404	14.58066384644869
H	-0.58904573656921	5.45995408935782	16.27844451673641
H	0.13571140992301	2.29136216024795	13.40456645531649
H	0.23807236801290	4.00789545583242	12.93372769011516
H	-1.34964335820396	3.27340705475926	13.27793205044521
C	-0.33051684324713	2.27267245040170	18.41524604943524
C	0.00190446153781	0.43091376573101	16.75108946115646
C	1.69666869523375	11.19195232001698	10.01404233992277
C	-0.44038784395926	12.28758426032735	10.75865279897436
C	3.74422824173236	10.02020231772049	9.17825901987110
H	5.18787984881642	9.94148407186261	20.82680025083823
H	4.79324461286258	8.33527690575304	20.21443591465959
H	3.31910720983019	11.84425362823516	18.28046316674385
H	4.89436294297541	11.70594020056969	19.10629433518552
H	3.40056829179275	11.92369706334240	20.05799777822692
H	4.09698345132227	8.20790338903961	17.91183786094197
H	5.37611957273942	9.44750629032539	17.96548865471082
H	3.88488068213404	9.74328225307899	17.03582151417469
C	3.21572474553694	10.27582941705271	22.58826844186886
C	3.51018130302627	7.76934275874643	22.35968298274652
H	0.60112750115493	8.31548619956067	22.23473988528752
H	-2.96948098030040	9.87025444587179	19.72533242925050
H	-3.35510172365785	8.28176431188372	19.06510655718992
H	-2.88238464110297	10.49858462878349	17.19680023292164
H	-1.17623373628101	10.72262670885428	16.72141864724416
H	-1.75559624629482	11.36863013763887	18.27522234244548
H	-1.27514747825526	7.09606304853178	17.65680147406822
H	-0.85024683327513	8.26018134900494	16.39056530464329
H	-2.56417780388216	7.97404807757182	16.79488917259593
C	-2.30242843590515	8.77531257230991	21.97116462505279
C	-1.74910530106159	6.73870664054265	20.56377894460192
C	4.86296353155801	4.21010959453535	19.21029598853025
H	4.62963082728820	2.06849828029089	19.21780106866056
H	6.29990130022651	2.59530015629994	19.01456596296261
C	5.34463111790335	0.65118681683515	15.45663057736645
H	6.58957898246715	0.98311443601652	17.20104042550545
H	4.94326740203026	0.46707655646502	17.56550185670013
H	-1.29553333762361	1.78565538883874	18.65219780691162
H	0.42465108866822	1.76492618420343	19.04154342734528
C	-0.38398758635865	3.74973253015962	18.80171351624233
C	0.45810051137854	-0.07963935677341	15.38556006013391
H	0.64837224918912	-0.00965866652059	17.53106263726349
H	-1.01890924933614	0.06496328665770	16.96563237117977
H	1.65473296825287	11.75515678071482	9.08275412846256
F	-1.56203500317349	11.60051403021424	10.44985816694502
F	-0.21801627142718	13.13789093577731	9.73534013289516
F	-0.73845458138481	13.03622604475202	11.83850326018780

F	4.90096338421173	9.53360972194400	9.66131796869878
F	3.29457645680405	9.12278091657418	8.27189455924307
F	4.04229727963356	11.13990194503807	8.48421755659006
H	3.04782690463996	11.20271661813541	22.01717525082834
H	2.31160096014589	10.14027022983542	23.20788285012876
C	4.42937658348303	10.45611860678638	23.50116659178896
C	3.27261395024703	6.48875592066614	21.56136867399891
H	4.54582432451827	7.77300682534735	22.74186794663700
H	2.86313825732050	7.76487581692489	23.25661980588658
C	-2.11767634452608	10.26248844454777	22.27458008855153
H	-3.37593105710293	8.52401231825034	22.02488882228073
H	-1.81909666569684	8.18348873493605	22.76996619087602
C	-3.09401689913978	6.04002289977105	20.75771362065242
H	-1.30807169861694	6.42134382789029	19.60768062895204
H	-1.04310979445358	6.38785479718528	21.33690478993286
H	5.54919225562574	4.98951272997919	18.84346743030397
H	4.89350094715447	4.24627896714879	20.31036008767711
H	3.84783535587832	4.49372250754512	18.88929929784630
H	5.94442878598804	1.24490522029153	14.74895751256109
H	4.29142157154437	0.73924611651045	15.14745266007611
H	5.64658617330547	-0.40288150809154	15.34181796544014
H	-1.21891880278239	4.27030407959201	18.30678929186776
H	0.53873756405909	4.29295245195612	18.54108756446616
H	-0.54016451906925	3.85769556224501	19.88755051843892
H	-0.18726440001611	0.28970162060668	14.57330556662023
H	0.43594051305492	-1.18130276282858	15.34953943912372
H	1.48603013545145	0.24721236327205	15.16364333571670
H	5.36079357202144	10.57083891690528	22.92309945071088
H	4.56555536964287	9.60133407184800	24.18197096126956
H	4.31660816008605	11.35849988597892	24.12437913028191
H	3.81754298169254	6.48756106354418	20.60650880864901
H	2.20944878798159	6.34963779606727	21.31740780178539
H	3.60140767469657	5.60459918110254	22.13083493678662
H	-2.59390251823433	10.89876435070862	21.51182962990833
H	-1.05232796134865	10.53659795498259	22.30714693363821
H	-2.56622546617335	10.52218968918192	23.24769438458706
H	-3.83967899032707	6.37381608743320	20.01785839405132
H	-3.51689769214669	6.21685455672175	21.75970391005849
H	-2.97794660399412	4.95115240428167	20.63478324380165

$S = 3/2$

Fe	1.90131262753296	7.08665724514461	16.56899411461964
O	2.04490757172573	6.20603080219162	18.19165313961581
N	2.17682705310185	6.65090087247348	14.64538254820574
N	1.46410468132878	8.99969283136872	16.16746216520300
H	1.82986238253753	6.74671620772011	18.96109278148511
C	2.30717645076455	5.37699830866108	14.16481195265246
C	2.02601443894390	7.51763312613929	13.56722958713964
C	1.34642723031090	9.95997105984222	17.13526340048766
C	1.65111164416281	9.63177462248818	14.94271561153256
C	2.44306053986395	4.25577714973679	15.13240935389834
C	2.24493453523028	5.39332264423153	12.76590161500399
C	1.83336715647258	8.93660455406347	13.69614348196995
C	2.05983005762671	6.73550632490440	12.38080621969454
C	1.46765387622571	11.23012894942671	16.55833798654911
C	1.10540212378660	9.56038515143049	18.54647704021991
C	1.65956683310639	11.03306200109398	15.17627359087666
C	3.69947858972712	3.90904693330379	15.68337322137124
C	1.30239725919483	3.56061738650929	15.60127712999178
H	2.33114042244885	4.52770498259010	12.11079816873866
C	1.79524515554372	9.73263332118254	12.46055939180305
H	1.93527567223113	7.10049963974657	11.36434400424135
H	1.38896609200401	12.18120773478270	17.08320618088406
C	2.16968970082013	9.45323012563349	19.47215248399511
C	-0.19006579315064	9.20886250540565	18.99345296942005
H	1.81035786387998	11.80838665179583	14.42845387770807
C	5.08489348756053	4.46925737800821	15.32731571776321
C	3.79422895633250	2.92894006522986	16.68033650955759
C	-0.15377843180787	3.68231629525256	15.12602102687868
C	1.41815435827855	2.60824244558229	16.62273225026944

C	0.78669277381208	10.69563583203003	12.23629213546039
C	2.75305452643319	9.55977193297038	11.43396343526632
C	3.65379175741749	9.78831135428413	19.26932576518803
C	1.94038612449324	9.00951617089041	20.78452852902340
C	-1.51197950344074	9.20401820109733	18.20924640913931
C	-0.39678286920452	8.76101514058128	20.30806624330411
C	6.05255309535548	3.61507194537361	16.21697304929306
C	5.22132524378624	5.96428637803835	15.67672393258162
C	5.41794802818549	4.26692108552394	13.83773587983373
C	5.22110030707328	2.66607749255350	17.12758417414627
C	2.65808387447134	2.29039095770192	17.17183778776526
C	-0.92157466866908	2.69430764650820	16.06994808345924
C	-0.71470199907417	5.10698780659961	15.27486675665739
C	-0.30349493075741	3.23168271910832	13.66006265587886
C	0.08556134411817	2.01298302041700	17.04088627932991
C	0.73401665553940	11.43306071024626	11.05272480391803
H	0.02571443331948	10.84784544846428	13.00227434768380
C	2.69325814446562	10.29668999045770	10.25278913116222
H	3.56246933543207	8.84627891376717	11.58385224026468
C	4.30595677166280	9.16472870815364	20.54133464826382
C	3.85554384722857	11.31730174878272	19.23157284131478
C	4.28251019691579	9.16962362240263	18.01009352513523
C	3.20860909403184	8.97123936029286	21.62301977224104
C	0.66028284541011	8.65871473640743	21.21032321822479
C	-2.56104834790317	8.97446460564043	19.34088457766403
C	-1.80258221024284	10.52646416926315	17.48147258630503
C	-1.56073073188439	8.05112634332361	17.18742591068902
C	-1.83887058332336	8.35182142989140	20.56442914874083
H	6.73210006143160	3.03220386569775	15.57694811703452
H	6.69794714646940	4.26979396241021	16.82065201145531
H	4.91501357339046	6.15467621027362	16.71506857168522
H	4.59863244137266	6.58969124287417	15.02491433231949
H	6.27218718348092	6.28043175247084	15.56203819909677
H	6.45968572676367	4.57185632935137	13.63843122431263
H	4.75512521193798	4.86528929659151	13.19813005956976
H	5.30908693333098	3.20880585167578	13.54982957018613
C	5.39789701281986	2.98685403781894	18.63244867181010
C	5.60462337116085	1.17903981147670	16.91324249729759
H	2.73787789249027	1.54584767271663	17.96928986147588
H	-1.69377831250886	3.23480033180216	16.63563599938495
H	-1.45784872320626	1.94039583519852	15.47409489893603
H	-1.80008330634516	5.10956581262160	15.07750698955834
H	-0.23942919033544	5.80899818193679	14.57801882763976
H	-0.55305108248023	5.48156391548516	16.29513048605312
H	0.11884328899012	2.22614296740732	13.50712457389341
H	0.20694807743961	3.92769238097203	12.98208713395949
H	-1.37117708496490	3.20144252090959	13.38211837548254
C	-0.21988827246742	2.34454916555631	18.52534877317787
C	0.08890216376556	0.47038875345161	16.89488857096651
C	1.68434731508122	11.24570151104210	10.04369489625912
C	-0.40968909781759	12.38764934286616	10.84272865913918
C	3.69106629552100	10.03571420931426	9.15821369804143
H	5.14902841282413	9.77369305695000	20.90283295019934
H	4.72285573870798	8.18203301441418	20.27354975641448
H	3.37923839116450	11.74340197865591	18.33774210195305
H	4.93151662628101	11.55947014671168	19.19710270476959
H	3.42254418358828	11.80734141289111	20.11710778935993
H	4.07486350723661	8.08964523294414	17.96238015115680
H	5.37731420536822	9.30419648435856	18.03992936468766
H	3.90700833332520	9.63412606089497	17.08906667349530
C	3.16640955142897	10.14569684306489	22.63874845673179
C	3.37892652170416	7.63266946718988	22.38440150785562
H	0.49055190443312	8.29940521052847	22.22958033007586
H	-2.98654978611912	9.94766959247672	19.62972197559040
H	-3.40285427787503	8.35880923571433	18.98934384909110
H	-2.83757477679803	10.51987214717775	17.09825902348606
H	-1.12215747261969	10.68229897141942	16.63399664659865
H	-1.69539482192182	11.38496843788060	18.16447757658565
H	-1.33426136560703	7.08558628376282	17.65939657963006
H	-0.83144276736411	8.20652218574397	16.38216604306997

H	-2.56615016200899	7.98453917809599	16.73883369532034
C	-2.38843504000589	8.88666607811766	21.90800921505944
C	-1.89009339997413	6.79818966528952	20.55949608487938
C	5.04730523616599	4.41155765600912	19.05522810443473
H	4.76980954538948	2.27959863959121	19.20346836763263
H	6.44279463431265	2.75548730449927	18.91317951700921
C	5.35499213726374	0.62530708348575	15.51134654540287
H	6.67137789610151	1.05999616394611	17.17866333908077
H	5.04328589995497	0.56873392628872	17.64322686376829
H	-1.17655702587936	1.85726465278806	18.79352691908677
H	0.55176283045038	1.85298935945466	19.14447864435110
C	-0.27138215382802	3.82862795140249	18.88317283455495
C	0.50393504384608	-0.06812653169589	15.52704417465983
H	0.76553685527853	0.05427765368820	17.66258405404907
H	-0.92029729405930	0.09950595201306	17.15200760410245
H	1.64546561392745	11.82875324190450	9.12455949982490
F	-1.55329955191786	11.74142574006703	10.52727235853830
F	-0.17328440868242	13.25946026270695	9.84092797763908
F	-0.67558112373350	13.11387237124755	11.94574475743247
F	4.85295483660039	9.54513677237181	9.62501174235167
F	3.22432017689678	9.13812592819193	8.26093323359328
F	3.98334560127580	11.15280736844068	8.45799530437895
H	3.04604529696651	11.08516840084163	22.07618315478425
H	2.24569279626002	10.03798969877752	23.23911451809501
C	4.36768282555162	10.26670202334245	23.57762980817947
C	3.09098815899615	6.37075151156945	21.57195755282325
H	4.41297649383035	7.59051638908462	22.76843084830915
H	2.73031003937741	7.64499329400040	23.28016166298385
C	-2.15214798899188	10.37233732190201	22.18183639172117
H	-3.47170964414430	8.67837237289934	21.94756545991763
H	-1.94251053158046	8.29560227916853	22.72884405539920
C	-3.26218946936015	6.15547391666268	20.75840884000137
H	-1.45522349156934	6.44056939705826	19.61496513962843
H	-1.20452941345285	6.43921215831489	21.34716556368104
H	5.73459734545942	5.15006497226580	18.61341643863488
H	5.12241338437294	4.51603587807242	20.14904279568650
H	4.02525271130529	4.69546272299072	18.75645242527996
H	5.91377514951257	1.18419657729711	14.74419847442060
H	4.28765728069123	0.68345400575908	15.24688968543363
H	5.66585573912380	-0.43025444902093	15.44349392047162
H	-1.11359952161264	4.33772594441144	18.38842191030113
H	0.64746114977472	4.36588850366429	18.59787924594854
H	-0.41365457887722	3.95909192496551	19.96858279016002
H	-0.17723293961467	0.26898660739963	14.73025873418511
H	0.49953102989703	-1.17056710686776	15.52020002166158
H	1.51734358873582	0.26936679007188	15.25876034878329
H	5.31452683879849	10.35115272082436	23.01965059556692
H	4.45576999563621	9.39863342869807	24.24939503723270
H	4.27824589821828	11.16505545418287	24.21041267883962
H	3.62465643341555	6.36430311264810	20.61105460732870
H	2.02099616310323	6.26959777641154	21.33863112161638
H	3.39717315630036	5.46845456592291	22.12577678321853
H	-2.59096938524164	11.00904203036844	21.39724418477937
H	-1.07798965805156	10.60729978517621	22.22807489707995
H	-2.60750489410637	10.67011690576079	23.14080367775232
H	-3.98881163315950	6.49700199366824	20.00326778363604
H	-3.68602943132646	6.37490101789139	21.75145556671849
H	-3.18623386512669	5.05990714449774	20.66593957338586

$S = ½$

Fe	1.83027145762513	6.87062651348590	16.35391619692274
O	1.74166072465356	6.35751519052893	18.08999703420261
N	2.09188725927663	6.63104698589037	14.47800022191716
N	1.54617722133267	8.76489881913202	16.11558677531727
H	1.68870759718789	6.88461462982927	18.89093037046253
C	2.24789646451545	5.35894482248124	14.00376626463431
C	1.99653721127280	7.49764564540489	13.41032953044999
C	1.43741654091693	9.67563697017148	17.13858699604729
C	1.68510666317372	9.48415235869731	14.92331984306863
C	2.40826487095865	4.29527717130597	15.03027024565080

C	2.23955836258651	5.37954353019801	12.60446721814508
C	1.82201229109995	8.90230727273512	13.61947753403600
C	2.07283186008370	6.72955768008941	12.21845675151235
C	1.54554245552224	10.97570171103690	16.63297623763187
C	1.16590280506929	9.30366726333527	18.54844534955695
C	1.70055806606024	10.86564543004374	15.24099996301476
C	3.67830648110603	4.03716826145715	15.60240246719633
C	1.29220623196296	3.60455861117434	15.56236629467774
H	2.34755260010430	4.51717995802495	11.94825170640346
C	1.77886588322107	9.77642180190999	12.44228179134106
H	1.99922575786083	7.10525208041756	11.20051856536550
H	1.47219930905088	11.88792209453505	17.22293618364103
C	2.21019009784708	9.19134684172962	19.49514176688180
C	-0.15442123737045	9.07632580047469	18.99656768537436
H	1.83586897122633	11.68065821011485	14.53421576340616
C	5.05525378263212	4.53112638082105	15.12963403577218
C	3.79827799040431	3.18905097709327	16.71096137643207
C	-0.15898776740621	3.61913279036807	15.06247514951084
C	1.43759284880253	2.75716842194788	16.66881030933090
C	0.80087185869336	10.78721836090532	12.29955857219634
C	2.69728309526325	9.61855294230918	11.38071605271290
C	3.71574031233430	9.41763919597480	19.29892206540676
C	1.93755136264338	8.84243273687192	20.82776786046184
C	-1.46073233928224	9.08771043282345	18.18701145549625
C	-0.40639630712225	8.72857060264376	20.33303018902685
C	6.03997939380728	3.86066834925091	16.14894928085288
C	5.20623673530484	6.06476971678825	15.16160343562336
C	5.35637877086368	4.02676881956814	13.70567697370771
C	5.22927477445160	3.04314652764847	17.19582012906750
C	2.68196001341834	2.56427435931774	17.26485982272370
C	-0.88717335478806	2.64736725772008	16.05332206842839
C	-0.79579124480486	5.01853603411474	15.12261025794594
C	-0.25291651468939	3.08561281497134	13.61991136912966
C	0.13575255508246	2.09879225720491	17.09171788319556
C	0.74301387215784	11.58345606034981	11.15559564944459
H	0.06911728706234	10.92636739243757	13.09703361413014
C	2.62777963413829	10.41091945944050	10.23476743756925
H	3.48525780735806	8.86988535090794	11.47593301058822
C	4.30708975176971	8.76715067960556	20.58729860429689
C	4.01844175978592	10.92974078638096	19.24664022812193
C	4.32063694567696	8.74953604687777	18.05402740869931
C	3.19549241489426	8.70862161194431	21.67249875903088
C	0.63041304702438	8.61547852516485	21.25777930774370
C	-2.54014560128646	8.98873254002001	19.30973673355924
C	-1.68197462540326	10.37039007964017	17.36764751951253
C	-1.54069851877986	7.87499703726339	17.23962478487493
C	-1.87334458425572	8.41713145951646	20.58718653371055
H	6.74528478997358	3.20631932856331	15.61522146174374
H	6.65527411955463	4.62461336688662	16.64549726159555
H	4.92232362009924	6.47497568530268	16.14156028420688
H	4.57614064558108	6.55173075658183	14.40637331718433
H	6.25737787011723	6.33804030484900	14.96710866987090
H	6.39142952287204	4.28592804404105	13.42415412102781
H	4.67577613719770	4.48213354965656	12.97417295197152
H	5.24881777669313	2.93201982731362	13.64269169431868
C	5.38155407730325	3.61335143222389	18.62879314491786
C	5.65951813203877	1.55450791971845	17.23274266985336
H	2.78511060438440	1.91428898928597	18.13851996835314
H	-1.70497105716147	3.17522218734206	16.56367554125411
H	-1.35898764018163	1.82234783916076	15.49890167015619
H	-1.87115501461328	4.95417550071580	14.88493343513449
H	-0.32849934881769	5.71041770313770	14.40985529551873
H	-0.69800217784780	5.45138230614310	16.12848664533564
H	0.22513738688381	2.09819020908627	13.52635614462246
H	0.23681473209537	3.77318789762518	12.91751087393686
H	-1.31079321820176	2.98468265575524	13.32265670802681
C	-0.24116115848431	2.48139157655983	18.54571985766829
C	0.25656833689182	0.55360264570820	17.03171225220302
C	1.65083256294608	11.40420009723562	10.10381916350607
C	-0.36608860922888	12.59198391843728	11.02544833222583

C	3.59174035593019	10.14507152177915	9.11159611067421
H	5.20443426558846	9.30363090511099	20.93310130277975
H	4.62980785504570	7.74335512461997	20.34390399508144
H	3.58238728250632	11.37472704324360	18.34104297127521
H	5.10833958661592	11.10002394677626	19.22471057006318
H	3.60611591347230	11.45823606043854	20.11964789866299
H	4.03393223026516	7.69029886793064	18.00022905932015
H	5.42145164435760	8.81182685331650	18.10066703118492
H	3.99017854092151	9.23308463359166	17.12587181584976
C	3.27525116559128	9.89947355962205	22.66519469892337
C	3.23046304103478	7.37400592869412	22.46398635247934
H	0.42651307209340	8.32538779012602	22.29262465674611
H	-2.91910342290990	9.99972645058738	19.52367693863898
H	-3.40683136161089	8.39466238023033	18.98214074189615
H	-2.71737585678584	10.38867035350342	16.98569123767545
H	-0.99910380193664	10.43541176112525	16.50946695280342
H	-1.53038339208339	11.26850243781318	17.98829878337373
H	-1.37131916802535	6.93018032734910	17.77334127983686
H	-0.77526183486435	7.94534335433507	16.45666892924762
H	-2.53219716097310	7.83383570181599	16.75827587235181
C	-2.41473714797420	9.06933982824337	21.88062991853978
C	-2.00591105232924	6.87160393483473	20.68682124817846
C	5.00346664474975	5.08344503354679	18.79926776011039
H	4.75863544362498	3.00239576291653	19.30611511548467
H	6.42722139371148	3.45346695689359	18.95247513933886
C	5.44592312125761	0.76590852691838	15.94160389615825
H	6.72578460323476	1.51497743874886	17.52206696200593
H	5.10885760370121	1.05607710576178	18.05045080515057
H	-1.15913910201397	1.92611288538110	18.81640778995642
H	0.55063559491832	2.09300865355433	19.21119397818494
C	-0.43584969117346	3.97104526186982	18.81727911666173
C	0.74487421312405	-0.02884064380008	15.70654445016023
H	0.94180837473737	0.23299306915046	17.83657191510143
H	-0.72900857088994	0.12347910343190	17.28768641782779
H	1.60756080623622	12.03309292300380	9.21624439011492
F	-1.54682278427017	12.00791396700294	10.72289799236304
F	-0.12643930275896	13.49625119915030	10.05324477530841
F	-0.56405118946513	13.28219248949107	12.16505907026483
F	4.84961926119882	9.95109672752531	9.55468268402174
F	3.25527022585066	9.03555068335485	8.41703041620715
F	3.63785785587529	11.15562355870676	8.21977182525461
H	3.25387988850704	10.83567499916877	22.08548265073581
H	2.34972695399653	9.90087747412552	23.26821411626917
C	4.48457664978130	9.91005423512980	23.60159828492775
C	2.78017692447519	6.13251299481801	21.69200167894671
H	4.26117798298612	7.22068730649673	22.82843528497483
H	2.60731667542280	7.48104314393267	23.37111044589715
C	-2.09901881809056	10.55466543927604	22.05975135782437
H	-3.50849018066617	8.92394475261227	21.91360400482457
H	-2.01454906781117	8.51125718446486	22.74698935365332
C	-3.41318036514422	6.31482056910914	20.90060826749272
H	-1.57285988153253	6.43016457092481	19.77779634151637
H	-1.35438814794908	6.53432228546475	21.51223086690456
H	5.64688038261305	5.74458743266729	18.19946479762950
H	5.11912937252963	5.38992039768612	19.85026026592533
H	3.95990851430038	5.27736500904842	18.50292458617859
H	6.00986788582218	1.19642908835243	15.09922538065907
H	4.38304350470065	0.75384234877545	15.65405038548586
H	5.77696425263281	-0.27887512128088	16.06033645231667
H	-1.30717151491150	4.37034817736174	18.27409401981366
H	0.43566144857915	4.57717796377594	18.52324763660514
H	-0.61972866494136	4.14689270131259	19.88968411768147
H	0.05724389203784	0.20321363549018	14.87859711719909
H	0.82927352123008	-1.12629439994261	15.76715042058292
H	1.73407811828487	0.37309920270402	15.43724992338916
H	5.43345600432260	9.87732641469974	23.04167518607167
H	4.47910312199349	9.05264334995716	24.29272686889289
H	4.49603487586802	10.82652727759003	24.21433374822882
H	3.28958211016635	6.04444432523304	20.72288956035433
H	1.70162551401907	6.15176228709954	21.48017690961295

H	2.99479057354104	5.21515450735371	22.26430942515725
H	-2.50065063962675	11.16253855989428	21.23342169680744
H	-1.01355620412080	10.73280946615217	22.09719305657353
H	-2.54003278843428	10.93811990941370	22.99474072752303
H	-4.10405815134580	6.62758987247916	20.10087736051850
H	-3.84772140991827	6.63683184765138	21.86050796825441
H	-3.39213378108742	5.21282491987837	20.90048976951591

S = BS(4,1)

Fe	1.86248344027366	7.11244288339249	16.59763959300903
O	1.94465879106646	6.21805317841689	18.21800890501064
N	2.17520843512965	6.66414915310037	14.67718586097028
N	1.46109980439590	9.02943249841125	16.17387996894172
H	1.75104249857402	6.75946335713045	18.99245139690162
C	2.31986313404293	5.38957205473612	14.20140258136135
C	2.04801205237954	7.52708605213680	13.59193961237688
C	1.32424888980318	9.99852585298768	17.13030594635977
C	1.63916330333228	9.65077145523558	14.94284652301238
C	2.43012274761122	4.25917489538710	15.16049587512986
C	2.29199115387469	5.40218252091050	12.80150405971818
C	1.84320247600572	8.94489029768223	13.70681938909413
C	2.11302449316607	6.74206675380078	12.40851927386891
C	1.42093047715205	11.26399730075914	16.53883698668081
C	1.10699215774481	9.60538262963086	18.54689521636130
C	1.61914324824225	11.05456248622274	15.15923743783061
C	3.67231237294605	3.89899508961355	15.73449453719714
C	1.27773878928111	3.55300259828786	15.58241575509338
H	2.39664223887815	4.53427858591307	12.15217997737130
C	1.80993531908295	9.72665095314599	12.46124448645459
H	2.01161204289677	7.10405058438473	11.38852956349565
H	1.32607470938739	12.21946258173133	17.05299999391367
C	2.18720273571181	9.51870479385368	19.45600016649968
C	-0.17413134178846	9.22767335255350	19.01266981880165
H	1.75587923636352	11.82343843966210	14.40206136029129
C	5.06356980898118	4.48551060766611	15.44855647019725
C	3.74361738075918	2.88457181731999	16.69841517217627
C	-0.16931259904800	3.69619661933502	15.08440881472173
C	1.36923914184062	2.57115584299917	16.57797579200166
C	0.78068394561547	10.65824428274357	12.20495932118656
C	2.79022060360441	9.56289037762115	11.45475586485883
C	3.66151500151937	9.88009899872010	19.22878799534346
C	1.98688379372906	9.06951516683745	20.77104352786755
C	-1.50883193262499	9.20742492040588	18.25054423296865
C	-0.35018010055614	8.76546230736900	20.32682294033168
C	6.01028666983742	3.59988307591178	16.32983543189547
C	5.17000740008822	5.96187504002728	15.87761043284912
C	5.44971880960981	4.36053379286240	13.96359680090508
C	5.15878522310513	2.60461253563427	17.16995873017377
C	2.59651875848030	2.23054725080940	17.14126683034503
C	-0.95852930574556	2.68276523788228	15.98295070163348
C	-0.72412117211426	5.11932934337406	15.26854305781413
C	-0.29964027436276	3.29099439941549	13.60334532731165
C	0.02588534534338	1.97527904101549	16.95790876955108
C	0.72680344999128	11.37137690629699	11.00626820806050
H	0.00341361077507	10.80278915178870	12.95607152294838
C	2.72986247494519	10.27629547399649	10.25948252224174
H	3.61487603747584	8.87311370651738	11.63000470300495
C	4.34430091429214	9.27590058653188	20.49392656556637
C	3.83712376638727	11.41192242663042	19.17798111749548
C	4.28072704827088	9.26392863821113	17.96342068686208
C	3.26701139908177	9.06674747856417	21.59225266077468
C	0.72207466439641	8.68468091357004	21.21336537939843
C	-2.53292652978382	8.93262140855455	19.39507530806366
C	-1.83561045385394	10.54155008736296	17.55990961031045
C	-1.55466963085706	8.07775441581672	17.20291841858872
C	-1.77490016838194	8.30657327171383	20.59457054252749
H	6.71518973636291	3.05121367999277	15.68726296711392
H	6.62945213024053	4.23134792772633	16.98376495618972
H	4.83113091041565	6.09417130212427	16.91501656911258
H	4.55796484555095	6.61198232690102	15.23997523471046

H	6.21890984186162	6.29752307166321	15.81027121996066
H	6.49637835734864	4.67848354917561	13.81746317328954
H	4.80684562552978	4.98747560853784	13.33104178923494
H	5.35658060688692	3.31781665167062	13.61941375098712
C	5.29270408069989	2.84932067870744	18.69295697732796
C	5.55298745466211	1.13102236527617	16.89113623401108
H	2.65762133981191	1.45943820161742	17.91465378986922
H	-1.74599002137755	3.20608588326489	16.54375198285081
H	-1.47752457094064	1.94453306657441	15.35327339881184
H	-1.80607502252308	5.13546714227327	15.05376609093734
H	-0.23303844669941	5.83831351056248	14.60052774499284
H	-0.57595851826153	5.46194297184830	16.30210597368118
H	0.12337625229223	2.28954543767331	13.42641915085534
H	0.22009307571432	4.00554893664255	12.95241400895911
H	-1.36382064299627	3.27035961331009	13.31153149203299
C	-0.30231170496877	2.28163436885937	18.44278800593257
C	0.02078986314727	0.43600544830314	16.78101253050944
C	1.69814760439800	11.19202914741383	10.01657049769172
C	-0.44161812068270	12.28507740987437	10.75699989442663
C	3.74722013502430	10.02228526160581	9.18174128087999
H	5.18144558108916	9.90169071743463	20.84012511462985
H	4.77485633511442	8.29968246538471	20.22452163564195
H	3.33765627386065	11.82409069710271	18.29038415340545
H	4.90815165771885	11.67139258571418	19.12248548933788
H	3.41228289704111	11.90037360762686	20.06830413827496
H	4.08500377121150	8.18153678908196	17.92006995500674
H	5.37439323290013	9.41007950110450	17.97914594491468
H	3.88886224676126	9.72019272242249	17.04520352681216
C	3.20932726281243	10.24971154932020	22.59766934777616
C	3.47959795255855	7.74082141606011	22.36373098126090
H	0.57637362034863	8.31575797444952	22.23286921031047
H	-2.97574527577156	9.89010625501029	19.70906284818519
H	-3.36607327176970	8.30356803039226	19.04682975710777
H	-2.87211883394606	10.52144347059745	17.18131699254929
H	-1.16227865178781	10.73505053234972	16.71448452707548
H	-1.74505602968325	11.38300716291550	18.26628484392231
H	-1.29301715446189	7.10756877996451	17.64626472423152
H	-0.84973554829242	8.26836779293846	16.38368065677276
H	-2.56903174443903	7.99736181451182	16.77750185376872
C	-2.32321183671325	8.79072971161820	21.95743408862922
C	-1.77344168180663	6.75223006750814	20.55128258421250
C	4.89868659435323	4.24141777704333	19.18130827744283
H	4.66736016815713	2.09975882656201	19.21043318169594
H	6.33540463492053	2.62604998596634	18.98767643403444
C	5.35120590741682	0.65069136309793	15.45486044423326
H	6.61039847875951	0.99810894921160	17.18593445111306
H	4.96771531400370	0.48389039170457	17.56863070407418
H	-1.26347148226180	1.79134591853841	18.68854718675982
H	0.45967328640276	1.77890833638835	19.06485744381352
C	-0.35910891648153	3.75966810896505	18.82507742481624
C	0.46710157134633	-0.07723784199408	15.41320917603629
H	0.67394219657452	-0.00171122699990	17.55702751318269
H	-0.99778274890588	0.06905408630786	17.00437061643204
H	1.65672257461917	11.75511510728774	9.08513337605482
F	-1.56244947788437	11.59574784642858	10.45127000428720
F	-0.22034559874123	13.13214907052845	9.73087530753912
F	-0.73919763334669	13.03648781982269	11.83485027633620
F	4.90318441279488	9.53503129320032	9.66543060785292
F	3.29736185825189	9.12577018739862	8.27481105291062
F	4.04580650308471	11.14249291373867	8.48893274081179
H	3.05395994957840	11.17988897851717	22.02836552016304
H	2.30157978726507	10.12275879015615	23.21382462067106
C	4.42173124498810	10.41363511989578	23.51537207272596
C	3.22562272661487	6.46446371990302	21.56337077575976
H	4.51585931659201	7.73174841586774	22.74411552039512
H	2.83415282206757	7.74222166180069	23.26184675914414
C	-2.13271012275248	10.27690796998558	22.26220299157290
H	-3.39808924041937	8.54439084783593	22.00680005342026
H	-1.84580337179898	8.19638056767190	22.75792606451786
C	-3.12154026938611	6.05858145995205	20.74077782536130

H	-1.33092128012067	6.43363913763376	19.59627577756519
H	-1.07105860247354	6.39797460410729	21.32613810773708
H	5.57933302078070	5.01825160422314	18.79897727500127
H	4.94130528793560	4.28872887562405	20.28061514420044
H	3.87947259777545	4.51975801115618	18.86874057269242
H	5.94328755130628	1.24022580362150	14.73723686803914
H	4.29510440566253	0.73383895123011	15.15430333370531
H	5.65468487736449	-0.40355573839452	15.34578410491268
H	-1.19996453743053	4.27493142736636	18.33463559671418
H	0.55942977882797	4.30586327439439	18.55587555921968
H	-0.50802924443539	3.87057258792203	19.91169335614749
H	-0.18507230409806	0.28930754270274	14.60512976042321
H	0.44620335034269	-1.17901648790955	15.37995785640945
H	1.49278473881653	0.25056444172088	15.18249472277861
H	5.35646630888606	10.51952677081215	22.94095030983180
H	4.54571054094252	9.55518864441739	24.19385748652737
H	4.31699901306876	11.31529803160472	24.14104096502448
H	3.76384936976061	6.46116329352490	20.60478546945685
H	2.15966404293701	6.33530296763835	21.32611962297129
H	3.55044992918540	5.57575597060500	22.12812317010050
H	-2.60282998381155	10.91568838497245	21.49774936623254
H	-1.06620832853678	10.54593508849671	22.29914599756900
H	-2.58394248268065	10.53837973963170	23.23360953996950
H	-3.86374831267009	6.39526033573677	19.99874450555610
H	-3.54685496745423	6.23659310668345	21.74154418091705
H	-3.00882015629156	4.96931350799361	20.61790509321466

Table S20. Coordinates of the optimized molecular structure for the (^{Me}L)Fe(OH).

$S = 2$

Fe	1.86453499044337	7.12763872550713	16.65415607808878
O	2.00472583260026	5.97332944733235	18.05323363476116
N	1.55352715529924	9.05628132877525	16.25464876244320
N	2.07448289722070	6.61193103722589	14.71957529030802
C	1.76804495371709	9.72316562520919	12.49979349224023
C	1.33300029687020	11.00686445598655	15.09679320738055
C	1.23930325324461	9.79305563456832	18.60499573721142
C	1.17285611939521	11.28056087144274	16.45194896475437
C	2.24544567595443	6.75888724716982	12.44665137347356
C	1.79023776368564	8.89411496613001	13.76582071122842
C	2.42261985122204	5.43676023762469	12.81306311990872
C	2.39893625526269	4.17600792777341	15.10382203336190
C	1.57196543527178	9.60862152096881	14.97001465867706
C	1.31649676755837	10.04898851545916	17.13351315974083
C	2.02262130614628	7.51243037519207	13.64542786693219
C	2.30702836233883	5.38539707980750	14.23207753056939
H	1.28381859370842	11.72590750756576	14.28207680905324
H	0.97470806559265	12.24574805815930	16.91522104676618
H	2.27348227921636	7.15201869738127	11.43339907457714
H	2.61437044100874	4.58742486992606	12.15927719619860
H	1.94008181037608	6.19186168207456	18.98688088888970
H	1.90818198572750	9.12175368431728	11.59689094915040
H	2.56222299212987	10.48550505680083	12.52636165405494
H	0.81029124743095	10.25709248101852	12.40507078279290
H	0.48836746960870	9.01927077033073	18.83876813074446
H	0.96497644181517	10.70763401519230	19.14900866386956
H	2.20647173911906	9.44004516855819	19.00301973562475
H	3.30198202993410	3.59142306943855	14.86444873323530
H	1.53365373126955	3.51187660341790	14.93456470048526
H	2.42124425683199	4.43818333964532	16.17200601347672

$S = 1$

Fe	1.86912015484430	7.06173661079362	16.55117924744654
O	1.95441629287784	6.55144539202272	18.25191797000512
N	1.55062017842707	8.91952441852168	16.18330458018096
N	2.05001652616643	6.66476809347219	14.67356348379597
C	1.84853354107240	9.76991417392597	12.46422556165902
C	1.34622317621538	10.94095643581830	15.13665607567444
C	1.16353002148302	9.57313386363493	18.57546540820844
C	1.14796368782285	11.12919394646992	16.49709990831139

C	2.27571017975310	6.75413495755095	12.40525186767642
C	1.83427761022448	8.91147525657096	13.70629341330265
C	2.41072284029820	5.43089148926497	12.80718007061768
C	2.30604092121524	4.23365736360147	15.13422278044970
C	1.59612292714009	9.55417559076603	14.93494789112262
C	1.28283296372611	9.85957749337981	17.11505064145320
C	2.04708238098926	7.52919437562822	13.58080662963195
C	2.26354948250277	5.41484167788005	14.21783201287634
H	1.31320224069086	11.70260633376547	14.36045061299488
H	0.92782483179293	12.06101425105687	17.01535966511127
H	2.33151882534645	7.12891672053419	11.38583203283851
H	2.59348498780064	4.56125477912330	12.17817328874156
H	2.34394329757738	6.84616658862823	19.07921965755059
H	2.04579018837438	9.18853713854140	11.55748074388423
H	2.62108188271781	10.55064882290825	12.54221079135792
H	0.88219685675237	10.28220150147429	12.33652318209811
H	0.52036195735251	8.70156848880899	18.76590098211041
H	0.75386523781056	10.44758518976952	19.10077320881783
H	2.14931697360739	9.34479079264590	19.01504420655360
H	2.60640666852260	3.32781377561242	14.58950784233884
H	1.31759267094870	4.04217810418696	15.58692838809406
H	3.01633049594666	4.38587637364148	15.96412785509386

S = 0 (open shell)

Fe	1.79798397984732	7.06823033355874	16.56627866790333
O	2.05971512592974	6.51236147892508	18.23966724616784
N	1.51934138475859	8.93421467601219	16.18598078847054
N	2.02708880320241	6.66055237682298	14.68844661294493
C	1.85451367176483	9.76721868630334	12.46973712024166
C	1.33502647794653	10.95415743864465	15.13369658077387
C	1.13656545112938	9.58791690910027	18.57414662615838
C	1.13069861450723	11.14782733192066	16.49337705770736
C	2.28909775532239	6.75892635208601	12.42246642105570
C	1.82521369613807	8.91332483834917	13.71513445563739
C	2.42477053373056	5.43591923250357	12.82106901229438
C	2.27268134788306	4.22347221550201	15.13340283631949
C	1.57801880116591	9.56514520113456	14.93783640981815
C	1.25282093644237	9.87880332728647	17.11414224390763
C	2.03846401515391	7.53033370739218	13.59692276093293
C	2.25352817050723	5.41381084548480	14.22963805315600
H	1.31080690553437	11.71417752769239	14.35544049597766
H	0.91549981654802	12.08337693920351	17.00703063922440
H	2.36265419363988	7.13799369750107	11.40571479841190
H	2.62438838997478	4.57044286947197	12.19137427723833
H	2.62177428980511	6.75456207718046	18.98120683407006
H	2.03284907747515	9.18002359213290	11.56292208501950
H	2.64595854353234	10.52964003740029	12.54221493530894
H	0.90053967916090	10.30265053079251	12.34524400055782
H	0.51493121884073	8.70097460754428	18.76547029984314
H	0.70694617752329	10.45117137140512	19.10201618431707
H	2.12645457663052	9.37924489968345	19.01443032219267

S = 0 (closed shell)

Fe	1.61999517184885	7.11661469197386	16.57359000451838
O	2.82252460521541	6.69431804391378	17.77720474268750
N	1.40693682622565	8.94888594395378	16.21103046579763
N	1.92377227052752	6.64434902462558	14.77479290635774
C	2.00348582690742	9.74384671294265	12.52956527835423
C	1.36852143059832	10.97710352620508	15.16797344760212
C	0.91205894050925	9.55763627897245	18.57218523959261
C	1.08785610781436	11.16998989785870	16.51451967146907
C	2.28508829847676	6.71590916638464	12.52251144092152
C	1.86178584414804	8.90312249531167	13.77582252059706
C	2.34401390553543	5.39270332978130	12.93386823198233
C	2.07201915935284	4.20747843758168	15.25391529174421
C	1.57022158204638	9.57823232663209	14.97582835344064
C	1.12079812382944	9.89118499853685	17.13036069797386
C	2.02036013535196	7.51010261872975	13.68111028253233
C	2.11491154741662	5.38601788923957	14.33591677419762
H	1.41832265898113	11.74136792085875	14.39511558040076
H	0.87788515032130	12.10994725447601	17.02231067444264

H	2.41075281037386	7.08389688536099	11.50693459061572
H	2.52902540921107	4.51343696709740	12.31905283131332
H	3.74490193958800	6.97542001694157	17.75928959589972
H	2.20555067574311	9.14224075821644	11.63730967089228
H	2.82611452144434	10.46716096114244	12.64528957992766
H	1.08606081295549	10.32502479756092	12.34833475271144
H	0.10524814630691	8.81660718773664	18.70186829972362
H	0.64302361882944	10.45599315683630	19.14516537513980
H	1.82097024695463	9.11688806485491	19.01380046733344
H	2.56277377816505	3.33755677981177	14.79416274676682
H	1.03170754680450	3.91682326376227	15.48389725344944
H	2.57299290851673	4.42992060269926	16.20980323161286

Table S21. Coordinates of the optimized molecular structure for the (^{Me}L)Fe(OH)(thf).

Fe	6.88761119383309	16.56080235346060	2.09822849761989
O	8.90447597766294	16.41824553866059	1.10276749710101
N	6.56277261380000	18.42988040026200	1.35758038271751
N	7.43915465454222	17.33567310658336	3.92617830925559
O	6.45210010340787	14.76708654838489	1.96801590328910
C	10.61806617579463	17.22454061723429	2.52096868420638
C	10.51453661522729	15.68361513083497	2.66942675279633
C	9.66291843318427	15.24721178315464	1.46857066822357
C	9.81079395911068	17.52833080703341	1.25090987869204
C	7.72217941129062	21.14231048180387	3.80271282557347
C	5.70148706682621	17.61931429523813	-0.81644384273839
C	6.09865872456959	20.10842034185518	-0.09564163135027
C	6.58743557122300	20.69434776180916	1.06346295024784
C	7.41485855800859	19.75139924107798	3.28768976015565
C	8.20086984625535	18.80430501825062	5.50809572520269
C	7.61035261609092	15.16193258285739	5.12026149926768
C	8.26285439492219	17.52602299103836	6.03340385213920
C	6.10398932282984	18.70583657949293	0.12806757286104
C	6.88245780769024	19.63765501281630	1.98320978019593
C	7.67465392560882	18.69129388624583	4.17867719469385
C	7.77405050749695	16.64455784097921	5.02131280269815
H	10.45842281238788	17.56524885575379	0.35619904535210
H	9.21174688469917	18.44551802406867	1.29726668771325
H	10.17473766983421	17.72773753261839	3.39081365888832
H	11.65794089657627	17.57026106939898	2.42350056684686
H	10.01504845337997	15.42240444273707	3.61162937484222
H	11.49729210973437	15.18940875520762	2.66330970869254
H	8.93885008140033	14.44916062327771	1.67620371263446
H	10.29420856991464	14.96220959509053	0.60738948245884
H	8.52400255723765	14.68806695705051	5.51579808483406
H	6.79536114340482	14.91091341584733	5.82159783755089
H	7.35907213447385	14.71186216139896	4.14869947767458
H	6.56520782157758	16.98578915383652	-1.08248788769218
H	4.94352071196652	16.95932059540254	-0.36173865545707
H	5.28312517417563	18.03385585380365	-1.74435226914019
H	7.02957397021159	21.41302463751619	4.61600159059144
H	8.74031094305272	21.18968384711103	4.21558360598505
H	7.64241658430912	21.90643629573704	3.02346978308053
H	5.77013573438875	20.60907202180397	-1.00512633367986
H	6.71173734858760	21.76005716181658	1.23966722049392
H	8.48572843374449	19.72626478658942	6.01043527489750
H	8.60336665260101	17.23091271713913	7.02499093525495
H	5.89820583296606	14.39454917771946	1.27639403532938

Table S22. Coordinates of the optimized molecular structure with solvation model for the p*K*a calculation.

[(^{Me}L)Fe(H_2O)]$^+$

Fe	1.861118	7.099190	16.519076
O	1.938175	6.146542	18.304910
N	1.559497	9.004842	16.231509
N	2.089205	6.577882	14.650685
C	1.763230	9.734115	12.482011

C	1.349608	10.973056	15.107361
C	1.260231	9.708933	18.598660
C	1.197796	11.224432	16.469139
C	2.241513	6.773137	12.385877
C	1.789776	8.887624	13.732156
C	2.430353	5.438701	12.726397
C	2.457470	4.143604	14.996416
C	1.578068	9.578456	14.949422
C	1.333492	9.985803	17.134326
C	2.024724	7.503181	13.591783
C	2.329599	5.353689	14.134354
H	1.303167	11.705023	14.303743
H	1.009692	12.183008	16.950418
H	2.256588	7.186654	11.380410
H	2.620342	4.603225	12.054513
H	1.895461	9.147837	11.568063
H	2.561063	10.492460	12.517396
H	0.808075	10.275200	12.403233
H	0.486356	8.955368	18.827648
H	1.009659	10.621964	19.156103
H	2.226029	9.338577	18.986471
H	2.629405	3.245225	14.388235
H	1.542765	3.980772	15.592885
H	3.301895	4.243566	15.700915
H	1.998857	6.603950	19.162033
H	2.232521	5.228184	18.437062

$[(^{Me}L)Fe(OH)]^{+}$

Fe	1.796140	7.095731	16.643779
O	1.759388	5.921331	18.049765
N	1.552762	9.043115	16.251096
N	2.097905	6.615581	14.714653
C	1.733433	9.720602	12.493061
C	1.332442	11.001134	15.099566
C	1.257605	9.779948	18.606386
C	1.187468	11.270364	16.456954
C	2.275713	6.766687	12.442644
C	1.777659	8.894525	13.757730
C	2.487019	5.446060	12.812757
C	2.489131	4.195457	15.110995
C	1.564300	9.600714	14.966403
C	1.329204	10.033935	17.135983
C	2.030613	7.512350	13.639200
C	2.366410	5.392348	14.227423
H	1.282259	11.721274	14.285221
H	1.001061	12.235176	16.927329
H	2.299204	7.162103	11.429791
H	2.705955	4.599986	12.162642
H	1.620255	6.236208	18.950506
H	1.873889	9.120464	11.588955
H	2.515500	10.495837	12.510620
H	0.767489	10.241608	12.406486
H	0.467231	9.049228	18.849858
H	1.041911	10.708426	19.153824
H	2.208507	9.371906	18.990730
H	2.856895	3.327851	14.545557
H	1.514382	3.925220	15.552373
H	3.177950	4.384612	15.950243

$[(^{Me}L)Fe(O)]^{-}$

Fe	7.085670	16.424097	2.305025
O	6.947248	14.898535	1.537002
N	7.436608	17.316275	4.131866
N	6.876391	18.310284	1.425679
C	7.656605	16.675089	5.291694
C	7.484701	18.690487	4.387506
C	7.012850	19.550026	2.063631
C	6.598759	18.534654	0.137313

C	7.855172	17.608309	6.346013
C	7.669399	15.182497	5.383171
C	7.749536	18.874144	5.780016
C	7.295513	19.721357	3.432370
C	6.801717	20.575601	1.078773
C	6.542407	19.939870	-0.122644
C	6.386912	17.424873	-0.840054
H	8.052102	17.359333	7.388887
H	7.847930	19.827641	6.295890
C	7.395560	21.135468	3.962738
H	6.835263	21.650460	1.244802
H	6.333539	20.401716	-1.087825
H	7.328288	21.890644	3.173127
H	8.348354	21.287823	4.492315
H	6.591078	21.334606	4.689239
H	5.383964	17.490015	-1.297340
H	6.492020	16.445270	-0.342546
H	7.114746	17.485538	-1.668137
H	8.385030	14.742268	4.668787
H	6.680910	14.755744	5.140616
H	7.943755	14.853362	6.396420

$[(^{Me}L)Fe(H_2O)(I)]^+$

Fe	8.343908	9.279896	5.359741
N	7.784337	8.626494	7.082841
N	8.747360	11.094356	5.891602
O	6.739364	9.340082	4.074893
I	10.091649	7.937975	4.140624
C	7.337746	7.383591	7.418131
C	7.911822	9.388127	8.259495
C	9.144975	12.132109	5.108157
C	8.723719	11.527576	7.232388
H	6.874808	9.191289	3.121654
C	7.104330	6.321020	6.401962
C	7.158720	7.314246	8.811558
C	8.348140	10.730716	8.332773
C	7.517288	8.557984	9.338277
C	9.252050	12.018616	3.627752
C	9.388237	13.260912	5.914675
C	9.130750	12.889647	7.233763
H	6.802797	6.442414	9.357916
C	8.402923	11.340149	9.710882
H	7.496762	8.847694	10.386816
H	9.713685	14.235078	5.553678
H	9.219973	13.529198	8.108740
H	8.761929	12.373585	9.703523
H	9.071515	10.753792	10.359967
H	7.405569	11.326409	10.176975
H	8.251783	11.975265	3.161517
H	9.794700	11.105562	3.333209
H	9.775737	12.889423	3.210738
H	8.041481	6.055858	5.884368
H	6.390272	6.654044	5.629819
H	6.700778	5.416550	6.876393
H	6.055555	10.027693	4.171194

$[(^{Me}L)Fe(OH)(I)]$

Fe	8.239071	9.299403	5.285525
N	7.742097	8.653989	7.082536
N	8.670220	11.130716	5.898238
O	6.908160	9.184806	4.047860
I	10.385200	8.112147	4.425492
C	7.252204	7.436547	7.404673
C	7.928708	9.385884	8.263575
C	9.040578	12.170291	5.122447
C	8.744544	11.527190	7.242772
H	7.080138	8.765773	3.193251
C	6.969132	6.398083	6.373065

C	7.102214	7.341603	8.808405
C	8.402944	10.714837	8.342550
C	7.526146	8.554308	9.346040
C	9.092827	12.072629	3.636319
C	9.370021	13.280664	5.938002
C	9.189027	12.883326	7.257564
H	6.724893	6.472641	9.345598
C	8.518249	11.303122	9.728193
H	7.541767	8.828602	10.398940
H	9.700590	14.253309	5.576674
H	9.349174	13.495145	8.142394
H	9.009833	12.281093	9.732609
H	9.089402	10.634334	10.388750
H	7.518441	11.424203	10.175846
H	8.141516	11.694805	3.227375
H	9.882586	11.370672	3.317348
H	9.303305	13.053429	3.188301
H	7.904932	6.031780	5.916355
H	6.350402	6.804583	5.556531
H	6.445661	5.540157	6.817152

[(^{Me}L)Fe(O)(I)]$^-$

Fe	8.134011	9.294288	5.185600
N	7.692175	8.654040	7.074209
N	8.633582	11.157414	5.877325
O	6.933642	9.135584	3.984526
I	10.521451	8.077293	4.587958
C	7.225216	7.441784	7.410909
C	7.911167	9.381935	8.247775
C	9.021595	12.195506	5.122807
C	8.723938	11.533928	7.223508
C	6.930696	6.398957	6.384190
C	7.117566	7.335782	8.825666
C	8.390478	10.709995	8.318905
C	7.547892	8.547819	9.350416
C	9.082410	12.110809	3.634228
C	9.378809	13.297511	5.950252
C	9.194993	12.886705	7.262032
H	6.764445	6.461470	9.371852
C	8.536101	11.287136	9.709510
H	7.594348	8.821559	10.402817
H	9.728087	14.268223	5.599563
H	9.370084	13.483473	8.154576
H	9.013324	12.272164	9.711938
H	9.136223	10.621360	10.347511
H	7.548966	11.389221	10.188937
H	8.196439	11.594996	3.230901
H	9.965395	11.531459	3.310926
H	9.152379	13.111922	3.184257
H	7.861923	5.918093	6.035269
H	6.448187	6.842471	5.498398
H	6.279419	5.612968	6.794178

Table S23. Coordinates of the optimized molecular structure for the transition state of the reaction between (^{Me}L)Fe(OH) and CO_2.

$S = 2$

Fe	1.92073687446477	7.05237506782905	16.87017307140689
O	0.54352402461359	5.48178208533596	17.78819784060602
O	1.37019075846613	4.52681323105676	19.72526948073097
O	2.52166485448731	6.52427190565542	18.63363512463449
N	1.53107870878161	8.97347304992577	16.42518524642382
N	2.13001998050826	6.49396801650465	14.96167259013282
C	1.71050231261735	9.52535767329074	12.64956562267090
C	1.27163477965034	10.88572574663581	15.20520273071392
C	1.11608031165322	9.77234299061990	18.74139324628466
C	1.09471391945945	11.19447816328143	16.54529957295773
C	2.24327573622959	6.57401063172852	12.68552614022240

C	1.77064377088968	8.74191950950793	13.94389010408789
C	2.46242078161462	5.26422066690438	13.08851468894987
C	2.53054669496369	4.08683286960843	15.42369716536813
C	1.54521519684236	9.48562323257969	15.12189527018721
C	1.25976813398201	9.98263300384949	17.26899118913000
C	2.03105984676278	7.35646485398797	13.86285525883668
C	2.38439273061843	5.25560568165579	14.50413699193808
C	1.17447227077895	5.18574586008151	18.77473815643572
H	1.21494861958137	11.57792199705089	14.36806391842100
H	0.87084636279785	12.16775000095306	16.97907854860556
H	2.23535663228113	6.93492211830074	11.66026496612078
H	2.65858462661720	4.39957092095051	12.45680301996241
H	3.37945574380642	6.28781763705471	19.00265015756100
H	2.94935027800054	3.21988261205288	14.89415512568065
H	1.55572405240741	3.79157523148617	15.84690532259481
H	3.19381794981861	4.32401616160941	16.27233198778785
H	1.80576508828364	8.99836799750608	19.11157188802483
H	0.09240868479996	9.43903267924569	18.98909516076421
H	1.30076548323112	10.70632940420808	19.29173679330527
H	1.92401186975310	8.90918859112431	11.77146020303751
H	2.43659362481808	10.35224518666620	12.66522945305265
H	0.71297929641894	9.97192522175036	12.51572396335952

S = 1

Fe	1.70816074932654	6.92806346640138	16.82878436409294
O	0.60760992172572	4.94050845660921	17.10490030166192
O	-0.25381022304323	4.50924360361835	19.22337883297812
O	1.48105931040985	6.79206271316890	18.63663948784063
N	1.77657269535369	8.83808555896351	16.49121211236015
N	2.11689887833304	6.51052321012379	14.96812831487702
C	1.16068884612673	9.46532409550339	12.75679720162341
C	1.38495040585622	10.79954054160338	15.37224395319061
C	2.17430630102994	9.72860781509674	18.82119230488598
C	1.62946175975319	11.09554585621517	16.70452128659126
C	2.01242670910488	6.56974941811102	12.68457107658373
C	1.47187733617751	8.68060234135439	14.01009489504434
C	2.48422377607198	5.32485043831188	13.06955015164153
C	2.97880903815041	4.20185042670636	15.37083806476724
C	1.50049720973938	9.38881364742420	15.22837418873706
C	1.86221224251534	9.86395763045629	17.36632058144811
C	1.80321879970049	7.32425722260050	13.87950690594253
C	2.53123387323866	5.32436571666851	14.49161018955599
C	0.20924725267521	4.79058792019871	18.20365835089061
H	1.18077458082629	11.50870822361124	14.57334735306968
H	1.65603649292443	12.07694412905572	17.17538025011396
H	1.86376913834569	6.92163719429765	11.66622560276819
H	2.77940379769497	4.49805299830965	12.42531505406219
H	1.52742608416455	7.50626976446962	19.27347890206119
H	3.29919042294451	4.56479080100705	16.35894430198669
H	3.82450296094281	3.66810785073971	14.91082135043694
H	2.17153495595476	3.46756189562259	15.52912432422634
H	1.27414591932502	9.46303818403917	19.40340871431242
H	2.53616780677706	10.68918263849805	19.21566183627165
H	2.94452943513926	8.96587633216281	19.00491157470874
H	0.91781278737822	8.80948606028980	11.91329716790668
H	2.02117733343338	10.09018231607605	12.46584008203637
H	0.30643340190309	10.13781153268350	12.91683092132234

S = 0 (open shell)

Fe	1.89199087445504	6.93285272365059	16.78165887407791
O	0.83103039248810	5.16480088470669	17.56929886657603
O	0.85319590212222	4.96227142794960	19.88148164475557
O	2.36337713638240	6.72518122054697	18.57720814663716
N	1.60090854747471	8.82402737975177	16.42556234926446
N	2.10219595757746	6.49007835418881	14.89936567079259
C	1.62228749808366	9.55628406408997	12.66839906905721
C	1.27592525516624	10.79374814867607	15.30382635048941
C	1.32763740468871	9.60569251908700	18.80574160580279
C	1.16072527339649	11.04091899146528	16.66337732563067
C	2.23323508164284	6.59957581024154	12.61944841590204
C	1.72485162592158	8.72677659904880	13.92804560306790

C	2.49922735626104	5.29705054068920	13.01001999847431
C	2.62305220838337	4.07761528652839	15.30684944237229
C	1.54824715519449	9.40490317101540	15.15059485184899
C	1.36497124782135	9.80482708438142	17.32581660918010
C	1.99300254633129	7.35491942472472	13.80816954714654
C	2.40938176851237	5.26623013507027	14.42718660787093
C	0.99009002098192	5.22266330721865	18.75563853460173
H	1.17436479331854	11.51800844126838	14.49883751154137
H	0.95158719797731	11.99067940692179	17.15290589256596
H	2.22165513056297	6.97866702760970	11.60053434021863
H	2.73921235688179	4.44748425475892	12.37285987537398
H	2.65704790093810	7.33405733316155	19.26051970766207
H	3.16788601116550	3.29356163986436	14.76212548153917
H	1.66531589047056	3.65251193499511	15.64849193816440
H	3.20187512788701	4.33563470231848	16.20790879073753
H	0.73207826436430	8.72546254024122	19.08775745891481
H	0.89735951973852	10.49237746978369	19.29252987928060
H	2.34251120265763	9.46604500001490	19.21708090157946
H	1.75504170135141	8.95934757101370	11.76077522067643
H	2.38254257390276	10.35272396876268	12.66583891641642
H	0.63873907589797	10.04721163625309	12.60905457177791

S = 0 (closed shell)

Fe	1.39350195666535	7.03245069303032	16.86302417409263
O	1.32736026267601	5.18513031067169	18.01017247267268
O	3.30696674825223	4.70904791268972	19.12613172134284
O	2.71533594439680	7.13604813734352	18.13873130990279
N	1.23319236153598	8.87180619428664	16.41196155483145
N	1.83695896959678	6.49947973554756	15.07724186805775
C	2.26653278900853	9.56902566863425	12.81311339642225
C	1.32891915134710	10.90793525326511	15.38185172718101
C	0.46668540636444	9.47729576104846	18.69942408164093
C	0.88692674801267	11.09457821060440	16.68121903772183
C	2.41788370369733	6.63752250387906	12.86300134721989
C	1.95411276758431	8.79721430797301	14.07383654440384
C	2.38313290581599	5.29919868550425	13.22314160801189
C	1.89980048757298	3.98681206531053	15.38572043514496
C	1.54458190821255	9.50299184699496	15.21081039741932
C	0.84311993628440	9.80829273632946	17.29135793553327
C	2.07312495019908	7.39307614944420	14.01749363984436
C	2.02611771989827	5.24696329139984	14.59263415452435
C	2.39343972847928	5.14305265524421	18.55237427027745
H	1.46948385422538	11.67796316684367	14.62686982176929
H	0.61498438531501	12.03231403347187	17.16307612026772
H	2.64806628074303	7.04342285056158	11.88057533692643
H	2.58250196236020	4.43363831314690	12.59353940514592
H	3.54660824384136	7.58929352293290	17.95487482799482
H	1.96345476057246	3.12229558617925	14.71003689856003
H	0.94997490028516	3.92758685432410	15.93474569375128
H	2.71191519160319	3.88551368904787	16.12465509315435
H	-0.17082636853163	8.57867367883002	18.74730061287549
H	-0.08237402229776	10.30908444411094	19.16301228226297
H	1.36068463812151	9.26394768421794	19.30803225189733
H	3.27400464172714	9.32202290536154	12.44571486284592
H	2.21984728727819	10.65222320201956	12.96706784789821
H	1.55652979915623	9.30828794974864	12.01216726840043

Table S24. Coordinates of the optimized molecular structure for the (^{Me}L)Fe(κ^2-O,O-HCO_3).

S = 2

Fe	3.439405	-0.332880	16.678165
O	2.432166	-1.260403	18.298294
O	4.625750	-1.422217	18.066828
C	3.551764	-1.680964	18.696648
N	3.627150	-0.828662	14.762189
N	3.165561	1.608993	16.388159
O	3.598082	-2.420492	19.818277
C	3.844207	-2.041790	14.228628
C	3.573977	0.110063	13.722035
C	3.159807	2.181566	15.109331

C	2.957786	2.584938	17.288967
C	3.946273	-1.942268	12.817322
C	3.936679	-3.266375	15.079945
C	3.776831	-0.602578	12.498618
C	3.351448	1.494119	13.888563
C	2.932927	3.581406	15.269264
C	2.805747	3.831145	16.629593
C	2.929159	2.304895	18.756629
H	4.123271	-2.769501	12.132129
H	3.797354	-0.176338	11.498973
C	3.309566	2.354433	12.642818
H	2.869403	4.315335	14.469072
H	2.624077	4.788872	17.114465
H	4.528704	-2.648296	19.972096
H	3.453328	1.777994	11.724633
H	4.091833	3.127930	12.683086
H	2.342499	2.874565	12.566698
H	4.168667	-4.151980	14.472666
H	2.984834	-3.452349	15.606452
H	4.716856	-3.156646	15.851079
H	3.936805	2.048995	19.128432
H	2.270767	1.452393	18.989683
H	2.577159	3.182127	19.317126

$S = 1$

Fe	3.632415	-0.363290	16.679657
O	4.336223	-2.096516	17.427356
O	4.122183	-0.280208	18.633096
C	4.478652	-1.496953	18.527964
N	3.484793	-0.804241	14.805983
N	3.123022	1.472431	16.363183
O	5.008172	-2.148266	19.565992
C	3.438725	-2.036447	14.252833
C	3.647023	0.121299	13.767530
C	3.332091	2.149715	15.156095
C	2.734097	2.382490	17.282084
C	3.583339	-1.940874	12.844406
C	3.247092	-3.301503	15.022007
C	3.730703	-0.595938	12.540283
C	3.614919	1.515612	13.934430
C	3.085634	3.535271	15.374803
C	2.696130	3.675218	16.698762
C	2.378369	2.053896	18.694685
H	3.567720	-2.781928	12.153095
H	3.852867	-0.159689	11.551398
C	3.792586	2.378627	12.706948
H	3.158006	4.324201	14.629625
H	2.401370	4.588790	17.212544
H	5.041657	-1.531133	20.315044
H	4.498333	1.925030	11.999203
H	4.170576	3.375394	12.964499
H	2.831002	2.508156	12.182120
H	2.906002	-4.099795	14.347180
H	2.511029	-3.181048	15.829327
H	4.183610	-3.628171	15.499469
H	3.276093	1.959977	19.325134
H	1.841196	1.097640	18.765762
H	1.744210	2.848281	19.114361

$S = 0$ (open shell)

Fe	3.403409	-0.390943	16.674446
O	3.119052	-2.264343	17.357636
O	2.866222	-0.496210	18.625848
C	2.820754	-1.757611	18.473656
N	3.770481	-0.762556	14.815792
N	3.369524	1.519414	16.375414
O	2.457235	-2.557545	19.479513
C	4.177468	-1.933862	14.277503
C	3.426717	0.091960	13.760706
C	3.079502	2.121333	15.143709
C	3.409553	2.497769	17.307921

C	4.093911	-1.871791	12.862214
C	4.660733	-3.105962	15.066042
C	3.610359	-0.613337	12.537620
C	3.069234	1.441708	13.913757
C	2.911951	3.518828	15.359276
C	3.134505	3.752866	16.708223
C	3.714623	2.276615	18.752799
H	4.373293	-2.673486	12.180572
H	3.432226	-0.220781	11.538958
C	2.748153	2.226288	12.662776
H	2.684280	4.263250	14.599897
H	3.116698	4.708934	17.228898
H	2.259991	-1.995137	20.246173
H	2.182957	1.614875	11.946874
H	3.675432	2.551002	12.161588
H	2.152527	3.120187	12.881594
H	5.256985	-3.767504	14.421039
H	3.822211	-3.687971	15.478237
H	5.276137	-2.796654	15.922426
H	4.517260	1.539113	18.893511
H	2.837289	1.891867	19.295092
H	4.019169	3.225716	19.217151

S = 0 (closed shell)

Fe	3.013098	-0.292248	16.747188
O	3.121330	-2.029827	17.897028
O	4.637238	-0.445231	17.832425
C	4.229548	-1.574226	18.270901
N	3.384802	-0.787015	14.935801
N	2.907827	1.561767	16.331454
O	4.988505	-2.275986	19.121682
C	3.543012	-2.039273	14.453711
C	3.554607	0.106104	13.860557
C	3.130423	2.199714	15.120426
C	2.638764	2.495756	17.258415
C	3.816952	-1.996337	13.061233
C	3.457278	-3.288907	15.267114
C	3.827535	-0.664005	12.689986
C	3.446665	1.505537	13.940749
C	2.979636	3.604256	15.324888
C	2.669250	3.787707	16.666925
C	2.414349	2.127169	18.690380
H	3.978128	-2.866441	12.427113
H	3.998003	-0.272553	11.690416
C	3.690456	2.340057	12.705824
H	3.082115	4.377781	14.566489
H	2.479791	4.727728	17.182831
H	5.800646	-1.767370	19.278844
H	3.830832	1.737148	11.803348
H	4.589651	2.962442	12.842055
H	2.847702	3.025477	12.529724
H	3.475272	-4.162774	14.600662
H	2.546947	-3.327839	15.880347
H	4.304789	-3.378522	15.965182
H	1.877598	1.166063	18.782865
H	1.815817	2.888965	19.210624
H	3.371274	2.020913	19.229672

Table S25. Coordinates of the optimized molecular structure for the (^{Me}L)Fe(κ^2-O,OH-HCO_3).

S = 2

Fe	3.643047	-0.213111	16.695736
O	2.277238	-2.514948	17.931511
O	3.831398	-1.003063	18.393174
C	3.154967	-2.001452	18.878783
N	3.807408	-0.723316	14.782488
N	3.319587	1.712587	16.340147
O	3.222083	-2.473811	19.988814
C	4.074333	-1.942355	14.278053
C	3.634231	0.167736	13.712588

C	3.216170	2.258062	15.054566
C	3.133197	2.702113	17.235205
C	4.080509	-1.891814	12.861767
C	4.333988	-3.121888	15.157102
C	3.805019	-0.578914	12.506507
C	3.357195	1.544651	13.843275
C	2.953828	3.651691	15.199487
C	2.904051	3.927032	16.560822
C	3.159805	2.453224	18.708227
H	4.269486	-2.733137	12.197384
H	3.736254	-0.191084	11.493346
C	3.197680	2.359444	12.577953
H	2.822046	4.366067	14.389902
H	2.726466	4.889775	17.037019
H	1.800570	-3.240228	18.363793
H	4.438421	-4.038813	14.561075
H	3.519168	-3.265032	15.885097
H	5.264041	-2.985957	15.735740
H	4.036848	1.855719	19.003601
H	2.268827	1.889443	19.033413
H	3.180732	3.400391	19.264971
H	3.303377	1.758054	11.670510
H	3.948897	3.163336	12.540674
H	2.207245	2.839008	12.552250

$S = 1$

Fe	3.244147	-0.332363	16.704602
O	2.975228	-2.141437	17.517695
O	2.497684	-0.277678	18.516461
C	2.322150	-1.528918	18.686867
N	3.754524	-0.740866	14.884130
N	3.310877	1.564851	16.380032
O	1.791219	-2.220360	19.504764
C	4.197774	-1.919181	14.385072
C	3.483225	0.097588	13.798507
C	3.082499	2.151640	15.126673
C	3.344766	2.552891	17.297749
C	4.209286	-1.875562	12.970861
C	4.624077	-3.083206	15.220951
C	3.746513	-0.618214	12.600515
C	3.124561	1.456677	13.911837
C	2.939603	3.558988	15.317536
C	3.122212	3.807426	16.666172
C	3.602641	2.353535	18.753333
H	4.536041	-2.682644	12.317060
H	3.637406	-0.235197	11.588276
C	2.869996	2.214732	12.630567
H	2.759367	4.296150	14.538788
H	3.113747	4.770913	17.172844
H	2.704702	-3.057941	17.372149
H	5.344097	-3.700120	14.663700
H	3.772287	-3.741442	15.469026
H	5.093157	-2.767739	16.162740
H	4.349784	1.567444	18.929310
H	2.689480	2.038503	19.280722
H	3.958086	3.292871	19.200687
H	2.254886	1.619862	11.941013
H	3.820180	2.434693	12.116105
H	2.351906	3.163515	12.808236

$S = 0$ (open shell)

Fe	3.254889	-0.329686	16.703855
O	3.033520	-2.143325	17.551332
O	2.486403	-0.277606	18.510060
C	2.363705	-1.531378	18.707510
N	3.746952	-0.743211	14.878304
N	3.309054	1.569846	16.378908
O	1.865872	-2.224818	19.545331
C	4.175800	-1.925932	14.373393
C	3.490429	0.102958	13.793980
C	3.088458	2.157552	15.123927

C	3.328987	2.557892	17.297346
C	4.192985	-1.876040	12.960382
C	4.581827	-3.102183	15.202163
C	3.748959	-0.610353	12.594175
C	3.138528	1.463922	13.908994
C	2.935865	3.564130	15.315236
C	3.103918	3.811914	16.665683
C	3.577554	2.358796	18.754540
H	4.510282	-2.684684	12.303861
H	3.648387	-0.221565	11.583274
C	2.894330	2.223997	12.626612
H	2.757388	4.301642	14.536483
H	3.084064	4.774673	17.173417
H	2.822116	-3.080671	17.447689
H	5.265450	-3.744488	14.628253
H	3.713244	-3.728762	15.472727
H	5.086775	-2.802373	16.130653
H	4.330140	1.578853	18.935027
H	2.663514	2.034473	19.274687
H	3.921187	3.300464	19.206211
H	2.265320	1.638588	11.941301
H	3.846550	2.423459	12.107704
H	2.395659	3.183326	12.801965

S = 0 (closed shell)

Fe	3.877843	-0.127119	16.826332
O	2.333716	-0.903764	17.722239
O	4.368617	-1.660452	18.033947
C	3.179695	-2.049169	18.228319
N	3.891992	-0.698881	14.995818
N	3.367506	1.647336	16.353811
O	2.630182	-3.021312	18.653352
C	4.182022	-1.941145	14.548268
C	3.629895	0.106847	13.869982
C	3.161658	2.205107	15.100063
C	3.168221	2.603887	17.279096
C	4.122959	-1.975533	13.129725
C	4.499742	-3.112060	15.418697
C	3.780238	-0.705027	12.705340
C	3.286983	1.468823	13.910039
C	2.823672	3.579517	15.275240
C	2.829200	3.827640	16.643007
C	3.269536	2.306177	18.741402
H	4.320151	-2.852867	12.516233
H	3.654927	-0.378211	11.676250
C	3.027741	2.210655	12.620245
H	2.611811	4.293936	14.482320
H	2.620684	4.769396	17.148162
H	1.502987	-1.222840	17.339617
H	4.831938	-3.954252	14.795869
H	3.618335	-3.451224	15.988145
H	5.279946	-2.884103	16.157311
H	4.095509	1.606478	18.960765
H	2.342946	1.843240	19.122437
H	3.451118	3.222376	19.321249
H	3.167642	1.586665	11.732126
H	3.700816	3.078078	12.536067
H	1.997882	2.601211	12.603506

Table S26. Coordinates of the optimized molecular structure during the reaction between (^{Me}L)Fe(H) and CO_2.

(^{Me}L)Fe(H)

Fe	4.76838862952888	7.24020999482804	16.67779001160359
N	4.33159868868855	5.30109243642709	16.41295069983004
N	4.99535798263368	7.62361097973536	14.72289940279456
H	4.90444640866079	8.18046494478317	18.00290602434431
C	4.28064914956159	4.68395059503697	15.15572540426282
C	4.04723821135299	4.37599429998517	17.34538277844351

C	4.84967863230559	6.66849117770691	13.70728647315931
C	5.31133126203402	8.79927693751223	14.15104577637490
C	4.51874867487295	5.31198810496122	13.91417753314421
C	3.94670437922969	3.31136818559100	15.36360289479639
C	3.80103213516440	3.11988908679177	16.72929471351149
C	4.00972461416959	4.69328611671126	18.80543152878336
C	5.09657129078753	7.32595408876524	12.46267909116930
C	5.53519757502343	10.04030213817995	14.95364675008951
C	5.38455407880753	8.65423082073235	12.74265154013484
C	4.40255828442154	4.41977324803466	12.69596295643840
H	3.82912213999726	2.55665545480029	14.58902585832059
H	3.54844829199630	2.19561090120597	17.24659137781864
H	5.06661558351252	6.87245973062347	11.47517120454257
H	5.62337729796096	9.44521788457968	12.03369659306175
H	4.27287617320881	5.74409352044107	18.99814626211252
H	4.71256897908923	4.05361382570774	19.36490524051634
H	3.00507611017513	4.50887246374774	19.22207767064291
H	5.80022092511583	10.88362688563035	14.30078234147818
H	6.34578447581608	9.90321892162072	15.68843582729553
H	4.63308350852317	10.31640729967638	15.52477424472057
H	4.58286775860490	4.95565806056199	11.75969126991649
H	3.39852190222230	3.97181397785227	12.64049585331749
H	5.12467685653470	3.59075791776986	12.75513267737567

Transition state

Fe	2.06415089077721	6.97843688842272	16.80779779103222
O	1.60198316744006	4.86469374864571	19.69750451885496
O	0.41521188451854	5.77727500198883	17.92106148269246
H	2.81210009837803	6.26992792633330	18.12943372006828
N	1.60903119720557	8.88295222451234	16.49835556270498
N	2.19589728564650	6.45480906953423	14.89260191910817
C	1.58491760819213	9.57362202718039	12.74007959650921
C	1.23583696711532	10.82090555665944	15.35745873239650
C	1.34423320347282	9.59354056206126	18.86026654792299
C	1.13630349127023	11.08616967583741	16.71760252932931
C	2.22174004958770	6.64845185196152	12.61975977836718
C	1.72361401657756	8.73753448088801	13.99513164327551
C	2.49971027719636	5.33094599340566	12.95001278643105
C	2.66717664136765	4.04093672288022	15.22129766694512
C	1.53394971171980	9.43365114844374	15.21180662797018
C	1.37073473493666	9.86074365737500	17.39008914496560
C	2.02738682633010	7.36856010329267	13.84065439376829
C	2.47142652590935	5.25082638961433	14.36682428982279
C	1.19930518146956	5.41369905366697	18.74990262658999
H	1.11410606273265	11.53826802840755	14.54908779606246
H	0.91928804073865	12.04108205061490	17.19340221710364
H	2.16791971960080	7.05688568265994	11.61391315955134
H	2.70254737958657	4.50560429396075	12.26964755426838
H	3.20414908278071	3.25387474196002	14.67304031144180
H	1.69583036249652	3.62330558199900	15.53983593004117
H	3.23549978854891	4.28189803634090	16.13399969611063
H	0.43923099137572	9.02781273715199	19.14329996960176
H	1.34442056169747	10.53253138390822	19.43113094608366
H	2.21426889051663	8.99372179830239	19.17409462258371
H	1.66761934037545	8.98183452141894	11.82429570970550
H	2.36224580941810	10.35339427875094	12.71117536223526
H	0.61139421101978	10.08589478181710	12.72341536644740

(^{Me}L)Fe(O_2CH)

Fe	4.67250160307968	7.12976408617567	16.72940396815807
O	3.66988578128968	8.07318869794670	18.36635622807785
N	4.35939671070176	5.18908262541891	16.45552362094940
O	5.84542991953884	8.28347552154050	18.08064884455900
N	4.88714843567973	7.59106302351678	14.80359979374968
C	4.78937624912017	8.53278723109250	18.73018304354470
C	4.34411686610317	4.59977998624485	15.18471232293780
C	4.12786871451470	4.22869870623949	17.36825235755467
C	4.81982233842753	6.64094009566646	13.77446745683666
C	5.14182962176418	8.79065465204062	14.25632992830963
C	4.56068659535346	5.26593966155424	13.95592739115931
C	4.08548017426799	3.20788180942123	15.36117834533754

C	3.94943443962012	2.97843312541803	16.72450393844679
C	4.09877436764301	4.52627795883242	18.83248948627171
C	5.05366269879129	7.33173397923721	12.54379299765876
C	5.25681902882116	10.02672259630446	15.08778341305942
C	5.25592018789306	8.66998150284650	12.84720601455042
C	4.50865870550658	4.39019287335169	12.72154663129259
H	4.00875506526462	2.46521421220673	14.57028222549852
H	3.74600366670956	2.03102703697891	17.22089780546366
H	5.07389855746580	6.89169231265093	11.55012545469816
H	5.46296882361320	9.48323565331265	12.15378099580606
H	4.84769326968863	9.16828189358468	19.63762843060999
H	5.09897951715201	4.82073691146982	19.19521868901840
H	3.77787522933826	3.64546874999722	19.40575336680917
H	3.41379820393881	5.35958339842718	19.05861453967197
H	4.65120212065527	4.95423144906167	11.79547302355330
H	3.53843037621948	3.87483945745829	12.65594828208441
H	5.28667514394931	3.61256638726598	12.76825431084071
H	5.54311899863304	10.88974706979644	14.47126441760456
H	6.00466090376193	9.90247627319104	15.88769854939906
H	4.29587768549368	10.26151106174981	15.57738412648684

Table S27. Coordinates of the optimized molecular structure during the reaction between (^{Me}L)Fe(CH_3) and CO_2.

(^{Me}L)Fe(CH_3)

Fe	4.55548503801685	7.18574769982678	16.81029167060345
N	4.19950449506658	5.22231731243614	16.52938678968171
N	4.77626555112526	7.57771379172848	14.84993498188938
C	4.69688916457542	8.44435013833926	18.39112092798369
C	3.94433693599411	4.27854248127641	17.45111589716425
C	4.19949556332455	4.60858639843255	15.26900974008247
C	5.03676607028403	8.76955710585000	14.28546767132230
C	4.69264692480784	6.62097609242790	13.83062371202356
H	3.90775691629294	9.21755777617830	18.33679505908889
H	5.66870102523197	8.97204964667267	18.37648602044578
H	4.60733896380606	7.95749822915643	19.37792251561149
C	3.87026156683120	4.57836338019254	18.91353845757572
C	3.76883912482443	3.01475804189074	16.82596412986036
C	4.42605636001502	5.24982328510395	14.03191733725592
C	3.92844022147082	3.22005772650043	15.46443457960461
C	5.18492333030885	10.01841219113783	15.09405295510120
C	5.13495986927695	8.63391875935545	12.87704475747507
C	4.92003860142730	7.29356999618896	12.58984125613095
H	3.55117706101579	2.07704000800986	17.33502456919353
C	4.37313433459599	4.35498560979260	12.81067344560925
H	3.85989023678887	2.46475117573724	14.68460231318347
H	5.34057426182297	9.43869964274664	12.17312941328601
H	4.92578177844679	6.84248558505441	11.60080119692781
H	4.53696544168996	4.90235000870667	11.87796876040758
H	3.39535012691575	3.85416483481546	12.74094541511340
H	5.13793500224389	3.56572482621221	12.87817826066589
H	5.40373745270050	10.87842747451046	14.44594445492458
H	5.99904741889848	9.92555146792769	15.83152914851776
H	4.26540563813165	10.23965976358491	15.66114744498488
H	4.03042923090601	5.64682713716786	19.11528916556435
H	4.63051160414753	4.00604985035766	19.47155869643712
H	2.88722468901554	4.29328256268143	19.32447925628331

Transition state

Fe	1.72951707342970	6.92368819707578	16.59922006509199
O	2.02984915355942	4.67745153023404	20.60074727122062
O	2.14111966364563	6.94225372436008	20.32155041916666
C	1.89361411824745	5.58834431665131	18.35340187527441
N	1.46650118100959	8.85215225351182	16.43704047100395
N	1.96133069930597	6.45832939164976	14.72759319117929
C	2.09293020805481	9.72812368349209	12.74570095407139
C	1.49280206094659	10.90138416460901	15.43280181396010
C	0.87221423480547	9.44578984141320	18.78439347951646
C	1.20481102236637	11.07459718677361	16.77507163998920
C	2.29906913368063	6.82530845247608	12.50885607508266

C	1.92881750117022	8.86148278421152	13.97564172248343
C	2.34238514655857	5.45071131761734	12.74040005860970
C	2.10306716309015	3.97414283483968	14.88005239301643
C	1.65623887628354	9.49975886619412	15.19759491457814
C	1.19017363952612	9.78527437604138	17.37066384349589
C	2.05766563816411	7.46479434099263	13.75252937076660
C	2.13303743351326	5.26003663827722	14.12054685684496
C	2.04485974802373	5.74783352483317	20.01069760160492
H	1.57531954920296	11.69037060038674	14.68980890711905
H	1.01728169197611	12.01064067813280	17.29766264458707
H	2.42278581986924	7.32033221087256	11.54861143783041
H	2.50567605471854	4.66150128363047	12.00884156471010
H	2.16965342098667	3.11742735904683	14.19591092375789
H	1.17367866438069	3.87034359411211	15.46438163198522
H	2.94662048389649	3.90400135799992	15.58806678807783
H	1.48076126051405	8.61740305949016	19.19209772740137
H	-0.18601630020752	9.13857094618043	18.87086170798666
H	1.00751275025458	10.32360315357432	19.43155215729789
H	3.07542839452679	9.55369944986670	12.28158331434884
H	2.01113091766826	10.79631255717141	12.96413987109359
H	1.32876014528727	9.47646771579142	11.99423134924570
H	1.90047377258026	4.55283383724385	17.99531754452885
H	0.89468566759855	6.06329315860367	18.19806684549961
H	2.81085401136500	6.14219161263960	18.03415156756620

$(^{Me}L)Fe(O_2CCH_3)$

Fe	4.56441671555504	7.23001350194502	16.74051068268080
O	3.70157080839585	8.79548400230426	17.86973250062002
N	4.20921758228497	5.28975915924710	16.44760297657017
O	5.47887450787994	7.71729619927728	18.57747056982229
N	4.89549725638319	7.66262003861491	14.81791834662425
C	4.59789111970054	8.62003999689732	18.75540047476245
C	4.25188364010478	4.69139760754172	15.18232253166680
C	3.93069356854288	4.33705632992344	17.35417085244512
C	4.80108231145370	6.72098426657306	13.78379126320628
C	5.20214989404813	8.85328565501129	14.27795195266078
C	4.61701117184288	9.45213578759600	20.01005035739139
C	4.51047538690137	5.35144980952164	13.95915855737681
C	3.98291296153324	3.30044361153302	15.35561670084447
C	3.78884394247505	4.65118075239081	18.80836508240731
C	5.06831658812741	7.40753275265378	12.55729914507562
C	5.39566773646945	10.07452016807784	15.11707994545938
C	5.31675664233416	8.73595477406439	12.86819462002930
H	4.37934381314622	8.80415340175364	20.86899150493013
H	3.88632662455551	10.26846714395206	19.95530235228441
H	5.62875450331869	9.85082461052537	20.17616007734684
C	4.47564615050259	4.47530282703138	12.72424929386864
H	5.07698769551158	6.97165805198907	11.56162311186006
H	5.55549961743827	9.54515459574818	12.18013116241707
H	4.62928978772808	5.03965340450219	11.80006446803380
H	3.50901528588556	3.95551361415516	12.64629464146530
H	5.25683363497223	3.70112484581422	12.78081236956582
H	3.71416672169782	3.73049045116161	19.40376433151764
H	2.88028302573390	5.25008283197894	18.99502774762215
H	4.64477289663757	5.24014554966822	19.17496646162747
H	5.54168433958521	10.96351967433595	14.48815931909312
H	6.28149720859991	9.96791367394038	15.76710668900840
H	4.53081545589498	10.24722275068789	15.77757215414112
C	3.78595962798645	3.08048014891304	16.71241048699844
H	3.94537398756466	2.55197008643556	14.56745005040759
H	3.56287778920792	2.13617792423392	17.20646721816799

Table S28. Coordinates of the optimized molecular structure during the reaction between $(^{Me}L)Fe(CH_2CH_3)$ and CO_2.

$(^{Me}L)Fe(CH_2CH_3)$

Fe	4.67569465921698	7.20249107057877	16.74141914801172
N	4.22652253100521	5.26048879817635	16.48353110712565
N	4.88657834883686	7.55999458269630	14.77014102655947
C	4.81586047262064	8.39651950511634	18.37915952164645

C	3.92069956002719	4.35207530975437	17.42604723399989
C	4.19315588884459	4.62258882698367	15.23857544743684
C	5.17939766549826	8.73238484687180	14.18455364468374
C	4.76040587990459	6.59029753233642	13.76654276838751
C	3.54369884479676	9.19001965149619	18.71575319127860
H	5.66199079354491	9.09429938811081	18.22427600312235
H	5.10699622297008	7.77147201832556	19.24537297868397
C	3.87731967140366	4.70222767681605	18.87923930311364
C	3.67590321817359	3.08897320588579	16.82878135885820
C	4.44302486692922	5.23384439995964	13.98886891676124
C	3.84804763676539	3.25562648156159	15.46078099798344
C	5.38617546227732	9.98491050585674	14.97440220832482
C	5.25607654371309	8.57351613057944	12.77618197180092
C	4.99631245319747	7.23688995963548	12.51239879556491
H	3.40568268512754	2.17662419845613	17.35811727466035
C	4.33849110657305	4.32565882608185	12.78170653946633
H	3.73559992324001	2.48927701042299	14.69704268215598
H	5.47741170627653	9.36099148857565	12.05760361104368
H	4.97320233539802	6.77143462903197	11.53023017151115
H	4.62075881856739	4.82522699394552	11.85043256546555
H	3.30758699040575	3.95442695767242	12.66822464208749
H	4.98809274614123	3.44611118631287	12.90243268747745
H	5.54691648905487	10.84471720564232	14.30940114402374
H	6.26074840822014	9.89830100029389	15.64063427168043
H	4.51674453238140	10.20264331849223	15.61624299328065
H	3.22673470597670	5.57263522306059	19.06420225639502
H	4.87951204423300	4.96596288906883	19.25759093030901
H	3.50109759730180	3.85794163799347	19.47373329940936
H	3.23132951627491	9.84254032823779	17.88088837030434
H	3.66352778784958	9.84603755893481	19.59953595048454
H	2.68852188725195	8.52528965703495	18.92999498690047

Transition state

Fe	1.77246625838001	7.13791130642707	16.69878519236395
O	3.61571220051976	4.15905565074813	18.30926226748797
O	2.32156278412591	4.09589367658096	20.20117425120098
C	1.79476239436322	5.91286647171375	18.47919227228730
N	1.48133442955882	9.04331745238528	16.33957607285050
N	2.12037705813985	6.52682526039787	14.85430698004529
C	1.94541806504095	9.62203082289542	12.58451797272152
C	1.35174398812532	10.95348853271549	15.10412176099508
C	1.04409168315491	9.89079932127093	18.64364752707806
C	1.12405913012322	11.27696851106938	16.43867535143680
C	2.46655800204360	6.68051230853211	12.60262216359691
C	1.88531635990361	8.81223474894808	13.86278521863551
C	2.63125744453490	5.35933306389374	12.98176959842155
C	2.43233118790571	4.08337683591959	15.24643960077312
C	1.58077601080409	9.55249040816113	15.03366967736600
C	1.21274730208453	10.07471494039835	17.17201793566963
C	2.14127976590489	7.43183693919293	13.77563076554684
C	2.40510925428670	5.29739435280421	14.38172289936479
C	2.76158933947405	4.43823413903510	19.13100345298084
H	1.36258707779696	11.64636370832503	14.26620225885569
H	0.92049773864010	12.26111096560605	16.85685858211448
H	2.57098475202359	7.07377288155981	11.59496032656513
H	2.88512533079359	4.51305322435262	12.34625359053030
H	2.82930632247016	3.22309370172021	14.69084652516024
H	1.41231067935390	3.81839508156643	15.57768287220930
H	3.04380870961310	4.21919810283231	16.15482163668237
H	1.88526126565469	9.32796032531999	19.07959259177612
H	0.12299480458106	9.33284837218780	18.87969419355055
H	0.98674657841707	10.86355384303497	19.15081749702538
H	2.12823321857078	9.00632963473900	11.69976936549165
H	2.74645497893540	10.37514656126037	12.64452679776808
H	1.00072751298979	10.16369310205405	12.42740557441694
H	1.38236987785678	5.28569568778456	17.66964790953454
C	0.83027611861963	6.45145761029206	19.51801682485923
H	2.76922725418705	6.41708887738253	18.31907559835958
H	-0.06354202223857	6.95065165217983	19.10687074709270
H	1.33901753093365	7.16356753991772	20.18389694544856

H	0.49603961232671	5.61207438479285	20.14883920173233

(^{Me}L)Fe($O_2CCH_2CH_3$)

Fe	4.62745008987091	7.19522633711003	16.72656069993176
O	3.77334181954975	8.78032154138223	17.85433453253050
N	4.23591948990950	5.26203283483938	16.42911734227479
O	5.53371520795156	7.67393533940013	18.55996475414786
N	4.94008479007029	7.63165921591543	14.80242626180101
C	4.66575038567558	8.59094157803772	18.73919255320252
C	4.24352534641094	4.67133298596377	15.15941381530339
C	3.94947047357878	4.31051544302016	17.33407312148688
C	4.81417845190391	6.69773265489724	13.76478517492650
C	5.26168530745347	8.81958732257569	14.26489339758615
C	4.71739184002009	9.42931102595660	19.99602578136816
C	4.49852770698379	5.33303905592235	13.93658812209250
C	3.94448094567965	3.28571717131128	15.32848874172747
C	3.83449668957742	4.61785693369149	18.79221180949322
C	5.07823875560709	7.38599385392455	12.53865969230969
C	5.48654933867758	10.03245382452805	15.10841994867951
C	5.35548136768747	8.70797416867047	12.85327828446860
H	4.69170659475955	8.72338978404151	20.84421858004085
C	3.62238581848943	10.48349947662794	20.11095011150572
H	5.72364848861388	9.88145824617376	20.03282652196201
C	4.42836059927436	4.46523023209491	12.69733724994101
H	5.06564342387950	6.95533054765598	11.54071265497656
H	5.60057428286758	9.51614617512443	12.16623045362879
H	4.58381187274389	5.03128597017732	11.77452084145697
H	3.44880961748365	3.96872289691283	12.62808812707629
H	5.19177803934481	3.67271997713880	12.74024180490613
H	3.79972241973161	3.69379729713987	19.38619017565680
H	2.91253855253441	5.18831023765850	19.00228953907354
H	4.68054788385988	5.23095875968874	19.14111265861006
H	5.65355657075448	10.92012187963821	14.48285956318118
H	6.36998776797771	9.90172172777753	15.75719240587969
H	4.62667155619085	10.22380750619968	15.77039193738511
C	3.76437626558359	3.06162955059641	16.68672900670787
H	3.87648805444042	2.54362035769951	14.53621693560432
H	3.52722111980256	2.11973798579629	17.17874012545154
H	3.66259867866974	11.19123706724741	19.26947527236965
H	3.72975646158133	11.05324958617302	21.04707948609749
H	2.62415792480881	10.02105345129051	20.09983251515731

Table S29. Coordinates of the optimized molecular structure during the reaction between (^{Me}L)Fe(NH_2) and CO_2.

(^{Me}L)Fe(NH_2)

Fe	1.73817669261361	7.05184173408890	16.63792732699978
N	1.69112579087319	6.03851885872227	18.20794989030114
N	2.04154593231676	6.57158907528424	14.70515781850040
N	1.52450446135035	9.00685439440435	16.23764358893182
C	2.24564843867314	5.34075023309210	14.21067329372804
C	2.05869042357785	7.47924309534053	13.63895860082347
C	1.29452223706007	9.99437115603887	17.11926105304041
C	1.62764973144036	9.57648146467303	14.96439087130392
C	2.30672719715840	4.13598901277230	15.09360417584025
C	2.40456702775911	5.39667145844195	12.80061472970983
C	1.87026579043206	8.87101011945922	13.76446369435763
C	2.28964117233501	6.73109788361288	12.44121536533972
C	1.24066226300852	11.24553711956222	16.45198724707393
C	1.10703713699156	9.72054008488125	18.57701187660293
C	1.44786226900396	10.98555759533276	15.10349308230261
H	2.58031267182298	4.54590278359927	12.14445034085201
C	1.93111443799762	9.71148879162763	12.50699057642605
H	2.35710186843499	7.13058722453115	11.43253857720249
H	1.06997473094592	12.21218411762541	16.92300528465712
H	1.47513939201983	11.71816911210575	14.29989535241600
H	2.06026623185360	5.10429917830617	18.31859332866751
H	1.31099806109296	6.34085613089055	19.09417590900739
H	1.87175503712446	9.02231977856351	18.95509524782694
H	0.12152357970557	9.26167872488432	18.77135702546010

H	1.16496241910021	10.64875964678997	19.16287084968690
H	2.24103785645011	3.21112548581405	14.50345763111077
H	1.48782983558679	4.13949649663256	15.83129718824258
H	3.25457779650814	4.10721199973516	15.65952284574523
H	2.09433731199464	9.11413478838433	11.60522498997606
H	2.74593296147955	10.44898295843531	12.57619398204509
H	0.99540924328862	10.27579949636770	12.37436825582126

Transition state

Fe	1.36752660751418	6.87478696545327	16.65745757457085
O	3.17223516311803	4.70818935418610	18.46554677440733
O	2.66148494649110	6.51119027439901	19.79874558075010
N	0.94086150680277	5.73301319400641	18.20098404976803
N	1.32114923622249	8.83801891579630	16.42297051239592
N	1.96807969468240	6.47851877303023	14.82058938565224
C	2.12420624116978	9.70202599942815	12.77339296584618
C	1.41124893099678	10.88973743634762	15.43608550303145
C	0.76976850581466	9.40292795569872	18.77819149474969
C	1.11474447927981	11.06261271603773	16.78147756972251
C	2.53453053457387	6.79317840740251	12.63671430171309
C	1.90005719758917	8.84137366861782	13.99747836863753
C	2.65853848856073	5.44064620250880	12.93224306129620
C	2.24363769195902	4.02490524760214	15.09714894082830
C	1.54997101214185	9.48783995995576	15.20154626635923
C	1.06549585935525	9.76998580504489	17.36031440761414
C	2.10510485202789	7.45425576866278	13.82434185495832
C	2.29751261364569	5.28309201644039	14.29302173914289
C	2.58765138759102	5.62095148459964	18.99156650663855
H	1.53319856379991	11.68040838474971	14.69992347135546
H	0.95371524917848	12.00086997427148	17.30931491998242
H	2.74266392474550	7.26721854839292	11.68009897820175
H	2.97534059095987	4.64199728754137	12.26383583015593
H	2.75288995034522	3.20465194059481	14.57260726393976
H	1.19838410164120	3.70920408748850	15.26644946874420
H	2.71793073419350	4.15121380546459	16.08489020550073
H	1.49659624950663	8.67233235728587	19.17178286004358
H	-0.23552896012978	8.95356824396717	18.86725335680846
H	0.79113134438293	10.29159894713759	19.42386165943654
H	1.58689005255624	9.29233107438022	11.90620336590438
H	3.19478425144977	9.72641268856928	12.51259887805902
H	1.78774590239463	10.73334046149263	12.91707145848789
H	0.66565306704501	4.77586586457240	18.00352699242874
H	0.31399002839383	6.10690618887041	18.90651443286348

$(^{Me}L)Fe(\kappa^2\text{-}O,N\text{-}O_2CNH_2)$

Fe	2.00737289709001	6.97277501443221	16.87020223832822
O	2.76851806158516	5.89493350271768	18.34811229047263
O	1.64228491126719	4.14643535774296	19.26864820208532
N	0.51734188774654	5.87427391058832	18.10995530085266
N	1.62904767236708	8.89816733816273	16.48368999386495
N	2.25307684409435	6.50957232847261	14.91218318272837
C	1.84188567752895	9.64359685922751	12.74493082671094
C	1.33014359990695	10.85970200406931	15.36377829931844
C	1.25912956967334	9.56581394750359	18.84351134904949
C	1.16122308522321	11.10183991026114	16.72287437206841
C	2.44823458979287	6.70186600323655	12.64307555587808
C	1.88617382060007	8.79226061533902	13.99538185027339
C	2.67256417169804	5.38173346867487	12.99017448423166
C	2.66748546979426	4.09416287363774	15.27513718226667
C	1.62484970194831	9.47308559392162	15.21042795925392
C	1.35456881523466	9.86289005557820	17.38090109499552
C	2.18060548305083	7.42400734050418	13.85418336167498
C	2.53982471189495	5.30353092420783	14.40626695066200
C	1.73177530299953	5.19252524558698	18.67501132462606
H	1.25794455475650	11.59214526076763	14.56288400762075
H	0.93003957524429	12.05070908465724	17.20419292777211
H	2.47297383040160	7.11425915174746	11.63757794567095
H	2.90639081483235	4.55060415860219	12.32691853180855
H	2.99435565216609	3.22163310319302	14.69321501393231
H	1.70213529379349	3.84003542436646	15.74665926206080
H	3.38812960277870	4.25687413451630	16.09314030468060

H	2.02449383599262	8.83669818869410	19.15785713632454
H	0.26954786270944	9.14727939769365	19.10328491441495
H	1.39217348057133	10.47743933943271	19.44331872852116
H	2.00969953407894	9.06435141030436	11.83227330937285
H	2.60806904927093	10.43328588972522	12.79140252245969
H	0.86627390433757	10.14471566938405	12.65308325848350
H	-0.20546672997120	5.19088894228494	17.90593642269122
H	0.13632346554011	6.51107855076201	18.80555989483669

(^{Me}L)Fe(κ^2-O,O-O_2CNH_2)

Fe	9.92902556385725	7.20494831943776	16.79594621543314
O	10.86412505825152	7.72406632630564	18.60782865460254
O	9.04387051781433	8.76601546875603	17.90005659591101
N	10.22049823883250	7.63990973639462	14.86312780780967
N	9.61513989835293	5.25431664799334	16.50612232827544
C	9.96376133649578	8.61694690745332	18.77543933333358
C	10.50301093374344	8.83396476058826	14.31886165189895
C	10.10840273094932	6.70004515828885	13.82958444124192
C	9.62293078530197	4.66108450245489	15.23767721343662
C	9.38086955309769	4.29435647479016	17.41719298567851
N	9.99002180978698	9.40767928628119	19.87509277817022
C	10.58327283291115	8.72101754350207	12.90581243323203
C	10.70511279634485	10.05496386739215	15.15625693124105
C	10.33841385818513	7.39148144434818	12.59781373334268
C	9.83696157186169	5.32737222615716	14.00929971666826
C	9.37848111820954	3.26564186884470	15.41373722202773
C	9.23133730154681	3.03780661352139	16.77528529711056
C	9.27644439913956	4.59982496686183	18.87652499945165
H	10.69368499771097	9.26078473334620	20.57597441147058
H	9.25691982501231	10.07736859597830	20.02329298757741
H	10.79863806693539	9.53359685541748	12.21395279787608
H	10.32662245517368	6.95752942905286	11.60125961055982
C	9.77384059017202	4.45415050830191	12.77330080185033
H	9.32534143040970	2.51966689356847	14.62392373165862
H	9.03924305329926	2.08852398935901	17.27283620505299
H	10.06956905946473	5.29273260182831	19.19879323442625
H	9.34374527967901	3.68057056949442	19.47553807958404
H	8.31011647211288	5.08178440860752	19.10852918583557
H	9.89972890701369	5.02178482631135	11.84692215258265
H	8.80772568121335	3.92999792937725	12.71956871523436
H	10.55956753662744	3.68332515158618	12.80687810931420
H	10.85110957611256	10.94321844214373	14.52619700985224
H	11.59258011834859	9.94500953744988	15.80318980541374
H	9.84428664603148	10.22927340880526	15.82159282284493

Table S30. Coordinates of the optimized molecular structure during the reaction between (^{Me}L)Fe(F) and CO_2.

(^{Me}L)Fe(F)

Fe	4.54552271479470	7.26556088793212	16.64801613210098
N	4.26057960321005	5.31233928208509	16.39674555538117
N	4.98354160150383	7.60338771199007	14.72343570196937
F	4.42488889515862	8.48187776309094	17.96455426385965
C	4.22223070984661	4.68770345346537	15.14309971564988
C	4.00714672929980	4.38197527800487	17.33655911776640
C	4.83761977502070	6.65717531002864	13.69978225528163
C	5.37545002900480	8.76413956930759	14.17501068818882
C	4.47155775466757	5.31216207986035	13.89877484616526
C	3.91894970432196	3.31169063032656	15.35745263652318
C	3.78825110908760	3.12114900731499	16.72759279519973
C	4.02006166133431	4.70440694472358	18.79567462651217
C	5.15783778833856	7.31056825194046	12.46755024476314
C	5.65473296220372	9.98091730377202	14.99538346344884
C	5.49609093315406	8.62071276642671	12.76574473019047
C	4.35693341605228	4.42207380304075	12.67993931211092
H	3.81512983050036	2.55141060023807	14.58631164663696
H	3.56309378619606	2.19230022541428	17.24912131198876
H	5.14587316776356	6.86121945645881	11.47750798709781
H	5.79821358030141	9.40175026387689	12.07001077626554
H	3.77391156599353	5.76144911597561	18.98406349516191

H	5.01823234822546	4.51649150984284	19.22987783387400
H	3.30253420541205	4.07711791951126	19.34550219281754
H	5.21303069313337	10.87738187926256	14.53264365137397
H	6.74218569285760	10.15806239634358	15.06799796076638
H	5.26090466983475	9.88416625562038	16.01848751838251
H	4.47119394941467	4.97430292540633	11.74235797515817
H	3.37705332402394	3.92224715212162	12.65834319821871
H	5.12426779934326	3.63215025661636	12.70481836714352

Transition state

Fe	1.80181647082611	6.95430967979614	16.72884390574705
O	1.92717288165617	5.56721488154505	18.00317357053093
O	2.14116978979220	4.68029476116056	20.06273861747838
F	2.01546186669236	6.88027786292341	19.76013519362801
N	1.50169793050453	8.87565194054839	16.38553283856086
N	2.03049421953713	6.42633495925027	14.82483565010531
C	1.79736960918142	9.55897749425106	12.62822526516281
C	1.32868509571243	10.82565350103244	15.21312852839812
C	1.11937412500538	9.65780599964801	18.72557733207913
C	1.13209873605655	11.10491456756762	16.56003890102648
C	2.22847936782873	6.59085175729242	12.55857714683344
C	1.79089300267004	8.72105243509401	13.88913678726078
C	2.37864222072793	5.25938571043023	12.91630997378245
C	2.33199722984661	3.98063811079997	15.19355681310071
C	1.55945122362759	9.42634398948849	15.09347232755021
C	1.24577899753932	9.87849516672940	17.25462539100527
C	2.00637928199362	7.33571772173213	13.75782111603679
C	2.25004861405194	5.19423935042342	14.32733929113160
C	2.03482266510493	5.57766133084907	19.28595023638941
H	1.30677817755203	11.54178102422060	14.39497769466099
H	0.92712535435575	12.07258091244789	17.01448118273801
H	2.27064536269300	6.99097559042735	11.54887792059944
H	2.56021747606928	4.41202325583883	12.25762451399589
H	2.49534513094655	3.08118888583518	14.58485868272869
H	1.40995986795232	3.84292223001209	15.78073609604422
H	3.15230136252847	4.06180967630366	15.92467278853361
H	2.07481073059060	9.34039348164734	19.17317707004058
H	0.38797128095592	8.86744360312329	18.95831058042676
H	0.79715490329740	10.58153314993255	19.22557427920730
H	1.98348086502151	8.96631533193835	11.72810336198897
H	2.57380726553976	10.33700449738807	12.68643998459754
H	0.83179889414163	10.07199714031940	12.50112695862381

(^{Me}L)Fe(κ^2-O,O-O_2CF)

Fe	4.68854919395626	7.12991590732665	16.69674198437843
O	3.71494925234230	8.47210160105272	18.05119438987589
N	4.36371326650333	5.19957352213918	16.45160468798743
O	5.77981889360247	7.77089969729457	18.42768881858047
N	4.90757175774591	7.59928624745246	14.78713765994450
C	4.76257352923094	8.43276033186094	18.73639342546716
C	4.36775698890084	4.60272832635972	15.18369703807503
C	4.14141690678446	4.24316250112527	17.37026291231219
C	4.80910686809298	6.64608520423056	13.76161488219103
C	5.14233477347560	8.79953069750785	14.23076218974806
C	4.56579670038019	5.26816061592728	13.95147769602340
C	4.13269973188314	3.20805039227047	15.36974509697386
C	3.99294564344424	2.98546919183695	16.73424348215974
C	4.07441814389236	4.56197701973765	18.82851386668512
C	5.00103827435622	7.33723013095896	12.52496627408357
C	5.29823306870386	10.03006941932492	15.06360629593590
C	5.20721502315759	8.67764868718821	12.81997687964171
C	4.50956897905156	4.38787810071035	12.72048558650993
H	4.07527458736682	2.45810914470797	14.58412376050888
H	3.80525533572289	2.03775400598099	17.23599622339350
H	4.98793915747384	6.89712729102082	11.53123656486539
H	5.38588026392863	9.49064837828450	12.11847120400855
F	4.79474249161798	9.13532729818789	19.86364814225839
H	5.01393461261232	5.02148962462010	19.17873426912785
H	3.89097194896531	3.65635199424538	19.42256285865668
H	3.26291218819788	5.27923412135175	19.03949524226410
H	4.64196968825015	4.94887721035793	11.79115362077258

H	3.54332025920339	3.86410908463199	12.66441509273258
H	5.29476075202573	3.61736698182858	12.76446002005607
H	5.47613620254500	10.91272968878710	14.43445782372538
H	6.14703765472983	9.93068694357082	15.76153647603589
H	4.39690786185571	10.21487063811906	15.67182553501986

Table S31. Coordinates of the optimized molecular structure during the reaction between (^{Me}L)Fe(SH) and CO_2.

(^{Me}L)Fe(SH)

Fe	7.85462870983090	9.38470835649817	5.49286693562387
S	7.79719664700476	8.13864255807748	3.60962933879507
N	7.55910362850497	8.97877814525561	7.41642585381749
N	8.20924798565309	11.33074234882718	5.75143562235965
C	8.12148055190497	12.13031767092158	9.50411301382717
C	7.02424278820351	6.55915359790278	7.19526469196187
C	7.12011092838296	7.96780449852167	9.39505505188458
C	7.42098375357038	9.29581727794651	9.67057015217055
C	8.02969105294257	11.30006845284318	8.24118896226118
C	8.58304495714986	13.32469599390129	6.79664531505123
C	8.45663228890872	11.94695630492475	3.35472419887514
C	8.70789425530103	13.51683396702122	5.42906789170722
C	7.21947716248886	7.80892464078695	7.99049732725582
C	7.69596333184448	9.93879090210742	8.42805975108184
C	8.26542536517500	11.94713929031294	7.01088654137165
C	8.46811642593578	12.26050589252818	4.81509158570609
H	7.14242077556454	12.17598287794222	10.00647696896914
H	8.82950687667201	11.67679853528757	10.21365187495908
H	8.44972720247774	13.15607668956966	9.31357257980213
H	9.02061802430993	11.02731556910395	3.13193618446659
H	7.42557676599693	11.78150832589453	2.99648387566564
H	8.88570119003201	12.77544397615686	2.77418478939361
H	7.99496504798767	6.12063556525113	6.90356586158388
H	6.48130222836509	5.80405233740742	7.78108742186394
H	6.46656755465557	6.75399801238162	6.26561274097494
H	6.85559969218887	7.18621182984763	10.10530336236625
H	7.43563471597883	9.76512678314479	10.65165668973219
H	8.70455016711182	14.08576019951644	7.56323215721392
H	8.94272065345122	14.44349422476210	4.90821516805163
H	8.42830927240558	7.08044517535685	4.15784809120643

Transition state

Fe	1.88231526992780	6.76534629611075	16.67536604616935
O	1.03684073732898	5.12023116420574	20.14784524573720
O	0.49184351195686	6.00730482962870	18.09749230229398
S	3.24662726240290	5.49524920149120	18.21965664441244
N	1.61987987021930	8.72845184529617	16.52316658624400
N	1.99328822264609	6.38518982339586	14.73144064940464
C	1.66611913984706	9.69884735608507	12.82509353453132
C	1.38567555661093	10.76670337717573	15.52609110597353
C	1.43196496124470	9.29688729296129	18.93363613342661
C	1.30299360338383	10.93950907559399	16.90177124126041
C	2.08021679649671	6.75254102877184	12.48232987239894
C	1.73147129458468	8.76227876109886	14.01359468308066
C	2.23506765005272	5.39308889788899	12.71044968048316
C	2.28876765624524	3.92849901392799	14.88187087828589
C	1.58422465355541	9.37556434318495	15.27994636265231
C	1.45356783849224	9.65573676083578	17.48326932488490
C	1.92766749133970	7.38916652609109	13.75420790428252
C	2.17349803725071	5.20559851697312	14.11520314849272
C	1.11047735416338	5.53372990350797	19.04849559642916
H	1.31417845719805	11.54733617222156	14.77219597295699
H	1.15196775707264	11.87033438750991	17.44592150628083
H	2.07490472361014	7.24043067646215	11.51106087348029
H	2.37578896873427	4.60826328999419	11.96926488495721
H	2.34423589904672	3.06564815976392	14.20420581583671
H	1.42158601641408	3.78505221048784	15.54842816183972
H	3.19106664030907	3.92461900613809	15.51657297499522
H	2.34221481444293	8.74532731244022	19.22244381639095

H	0.57102584085158	8.65034004327035	19.17185079862450
H	1.36702145713137	10.19737134369439	19.55967656348788
H	1.78451715480946	9.18004783152292	11.86972181718967
H	2.45347865744948	10.46497002192880	12.89363414472294
H	0.70030822028838	10.22659751044172	12.80435625724811
H	3.70773848489211	6.49290801989708	19.00844947154135

(^{Me}L)Fe(κ^2-O,SH-O_2CSH)

Fe	1.80550589515773	6.74498953137320	16.65942093189658
O	1.25989409240156	5.28370099962022	20.22275916525150
O	0.59306647296314	5.95918433950109	18.14260576900213
S	3.26959787415961	5.52131026487432	18.21464618389065
N	1.58607015113005	8.71761005594863	16.51916817366379
N	1.95656309103513	6.37785144173791	14.72174140978636
C	1.72381988279778	9.70839137790152	12.82915487758042
C	1.39223630965200	10.76509973931973	15.53040844309731
C	1.34630642140628	9.26980031037950	18.92744962519922
C	1.27684684383810	10.92875430145933	16.90378991701073
C	2.10309488724634	6.75844133944926	12.47947978397808
C	1.75166197155628	8.76545930602313	14.01418159491846
C	2.22709929456588	5.39347335315938	12.70141681994759
C	2.20562707542894	3.91591082403135	14.86501179146108
C	1.58498260114841	9.37278627918612	15.27933640022504
C	1.40344050578775	9.63857326820003	17.48048448687733
C	1.93193992162444	7.38933467157668	13.75027207814324
C	2.12954348927070	5.19778511024226	14.10121949425437
C	1.33256105977045	5.58258506281100	19.07798096947253
H	1.34429578653830	11.55095530733240	14.78006854169585
H	1.11888910262231	11.85665171053091	17.45094936241651
H	2.12998692317938	7.25226774240472	11.51157196507542
H	2.37032325714568	4.61174301986917	11.95746704986196
H	2.23018678907258	3.05278006150244	14.18577287810912
H	1.33747501971272	3.79964942651162	15.53538397756698
H	3.11173280378612	3.87973091464132	15.49368548560029
H	2.26084799860562	8.73808314203807	19.23995708753016
H	0.49621952534564	8.59973956754186	19.13636483329035
H	1.24240559379676	10.16424293858053	19.55678033850289
H	1.87475128793040	9.19508937150132	11.87538732235245
H	2.50624321385708	10.47620093097841	12.92682584948894
H	0.75765365296099	10.23384727094993	12.77932306758466
H	3.78167120450532	6.49714701882066	18.99864432526387

(^{Me}L)Fe(κ^2-O,S-O_2CSH)

Fe	3.14997773400600	-0.40473582741849	16.67588538844344
S	1.91197495835306	-1.73825437369359	18.27977449790995
O	4.46430825168602	-1.20526817215328	18.20021241405760
C	3.55149918525065	-1.80351610642718	18.81788447911001
N	3.48862429161563	-0.89704989448553	14.78200199755203
N	2.98813534473052	1.54939467185528	16.40890595081532
O	3.84485351020928	-2.51171944291846	19.91781209642237
C	3.70793610577948	-2.10789739917051	14.24563834897057
C	3.51005540193103	0.05505712546656	13.75149553117830
C	3.08257603459580	2.12932444577173	15.13631265754251
C	2.75673006771950	2.52679745051345	17.30234751570195
C	3.87905498962708	-1.99575831648039	12.84133038619899
C	3.77168211595680	-3.33997822242300	15.08828922226899
C	3.75671338110859	-0.64941218099755	12.53088984403472
C	3.31898077362583	1.44324292769887	13.92166985171981
C	2.89343983285411	3.53502230147908	15.29292630814644
C	2.68949347317901	3.78124850385136	16.64484218329645
C	2.63082857748480	2.23990353446724	18.76344379080486
H	4.06929735188464	-2.81930841739215	12.15516177625449
H	3.83437638937874	-0.21436874423613	11.53785033666080
C	3.37282257053861	2.31559013437053	12.68396206759973
H	2.90472389750529	4.27602899039510	14.49678485670820
H	2.51060805778693	4.74223353067157	17.12442435513422
H	4.80468881780499	-2.42925866777407	20.06191964687373
H	3.54051980261061	1.74240014734764	11.76783157929667
H	4.18156746854091	3.05758253144287	12.77143491856885
H	2.43184244441039	2.87397689015319	12.56518974724345
H	2.00360378249242	1.35159840484662	18.94243522841083

H	2.19166162594243	3.09459045243592	19.29707342661765
H	3.61955993390614	2.03459159915384	19.20985611443902
H	3.71052494009500	-4.24562792064031	14.46879134961650
H	2.95397920413299	-3.35995961686390	15.82677816881153
H	4.71944968325658	-3.37880033884631	15.65328396358925

(^{Me}L)Fe(κ^2-O,O-O_2CSH)

Fe	3.47038629523830	-0.36403563375180	16.65605140963401
O	2.52391131678175	-1.78443444841188	17.93890956561310
O	4.53606733715580	-0.98910427360937	18.38626779928143
C	3.55303831689804	-1.73335582089646	18.67455839791889
N	3.67954865992504	-0.84675898593698	14.74250895093108
N	3.17741197336760	1.57417675116685	16.38625685698853
S	3.67747985211982	-2.70979106490561	20.16187970016395
C	3.90363984304538	-2.05426847527224	14.19946873671579
C	3.59277996805409	0.09643355482621	13.70796431897495
C	3.17980022121298	2.15771536501122	15.11216435245242
C	2.96974407200640	2.54240480511173	17.29528422330506
C	3.97154748700273	-1.94772137769769	12.78665193917983
C	4.05495462101051	-3.27694159419275	15.04510452904790
C	3.77940268691548	-0.60869087803047	12.47767924384530
C	3.36606887270320	1.47854386053605	13.88564780832469
C	2.96037064864253	3.55715851642603	15.28436696423052
C	2.83134110000889	3.79575569543042	16.64674207806105
C	2.88600237055031	2.24018742364878	18.75639515320158
H	4.14284875897132	-2.76976583178180	12.09382399323147
H	3.77219876258458	-0.17837722130003	11.47957400083276
C	3.31521075403880	2.34752116745936	12.64633470991538
H	2.90916476673118	4.29964584348312	14.49119278729819
H	2.65895626635132	4.75115320401630	17.13949525995522
H	2.45655581361932	-3.27695774902850	20.03212907714276
H	3.69335012415265	1.55939959478640	19.07100830482660
H	1.92915710937255	1.74718282998129	19.00259201631938
H	2.95241711207853	3.16038142880291	19.35349744346523
H	4.16382873646616	-4.17574609197565	14.42293484694745
H	3.18431357887285	-3.41267546813164	15.70760754083838
H	4.94429308760195	-3.20034576844030	15.69395496111779
H	3.44707092863080	1.77708444373740	11.72263077200826
H	4.10251889413597	3.11611784133598	12.68395519282643
H	2.35053966375283	2.87374835760307	12.58390706540306

Table S32. Coordinates of the optimized molecular structure of the reaction product from reaction between (^{Me}L)Fe(OH) and CS_2.

(^{Me}L)Fe(κ^2-O,S-S_2COH)

Fe	1.987948	6.960399	16.663824
S	3.391194	5.973555	18.389884
O	0.844690	5.671955	17.949377
S	1.461600	4.181400	19.985377
N	1.563840	8.886236	16.477352
N	2.103145	6.525497	14.739614
C	1.527926	9.726327	12.732280
C	1.175003	10.891308	15.464583
C	1.319527	9.441093	18.879175
C	1.079636	11.067806	16.837365
C	2.108131	6.870418	12.486939
C	1.663175	8.876272	13.978683
C	2.358079	5.524730	12.726014
C	2.547685	4.094725	14.916427
C	1.479878	9.514334	15.225244
C	1.326869	9.802379	17.429671
C	1.944661	7.508256	13.753217
C	2.346984	5.351337	14.133429
C	1.807085	5.333819	18.677074
H	1.040300	11.663238	14.711155
H	0.859722	11.989768	17.372992
H	2.045661	7.351658	11.513572
H	2.531927	4.741548	11.989930
H	2.861091	3.268597	14.263296

H	1.615296	3.794174	15.424645
H	3.309885	4.231760	15.701142
H	1.383929	10.338216	19.510787
H	2.160316	8.773089	19.126831
H	0.391453	8.905775	19.145761
H	0.648323	9.413084	12.147470
H	2.408210	9.607144	12.083912
H	1.421376	10.791483	12.956581
H	2.727999	4.127785	20.455107

(^{Me}L)Fe(κ^2-S,SH-S$_2$COH)

Fe	1.653987	6.765753	16.635764
O	1.772136	5.329693	20.437378
S	0.311035	5.553531	18.184878
S	3.290128	5.672060	18.178814
N	1.536913	8.735281	16.504125
N	1.892736	6.378327	14.710539
C	1.799575	9.717313	12.818747
C	1.461450	10.787341	15.513138
C	1.256632	9.292635	18.906230
C	1.312937	10.955767	16.883898
C	2.075545	6.757181	12.470033
C	1.770551	8.773079	14.002889
C	2.158914	5.389985	12.691978
C	2.093121	3.911580	14.854224
C	1.601585	9.389155	15.265099
C	1.364879	9.662436	17.462674
C	1.907591	7.392441	13.740119
C	2.042270	5.195311	14.091939
C	1.681378	5.465914	19.259159
H	1.468360	11.575215	14.763399
H	1.180281	11.890003	17.427000
H	2.126153	7.250344	11.502726
H	2.287711	4.605081	11.948665
H	1.984767	3.048877	14.182405
H	1.297711	3.868143	15.615525
H	3.054725	3.807201	15.386335
H	2.184033	8.814467	19.265379
H	0.440781	8.570802	19.073894
H	1.071368	10.179997	19.526853
H	1.951824	9.199907	11.867464
H	2.606310	10.457175	12.933131
H	0.853666	10.276532	12.750805
H	3.951486	6.530638	18.993504

(^{Me}L)Fe(κ^2-S,S-S$_2$COH)

Fe	1.668909	6.923226	16.731047
O	1.664627	4.631504	20.144984
S	0.218490	5.651115	18.224476
S	3.173622	5.997626	18.445809
N	1.515024	8.879957	16.492580
N	1.891677	6.447527	14.819130
C	1.785360	9.689832	12.767961
C	1.442082	10.885339	15.411637
C	1.236335	9.533468	18.865605
C	1.294200	11.115078	16.774034
C	2.073425	6.715467	12.562141
C	1.754373	8.802296	13.995262
C	2.169474	5.362101	12.852367
C	2.093441	3.994621	15.089178
C	1.581759	9.477026	15.226820
C	1.344476	9.848011	17.408989
C	1.897173	7.411325	13.799922
C	2.052674	5.237467	14.261214
C	1.692164	5.368673	19.027537
H	1.450580	11.638991	14.627379
H	1.163699	12.073045	17.274832
H	2.123634	7.160628	11.571795
H	2.307968	4.541990	12.149834
H	2.216317	3.102984	14.459379
H	1.168567	3.882661	15.679257

H	2.926615	4.030749	15.811090
H	2.152129	9.039309	19.231609
H	0.401338	8.839673	19.059589
H	1.074505	10.445784	19.456141
H	1.921172	9.126642	11.840394
H	2.604410	10.421583	12.841982
H	0.847947	10.260576	12.682327
H	2.572706	4.560005	20.488051

(^{Me}L)Fe(κ^2-OH,S-S_2COH)

Fe	1.432456	6.985383	16.723422
S	0.318047	5.690755	18.366156
S	2.396161	4.780705	20.444877
O	2.795581	6.281057	18.318457
N	1.315614	8.931216	16.397911
N	1.940386	6.438267	14.889310
C	1.998849	9.588036	12.694313
C	1.321843	10.884730	15.223714
C	0.767451	9.688783	18.696177
C	1.014449	11.169323	16.548591
C	2.393439	6.621337	12.661899
C	1.842062	8.753183	13.948482
C	2.493919	5.285318	13.021508
C	2.205409	4.003257	15.286821
C	1.512873	9.474910	15.120261
C	1.020204	9.934222	17.244098
C	2.044753	7.360547	13.835560
C	2.206957	5.212046	14.408982
C	1.875966	5.555900	19.101792
H	1.402654	11.603450	14.411373
H	0.806239	12.144112	16.986464
H	2.553098	7.027828	11.666593
H	2.743443	4.443200	12.378334
H	3.677215	6.208912	18.726345
H	2.246135	3.082696	14.688327
H	1.308487	3.971353	15.925835
H	3.077989	4.006030	15.962919
H	1.689235	9.365583	19.210047
H	0.024111	8.888984	18.845257
H	0.406093	10.600407	19.191832
H	2.204585	8.984484	11.805917
H	2.822534	10.309464	12.811850
H	1.084314	10.168714	12.501486

Table S33. Coordinates of the optimized molecular structure during the reaction between (^{Me}L)Fe(H) and C_2H_4.

Transition state

Fe	2.64396173438705	7.06535886353608	16.48984713124442
C	4.43553930907629	6.67954382695899	17.54887236011061
H	1.89868191967166	6.05682215603585	17.57148544127058
N	1.95984321928127	8.91829053234260	16.33117762410724
N	2.49514255559003	6.62294904242840	14.55818964565874
C	1.26628477419156	9.72503730410828	12.66283975086145
C	1.20793050150224	10.84387557484892	15.36645603555574
C	2.02680017726002	9.51109192511742	18.73466878681978
C	1.29133831803128	11.04328130546025	16.73874424267353
C	2.12431673994972	6.87350505349469	12.31997608244407
C	1.68637954708390	8.87239094547746	13.84149870673952
C	2.55653987455279	5.57574364861299	12.55061601512453
C	3.24457488409132	4.26892106574319	14.71597497842020
C	1.62705454514454	9.50478610084381	15.10365495115250
C	1.76153581350978	9.82847624152030	17.29904516747125
C	2.08041793924460	7.54301876779721	13.58388922971879
C	2.77584426887600	5.46137806379373	13.94787177912245
C	3.38811349335205	5.94735305362574	18.19152043930616
H	0.87987442522366	11.56987848453735	14.62611267871374
H	1.04633713992892	11.94714768003696	17.29436980450862
H	1.86192151030345	7.29871714716070	11.35465427418438

H	2.70302697321518	4.78523750230134	11.81627001025455
H	4.20950277967290	4.46860100188430	15.21247161671647
H	3.37545727779914	3.39957050847021	14.05698005757358
H	2.52126957866340	3.99871908174735	15.50435833255227
H	1.44941130287811	8.62769411824608	19.05719464723463
H	1.75381581208988	10.35480223734049	19.38345567461657
H	3.09330793749687	9.28350705883767	18.90335997475561
H	1.38691794532729	9.21170926197880	11.70449265022952
H	1.85930055442369	10.65118986590863	12.62561176733363
H	0.20949898587385	10.02032113325770	12.75874350976033
H	5.15407165999872	6.15079979297233	16.91572999283787
H	4.81051194494125	7.59959936897722	18.00671928539968
H	3.36743460833629	4.85608567133758	18.09806724350686
H	3.02929994903016	6.28275661325559	19.17074011201265

References

1. Oh, J.; Zheng, S.-L.; Carsch, K. M.; Latendresse, T. P.; Casaday, C. E.; Campbell, B. M. and Betley, T. A., An Open-Shell Fe^{IV}Nitrido. *J. Am. Chem. Soc.* **2025**, *147*, 3174.

2. Heurich, T.; Nesterov, V.; Schnakenburg, G.; Qu, Z. W.; Grimme, S.; Hazin, K.; Gates, D. p.; Engeser, M.; Streubel, R., Strong Evidence of a Phosphanoxyl Complex: Formation, Bonding, and Reactivity of Ligated Phosphorus Analogues of Nitroxides. *Angew. Chem., Int. Ed.* **2016**, *55*, 14439-14443.

3. Chavez, I.; Alvarez-Carena, A.; Molins, E.; Roig, A.; Maniukiewicz. W.; Arancibia. A.; Arancibia, V.; Brand, H.; Manrıquez, J. M., Selective Oxidation of Organometallic Compounds Containing a Stabilising Anion of Highly Reactive Cation: $(3,5(CF_3)_2C_6H_3)_4^-)Cp_2Fe^+$ and $(3,5(CF_3)_2C_6H_3)_4^-)Cp^*_2Fe^+$, *J. Organomet. Chem.* **2000**, *600*, 126-132.

4. Job, R.; Earl, R., Iron(0) Oxidation by Iodine in Tetrahydrofuran: A Facile Synthesis of Anhydrous Iron(II) Iodide. *Inorg. Nucl. Chem. Letters* **1979**, *15*, 81.

5. Ittel, S. D.; English, A. D.; Tolman, C. A.; Jesson, J. P., Synthesis of Iron(II) Phosphite Complexes. *Inorg. Chimica. Acta.* **1979**, *33*, 101.

6. Vela, J.; Vaddadi, S.; Cundari, T. R.; Smith, J. R.; Gregory, E. A.; Lachicotte, R. J.; Flaschenriem, C. J.; Holland, P. L., Reversible Beta-Hydrogen Elimination of Three-Coordinate Iron(II) Alkyl Complexes: Mechanistic and Thermodynamic Studies. *Organometallics* **2004**, *23*, 5226-5239.

7. Holland, P. L., Electronic Structure and Reactivity of Three-Coordinate Iron Complexes. *Acc. Chem. Res.* **2008**, *41*, 905-914.

8. Eckert, N. A.; Smith, J. M.; Lachiotte, R. J.; Holland, P. L., Low-Coordinate Iron(II) Amido Complexes of β-Diketiminates: Synthesis, Structure, and Reactivity. *Inorg. Chem.* **2004**, *43*, 3306-3321.

9. Bain, G. A.; Berry, J. F., Diamagnetic Constant and Pascal's Constants. *J. Chem. Ed.* **2008**, *85* (4), 532.

10. Hagen, W.R. Biomolecular EPR Spectroscopy, CRC Press, Boca Raton, Fl, USA, 2009.

11. Otsuka, F. S.; García Otaduy, M. C.; Nascimento, O. R.; Garrido Salmon, C. E.; Huber, M. Challenges of Continuous Wave EPR of Broad Signals – The Ferritin Case. *Appl. Magn. Reson.* **2024**, *55*, 1605.

12. Carsch, K. M.; DiMucci, I. M.; Iovan, D. A.; Li, A.; Zheng, S.-L.; Titus, C. J.; Lee, S. J.; Irwin, K. D.; Nordlund, D.; Lancaster, K. M.; Betley, T. A., Syntheses of a Copper-Supported Triplet Nitrene Complex Pertinent to Copper-Catalyzed Amination. *Science* **2019**, *365*, 1138.

13. Rosenthal, A. J.; Devillard, M.; Miqueu, K.; Bouhadir, G.; Bourissou, D., A Phosphine-Coordinated Boron-Centered Gomberg-Type Radical, **2015**, *54*, 9198-9202.

14. Bruker. APEX3, Bruker AXS Inc., Madison, Wisconsin, USA, **2015**.

15. Krause, L; Herbst-Irner, R.; Sheldrick, G. M.; Stalke, D., Comparison of Silver and Molybdenum Microfocus X-ray Sources for Single-Crystal Structure Determination. *J. Appl. Crystallogr.* **2015**, *48*, 3-10.

16. Sheldrick, G., SHELXT – Integrated Space-Group and Crystal-Structure Determination. *Acta Cryst. Sect. A* **2015**, *71*, 3-8.

17. Sheldrick, G., Crystal Structure Refinement with SHELXL. *Acta Crystallogra. A* **2015**, *71*, 3-8.

18. Dolomanov, O. V.; Bourhis, L. J.; Gildea, R. J.; Howard, J. A. K.; Puschmann, H., OLEX2: A complete Structure Solution, Refinement and Analysis Program. *J. Appl. Crystallogr.* **2009**, *42*, 339-341.

19. Ramadhar, T. R.; Zheng, S.-L.; Chen, Y.-S.; Clardy, J., Analysis of rapidly synthesized guest-filled porous complexes with synchrotron radiation: practical guidelines for the crystalline sponge method. *Acta Crystallogr., Sect. A* **2015**, *71,* 46.
20. Spek, A., Single-Crystal Structure Validation with Program PLATON. *J. Appl. Crystallogr.* **2003**, *36*, 7-13.
21. Bruker. Twinabs, Bruker AXS Inc., Madison, Wisconsin, USA, **2001**.
22. Neese, F., Software update: The ORCA program system—Version 5.0., *Wiley Interdisciplinary Reviews: Computational Molecular Science*, **2022**, *12*, e1606.
23. Lee, C.; Yang, W.; Parr, R. G., Development of the Colle-Salvetti correlation-energy formula into a functional of the electron density. *Phys. Rev. B* **1988**, *37,* 785
24. Schäfer, A.; Horn, H.; Ahlrichs, R., Fully optimized contracted Gaussian basis sets for atoms Li to Kr. *J. Chem. Phys.* **1992**, *97,* 2571.
25. Schäfer, A.; Huber, C.; Ahlrichs, R., Fully optimized contracted Gaussian basis sets of triple zeta valence quality for atoms Li to Kr. *J. Chem. Phys.* **1994**, *100,* 5829.
26. Neese, F.; Wennmohs, F.; Hansen. A.; Becker, U., Efficient, approximate and parallel Hartree-Fock and hybrid DFT calculations. A 'chain-of-spheres' algorithm for the Hartree-Fock exchange. *Chem*. *Phys*. **2009**, *356*, 98.
27. Grimme, S.; Ehrlich, S.; Goerigk, L., Effect of the damping function in dispersion corrected density functional theory. *J. Comput. Chem.* **2011**, *32,* 1456.
28. Sinnecker, S.; Rajendran, A.; Klamt, A.; Diedenhofen, M.; Neese, F. Calculation of Solvent Shifts on Electronic g-Tensors with the Conductor-Like Screening Model (COSMO) and Its Self-Consistent Generalization to Real Solvents (Direct COSMO-RS). *J. Phys. Chem. A*, **2006**, *110*, 2235
29. Neese, F. Prediction and interpretation of the 57Fe Isomer Shift in Mössbauer Spectra by Density Functional Theory. *Inorg. Chim. Acta.* **2002**, *337*, 181-192.
30. Gütlich, P.; Bill, E.; Trautwein, A. X. Mössbauer Spectroscopy and Transition Metal Chemistry: Fundamentals and Applications. *Springer Science & Business Media*, **2010**.
31. Iovan, D. A.; Betley, T. A. Characterization of Iron-Imido Species Relevant for *N*-Group Transfer Chemistry. *J. Am. Chem. Soc.* **2016**, *138*¸1983.
32. Nieto, I.; Ding, F.; Bontchev, R. P.; Wang, H.; Smith, J. M. Thermodynamics of Hydrogen Atom Transfer to a High-Valent Iron Imido Complex. *J. Am. Chem. Soc* **2008,** *130*, 2716-2717.
33. Johnson, E. J.; Kleinlein, C.; Musgrave, R. A.; Betley, T. A. Diiron Oxo Reactivity in a Weak-Field Environment. *Chem. Sci.* **2019**, *10*, 6304-6310.
34. Kutt, A.; Selberg, S.; Kaljurand, I.; Tshepelevitsh, S.; Heering, A.; Darnell, A.; Kaupmess, K.; Piirsalu, M.; Leito, I., p*K*a Values in Organic Chemistry – Making Maximum Use of the Available Data. *Tetraheron Lett.* **2018**, *59*, 3738.
35. Zhurko, G.; Zhurko, D., Chemcraft - Graphical Software for Visualization of Quantum Chemistry Computations. http://www.chemcraftprog.com/
36. Tain, Z.; Kass, S. R., Carbanions in the Gas Phase. *Chem. Rev.* **2013**, *113*, 6986-7010.
